# Effectiveness of ITS and sub-regions as DNA barcode markers for the identification of Basidiomycota (Fungi)

**DOI:** 10.1186/s12866-017-0958-x

**Published:** 2017-02-23

**Authors:** Fernanda Badotti, Francislon Silva de Oliveira, Cleverson Fernando Garcia, Aline Bruna Martins Vaz, Paula Luize Camargos Fonseca, Laila Alves Nahum, Guilherme Oliveira, Aristóteles Góes-Neto

**Affiliations:** 1 Centro Federal de Educação Tecnológica de Minas Gerais (CEFET-MG), Departamento de Química, 30.421-169 Belo Horizonte, MG Brazil; 2 Fundação Oswaldo Cruz (FIOCRUZ), Centro de Pesquisas René Rachou – CPqRR, 30190-002 Belo Horizonte, MG Brazil; 3 Universidade Federal de Minas Gerais, Departamento de Microbiologia, Av. Antônio Carlos, Belo Horizonte, 6627, 31270-901 MG Brazil; 4 Faculdade de Minas (FAMINAS), 66055-090 Belo Horizonte, MG Brazil; 5 Faculdade Promove de Tecnologia, 30140-061 Belo Horizonte, MG Brazil; 6 Instituto Tecnológico Vale, 66055-090 Belém, PA Brazil

**Keywords:** ITS, ITS1, ITS2, Probable correct identification, Barcode gap, Basidiomycota

## Abstract

**Background:**

Fungi are among the most abundant and diverse organisms on Earth. However, a substantial amount of the species diversity, relationships, habitats, and life strategies of these microorganisms remain to be discovered and characterized. One important factor hindering progress is the difficulty in correctly identifying fungi. Morphological and molecular characteristics have been applied in such tasks. Later, DNA barcoding has emerged as a new method for the rapid and reliable identification of species. The nrITS region is considered the universal barcode of Fungi, and the ITS1 and ITS2 sub-regions have been applied as metabarcoding markers. In this study, we performed a large-scale analysis of all the available Basidiomycota sequences from GenBank. We carried out a rigorous trimming of the initial dataset based in methodological principals of DNA Barcoding. Two different approaches (PCI and barcode gap) were used to determine the performance of the complete ITS region and sub-regions.

**Results:**

For most of the Basidiomycota genera, the three genomic markers performed similarly, i.e., when one was considered a good marker for the identification of a genus, the others were also; the same results were observed when the performance was insufficient. However, based on barcode gap analyses, we identified genomic markers that had a superior identification performance than the others and genomic markers that were not indicated for the identification of some genera. Notably, neither the complete ITS nor the sub-regions were useful in identifying 11 of the 113 Basidiomycota genera. The complex phylogenetic relationships and the presence of cryptic species in some genera are possible explanations of this limitation and are discussed.

**Conclusions:**

Knowledge regarding the efficiency and limitations of the barcode markers that are currently used for the identification of organisms is crucial because it benefits research in many areas. Our study provides information that may guide researchers in choosing the most suitable genomic markers for identifying Basidiomycota species.

**Electronic supplementary material:**

The online version of this article (doi:10.1186/s12866-017-0958-x) contains supplementary material, which is available to authorized users.

## Background

Fungi are one of the major eukaryotic lineages that are equivalent in species number to animals but exceed that of plants [1]. Fungi are among the most important organisms in the world because of their vital roles in decomposition, nutrient cycling, and obligate mutualistic symbioses with plants, algae, and cyanobacteria [2]. Fungi also have great economic importance for industrial fermentation, pharmaceutical, and biotechnological industries [3]. They may also cause food spoilage and diseases in plants and animals [4]. The diversity of activities is reflected in the high number of taxa, morphologies, habitats, and life strategies used by this group of organisms. Further studies are necessary to better understand their complex interactions with other organisms and environments.

The phylum Basidiomycota is the second largest of the Fungi kingdom and comprises approximately 30% of all described fungal species [5]. This diverse phylum includes primarily macroscopic but also microscopic fungi, such as mushrooms and basidiomycotan yeasts, respectively [6, 7]; saprotrophs, such as wood-decaying fungi [8]; pathogens of plants [3] and animals [9, 10]; and mycorrhizal symbionts [11]. Basidiomycota species are grouped into the following subphyla: Agaricomycotina, Pucciniomycotina, and Ustilaginomycotina. The first is the largest subphylum with approximately one-third of all described fungal species [5, 12]. Thus, a substantial amount of the data that is currently available on diversity, distribution, and sequencing has targeted Agaricomycotina, particularly in the orders of Agaricales, Polyporales, and Boletales. This subphylum is primarily composed of wood decayers, litter decomposers, and ectomycorrhizal fungi, as well as pathogens and poisonous, hallucinogenic, or edible species [13].

The identification of fungi at the species level is critical to many research areas, such as health sciences and agriculture, where the determination of causal agents of diseases is central to the definition of the suitable treatment, elucidation of outbreaks, and transmission mechanisms [14, 15]. Furthermore, the understanding of the specific roles of microorganisms in an ecosystem, their abundance, and their community composition in ecological and biodiversity studies can only be attained through their reliable identification [16]. However, discovering and describing all extant fungal species appears challenging. According to the *Dictionary of Fungi*, only approximately 100,000 species have been described thus far [12], and the estimated diversity ranges from 1.5 to 5.1 million [1, 17, 18].

Morphological characteristics are useful for species description; however, they may be limited because many macroscopic structures are produced infrequently and temporarily [19], and many taxa often harbor cryptic species complexes [20]. Molecular tools complementing morphological ones are very promising in identifying species and can be used to rapidly and reliably evaluate biological diversity. These markers have been applied to the identification of fungal species since the 1990s [21, 22]; however, the strategy based on the sequencing of standardized genomic fragments (DNA barcoding) was recognized afterwards [23]. The primary difference between molecular identification tools and the “DNA barcode” approach is that the latter involves the use of a standard DNA region that is specific for a taxonomic group. The use of a segment of the mitochondrial gene encoding the cytochrome c oxidase subunit I (COI) has been proposed for animals [24]. For plants, various loci combinations have been proposed [25]; however, a study conducted by the Consortium for the Barcode of Life (CBOL) Plant Working Group agreed that the combination of sequences of two plastid genes, *mat*K and *rbc*L, is the most promising plant barcode [26]. In 2012, the study conducted by Schoch and colleagues compared six DNA regions as promising universal barcodes for fungi. Mitochondrial COI and other protein-coding nuclear gene regions were excluded as potential markers for various reasons such as difficulties in amplifying DNA and insufficient variability. The nuclear ribosomal RNA internal transcribed spacer (ITS) region exhibited the highest probability of correct identification (PCI) for a wide number of fungal lineages analyzed and the most clearly defined barcode gap [27]. Since then, the ITS region has been accepted as the standard barcode marker for fungi. However, a thorough study of ITS sequences in the International Nucleotide Sequence Database (INSD: GenBank, EMBL and DDBJ) revealed that this region is not equally variable in all groups of fungi [28]. Notably, for some genera of Ascomycota, including *Alternaria* [29], *Aspergillus* [30], *Cladosporium* [31], *Penicillium* [30], and *Fusarium* [32], identification using the ITS barcode has been difficult.

One advantage of using the ITS region as a standard marker is that most fungal species have been identified based on this genomic region. GenBank [33] is the most comprehensive and widely used sequence repository in the field. A database specific for fungal sequences, the UNITE (User-friendly Nordic ITS Ectomycorrhiza Database) has been developed [34]. UNITE aims to unify the fungal taxonomic identification and correct the annotations associated with the taxonomic names to the greatest extent possible. The Barcode of Life Data System - BOLD [35] represents another bioinformatics platform; however, fungi remain underrepresented in it. BOLD supplies tools for the storage, quality warranty, and analysis of specimens and sequences to validate a barcode library. To obtain a barcode status on BOLD, sequences must fulfill some requirements, such as voucher data, collection record, and trace files. In the last few years, the scientific community has observed the rapid improvement of DNA sequencing technologies and the huge volume of data generated. Trimming and identifying this enormous amount of data requires bioinformatics tools, such as automated pipelines and various programs. However, the success of the analysis greatly depends on the correct taxonomic identification of sequences. Specifically, in the case of publicly available fungal ITS sequences, the reliability and technical quality vary significantly [34, 36]. Schoch and colleagues [27] estimate that only approximately 50% of the ITS sequences that are deposited in public databases are annotated at the species level. Moreover, Nilsson and colleagues [37] estimated that more than 10% of these fully identified fungal ITS sequences are incorrectly annotated at the species level. On the other hand, excellent initiatives, such as UNITE and that from NCBI that include a tool which allows flagging a GenBank sequence with type material [38] have emerged to minimize such a problem.

The ITS region comprises two sections (ITS1 and ITS2) that flank the conserved 5.8S region. The identification of multiple species from environmental samples (the DNA metabarcode) requires the use of high-throughput technologies, which may have limitations in sequencing read lengths [39]. For such approaches, only a portion of the ITS region is usually used, the ITS1 or the ITS2. The efficiency of these sub-regions in the identification of species in many fungal lineages has been evaluated, and some authors claim that ITS1 is more variable than ITS2 [28, 40–42]. Others have found opposite results [43] or that both the sub-regions are suitable as metabarcoding markers [44, 45]. In a recent work, Guarnica and colleagues [46] demonstrated that the ITS1 region is not more variable than the ITS2 region for *Cortinarius*. Furthermore, the complete ITS region is highly effective in discriminating among species in this highly sampled genus of Basidiomycota.

In the present study, an extensive comparative analysis based on the probability of correct identification (PCI) and barcode gap analyses was performed using a trimmed dataset composed of all Basidiomycota sequences deposited in GenBank. We evaluated the most widely used genomic markers for Fungi (the complete ITS region and the ITS1 and ITS2 sub-regions) to determine which is the most suitable for the identification of Basidiomycota species. Issues related to the need of additional molecular barcode markers as well as the taxonomic complexities within the subphyla are discussed.

## Methods

### Data acquisition and filtering

In this study, only sequences with complete nuclear ribosomal ITS from permanent collections whose taxonomic identifications were curated by specialists (*voucher* specimens) and deposited in GenBank [33] were used. Taxonomic information regarding the specimens was enriched, when available, from the UNITE database [34]. This step was used after downloading sequences from GenBank and before logical and quality filters were applied. For this enrichment, we firstly downloaded the FASTA sequence files from UNITE, and then we generated a tabular file with the UNITE data, keeping only the access numbers that corresponded to our specimens. Then, we retrieved the information related to sampling area and fungal classification from UNITE. Finally, we used the UNITE information to enrich the GenBank information.

Quality filters removed sequences with one or more IUB/IUPAC ambiguous characters, and logic filters ensured that the sequences were suitable for DNA barcode study in accordance with Barcode of Life recommendations (http://www.barcodeoflife.org/). The first logic filter guaranteed that only sequences identified at the species level were maintained in the database. Therefore, species with inconclusive names ('sp.', 'aff.', 'cf.', and 'uncultured') were removed. FungalITSExtractor [47] was used to guarantee that only sequences with complete ITS regions were maintained in the database. More than 99% of fungal complete ITS sequences deposited in GenBank are shorter than 800 or longer than 400 pb; thus all sequences outside of this interval were excluded from the dataset. The low representativeness together with the potential to distort the multiple sequence alignment justified this filter. Only species with specimens collected from at least three different localities were included to guarantee that only distinct and geographically distant specimens were evaluated and to avoid the possibility of working with genetically identical specimens. The list of all species used to perform the analyses of this study is provided (Additional file 1). All filters were performed using custom scripts written in the Perl programming language and are available upon request. The FungalITSExtractor software was used to identify and extract the ITS, ITS1, and ITS2 regions.

### Data Analysis

The ITS, ITS1, and ITS2 datasets were partitioned in several sub-datasets, each containing sequences belonging to only one genus. Sequences from each sub-dataset were aligned using MUSCLE (version 3.8.31) with default parameters [48]. Distance matrices were generated using an uncorrected p-distance because it is simple and without any biological assumptions [49]. To evaluate the discriminative power of the three genomic markers, the probability of correct identification (PCI) was calculated as the ratio of species successfully identified per total number of species. A species was considered successfully identified if the minimum interspecific distance was larger than its maximum intraspecific distance [50]. Custom Perl scripts were written to calculate the distance matrices and the PCI values. Boxplots were plotted in R language.

Two statistical analyses were performed to graphically represent the data, a scatter plot and a dot plot. The scatter plot aimed to evaluate the correlations between the PCI values for the genomic regions pairwise combinations (ITS versus ITS1, ITS versus ITS2, and ITS1 versus ITS2), and the Spearman correlation coefficient was determined. The dot plot was used to compare the PCI with the barcode gap analyses. For this purpose, the PCI values for the four groups previously defined from the barcode gap analyses (Groups 1 to 4) were represented for each genomic region. All data and graphics were generated using Minitab (Minitab Statistical Software, version 17.3.1, State College, Pennsylvania: Minitab Inc., 2016).

## Results

Our primary dataset was comprised of all complete ITS (ITS1 + 5.8S + ITS2) sequences of Basidiomycota and consisted of 37,699 sequences. The exclusion of sequences without the field ‘specimen_voucher’ in GenBank file reduced the number to 37,342. Removing sequences with ambiguous nucleotides led to 27,459 sequences, and removing sequences with inconclusive species names resulted in 21,238 sequences. After applying FungalITSExtractor, 19,578 sequences remained. ITS sequences with less than 400 bp and more than 800 bp were also excluded from the dataset, as well as ITS1 and ITS2 sequences less than 100 bp, leaving 19,149 sequences. The last filter was used to ensure that only species with at least three sequences collected from different geographic locations were retained in the dataset. Because most of the sequences did not include information regarding their origin, our final dataset had this number reduced to 7,731 sequences from 112 countries from six continents. This dataset was used to perform all subsequent analyses and represented three subphyla, five classes, 25 orders, 73 families, 211 genera, and 936 species (Additional file 2). This dataset has 167 sequences whose DNA were originated from biological specimens considered as type material. Many sequences from type materials were not included in our dataset only because they did not pass in quality and logic filters.

**Fig. 1:**
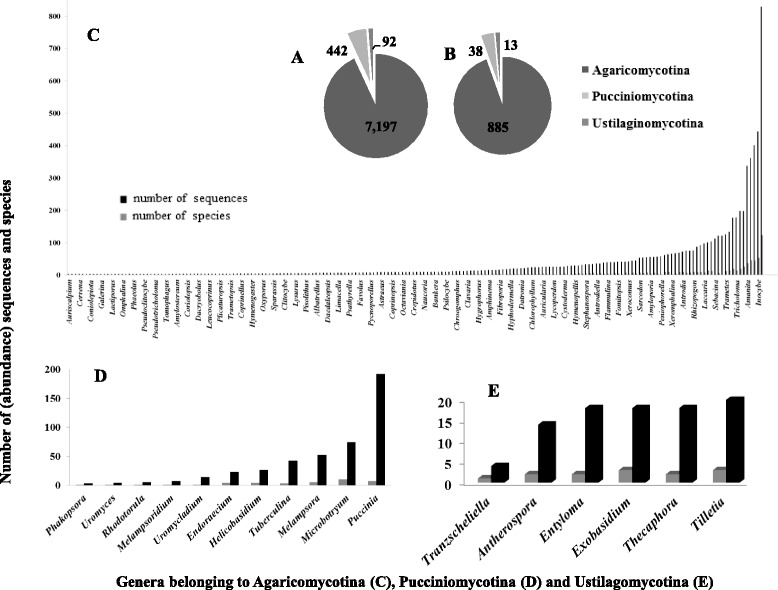
Pie charts represent abundance (number) of sequences (**a**) and species (**b**) for the three subphyla represented in the dataset used in this study. The histograms show the number of species and sequences distributed for genera belonging to Agaricomycotina (**c**), Pucciniomycotina (**d**) and Ustilagomycotina (**e**) phylum

Although GenBank is known to be the most complete available public database, the amount of sequences is biased in our trimmed dataset as follows: 93.1% (7,197 sequences) belong to species of Agaricomycotina, whereas only 5.7% (442 sequences) come from Pucciniomycotina and 1.2%. (92 sequences) from Ustilaginomycotina. When other taxonomic ranks were analyzed, a similar distribution was observed with the vast majority of species belonging to Agaricomycotina (Fig. 1). Inside the subphyla, the imbalance in the amount of sequences is also enormous. For example, in Agaricomycotina, we found very well represented taxa (such as *Cortinarius*, with 829 sequences from 124 species) and others that were poorly represented (such as *Auriscalpium*, with only one species represented by three sequences). Most of the genera from the Agaricomycotina dataset were underrepresented; 126 of 194 had 20 or fewer sequences, whereas only 16 genera were represented by more than 100 sequences (Fig. 1 and Additional file 2).

The probability of correct identification (PCI) for the three genomic regions under study was estimated using our trimmed dataset (7,028 sequences from 113 genera). The number of genera analyzed decreased compared with the original dataset (211 genera) because we needed at least two species to estimate intraspecific and interspecific distances. Moreover, the sequences identified as type material are distributed in 27 distinct genera (23.9% of total) (Additional File 3), and only 25 sequences with RefSeq accessions interchangeably with GenBank numbers were identified (Additional File 4). This represented approximately only 0.36% of the sequences that comprised the dataset used to estimate PCI and barcode gap indices.

**Fig. 2:**
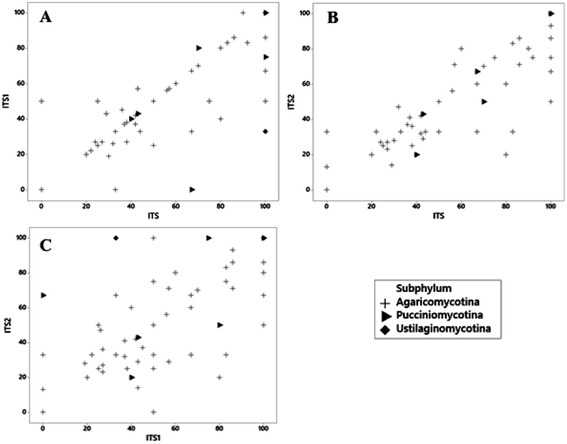
Pairwise correlations (**a**, ITS X ITS1, **b**, ITS X ITS2 and **c**, ITS1 X ITS2) between PCI values of all genera from our dataset

The mean PCI value for the complete ITS region was 63%, those for the sub-regions were slightly smaller as follows: 59% for ITS1 and 58% for ITS2. For the ITS region, 53.1% of the genera had PCI values higher than the mean, whereas for ITS1 and ITS2, these values were 46% and 48%, respectively (Table 1). The pairwise correlation between the three markers (ITS versus ITS1, ITS versus ITS2 and ITS1 versus ITS2) was estimated considering the PCI values of all genera composing the dataset. The comparisons between complete ITS and the sub-regions showed most of the data on or near the regression line, meaning that most of the PCI values were similar for the genera (Spearman correlation factor for ITS versus ITS1 = 0.8825 and for ITS versus ITS2 = 0.9102). When the sub-regions were associated (ITS1 versus ITS2), the distribution of data had a different profile and a lower correlation was observed (0.8158) (Fig. 2). The pairwise correlation between the genomic regions was carried out at the subphylum level; however, there were no observable patterns at this taxonomic level.

**Table 1:** Probable Correct Identification (PCI) values (%) for all of the Basidiomycota genera from our trimmed dataset. The PCI values were estimated for the three genomic regions studied, the complete ITS region (ITS1 + 5.8S + ITS2) and the sub-regions ITS1 and ITS2

Genera	ITS (ITS1 + 5.8S+ ITS2)	ITS1	ITS2
*Agaricus*	100	100	100
*Alnicola*	44	33	33
*Amanita*	24	27	27
*Amyloporia*	25	25	25
*Antherospora*	100	100	100
*Antrodia*	70	70	70
*Antrodiella*	75	50	75
*Armillaria*	20	20	20
*Auricularia*	80	40	60
*Boletus*	32	26	47
*Butyriboletus*	67	33	67
*Calvatia*	0	0	0
*Ceriporiopsis*	100	100	100
*Chlorophyllum*	100	100	100
*Chroogomphus*	50	50	50
*Clavaria*	100	100	100
*Clavulina*	33	33	33
*Collybia*	50	50	50
*Coprinopsis*	50	50	50
*Cortinarius*	36	45	37
*Crepidotus*	50	50	50
*Cystoderma*	50	50	33
*Cystodermella*	100	100	100
*Datronia*	0	50	0
*Endoraecium*	100	100	100
*Entoloma*	100	86	93
*Entyloma*	100	100	100
*Exobasidium*	100	100	100
*Favolus*	100	100	100
*Fibroporia*	100	100	67
*Flammulina*	100	50	75
*Fomitopsis*	50	25	50
*Fuscoporia*	100	100	100
*Ganoderma*	43	43	43
*Geastrum*	100	67	67
*Gloeophyllum*	100	100	100
*Gymnopilus*	100	100	50
*Gymnopus*	67	67	60
*Hebeloma*	42	37	32
*Helicobasidium*	100	75	100
*Hohenbuehelia*	0	0	0
*Hydnellum*	56	56	56
*Hydnum*	50	50	50
*Hygrocybe*	0	0	0
*Hygrophorus*	67	67	33
*Hymenopellis*	100	100	100
*Hyphoderma*	60	60	80
*Hyphodermella*	100	100	100
*Hypholoma*	0	0	0
*Inocybe*	30	19	28
*Laccaria*	0	0	13
*Lactarius*	37	37	41
*Lactifluus*	50	50	50
*Lentinellus*	43	43	29
*Lentinus*	57	57	71
*Lepiota*	92	83	75
*Lepista*	100	100	100
*Leucoagaricus*	90	100	80
*Leucopaxillus*	100	100	100
*Lycoperdon*	100	100	100
*Lyomyces*	100	100	100
*Lyophyllum*	100	100	67
*Macrolepiota*	50	50	33
*Megacollybia*	83	83	33
*Melampsora*	40	40	20
*Melanoleuca*	38	38	25
*Microbotryum*	70	80	50
*Mucidula*	0	0	0
*Mycena*	22	22	33
*Neofavolus*	100	100	100
*Octaviania*	100	100	100
*Oligoporus*	100	100	100
*Parasola*	100	67	67
*Paxillus*	0	0	33
*Peniophorella*	50	50	50
*Phaeocollybia*	0	0	0
*Phanerochaete*	67	67	67
*Phellinus*	100	33	67
*Phellodon*	86	86	71
*Piloderma*	50	50	50
*Pisolithus*	0	0	0
*Pleurotus*	29	43	14
*Pluteus*	27	27	23
*Polyporus*	100	100	86
*Porodaedalea*	100	100	100
*Postia*	50	50	50
*Psathyrella*	100	100	100
*Psilocybe*	100	100	100
*Puccinia*	43	43	43
*Pycnoporellus*	100	100	100
*Ramaria*	33	33	33
*Resinicium*	100	100	100
*Rhizopogon*	27	27	27
*Rhodocollybia*	100	100	100
*Rigidoporus*	100	100	50
*Russula*	38	27	36
*Sarcodon*	86	86	86
*Scleroderma*	33	33	33
*Sebacina*	33	0	33
*Stephanospora*	80	80	20
*Strobilurus*	67	67	67
*Suillus*	83	83	83
*Thecaphora*	100	100	100
*Thelephora*	0	0	0
*Tilletia*	100	33	100
*Tomentella*	25	50	25
*Trametes*	42	42	42
*Tricholoma*	43	57	29
*Tricholomopsis*	100	100	100
*Tuberculina*	67	0	67
*Vuilleminia*	33	33	33
*Xerocomus*	100	50	100
*Xeromphalina*	100	100	50

**Fig. 3:**
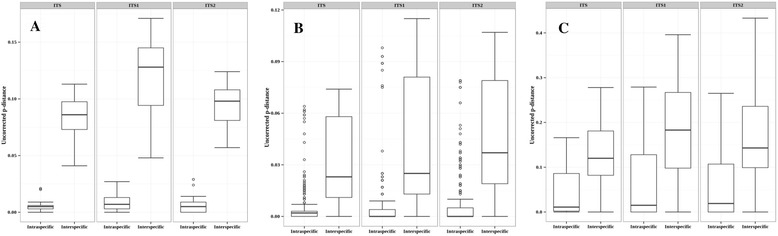
Examples of the barcode gap performance classifications used in this study. **a**. Clear barcode gap (identification performance classified as *good*) for the genera *Agaricus*, **b**. *Intermediate* separation between the intra- and interspecific distances for *Hebeloma* and **c**. A *poor* barcode gap for *Lactarius*

Based on the analysis of the barcode gaps, we assessed and compared the efficiency of the three genomic markers for the identification of Basidiomycota. Thus, we classified the marker performance into the following three distinct categories: *good, intermediate*, or *poor.* When a clear barcode gap was present (e.g., *Agaricus*, Fig. 3a), we conventionally stated that the identification was *good*, even if outliers were overlapping. The genomic markers were considered *intermediate* if the whiskers from an intraspecific distance overlap those from an interspecific distance (e.g., *Hebeloma,* Fig. 3b), and *poor* if the boxes overlap or the intraspecific distance values were superior to those of interspecific distance (e.g., *Lactarius,* Fig. 3c). For most of the genera (91.5%) evaluated, the three genomic regions performed similarly, i.e., when the identification is *good* for one region, it is also *good* for the others. The same occurred when the performance was *intermediate* or *poor*. However, for some genera, we found some genomic regions with superior identification performance than others. For instance, the complete ITS had a clearer barcode gap for the genera *Auricularia, Flammulina*, *Lentinellus*, *Microbotryum*, *Parasola*, and *Tuberculina* compared with the ITS1 or ITS2 sub-regions. ITS1 performed better than the other regions in the identification of species from the genera *Hygrophorus* and *Stephanospora,* as well as ITS2 for the species belonging to the genera *Amanita, Amyloporia*, *Fomitopsis*, *Scleroderma*, and *Strobilurus* (Table 2, Group 2)*.* In some instances, one of the three genetic markers performed worse than the other(s). The ITS1 sub-region is not sufficient to differentiate the species of the genera *Collybia* and *Pleurotus*, and the ITS2 is not a good marker for *Sebacina*, *Hydnellum*, or *Vuilleminia*. Finally, it is important to note that for 11 out of the 113 genera evaluated (*Botyriboletus, Clavulina, Crepidotus, Hohenbuehelia, Hydnum, Laccaria*, *Lactarius, Mucidula*, *Peniophorella*, *Phaeocollybia*, and *Pisolithus*), none of the complete ITS, ITS1 or ITS2 sub-regions could be used to differentiate the species based on the barcode gap analyses (Table 2, Group 4)*.* For a detailed classification of genera considering their barcodes, see Table 2 and Additional File 5, where the boxplots for all analyzed genera are shown.

**Table 2:** Grouping of Basidiomycota genera based on the barcode gap analyses (See Additional file 5 for more details)

Group 1	Group 2	Group 3	Group 4
Genera for which all the three genetic regions are *good* markers	Genera for which one or two genetic regions showed a clearer barcode gap and are recommended over the other (s)	Genera for which most of the genetic regions showed *intermediate* barcode gap and their use should be carefully evaluated	Genera for which all three genetic regions are *poor* markers
*Agaricus, Antherospora, Antrodia, Ceriporiopsis, Chroogomphus, Clavaria, Coprinopsis, Cystodermella, Datronia, Edoraecium, Entoloma, Entyloma, Exobasidium, Favolus, Fibroporia, Fuscoporia, Geastrum, Gloeophyllum, Helicobasidium, Hygrocybe, Hymenopellis, Hyphodermella, Lactifluus, Lepiota, Lepista, Leucopaxillus, Lycoperdon, Lyomyces, Lyophyllum, Melampsora, Neofavolus, Octaviania, Oligoporus, Phanerochaete, Phellinus, Phellodon, Piloderma, Polyporus, Porodaedalea, Psathyrella, Psilocybe, Puccinia, Pycnoporellus, Resinicium, Rhodocollybia, Russula, Sarcodon, Suillus, Telephora, Thecaphora, Tilletia, Tricholomopsis, Xerocomus, Xeromphalina*	*Amanita* (ITS2), *Amyloporia* (ITS2), *Antrodiella* (ITS and ITS2), *Auricularia* (ITS), *Calvatia* (ITS and ITS1), *Chlorophyllum* (ITS and ITS1), *Flammulina* (ITS), *Fomitopsis* (ITS2), *Ganoderma* (ITS and ITS1), *Gymnopilus* (ITS and ITS1), *Gymnopus* (ITS and ITS1), *Hygrophorus* (ITS1), *Lentinellus* (ITS), *Leucoagaricus* (ITS and ITS1), *Macrolepiota* (ITS and ITS1), *Megacollybia* (ITS and ITS1), *Microbotryum* (ITS), *Parasola* (ITS), *Postia* (ITS and ITS2), *Rigidoporus* (ITS and ITS1), *Scleroderma* (ITS2), *Stephanospora* (ITS1), *Strobilurus* (ITS2), *Tuberculina* (ITS)	*Alnicola, Armillaria, Boletus, Collybia, Cortinarius, Cystoderma, Hebeloma, Hydnellum, Hyphoderma, Hypholoma, Inocybe, Lentinus, Melanoleuca, Mycena, Paxillus, Pleurotus, Pluteus, Ramaria, Rhizopogon, Sebacina, Tomentella, Tricholoma, Trametes, Vulleminia*	*Butyriboletus, Clavulina, Crepidotus, Hohenbuehelia, Hydnum, Laccaria, Lactarius, Mucidula, Peniophorella, Phaeocollybia, Pisolithus*

**Fig. 4:**
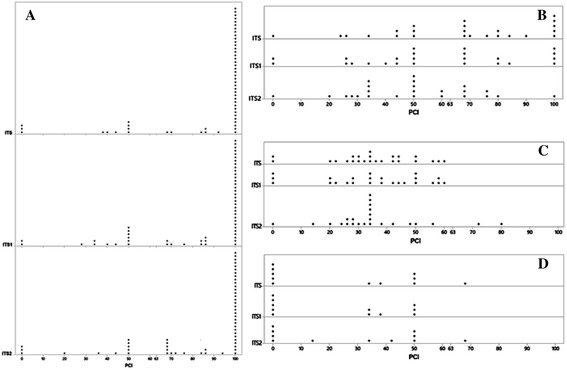
PCI values for the genera classified in the Group 1 (**a**), Group 2 (**b**), Group 3 (**c**) and Group 4 (**d**) are represented for the ITS, ITS1 and ITS2 genomic region

The results of barcode gap analyses were compared with the PCI values for each genus using a dot plot (Fig. 4). For the genera for which the three genomic markers were classified as *good* in barcode gap analyses (Group 1, Table 2), most of the genera exhibited PCI above the mean value (63%); however, some disagreements were found. Some genera within this group had a PCI equal to zero (*Datronia*, *Hygrocybe*, *Tecaphora*, and *Telephora*) or between 20 and 50% (*Chroogomphus*, *Coprinopsis*, *Lactifluus*, *Melampsora*, *Phellinus*, *Piloderma*, *Puccinia*, *Russula*, *Tilletia*, *Xerocomus*, and *Xeromphalina*) (Fig. 4a). When the group for which one or two genomic regions showing a clearer barcode gap (Group 2, Table 2) was compared with the PCI, most of the genera had a PCI below the mean value (Fig. 4b). When the group for which most of the genomic regions showed an intermediate barcode gap (Group 3, Table 2), only *Lentinus* and *Hyphoderma* had higher PCI than mean value (both for the ITS2 region, Fig. 4c). When the groups for which all three genomic regions were classified as *poor* markers considering the barcode gap (Group 4, Table 2), most of the genera also had a PCI below the mean value (Fig. 4b) with the exception of *Butyriboletus* (with a PCI value above the mean for ITS and ITS2, Fig. 4d).

## Discussion

The accepted DNA barcode for Fungi is the rDNA ITS region [27]. ITS is recognized as a fungal barcode because it is the most sequenced region of fungi and is routinely used for systematics, phylogenetics, and identification [51, 52]. In this study, we downloaded all complete ITS sequences of species belonging to the phylum Basidiomycota from GenBank. Although this is the most complete repository of available ITS sequences, misidentifications or low-quality sequencing have been encountered in this public database [37]. However, some authors think that it is unrealistic that future databases or even a barcode database could be more reliable than GenBank because misidentified sequences would be as common as they are currently and because *vouchers* will not be re-identified by taxonomic experts (for a wide discussion, see [53–55]). To overcome this drawback, logical and quality filters were applied to our original dataset to obtain the most reliable results possible. The restrictiveness in the filtering step aimed to create a high-quality dataset (accurate taxonomic annotation and presence of relevant metadata) that would meet the theoretical assumptions of the biological system of identification via DNA barcode and the principles recommended in BOLD Systems [24, 35].

More than 90% of our trimmed dataset belonged to the subphylum Agaricomycotina. This result is not surprising because it reflects the high diversity of this taxon compared with the other subphyla, which is widely mentioned in the literature [5, 12]. Kirk and colleagues [12] estimated that one-fifth of all known fungal species described belong to the Agaricomycete clade; this diversity is considered to be underestimated because new taxa are continually being described [1, 56]. This discrepancy in the amount of species and sequences from the subphyla may reflect a natural event or may occur due to the specific interests of the scientific community in Agaricomycotina species.

Some criteria have been traditionally used to test the DNA barcoding efficacy to classify and/or identify specimens at the species level, such as similarity measures, tree-based techniques, and identification based on direct sequence comparison [57, 58]. However, all of these approaches present several issues (see [55] for a detailed discussion). Similarity measures are generally used to cluster sequences in “molecular operational taxonomic units”; however, the choice of the threshold value for distinguishing intraspecific and interspecific distances is largely arbitrary [58, 59]. An important and acceptable measure of the efficacy of a genetic marker should reflect the probability of correctly identifying a species. This concept has emerged as the probability of correct identification (PCI) [50, 53, 55, 60]. However, there is no consensus for the definition and calculation of PCI, which currently embraces a broad class of measures. In this work, we assume the concept described by Hollingsworth and colleagues [50] in which the authors considered the “discrimination as successful if the minimum uncorrected interspecific p-distance involving a species was larger than its maximum intraspecific distance” to measure the PCI for each genus included in our dataset. Furthermore, the use of genetic distances enables the observation of the ‘barcoding gap’, which is possible by plotting the intraspecific and interspecific distances. Therefore, an ideal barcode marker would reveal intraspecific divergences lower than interspecific divergences [61].

In this study, we aimed to identify the most suitable genomic marker (complete ITS, ITS1 or ITS2) to identify fungal species belonging to Basidiomycota. Our findings, based on PCI and barcode gap analyses, indicated that for most of the genera, the three genomic regions perform similarly, i.e., when one genomic region was considered a good marker (a PCI above the mean value or the presence of a clear barcode gap) the other regions were also; the same was observed when the performance of genomic markers was considered insufficient. When the performance of the genomic markers was individually evaluated, barcode gap analyses provided a more optimistic view than PCI values. Approximately half of the genera exhibited PCI values lower than the mean (63%); however, the three genomic regions were classified as *good* for most of the genera (Table 2) when the barcode gap is taken into account. Accordingly, the comparison between barcode gap and PCI for each genus showed some disagreements. This was primarily observed for some of the genera that showed good identification performance using the barcode gap but had low PCI values (Fig. 4a). The opposite, i.e., high PCI values and poor identification performance via barcode gap, was observed for only one genus, *Botyriboletus* (Fig. 4d).

Initially, the low PCI values found for some genera (such as *Calvatia*, *Datronia*, *Hygrocybe, Hohenbuehelia*, *Hypholoma*, *Mucidula*, and *Pisolithus*) could be explained by dataset features, such as the low number of species (genera represented by sequences from only two species) and/or by the high number of outliers, which would have distorted the PCI estimates. Additionally, the taxonomy appears very complex for many of the genera for which the identification performance using ITS and sub-regions were insufficient. Taxonomy issues for two genera (*Hygrocybe* and *Thelephora*) for which PCI values were low and three genera (*Hypholoma, Phaeocollybia*, and *Pisolithus*) for which both PCI and barcode gap analyses proved that ITS, ITS1 and ITS2 are not sufficient markers for the identification of species are discussed below based on pertinent literature.

*Hygrocybe* species exhibit extremely high variability in the ITS region, with sequences diverging by more than 25%. Thus, the use of additional DNA barcode markers has been proposed to re-evaluate the taxonomy of this genus [62, 63]. Moreover, significant changes in the classification of *Hygrocybe,* such as its division, are expected [64].

The phylogenetic relationships between and within species of *Thelephora* are also doubtful with ITS. The existence of cryptic species was described, and the importance of integrating morphological and molecular data, as well as employing a meaningful number of samples for the accurate identification is highlighted [65]. *Hypholoma* has been poorly studied. However, a recent study based on the morphological and molecular aspects of *H. cinnabarinum* samples showed that this species is not a member of the genus *Hypholoma* but belongs instead to *Agaricus* [66]. The ecological role of *Phaeocollybia* is uncertain. Smith [67] argues that the genus harbors both saprobes and mycorrhiza formers. Singer [68] considered that members of the genus were not obligatorily ectomycorrhizal, whereas Norvell [69] presented evidence for the consideration of *Phaeocollybia* as a mycorrhizal genus. At the taxonomic level, the complexity remains, as may be exemplified in Norvell [70]. The author proposed the re-evaluation of the genus *Phaeocollybia* by revealing four new agaric species morphologically similar to *Phaeocollybia kauffmanii*. The wide genetic divergence among *Pisolithus* ITS sequences [71–73] indicates significant evolutionary divergence and suggests that this genus encompasses a species complex. This hypothesis was reinforced by Kope and Fortin [74] who separated three groups of *Pisolithus* using incompatibility tests and basidiospore spine morphology.

According to Bickford and colleagues [20], cryptic species are two or more distinct species that are erroneously classified under one species name. Large intraspecific genetic distances associated with morphological and geographical discrete differences have revealed a broad range of cryptic species for many organisms and habitats [75, 76]. Although our knowledge of fungal species remains limited, the presence of cryptic species inside the group is well recognized [20] and was subsequently described for many of the genera covered in this study.

The use of molecular techniques, primarily DNA sequences, generates information to re-evaluate classifications and provides more accurate species delimitations [77]. Currently, the utility of DNA barcoding is evident. However, a universal barcode for the clear identification of all fungal species does not appear feasible, and secondary barcodes for Fungi have already been proposed [78]. In addition to the known limitations of ITS barcodes for some genera of Ascomycota, our results indicated that for some genera of Basidiomycota, such as *Hygrocybe* and *Pisolithus,* additional barcode markers may contribute to a clear elucidation of the complex relationships between and within species. The failure to correctly identify biological species hampers the efforts of the scientific community to conserve, study, or utilize them. Future research in this field should include discovering characteristics that natural selection acts upon [20].

## Conclusions

Progress in many research areas fundamentally depends on the rapid and reliable identification of biological species. Most fungal diversity is unknown, and issues related to the conservation of these organisms are urgent; thus, studies related to species identification are crucial. Knowledge regarding the efficiency and limitations of the barcode markers that are currently used for specific groups of organisms optimize the work of many studies. Therefore, the present study contributes to the rational selection of barcode markers of species belonging to the phylum Basidiomycota.

## Additional files

Additional file 1: List of species used in this study, their accession and taxon ID in GenBank and taxonomic affiliations. (XLSX 345 kb)
Additional file 2: Number of species and sequences (specimens) recovered to each genus and their taxonomic affiliations. Data were compiled from our trimmed dataset. (DOCX 36 kb)
Additional file 3: List of genera with sequences originated from type specimens and their PCI values (ITS, ITS1, ITS 2) and groups according to the barcode gap analysis. (DOCX 61 kb)
Additional file 4: List of sequences with RefSeq accessions interchangeably with GenBank numbers. (DOCX 110 kb)
Additional file 5: Barcode gap of all the 113 genera studied for ITS, ITS1 and ITS2 genomic regions by plotting intra- and interspecific distances. (DOCX 9035 kb)

## Abbreviations

BOLD: The barcode of life data system
CBOL: Consortium for the barcode of life
INSD: International nucleotide sequence database
ITS: Nuclear ribosomal RNA internal transcribed spacer region
MUSCLE: Multiple sequence comparison by log-expectation
PCI: Probability of correct identification
UNITE: User-friendly nordic its ectomycorrhiza database

## References

[1] Blackwell M (2011). The fungi: 1, 2, 3 … 5.1 million species?. Am J Bot.

[2] Gadd GM, Jorgensen SE, Brian F (2013). Fungi and their role in the biosphere. Encyclopedia of ecology.

[3] Lane CR, Beales PA, Hughes KJD (2012). Fungal plant patogens.

[4] Fisher MC, Henk DA, Briggs CJ, Brownstein JS, Madoff LC, McCraw SL, Gurr SJ (2012). Emerging fungal threats to animal, plant and ecosystem health. Nature.

[5] Hibbett DS, Losos J (2014). Major events in the evolution of the Fungi. Princeton Guide to Evolution.

[6] Morin E (2012). Genome sequence of the button mushroom *Agaricus bisporus* reveals mechanisms governing adaptation to a humic-rich ecological niche. Proc Natl Acad Sci U S A.

[7] Stajich JE (2010). Insights into evolution of multicellular fungi from the assembled chromosomes of the mushroom *Coprinopsis cinerea* (*Coprinus cinereus*). Proc Natl Acad Sci U S A.

[8] Floudas D (2012). The Paleozoic origin of enzymatic lignin decomposition reconstructed from 31 fungal genomes. Science.

[9] Brown SM, Campbell LT, Lodge JK (2007). *Cryptococcus neoformans*, a fungus under stress. Curr Opin Microbiol.

[10] Dawson TL (2007). *Malassezia globosa* and *restricta*: breakthrough understanding of the etiology and treatment of dandruff and seborrheic dermatitis through whole-genome analysis. J Investig Dermatol Symp Proc.

[11] Martin F (2008). The genome of *Laccaria bicolor* provides insights into mycorrhizal symbiosis. Nature.

[12] Kirk PM, Cannon PF, Minter DW, Stalpers JA (2008). Dictionary of the fungi.

[13] Hibbett DS (2006). A phylogenetic overview of the agaricomycotina. Mycologia.

[14] Araujo R (2014). Towards the genotyping of fungi: methods, benefits and challenges. Cur Fung Infect Rep.

[15] McNeil M, Roberts AMI, Cockerell V, Mulholland V (2004). Real-time PCR assay for quantification of *tilletia caries* contamination of UK wheat seed. Plant Pathol.

[16] Peay KG, Kennedy PG, Bruns TD (2008). Fungal community ecology: a hybrid beast with a molecular master. Bioscience.

[17] Hawksworth DL (1991). The fungal dimension of biodiversity: magnitude, significance and conservation. Mycol Res.

[18] Hawksworth DL (2001). The magnitude of fungal diversity: the 1.5 million species estimate revisited. Mycol Res.

[19] Slepecky RA, Starmer WT (2009). Phenotypic plasticity in fungi: a review with observations on *Aureobasidium pullulans*. Mycologia.

[20] Bickford D, Lohman DJ, Sodhi NS, Ng PK, Meier R, Winker K, Ingram KK, Das I (2007). Cryptic species as a window on diversity and conservation. Trends Ecol Evol.

[21] White TJ, Bruns T, Lee S, Taylor J, Innis MA, Gelfand DH, Sninsky JJ, White TJ (1990). Amplification and direct sequencing of fungal ribosomal RNA genes for phylogenetics. PCR Protocols: a Guide to Methods and Applications.

[22] Bruns TD, White TJ, Taylor JW (1991). Fungal molecular systematics. Annu Rev Ecol Syst.

[23] Hollingsworth PM (2007). DNA barcoding: potential users. Genom Soc Pol.

[24] Hebert PD, Cywinska A, Ball SL, deWaard JR (2003). Biological identifications through DNA barcodes. Proc Biol Sci.

[25] Pennisi E (2007). Taxonomy. Wanted: a barcode for plants. Science.

[26] Hollingsworth PM (2009). A DNA barcode for land plants. Proc Natl Acad Sci U S A.

[27] Schoch CL, Seifert KA, Huhndorf S, Robert V, Spouge JL, Levesque CA, Chen W (2012). Fungal barcoding consortium. Nuclear ribosomal internal transcribed spacer (ITS) region as a universal DNA barcode marker for fungi. Proc Natl Acad Sci U S A.

[28] Nilsson RH, Kristiansson E, Ryberg M (2008). Intraspecific ITS variability in the kingdom fungi as expressed in the international sequence databases and its implications for molecular species identification. Evol Bioinforma.

[29] Pryor B, Michailides T (2002). Morphological, pathogenic, and molecular characterization of alternaria isolates associated with alternaria late blight of pistachio. Phytopathology.

[30] Skouboe P, Frisvadm J, Taylor J, Lauritsen D, Boysen M, Rossen L (1999). Phylogenetic analysis of nucleotide sequences from the ITS region of terverticillate *Penicillium* species. Mycol Res.

[31] Schubert K, Groenewald J, Braun U, Dijksterhuis J, Starink M, Hill C, Zalar P, de Hoog G, Crous P (2007). Biodiversity in the *Cladosporium* herbarum complex (Davidiellaceae, Capnodiales), with standardization of methods for *Cladosporium* taxonomy and diagnostics. Stud Mycol.

[32] O’Donnell K, Cigelnik E (1997). Two divergent intragenomic rDNA ITS2 types within a monophyletic lineage of the fungus Fusarium are nonorthologous. Mol Phylogenet Evol.

[33] Benson DA, Clark K, Karsch-Mizrachi I, Lipman DJ, Ostell J, Sayers EW (2015). GenBank. Nucleic Acids Res.

[34] Kõljalg (2013). Towards a unified paradigm for sequence-based identification of fungi. Mol Ecol.

[35] Ratnasingham S, Hebert PD (2013). A DNA-based registry for all animal species: the barcode index number (BIN) system. PLoS One.

[36] Bruns TD, Blackwell M, Edwards I, Taylor AF, Horton T, Zhang N (2008). Preserving accuracy in GenBank. Science.

[37] Nilsson RH, Ryberg M, Kristiansson E, Abarenkov K, Larsson KH, Koljalg U (2006). Taxonomic reliability of DNA sequences in public sequence databases: a fungal perspective. PLoS One.

[38] Federhen S (2014). Type material in the NCBI taxonomy database. Nucleic Acids Res.

[39] Cuadros-Orellana S, Leite LR, Smith A, Medeiros JD, Badotti F, Fonseca PL, Vaz ABM, Oliveira G, Góes-Neto A (2013). Assessment of fungal diversity in the environment using metagenomics: a decade in review. Fung Genom Biol.

[40] Mullineux T, Hausner G (2009). Evolution of rDNA ITS1 and ITS2 sequences and RNA secondary structures within members of the fungal genera *Grosmannia* and *Leptographium*. Fungal Genet Biol.

[41] Wang XC, Liu C, Huang L, Bengtsson-Palme J, Chen H, Zhang JH, Cai D, Li JQ (2015). ITS1: a DNA barcode better than ITS2 in eukaryotes?. Mol Ecol Resour.

[42] Ryberg M, Kristiansson E, Sjökvist E (2009). An outlook on the fungal internal transcribed spacer sequences in GenBank and the introduction of a web-based tool for the exploration of fungal diversity. New Phytol.

[43] Bazzicalupo AL, Bálint M, Schmitt I (2013). Comparison of ITS1 and ITS2 rDNA in 454 sequencing of hyperdiverse fungal communities. Fungal Ecol.

[44] Mello A, Napoli C, Murat C, Morin E, Marceddu G, Bonfante P (2011). ITS-1 versus ITS-2 pyrosequencing: a comparison of fungal populations in truffle grounds. Mycologia.

[45] Blaalid R, Kumar S, Nilsson RH, Abarenkov K, Kirk PM, Kauserud H (2013). ITS1 versus ITS2 as DNA metabarcodes for fungi. Mol Ecol Resour.

[46] Garnica S, Schön ME, Abarenkov K (2016). Determining threshold values for barcoding fungi: lessons from Cortinarius (Basidiomycota), a highly diverse and widespread ectomycorrhizal genus. FEMS Microbiol Ecol.

[47] Nilsson RH, Bok G, Ryberg M, Kristiansson E, Hallenberg N (2009). A software pipeline for processing and identification of fungal ITS sequences. Source Code Biol Med.

[48] Edgar RC (2004). MUSCLE: multiple sequence alignment with high accuracy and high throughput. Nucleic Acids Res.

[49] Russo CAM, Miyaki CY, Pereira SL, Matioli SR (2012). Reconstrução filogenética: Métodos geométricos. Biologia Molecular e Evolução.

[50] Hollingsworth ML (2009). Selecting barcoding loci for plants: evaluation of seven candidate loci with species-level sampling in three divergent groups of land plants. Mol Ecol Resour.

[51] Begerow D, Nilsson H, Unterseher M, Maier W (2010). Current state and perspectives of fungal DNA barcoding and rapid identification procedures. Appl Microbiol Biotechnol.

[52] Bellemain E, Carlsen T, Brochmann C, Coissac E, Taberlet P, Kauserud H (2010). ITS as an environmental DNA barcode for fungi: an in silico approach reveals potential PCR biases. BMC Microbiol.

[53] Spouge JL, Marino-Ramirez L (2012). The practical evaluation of DNA barcode efficacy. Methods Mol Biol.

[54] Seberg O (2004). The future of systematics: Assembling the Tree of Life. Systematist.

[55] Meier R, Shiyang K, Vaidya G, Ng PK (2006). DNA barcoding and taxonomy in Diptera: a tale of high intraspecific variability and low identification success. Syst Biol.

[56] Hibbett DS, Ohman A, Glotzer D, Nuhn M, Kirk P, Nilsson RH (2011). Progress in molecular and morphological taxon discovery in Fungi and options for formal classification of environmental sequences. Fungal Biol Rev.

[57] Blaxter M, Mann J, Chapman T, Thomas F, Whitton C, Floyd R, Abebe E (2005). Defining operational taxonomic units using DNA barcode data. Philos Trans R Soc Lond B Biol Sci.

[58] DeSalle R, Egan MG, Siddall M (2005). The unholy trinity: taxonomy, species delimitation and DNA barcoding. Philos Trans R Soc Lond B Biol Sci.

[59] Will KW, Rubinoff D (2004). Myth of the molecule: DNA barcodes for species cannot replace morphology for identification and classification. Cladistics.

[60] Erickson DL, Spouge JL, Resch A (2008). DNA barcoding in land plants: developing standards to quantify and maximize success. Taxon.

[61] Meyer CP, Paulay G (2005). DNA barcoding: Error rates based on comprehensive sampling. PLoS Biol.

[62] Bresinsky A (2008). Beiträge zu einer mykoflora deutschlands (2): Die gattungen hydropus bis hypsizygus mit angaben zur ökologie und verbreitung der arten. Regensburger mykologische schriften band 15.

[63] Babos M, Halász K, Zagyva T, Zöld-Balogh Á, Szegő D, Bratek Z (2011). Preliminary notes on dual relevance of ITS sequences and pigments in *Hygrocybe* taxonomy. Persoonia.

[64] Boertmann D (2010). Fungi of Northern Europe, Volume 1: The Genus Hygrocybe.

[65] Ramírez-López I, Villegas-Ríos M, Salas-Lizana R, Garibay-Orijel R, Alvarez-Manjarrez J (2015). *Thelephora versatilis* and *Thelephora pseudoversatilis*: two new cryptic species with polymorphic basidiomes inhabiting tropical deciduous and sub-perennial forests of the Mexican Pacific coast. Mycologia.

[66] Su M-S (2014). *Hypholoma cinnabarinum* revisited: A contribution to knowledge of *Agaricus* subgenus *Lanagaricus* section *Trisulphurati* (Agaricaceae, Agaricales). Mycoscience.

[67] Smith AH. 1957. A contribution toward a monograph of *Phaeocollybia*. Brittonia. 1957. 9:195–217

[68] Singer R (1986). The Agaricales in modern taxonomy.

[69] Norvell LL (1998). The biology and taxonomy of Pacific Northwest species of *Phaeocollybia* Heim (Agaricales, Cortinariaceae) [PhD dissertation].

[70] Norvell L (2000). *Phaeocollybia* in western North America. I. The Phaeocollybia kauffmanii complex. Can J Bot.

[71] Anderson IC, Chambers SM, Cairney JWG (1998). Molecular determination of genetic variation in Pisolithus isolates from a defined region in New South Wales, Australia. New Phytol.

[72] Martin F, Delaruelle C, Ivory M (1998). Genetic variability in intergenic spacers of ribosomal DNA in *Pisolithus* isolates associated with pine, eucalyptus and *Afzelia* in Lowland Kenyan forests. New Phytol.

[73] Díez J, Anta B, Manjón JL, Honrubia M (2001). Genetic variability of *Pisolithus* isolates associated with native hosts and exotic eucalyptus in the western Mediterranean region. New Phytol.

[74] Kope HH, Fortin JA (1990). Germination and comparative morphology of basidiospores of *Pisolithus arhizus*. Mycologia.

[75] Hebert PD, Penton EH, Burns JM, Janzen DH, Hallwachs W (2004). Ten species in one: DNA barcoding reveals cryptic species in the neotropical skipper butterfly *Astraptes* fulgerator. Proc Natl Acad Sci U S A.

[76] Grundt HH, Kjolner S, Borgen L, Rieseberg LH, Brochmann C (2006). High biological species diversity in the arctic flora. Proc Natl Acad Sci U S A.

[77] Taylor JW, Jacobson DJ, Kroken S, Kasuga T, Geiser DM, Hibbett DS, Fisher MC (2000). Phylogenetic species recognition and species concepts in fungi. Fungal Genet Biol.

[78] Stielow JB, Levesque CA, Seifert KA, Meyer W, Iriny L, Smits D, Renfurm R, Verkley GJ, Groenewald M, Chaduli D (2015). One fungus, which genes? Development and assessment of universal primers for potential secondary fungal DNA barcodes. Persoonia.

