# Supplementary material for: Effectiveness of ITS and sub-regions as DNA barcode markers for the identification of Basidiomycota (Fungi)
Source: BMC Microbiol. 2017 Feb 23;17:42. doi: 10.1186/s12866-017-0958-x (PMC5322588; doi:10.1186/s12866-017-0958-x)
Supplement: Additional file 2: — Number of species and sequences (specimens) recovered to each genus and their taxonomic affiliations. Data were compiled from our trimmed dataset. (DOCX 36 kb) [file 12866_2017_958_MOESM2_ESM.docx]

| **Genera** | **Family** | **Order** | **Class** | **Subphylum** | **Species*** | **Sequences**** |
| --- | --- | --- | --- | --- | --- | --- |
| *Agaricus* | Agaricaceae | Agaricales | Agaricomycetes | Agaricomycotina | 4 | 15 |
| *Albatrellus* | Albatrellaceae | Russulales | Agaricomycetes | Agaricomycotina | 1 | 7 |
| *Albomagister* | Tricholomataceae | Agaricales | Agaricomycetes | Agaricomycotina | 1 | 10 |
| *Alnicola* | Strophariaceae | Agaricales | Agaricomycetes | Agaricomycotina | 9 | 93 |
| *Alpova* | Paxillaceae | Boletales | Agaricomycetes | Agaricomycotina | 1 | 7 |
| *Amanita* | Amanitaceae | Agaricales | Agaricomycetes | Agaricomycotina | 37 | 338 |
| *Amphinema* | Atheliaceae | Atheliales | Agaricomycetes | Agaricomycotina | 1 | 16 |
| *Amyloporia* | Polyporaceae | Polyporales | Agaricomycetes | Agaricomycotina | 4 | 56 |
| *Amylostereum* | Amylostereaceae | Russulales | Agaricomycetes | Agaricomycotina | 1 | 4 |
| *Antherospora* | Floromycetaceae | Urocystidales | Ustilaginomycetes | Ustilaginomycotina | 2 | 14 |
| *Antrodia* | Fomitopsidaceae | Polyporales | Agaricomycetes | Agaricomycotina | 10 | 73 |
| *Antrodiella* | Phanerochaetaceae | Polyporales | Agaricomycetes | Agaricomycotina | 4 | 36 |
| *Armillaria* | Physalacriaceae | Agaricales | Agaricomycetes | Agaricomycotina | 5 | 45 |
| *Asterostroma* | Lachnocladiaceae | Russulales | Agaricomycetes | Agaricomycotina | 1 | 9 |
| *Astraeus* | Diplocystidiaceae | Boletales | Agaricomycetes | Agaricomycotina | 1 | 9 |
| *Auricularia* | Auriculariaceae | Auriculariales | Agaricomycetes | Agaricomycotina | 5 | 25 |
| *Auriscalpium* | Auriscalpiaceae | Russulales | Agaricomycetes | Agaricomycotina | 1 | 3 |
| *Bankera* | Bankeraceae | Thelephorales | Agaricomycetes | Agaricomycotina | 1 | 11 |
| *Battarrea* | Agaricaceae | Agaricales | Agaricomycetes | Agaricomycotina | 1 | 4 |
| *Bjerkandera* | Meruliaceae | Polyporales | Agaricomycetes | Agaricomycotina | 1 | 8 |
| *Boletopsis* | Bankeraceae | Thelephorales | Agaricomycetes | Agaricomycotina | 1 | 5 |
| *Boletus* | Boletaceae | Boletales | Agaricomycetes | Agaricomycotina | 19 | 178 |
| *Brevicellicium* | Hydnodontaceae | Trechisporales | Agaricomycetes | Agaricomycotina | 1 | 9 |
| *Butyriboletus* | Boletaceae | Boletales | Agaricomycetes | Agaricomycotina | 9 | 64 |
| *Byssocorticium* | Atheliaceae | Atheliales | Agaricomycetes | Agaricomycotina | 1 | 3 |
| *Caloboletus* | Boletaceae | Boletales | Agaricomycetes | Agaricomycotina | 1 | 3 |
| *Calocybella* | Lyophyllaceae | Agaricales | Agaricomycetes | Agaricomycotina | 1 | 4 |
| *Calvatia* | Agaricaceae | Agaricales | Agaricomycetes | Agaricomycotina | 2 | 12 |
| *Ceriporiopsis* | Phanerochaetaceae | Polyporales | Agaricomycetes | Agaricomycotina | 4 | 25 |
| *Cerrena* | Polyporaceae | Polyporales | Agaricomycetes | Agaricomycotina | 1 | 3 |
| *Chlorophyllum* | Agaricaceae | Agaricales | Agaricomycetes | Agaricomycotina | 4 | 24 |
| *Chromosera* | Hygrophoraceae | Agaricales | Agaricomycetes | Agaricomycotina | 1 | 3 |
| *Chroogomphus* | Gomphidiaceae | Boletales | Agaricomycetes | Agaricomycotina | 2 | 12 |
| *Cibaomyces* | Physalacriaceae | Agaricales | Agaricomycetes | Agaricomycotina | 1 | 3 |
| *Clavaria* | Clavariaceae | Agaricales | Agaricomycetes | Agaricomycotina | 4 | 13 |
| *Clavulina* | Clavulinaceae | Cantharellales | Agaricomycetes | Agaricomycotina | 3 | 11 |
| *Climacocystis* | Fomitopsidaceae | Polyporales | Agaricomycetes | Agaricomycotina | 1 | 6 |
| *Clitocybe* | Tricholomataceae | Agaricales | Agaricomycetes | Agaricomycotina | 1 | 6 |
| *Collybia* | Tricholomataceae | Agaricales | Agaricomycetes | Agaricomycotina | 2 | 9 |
| *Coniolepiota* | Agaricaceae | Agaricales | Agaricomycetes | Agaricomycotina | 1 | 3 |
| *Connopus* | Marasmiaceae | Agaricales | Agaricomycetes | Agaricomycotina | 1 | 18 |
| *Coprinellus* | Psathyrellaceae | Agaricales | Agaricomycetes | Agaricomycotina | 1 | 5 |
| *Coprinopsis* | Psathyrellaceae | Agaricales | Agaricomycetes | Agaricomycotina | 2 | 9 |
| *Coriolopsis* | Polyporaceae | Polyporales | Agaricomycetes | Agaricomycotina | 1 | 4 |
| *Cortinarius* | Cortinariaceae | Agaricales | Agaricomycetes | Agaricomycotina | 124 | 829 |
| *Craterellus* | Cantharellaceae | Cantharellales | Agaricomycetes | Agaricomycotina | 1 | 4 |
| *Crepidotus* | Inocybaceae | Agaricales | Agaricomycetes | Agaricomycotina | 2 | 10 |
| *Crustoderma* | Meruliaceae | Polyporales | Agaricomycetes | Agaricomycotina | 1 | 4 |
| *Cyphomyrmex* | Agaricaceae | Agaricales | Agaricomycetes | Agaricomycotina | 1 | 26 |
| *Cystoderma* | Agaricaceae | Agaricales | Agaricomycetes | Agaricomycotina | 6 | 27 |
| *Cystodermella* | Agaricaceae | Agaricales | Agaricomycetes | Agaricomycotina | 2 | 7 |
| *Cystolepiota* | Agaricaceae | Agaricales | Agaricomycetes | Agaricomycotina | 1 | 3 |
| *Dacryobolus* | Fomitopsidaceae | Polyporales | Agaricomycetes | Agaricomycotina | 1 | 4 |
| *Daedalea* | Fomitopsidaceae | Polyporales | Agaricomycetes | Agaricomycotina | 1 | 5 |
| *Daedaleopsis* | Polyporaceae | Polyporales | Agaricomycetes | Agaricomycotina | 1 | 7 |
| *Datronia* | Polyporaceae | Polyporales | Agaricomycetes | Agaricomycotina | 2 | 21 |
| *Dentocorticium* | Polyporaceae | Polyporales | Agaricomycetes | Agaricomycotina | 1 | 6 |
| *Descolea* | Cortinariaceae | Agaricales | Agaricomycetes | Agaricomycotina | 1 | 4 |
| *Earliella* | Polyporaceae | Polyporales | Agaricomycetes | Agaricomycotina | 1 | 6 |
| *Endoraecium* | Raveneliaceae | Pucciniales | Pucciniomycetes | Pucciniomycotina | 4 | 23 |
| *Entoloma* | Entolomataceae | Agaricales | Agaricomycetes | Agaricomycotina | 14 | 101 |
| *Entyloma* | Entylomataceae | Entylomatales | Exobasidiomycetes | Ustilaginomycotina | 2 | 18 |
| *Exobasidium* | Exobasidiaceae | Exobasidiales | Exobasidiomycetes | Ustilaginomycotina | 3 | 18 |
| *Favolus* | Polyporaceae | Polyporales | Agaricomycetes | Agaricomycotina | 2 | 8 |
| *Fibroporia* | Fomitopsidaceae | Polyporales | Agaricomycetes | Agaricomycotina | 3 | 17 |
| *Flammulina* | Physalacriaceae | Agaricales | Agaricomycetes | Agaricomycotina | 4 | 40 |
| *Flavodon* | Meruliaceae | Polyporales | Agaricomycetes | Agaricomycotina | 1 | 3 |
| *Fomes* | Polyporaceae | Polyporales | Agaricomycetes | Agaricomycotina | 1 | 5 |
| *Fomitopsis* | Fomitopsidaceae | Polyporales | Agaricomycetes | Agaricomycotina | 4 | 41 |
| *Fuscoporia* | Hymenochaetaceae | Hymenochaetales | Agaricomycetes | Agaricomycotina | 2 | 8 |
| *Galerina* | Strophariaceae | Agaricales | Agaricomycetes | Agaricomycotina | 1 | 3 |
| *Ganoderma* | Ganodermataceae | Polyporales | Agaricomycetes | Agaricomycotina | 7 | 56 |
| *Geastrum* | Geastraceae | Geastrales | Agaricomycetes | Agaricomycotina | 3 | 23 |
| *Gelatoporia* | Meruliaceae | Polyporales | Agaricomycetes | Agaricomycotina | 1 | 3 |
| *Gloeophyllum* | Gloeophyllaceae | Gloeophyllales | Agaricomycetes | Agaricomycotina | 2 | 14 |
| *Gloeoporus* | Meruliaceae | Polyporales | Agaricomycetes | Agaricomycotina | 1 | 122 |
| *Gymnopilus* | Strophariaceae | Agaricales | Agaricomycetes | Agaricomycotina | 2 | 14 |
| *Gymnopus* | Marasmiaceae | Agaricales | Agaricomycetes | Agaricomycotina | 15 | 134 |
| *Gyroporus* | Gyroporaceae | Boletales | Agaricomycetes | Agaricomycotina | 1 | 4 |
| *Hebeloma* | Strophariaceae | Agaricales | Agaricomycetes | Agaricomycotina | 19 | 198 |
| *Helicobasidium* | Helicobasidiaceae | Helicobasidiales | Pucciniomycetes | Pucciniomycotina | 4 | 26 |
| *Heliogaster* | Boletaceae | Boletales | Agaricomycetes | Agaricomycotina | 1 | 8 |
| *Helvellosebacina* | Sebacinaceae | Sebacinales | Agaricomycetes | Agaricomycotina | 1 | 7 |
| *Hohenbuehelia* | Pleurotaceae | Agaricales | Agaricomycetes | Agaricomycotina | 2 | 9 |
| *Hydnellum* | Bankeraceae | Thelephorales | Agaricomycetes | Agaricomycotina | 9 | 69 |
| *Hydnum* | Hydnaceae | Cantharellales | Agaricomycetes | Agaricomycotina | 6 | 54 |
| *Hygrocybe* | Hygrophoraceae | Agaricales | Agaricomycetes | Agaricomycotina | 4 | 39 |
| *Hygrophorus* | Hygrophoraceae | Agaricales | Agaricomycetes | Agaricomycotina | 3 | 14 |
| *Hymenogaster* | Strophariaceae | Agaricales | Agaricomycetes | Agaricomycotina | 1 | 5 |
| *Hymenopellis* | Physalacriaceae | Agaricales | Agaricomycetes | Agaricomycotina | 4 | 30 |
| *Hyphoderma* | Meruliaceae | Polyporales | Agaricomycetes | Agaricomycotina | 5 | 68 |
| *Hyphodermella* | Phanerochaetaceae | Polyporales | Agaricomycetes | Agaricomycotina | 2 | 19 |
| *Hypholoma* | Strophariaceae | Agaricales | Agaricomycetes | Agaricomycotina | 2 | 9 |
| *Hypochnicium* | Meruliaceae | Polyporales | Agaricomycetes | Agaricomycotina | 1 | 3 |
| *Inocybe* | Inocybaceae | Agaricales | Agaricomycetes | Agaricomycotina | 53 | 444 |
| *Laccaria* | Hydnangiaceae | Agaricales | Agaricomycetes | Agaricomycotina | 8 | 98 |
| *Lactarius* | Russulaceae | Russulales | Agaricomycetes | Agaricomycotina | 46 | 362 |
| *Lactifluus* | Russulaceae | Russulales | Agaricomycetes | Agaricomycotina | 2 | 24 |
| *Laetiporus* | Fomitopsidaceae | Polyporales | Agaricomycetes | Agaricomycotina | 1 | 3 |
| *Leiotrametes* | Polyporaceae | Polyporales | Agaricomycetes | Agaricomycotina | 1 | 10 |
| *Lentinellus* | Auriscalpiaceae | Russulales | Agaricomycetes | Agaricomycotina | 7 | 75 |
| *Lentinula* | Marasmiaceae | Agaricales | Agaricomycetes | Agaricomycotina | 1 | 3 |
| *Lentinus* | Polyporaceae | Polyporales | Agaricomycetes | Agaricomycotina | 7 | 44 |
| *Lepiota* | Agaricaceae | Agaricales | Agaricomycetes | Agaricomycotina | 12 | 104 |
| *Lepista* | Tricholomataceae | Agaricales | Agaricomycetes | Agaricomycotina | 2 | 7 |
| *Leucoagaricus* | Agaricaceae | Agaricales | Agaricomycetes | Agaricomycotina | 10 | 62 |
| *Leucocoprinus* | Agaricaceae | Agaricales | Agaricomycetes | Agaricomycotina | 1 | 4 |
| *Leucopaxillus* | Tricholomataceae | Agaricales | Agaricomycetes | Agaricomycotina | 3 | 10 |
| *Limacella* | Amanitaceae | Agaricales | Agaricomycetes | Agaricomycotina | 1 | 7 |
| *Lycoperdon* | Agaricaceae | Agaricales | Agaricomycetes | Agaricomycotina | 2 | 26 |
| *Lyomyces* | Corticiaceae | Corticiales | Agaricomycetes | Agaricomycotina | 2 | 40 |
| *Lyophyllum* | Lyophyllaceae | Agaricales | Agaricomycetes | Agaricomycotina | 3 | 20 |
| *Lysurus* | Phallaceae | Phallales | Agaricomycetes | Agaricomycotina | 1 | 6 |
| *Macrolepiota* | Agaricaceae | Agaricales | Agaricomycetes | Agaricomycotina | 6 | 30 |
| *Marasmius* | Marasmiaceae | Agaricales | Agaricomycetes | Agaricomycotina | 1 | 3 |
| *Megacollybia* | Marasmiaceae | Agaricales | Agaricomycetes | Agaricomycotina | 6 | 121 |
| *Melampsora* | Melampsoraceae | Pucciniales | Pucciniomycetes | Pucciniomycotina | 5 | 52 |
| *Melampsoridium* | Pucciniastraceae | Pucciniales | Pucciniomycetes | Pucciniomycotina | 1 | 7 |
| *Melanoleuca* | Tricholomataceae | Agaricales | Agaricomycetes | Agaricomycotina | 8 | 55 |
| *Melanophyllum* | Agaricaceae | Agaricales | Agaricomycetes | Agaricomycotina | 1 | 5 |
| *Microbotryum* | Microbotryaceae | Microbotryales | Microbotryomycetes | Pucciniomycotina | 10 | 74 |
| *Mucidula* | Physalacriaceae | Agaricales | Agaricomycetes | Agaricomycotina | 2 | 11 |
| *Mycena* | Mycenaceae | Agaricales | Agaricomycetes | Agaricomycotina | 9 | 88 |
| *Mycetinis* | Marasmiaceae | Agaricales | Agaricomycetes | Agaricomycotina | 1 | 5 |
| *Naucoria* | Strophariaceae | Agaricales | Agaricomycetes | Agaricomycotina | 1 | 10 |
| *Neofavolus* | Polyporaceae | Polyporales | Agaricomycetes | Agaricomycotina | 2 | 10 |
| *Octaviania* | Boletaceae | Boletales | Agaricomycetes | Agaricomycotina | 2 | 9 |
| *Oligoporus* | Polyporaceae | Polyporales | Agaricomycetes | Agaricomycotina | 2 | 6 |
| *Omphalina* | Tricholomataceae | Agaricales | Agaricomycetes | Agaricomycotina | 1 | 3 |
| *Omphalotus* | Marasmiaceae | Agaricales | Agaricomycetes | Agaricomycotina | 1 | 6 |
| *Oudemansiella* | Physalacriaceae | Agaricales | Agaricomycetes | Agaricomycotina | 1 | 4 |
| *Oxyporus* | Polyporaceae | Polyporales | Agaricomycetes | Agaricomycotina | 1 | 5 |
| *Pachylepyrium* | Strophariaceae | Agaricales | Agaricomycetes | Agaricomycotina | 1 | 4 |
| *Panaeolus* | Bolbitiaceae | Agaricales | Agaricomycetes | Agaricomycotina | 1 | 3 |
| *Panellus* | Mycenaceae | Agaricales | Agaricomycetes | Agaricomycotina | 1 | 26 |
| *Parasola* | Psathyrellaceae | Agaricales | Agaricomycetes | Agaricomycotina | 3 | 16 |
| *Paxillus* | Paxillaceae | Boletales | Agaricomycetes | Agaricomycotina | 3 | 41 |
| *Peniophorella* | Corticiaceae | Corticiales | Agaricomycetes | Agaricomycotina | 2 | 58 |
| *Perenniporia* | Polyporaceae | Polyporales | Agaricomycetes | Agaricomycotina | 1 | 3 |
| *Phaeocollybia* | Cortinariaceae | Agaricales | Agaricomycetes | Agaricomycotina | 3 | 21 |
| *Phaeolus* | Fomitopsidaceae | Polyporales | Agaricomycetes | Agaricomycotina | 1 | 3 |
| *Phakopsora* | Phakopsoraceae | Pucciniales | Pucciniomycetes | Pucciniomycotina | 1 | 3 |
| *Phanerochaete* | Phanerochaetaceae | Polyporales | Agaricomycetes | Agaricomycotina | 3 | 15 |
| *Phellinus* | Hymenochaetaceae | Hymenochaetales | Agaricomycetes | Agaricomycotina | 3 | 57 |
| *Phellodon* | Bankeraceae | Thelephorales | Agaricomycetes | Agaricomycotina | 7 | 41 |
| *Pholiota* | Strophariaceae | Agaricales | Agaricomycetes | Agaricomycotina | 1 | 5 |
| *Piloderma* | Atheliaceae | Atheliales | Agaricomycetes | Agaricomycotina | 2 | 7 |
| *Piptoporus* | Fomitopsidaceae | Polyporales | Agaricomycetes | Agaricomycotina | 1 | 7 |
| *Pisolithus* | Sclerodermataceae | Boletales | Agaricomycetes | Agaricomycotina | 2 | 6 |
| *Pleurotus* | Pleurotaceae | Agaricales | Agaricomycetes | Agaricomycotina | 7 | 75 |
| *Plicaturopsis* | Amylocorticiaceae | Agaricales | Agaricomycetes | Agaricomycotina | 1 | 4 |
| *Pluteus* | Pluteaceae | Agaricales | Agaricomycetes | Agaricomycotina | 26 | 198 |
| *Pogonoloma* | Tricholomataceae | Agaricales | Agaricomycetes | Agaricomycotina | 1 | 3 |
| *Polyporus* | Polyporaceae | Polyporales | Agaricomycetes | Agaricomycotina | 7 | 34 |
| *Ponticulomyces* | Physalacriaceae | Agaricales | Agaricomycetes | Agaricomycotina | 1 | 3 |
| *Porodaedalea* | Hymenochaetaceae | Hymenochaetales | Agaricomycetes | Agaricomycotina | 2 | 9 |
| *Postia* | Fomitopsidaceae | Polyporales | Agaricomycetes | Agaricomycotina | 6 | 40 |
| *Psathyrella* | Psathyrellaceae | Agaricales | Agaricomycetes | Agaricomycotina | 2 | 7 |
| *Pseudoclitocybe* | Tricholomataceae | Agaricales | Agaricomycetes | Agaricomycotina | 1 | 3 |
| *Pseudomerulius* | Tapinellaceae | Boletales | Agaricomycetes | Agaricomycotina | 1 | 3 |
| *Pseudotomentella* | Thelephoraceae | Thelephorales | Agaricomycetes | Agaricomycotina | 1 | 3 |
| *Pseudotricholoma* | Tricholomataceae | Agaricales | Agaricomycetes | Agaricomycotina | 1 | 3 |
| *Psilocybe* | Strophariaceae | Agaricales | Agaricomycetes | Agaricomycotina | 2 | 11 |
| *Puccinia* | Pucciniaceae | Pucciniales | Pucciniomycetes | Pucciniomycotina | 7 | 192 |
| *Pycnoporellus* | Fomitopsidaceae | Polyporales | Agaricomycetes | Agaricomycotina | 2 | 8 |
| *Pyrofomes* | Polyporaceae | Polyporales | Agaricomycetes | Agaricomycotina | 1 | 11 |
| *Ramaria* | Gomphaceae | Gomphales | Agaricomycetes | Agaricomycotina | 6 | 36 |
| *Resinicium* | Rickenellaceae | Hymenochaetales | Agaricomycetes | Agaricomycotina | 2 | 12 |
| *Rhizopogon* | Rhizopogonaceae | Boletales | Agaricomycetes | Agaricomycotina | 11 | 76 |
| *Rhodocollybia* | Marasmiaceae | Agaricales | Agaricomycetes | Agaricomycotina | 3 | 29 |
| *Rhodotorula* | Sporidiobolaceae | Sporidiobolales | Microbotryomycetes | Pucciniomycotina | 1 | 5 |
| *Rhodotus* | Physalacriaceae | Agaricales | Agaricomycetes | Agaricomycotina | 1 | 5 |
| *Rigidoporus* | Meripilaceae | Polyporales | Agaricomycetes | Agaricomycotina | 2 | 26 |
| *Royoporus* | Polyporaceae | Polyporales | Agaricomycetes | Agaricomycotina | 1 | 4 |
| *Russula* | Russulaceae | Russulales | Agaricomycetes | Agaricomycotina | 45 | 401 |
| *Sarcodon* | Bankeraceae | Thelephorales | Agaricomycetes | Agaricomycotina | 7 | 53 |
| *Sarcoporia* | Polyporaceae | Polyporales | Agaricomycetes | Agaricomycotina | 1 | 12 |
| *Schizophyllum* | Schizophyllaceae | Agaricales | Agaricomycetes | Agaricomycotina | 1 | 6 |
| *Scleroderma* | Sclerodermataceae | Boletales | Agaricomycetes | Agaricomycotina | 3 | 18 |
| *Sebacina* | Sebacinaceae | Sebacinales | Agaricomycetes | Agaricomycotina | 3 | 113 |
| *Sistotrema* | Hydnaceae | Cantharellales | Agaricomycetes | Agaricomycotina | 1 | 8 |
| *Sparassis* | Sparassidaceae | Polyporales | Agaricomycetes | Agaricomycotina | 1 | 5 |
| *Spongiporus* | Fomitopsidaceae | Polyporales | Agaricomycetes | Agaricomycotina | 1 | 6 |
| *Stephanospora* | Stephanosporaceae | Boletales | Agaricomycetes | Agaricomycotina | 5 | 33 |
| *Strobilomyces* | Boletaceae | Boletales | Agaricomycetes | Agaricomycotina | 1 | 28 |
| *Strobilurus* | Physalacriaceae | Agaricales | Agaricomycetes | Agaricomycotina | 3 | 10 |
| *Suillus* | Suillaceae | Boletales | Agaricomycetes | Agaricomycotina | 6 | 33 |
| *Tapinella* | Tapinellaceae | Boletales | Agaricomycetes | Agaricomycotina | 1 | 3 |
| *Thaxterogaster* | Cortinariaceae | Agaricales | Agaricomycetes | Agaricomycotina | 1 | 4 |
| *Thecaphora* | Glomosporiaceae | Urocystidales | Ustilaginomycetes | Ustilaginomycotina | 2 | 18 |
| *Thelephora* | Thelephoraceae | Thelephorales | Agaricomycetes | Agaricomycotina | 2 | 20 |
| *Tilletia* | Tilletiaceae | Tilletiales | Exobasidiomycetes | Ustilaginomycotina | 3 | 20 |
| *Tomentella* | Thelephoraceae | Thelephorales | Agaricomycetes | Agaricomycotina | 4 | 32 |
| *Tomentellopsis* | Thelephoraceae | Thelephorales | Agaricomycetes | Agaricomycotina | 1 | 3 |
| *Tomophagus* | Ganodermataceae | Polyporales | Agaricomycetes | Agaricomycotina | 1 | 3 |
| *Trametes* | Polyporaceae | Polyporales | Agaricomycetes | Agaricomycotina | 12 | 126 |
| *Trametopsis* | Polyporaceae | Polyporales | Agaricomycetes | Agaricomycotina | 1 | 4 |
| *Tranzscheliella* | Ustilaginaceae | Ustilaginales | Ustilaginomycetes | Ustilaginomycotina | 1 | 4 |
| *Tricholoma* | Tricholomataceae | Agaricales | Agaricomycetes | Agaricomycotina | 14 | 179 |
| *Tricholomopsis* | Tricholomataceae | Agaricales | Agaricomycetes | Agaricomycotina | 3 | 16 |
| *Tuberculina* | Helicobasidiaceae | Helicobasidiales | Pucciniomycetes | Pucciniomycotina | 3 | 42 |
| *Tylopilus* | Boletaceae | Boletales | Agaricomycetes | Agaricomycotina | 1 | 7 |
| *Typhula* | Typhulaceae | Agaricales | Agaricomycetes | Agaricomycotina | 1 | 3 |
| *Uromyces* | Pucciniaceae | Pucciniales | Pucciniomycetes | Pucciniomycotina | 1 | 4 |
| *Uromycladium* | Pileolariaceae | Pucciniales | Pucciniomycetes | Pucciniomycotina | 1 | 14 |
| *Volvopluteus* | Pluteaceae | Agaricales | Agaricomycetes | Agaricomycotina | 1 | 5 |
| *Vuilleminia* | Corticiaceae | Corticiales | Agaricomycetes | Agaricomycotina | 3 | 24 |
| *Xerocomellus* | Boletaceae | Boletales | Agaricomycetes | Agaricomycotina | 1 | 3 |
| *Xerocomus* | Boletaceae | Boletales | Agaricomycetes | Agaricomycotina | 2 | 42 |
| *Xeromphalina* | Mycenaceae | Agaricales | Agaricomycetes | Agaricomycotina | 2 | 65 |
| *Xerula* | Physalacriaceae | Agaricales | Agaricomycetes | Agaricomycotina | 1 | 4 |

* the total number of species recovered for each genera

** the total number of sequences (specimens) recovered for each genera
