# Supplementary material for: Effectiveness of ITS and sub-regions as DNA barcode markers for the identification of Basidiomycota (Fungi)
Source: BMC Microbiol. 2017 Feb 23;17:42. doi: 10.1186/s12866-017-0958-x (PMC5322588; doi:10.1186/s12866-017-0958-x)
Supplement: Additional file 3: — List of genera with sequences originated from type specimens and their PCI values (ITS, ITS1, ITS 2) and groups according to the barcode gap analysis. (DOCX 61 kb) [file 12866_2017_958_MOESM3_ESM.docx]

Additional File 3 List of genera with sequences originated from type specimens and their PCI values (ITS, ITS1, ITS 2) and groups according to the barcode gap analysis.

| Genera with type specimens | PCI values for ITS (ITS1+5.8S+ ITS2) | PCI values for ITS1 | PCI values for ITS2 | Group from barcode gap analyses  (see Table 2) |
| --- | --- | --- | --- | --- |
| *Amanita* | 24 | 27 | 27 | 2 |
| *Antherospora* | 100 | 100 | 100 | 1 |
| *Auricularia* | 80 | 40 | 60 | 2 |
| *Boletus* | 32 | 26 | 47 | 3 |
| *Butyriboletus* | 67 | 33 | 67 | 4 |
| *Cortinarius* | 36 | 45 | 37 | 3 |
| *Cystoderma* | 50 | 50 | 33 | 3 |
| *Endoraecium* | 100 | 100 | 100 | 1 |
| *Entoloma* | 100 | 86 | 93 | 1 |
| *Entyloma* | 100 | 100 | 100 | 1 |
| *Hebeloma* | 42 | 37 | 32 | 3 |
| *Hydnum* | 50 | 50 | 50 | 4 |
| *Hyphoderma* | 60 | 60 | 80 | 3 |
| *Inocybe* | 30 | 19 | 28 | 3 |
| *Lentinellus* | 43 | 43 | 29 | 2 |
| *Leucoagaricus* | 90 | 100 | 80 | 2 |
| *Megacollybia* | 83 | 83 | 33 | 2 |
| *Melanoleuca* | 38 | 38 | 25 | 3 |
| *Microbotryum* | 70 | 80 | 50 | 2 |
| *Octaviania* | 100 | 100 | 100 | 1 |
| *Peniophorella* | 50 | 50 | 50 | 4 |
| *Pluteus* | 27 | 27 | 23 | 3 |
| *Porodaedalea* | 100 | 100 | 100 | 1 |
| *Psilocybe* | 100 | 100 | 100 | 1 |
| *Stephanospora* | 80 | 80 | 20 | 2 |
| *Suillus* | 83 | 83 | 83 | 1 |
| *Xeromphalina* | 100 | 100 | 50 | 1 |
