# Supplementary material for: Effectiveness of ITS and sub-regions as DNA barcode markers for the identification of Basidiomycota (Fungi)
Source: BMC Microbiol. 2017 Feb 23;17:42. doi: 10.1186/s12866-017-0958-x (PMC5322588; doi:10.1186/s12866-017-0958-x)
Supplement: Additional file 4: — List of sequences with RefSeq accessions interchangeably with GenBank numbers. (DOCX 110 kb) [file 12866_2017_958_MOESM4_ESM.docx]

Additional File 4 List of sequences with RefSeq accessions interchangeably with GenBank numbers.

| NCBI RefSeq Id | NCBI Nucleotide ID | | Complete Taxonomic Identification |
| --- | --- | --- | --- |
| NR_130307 | KF732586 | *Cortinarius balteatialutaceus*;*Cortinarius*;Cortinariaceae;Agaricales;Agaricomycetes;Agaricomycotina | |
| NR_130224 | KF732321 | *Cortinarius herpeticus*;*Cortinarius*;Cortinariaceae;Agaricales;Agaricomycetes;Agaricomycotina | |
| NR_130260 | KF732420 | *Cortinarius sannio*;*Cortinarius*;Cortinariaceae;Agaricales;Agaricomycetes;Agaricomycotina | |
| NR_130252 | KF732406 | *Cortinarius purpurascens*;*Cortinarius*;Cortinariaceae;Agaricales;Agaricomycetes;Agaricomycotina | |
| NR_131885 | KP165563 | *Cortinarius privignipallens*;*Cortinarius*;Cortinariaceae;Agaricales;Agaricomycetes;Agaricomycotina | |
| NR_130213 | KF732294 | *Cortinarius cupreorufus*;*Cortinarius*;Cortinariaceae;Agaricales;Agaricomycetes;Agaricomycotina | |
| NR_130279 | KF732466 | *Cortinarius variicolor*;*Cortinarius*;Cortinariaceae;Agaricales;Agaricomycetes;Agaricomycotina | |
| NR_130212 | KF732293 | *Cortinarius cumatilis*;*Cortinarius*;Cortinariaceae;Agaricales;Agaricomycetes;Agaricomycotina | |
| NR_130242 | KF732380 | *Cortinarius percomis*;*Cortinarius*;Cortinariaceae;Agaricales;Agaricomycetes;Agaricomycotina | |
| NR_130225 | KF732325 | *Cortinarius infractus*;*Cortinarius*;Cortinariaceae;Agaricales;Agaricomycetes;Agaricomycotina | |
| NR_130246 | KF732387 | *Cortinarius porphyropus*;*Cortinarius*;Cortinariaceae;Agaricales;Agaricomycetes;Agaricomycotina | |
| NR_130262 | KF732423 | *Cortinarius scaurus*;*Cortinarius*;Cortinariaceae;Agaricales;Agaricomycetes;Agaricomycotina | |
| NR_132080 | KJ862361 | *Endoraecium peggii*;*Endoraecium*;Raveneliaceae;Pucciniales;Pucciniomycetes;Pucciniomycotina | |
| NR_119790 | GU233337 | *Cortinarius saturniorum*;*Cortinarius*;Cortinariaceae;Agaricales;Agaricomycetes;Agaricomycotina | |
| NR_130306 | KF732583 | *Cortinarius talimultiformis*;*Cortinarius*;Cortinariaceae;Agaricales;Agaricomycetes;Agaricomycotina | |
| NR_130222 | KF732315 | *Cortinarius glaucopus*;*Cortinarius*;Cortinariaceae;Agaricales;Agaricomycetes;Agaricomycotina | |
| NR_130232 | KF732350 | *Cortinarius multiformis*;*Cortinarius*;Cortinariaceae;Agaricales;Agaricomycetes;Agaricomycotina | |
| NR_130211 | KF732291 | *Cortinarius crassus*;*Cortinarius*;Cortinariaceae;Agaricales;Agaricomycetes;Agaricomycotina | |
| NR_130247 | KF732389 | *Cortinarius praestans*;*Cortinarius*;Cortinariaceae;Agaricales;Agaricomycetes;Agaricomycotina | |
| NR_130230 | KF732348 | *Cortinarius misermontii*;*Cortinarius*;Cortinariaceae;Agaricales;Agaricomycetes;Agaricomycotina | |
| NR_131816 | JN114080 | *Cortinarius cinnamomeus*, *Cortinarius*;Cortinariaceae;Agaricales;Agaricomycetes;Agaricomycotina | |
| NR_131846 | KP013203 | *Cortinarius orasericeus*;*Cortinarius*;Cortinariaceae;Agaricales;Agaricomycetes;Agaricomycotina | |
| NR_130199 | KF732271 | *Cortinarius variicolor*;*Cortinarius*;Cortinariaceae;Agaricales;Agaricomycetes;Agaricomycotina | |
| NR_130281 | KF732469 | *Cortinarius varius*;*Cortinarius*;Cortinariaceae;Agaricales;Agaricomycetes;Agaricomycotina | |
| NR_130273 | KF732454 | *Cortinarius subtortus*;*Cortinarius*;Cortinariaceae;Agaricales;Agaricomycetes;Agaricomycotina | |
