# Supplementary figures and images for: Effectiveness of ITS and sub-regions as DNA barcode markers for the identification of Basidiomycota (Fungi)
Source: BMC Microbiol. 2017 Feb 23;17:42. doi: 10.1186/s12866-017-0958-x (PMC5322588; doi:10.1186/s12866-017-0958-x)

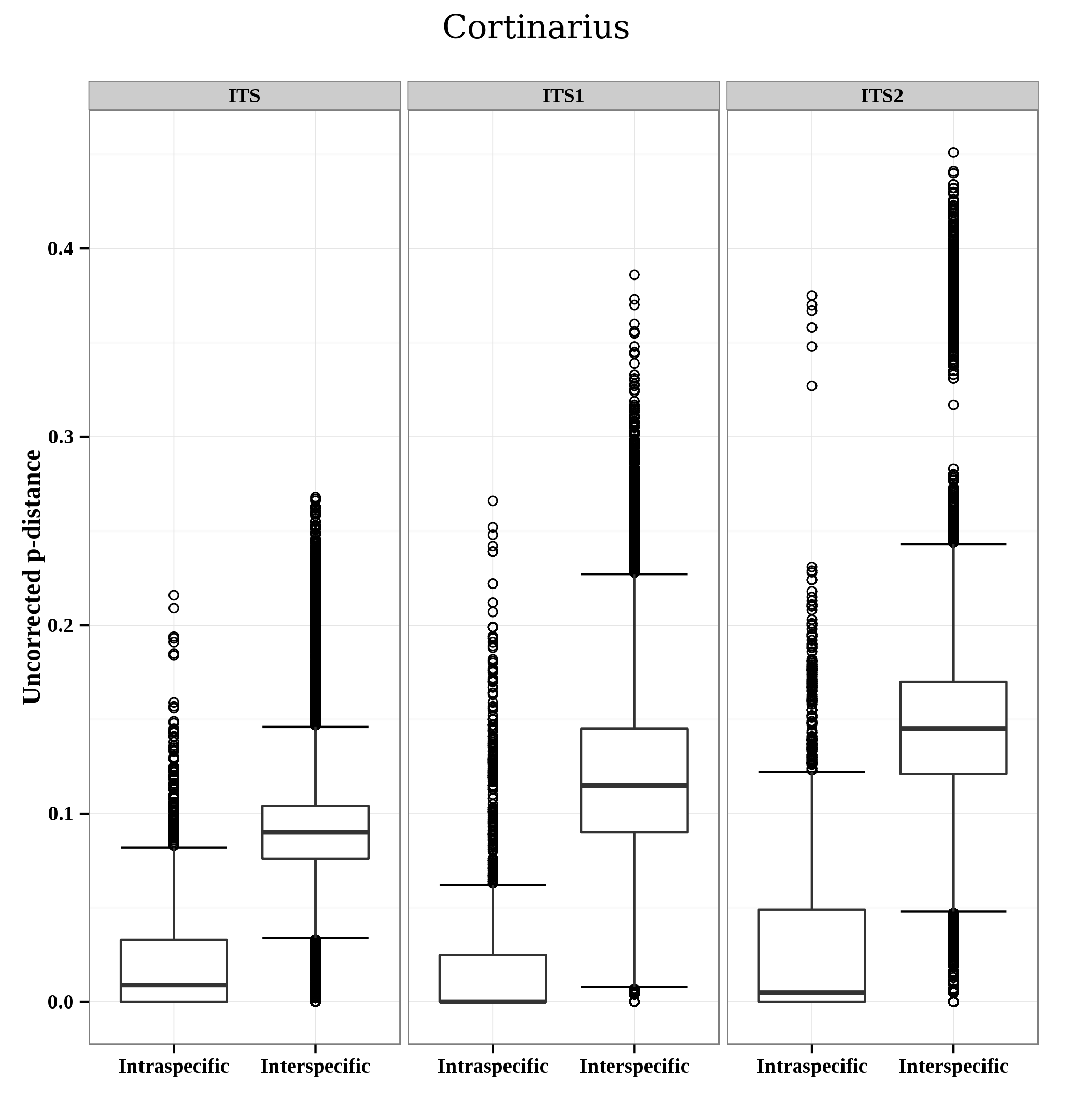

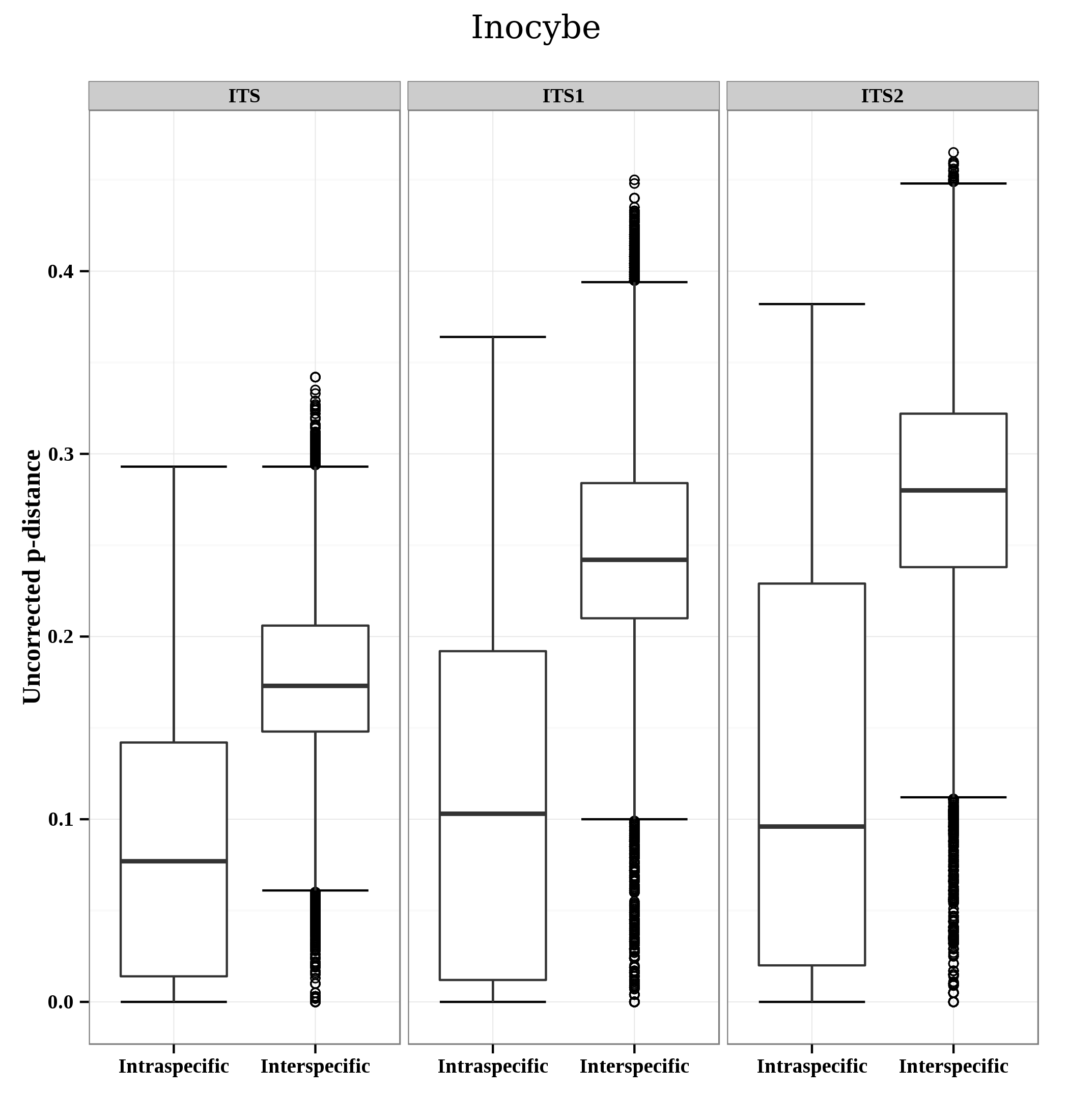

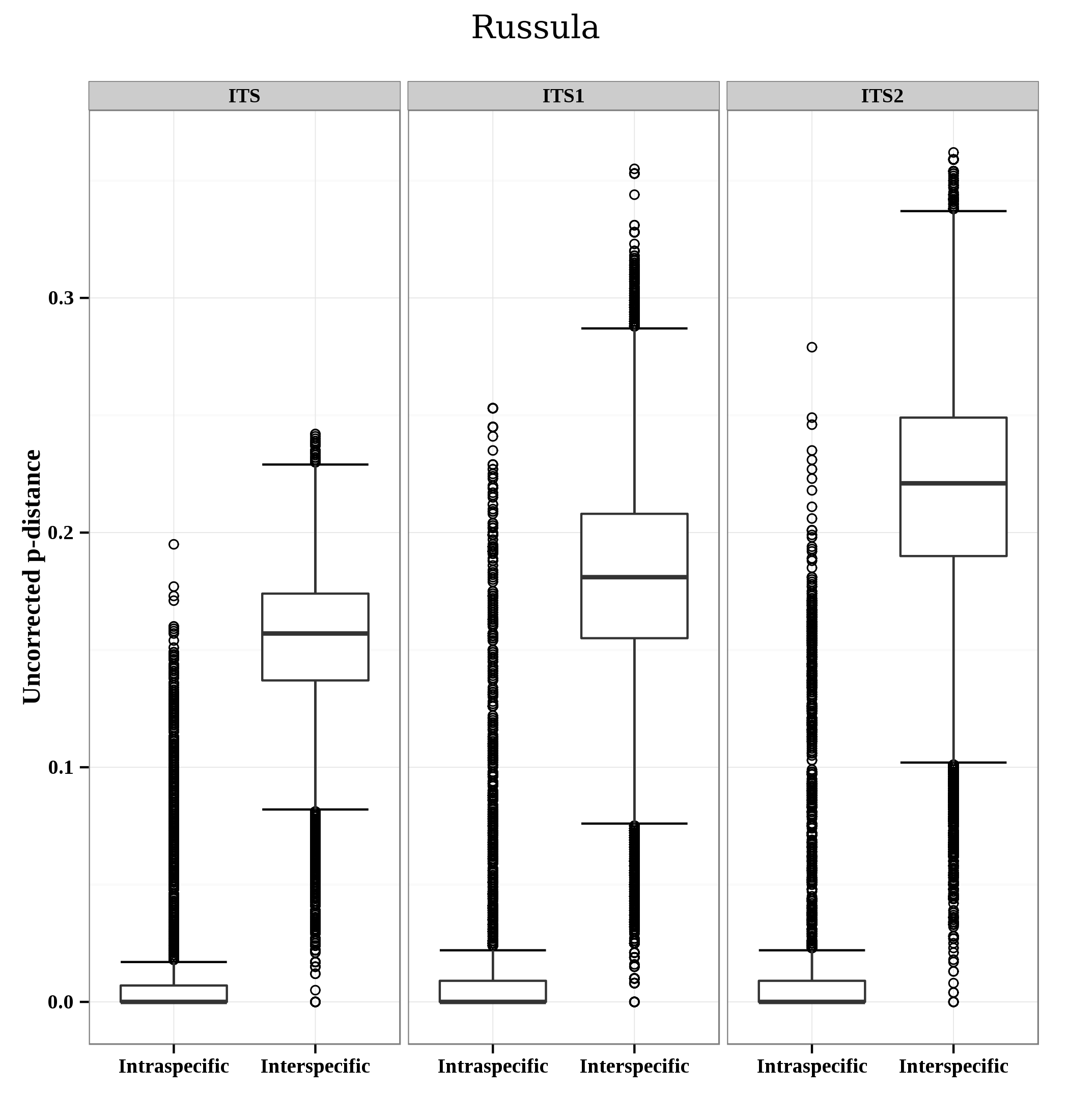

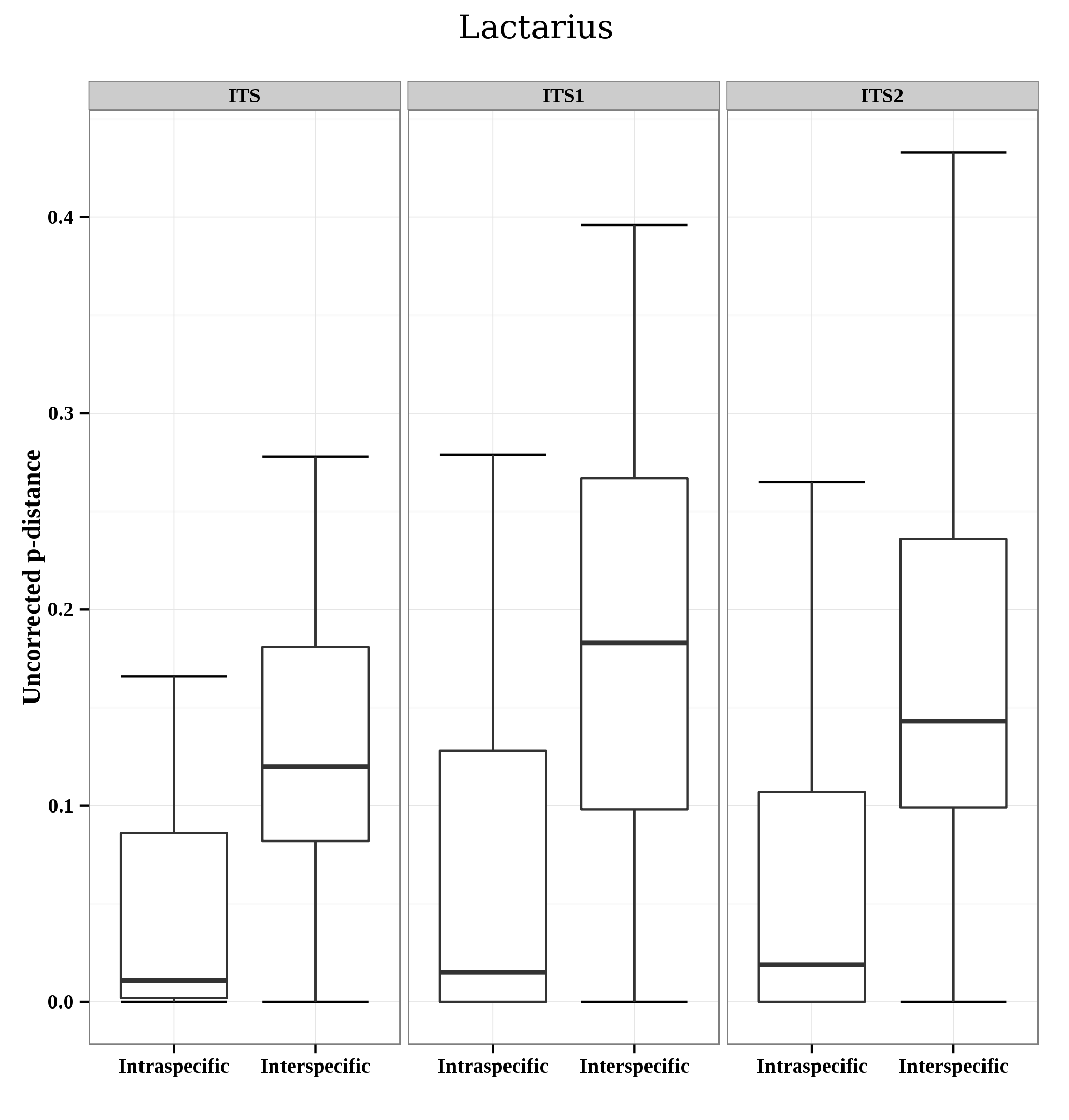

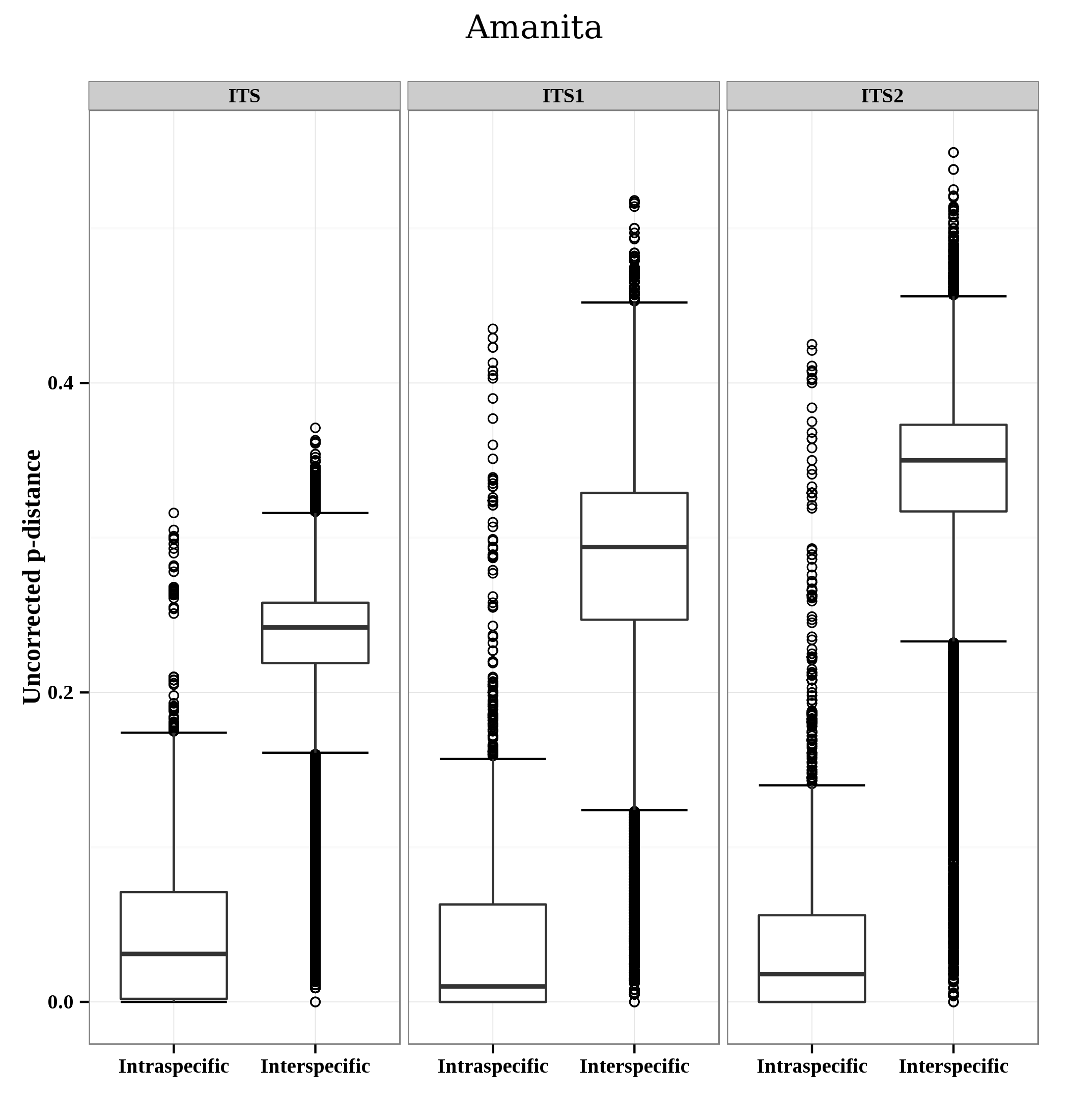

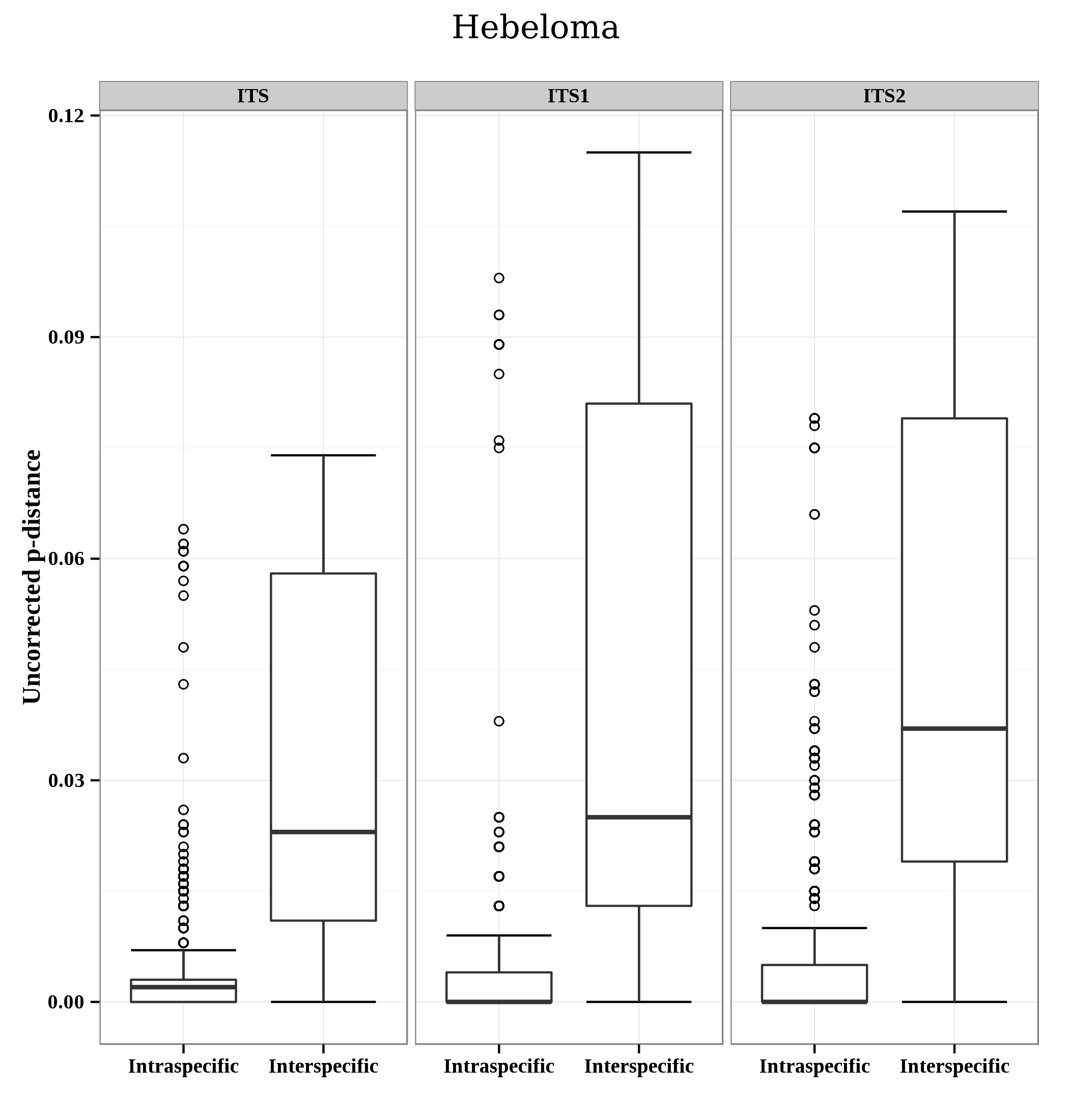

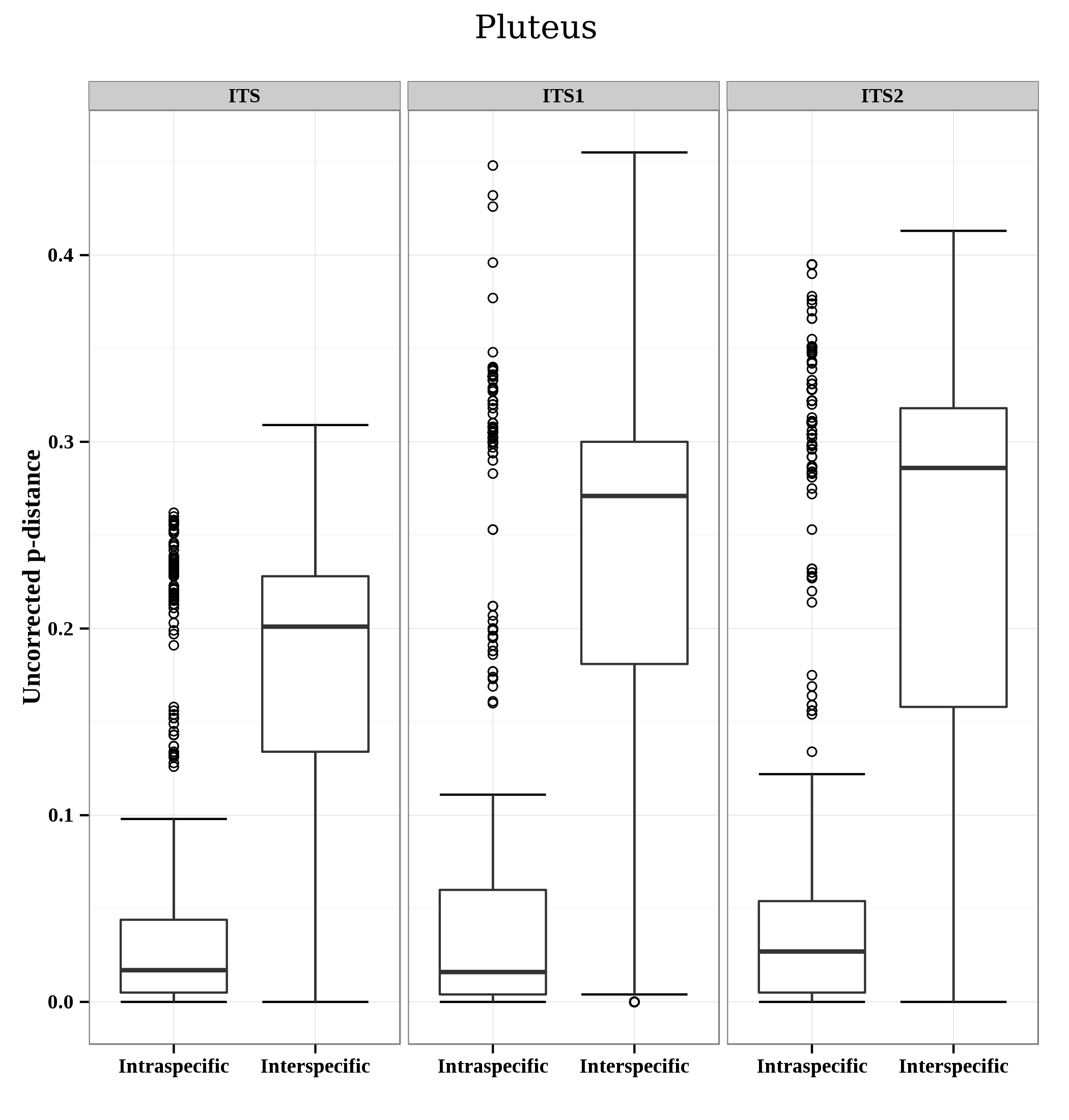

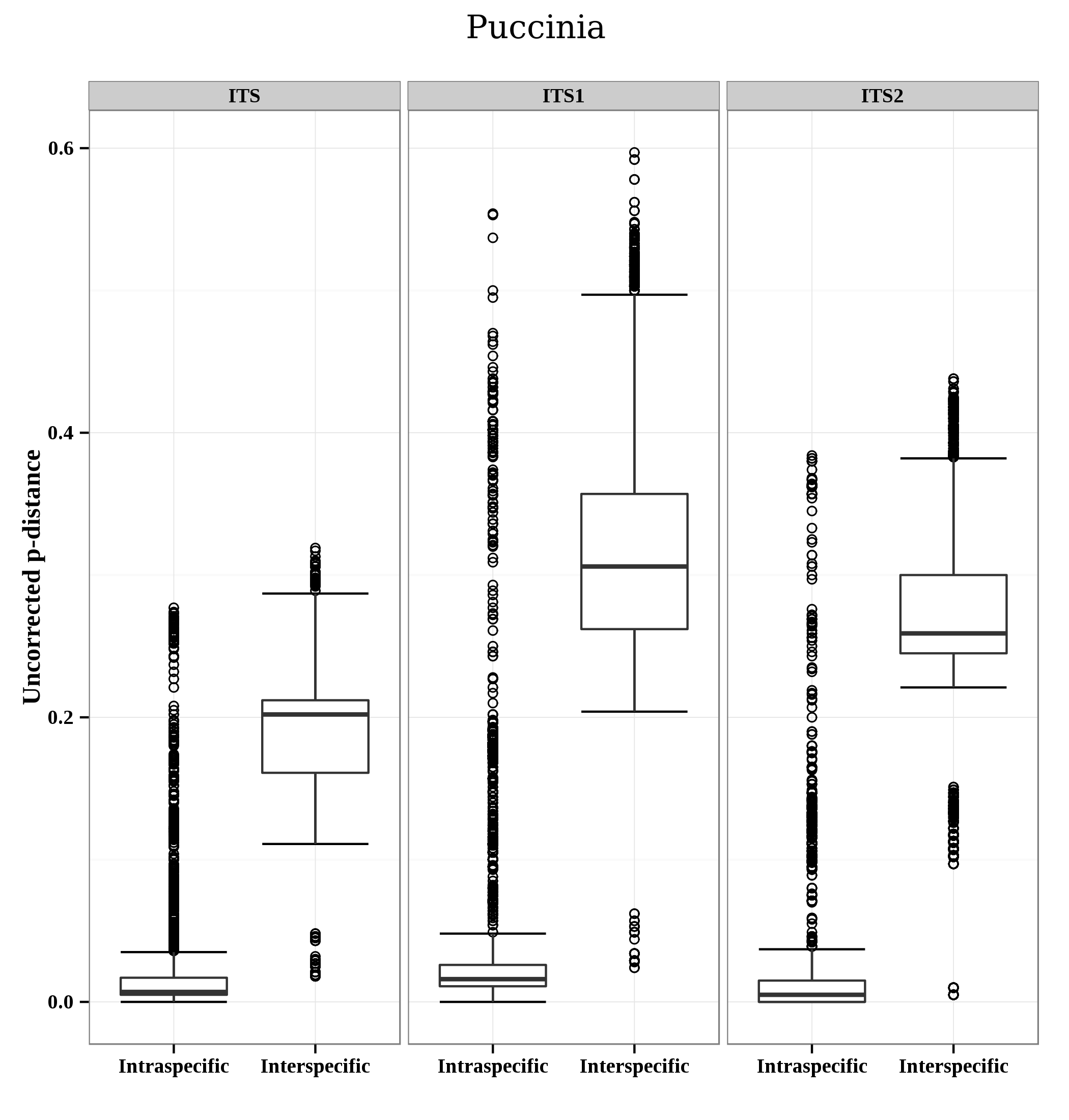

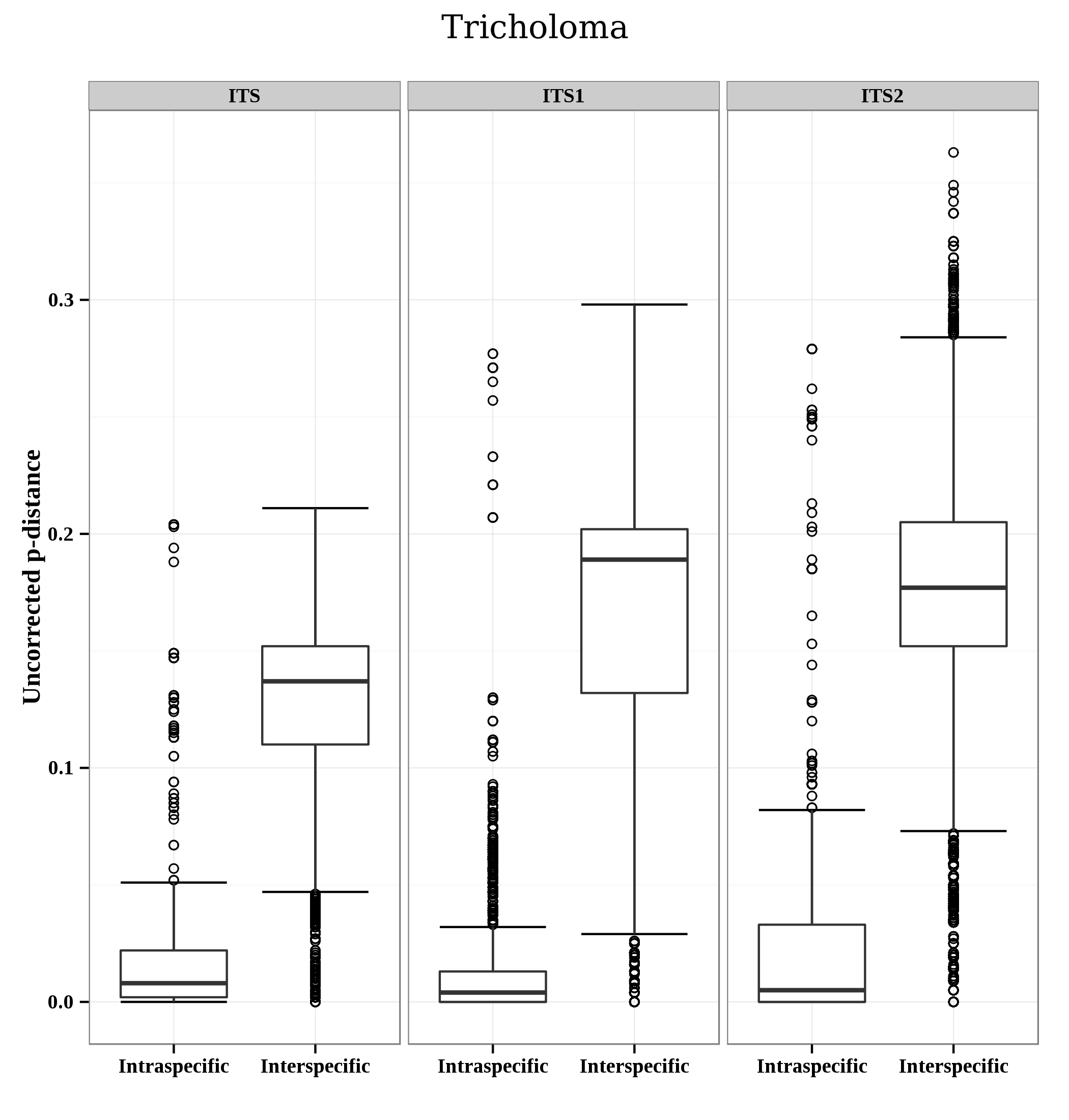

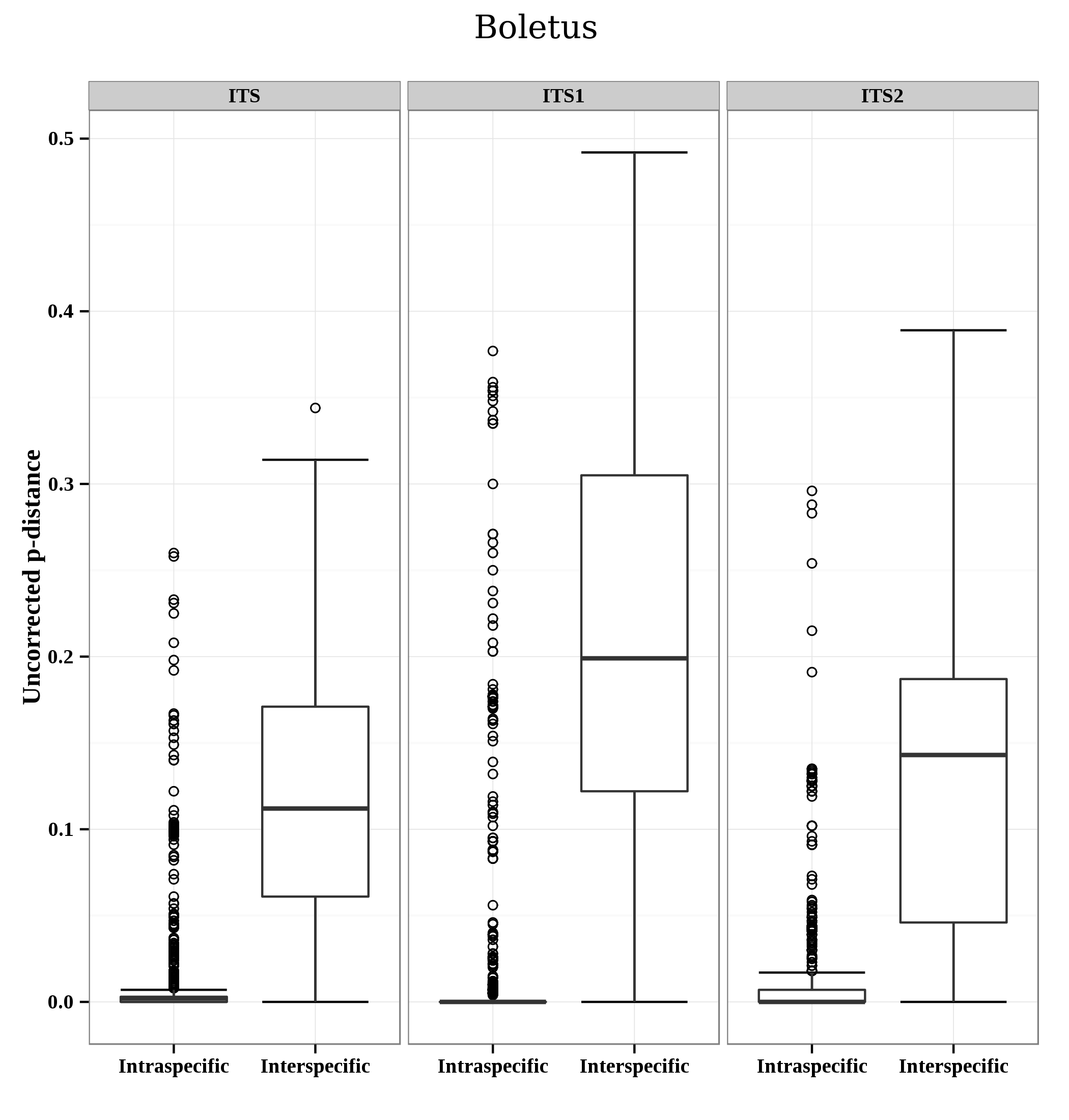

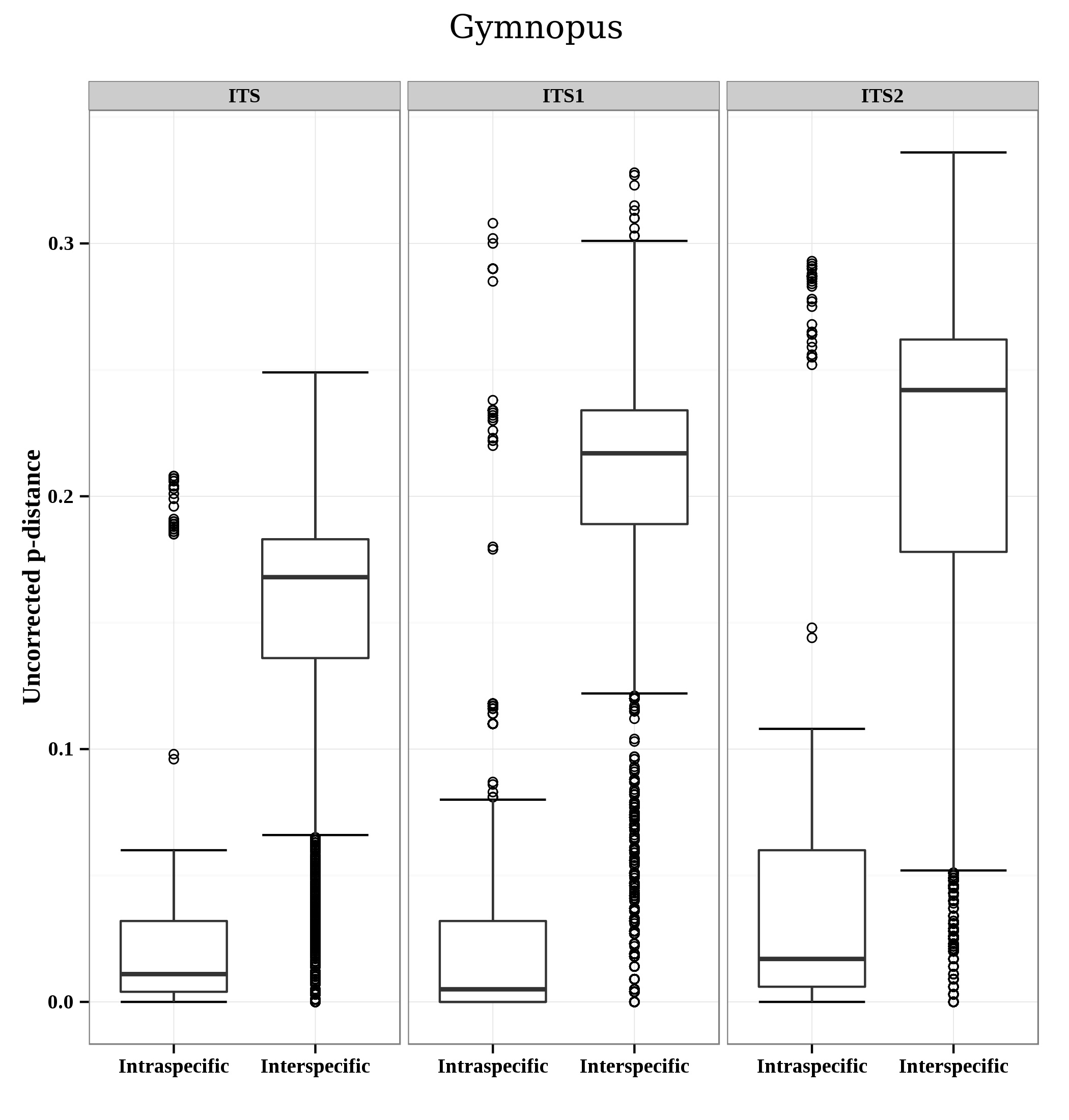

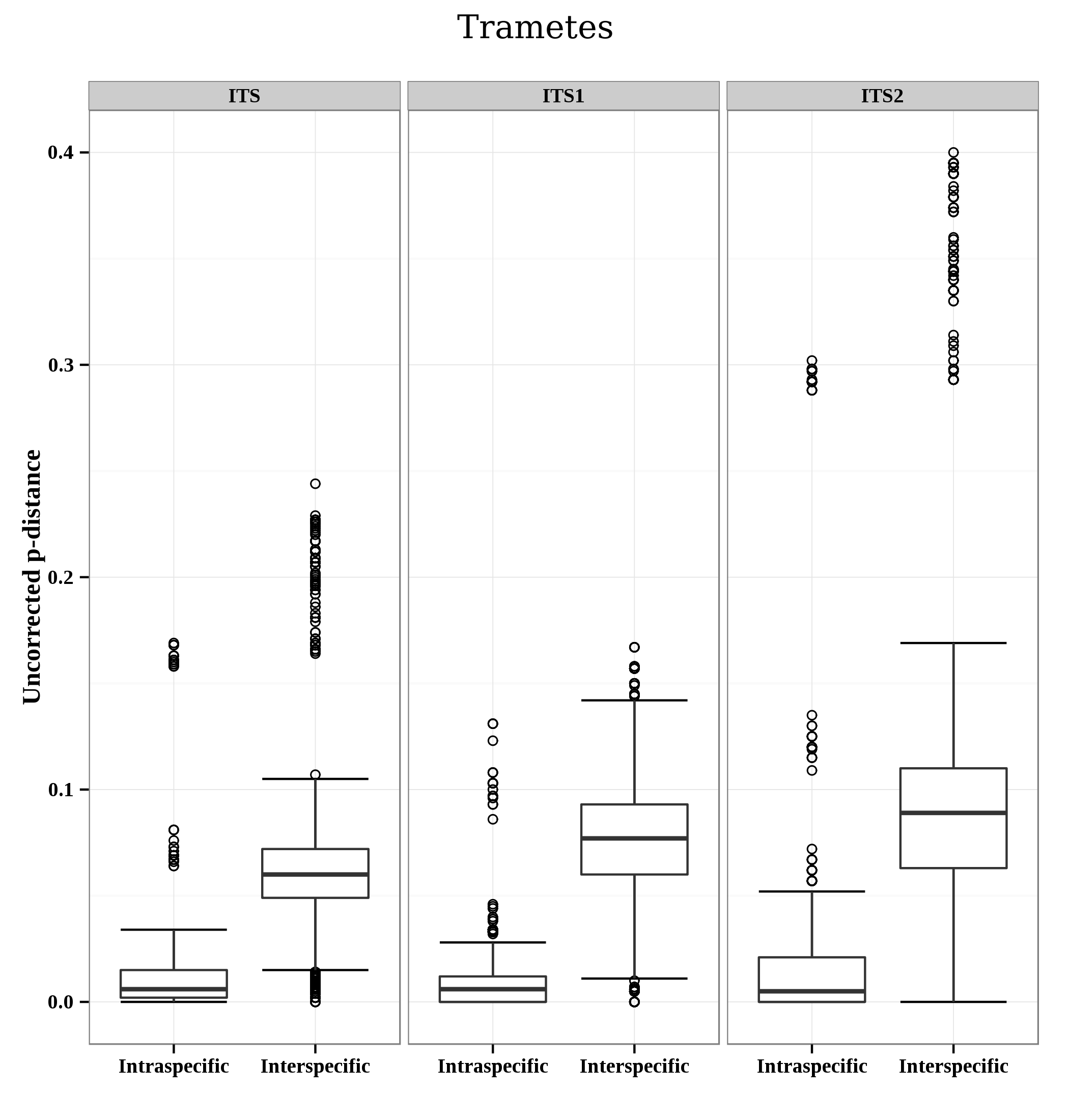

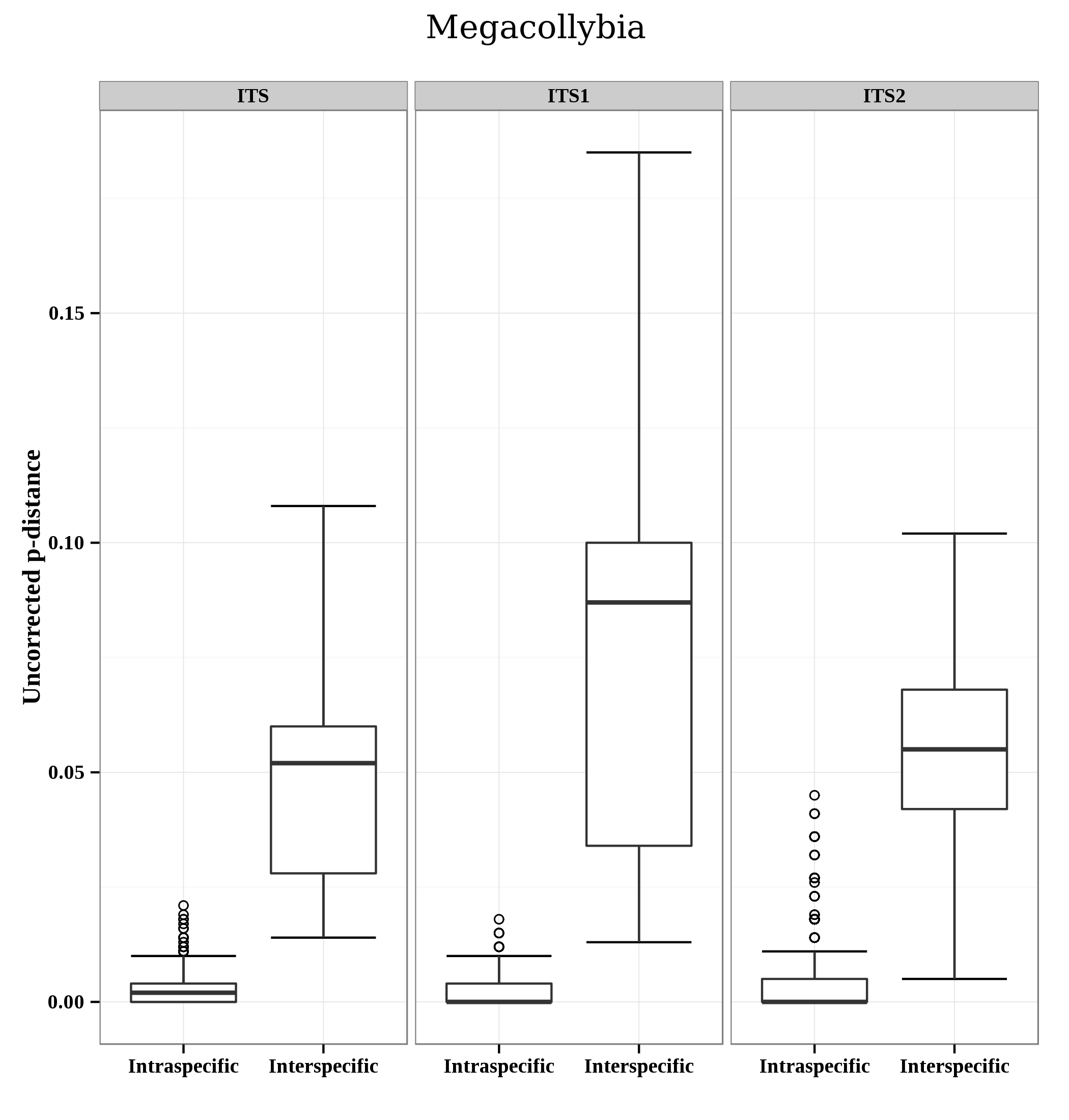

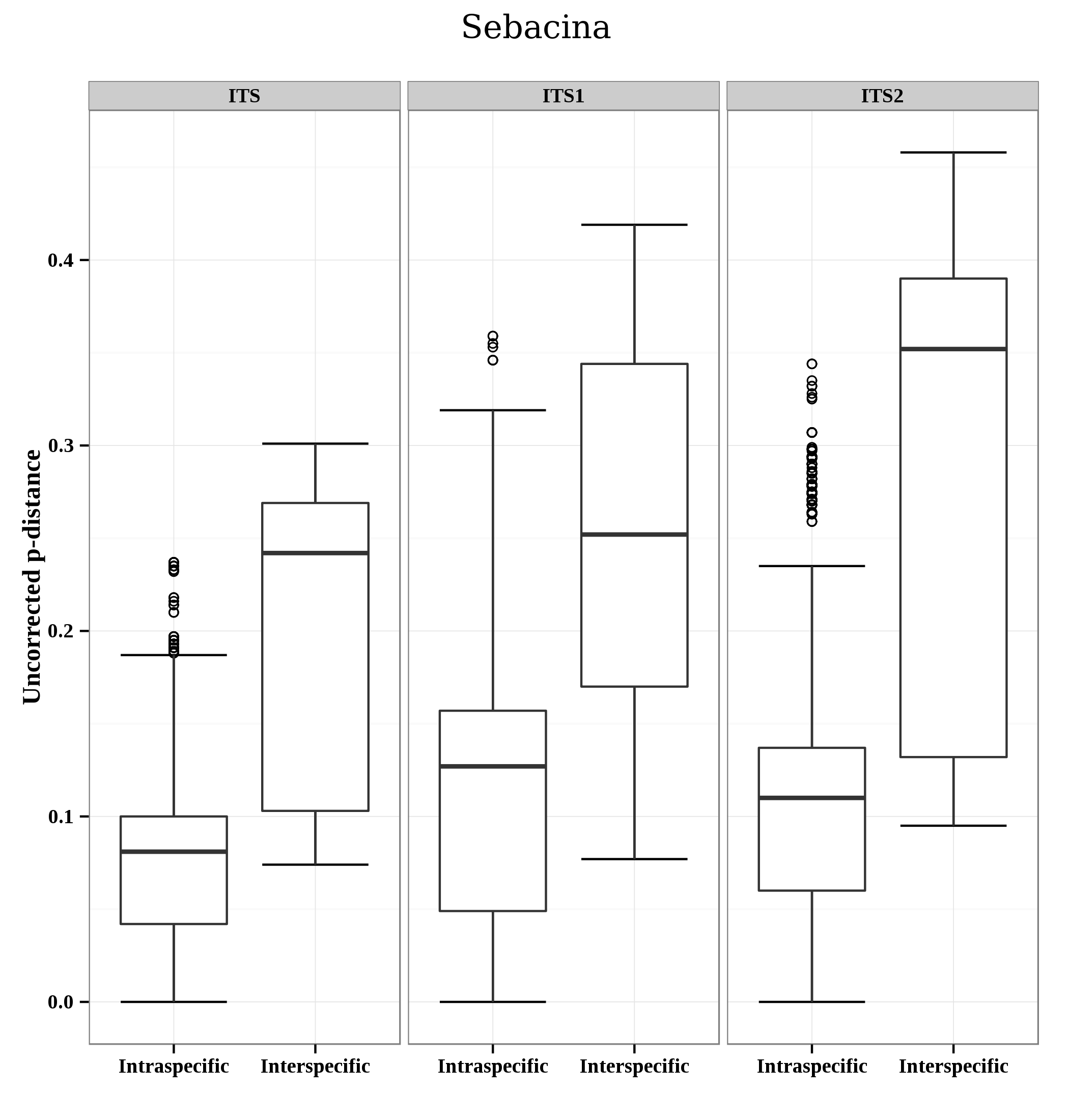

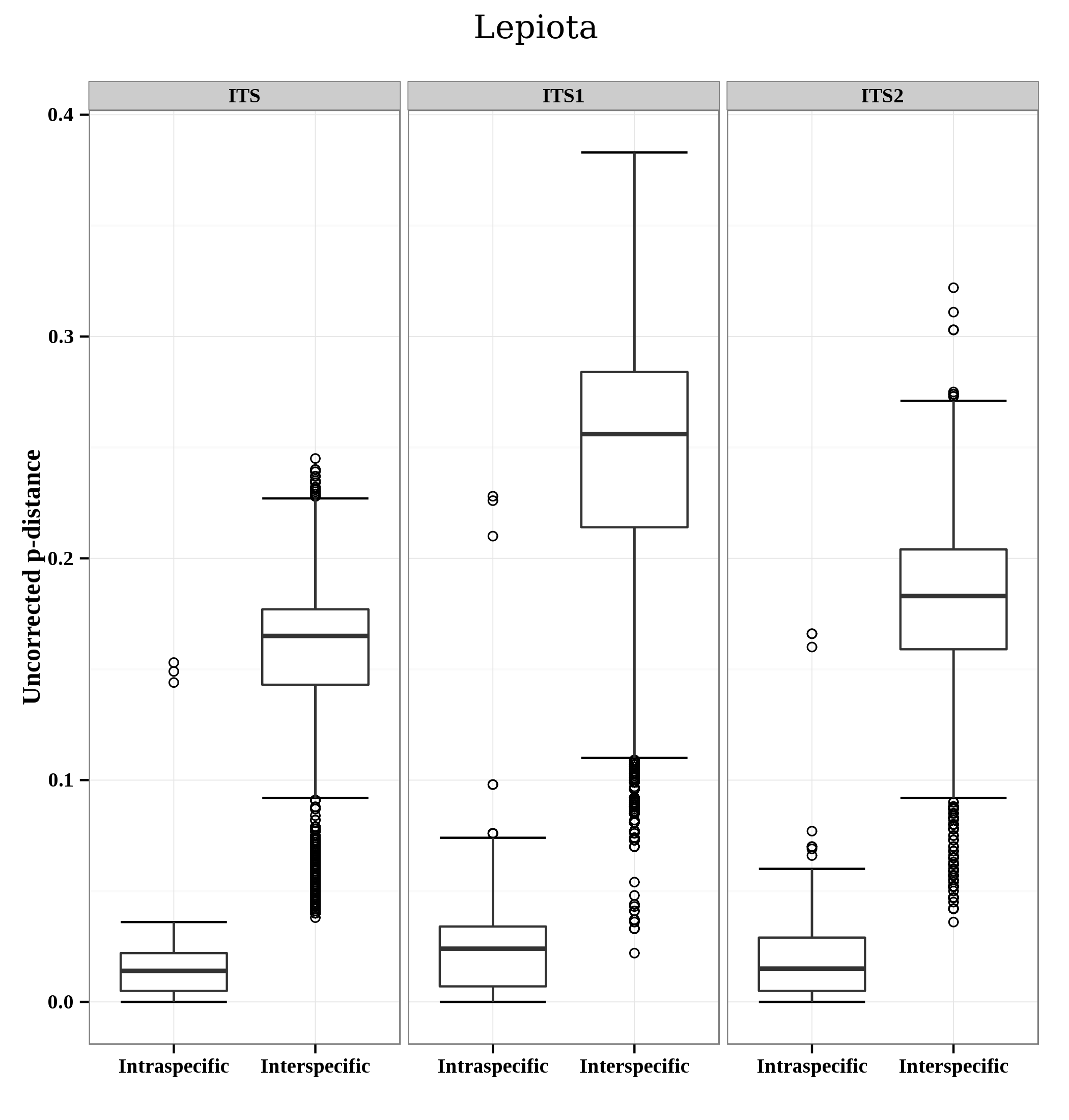

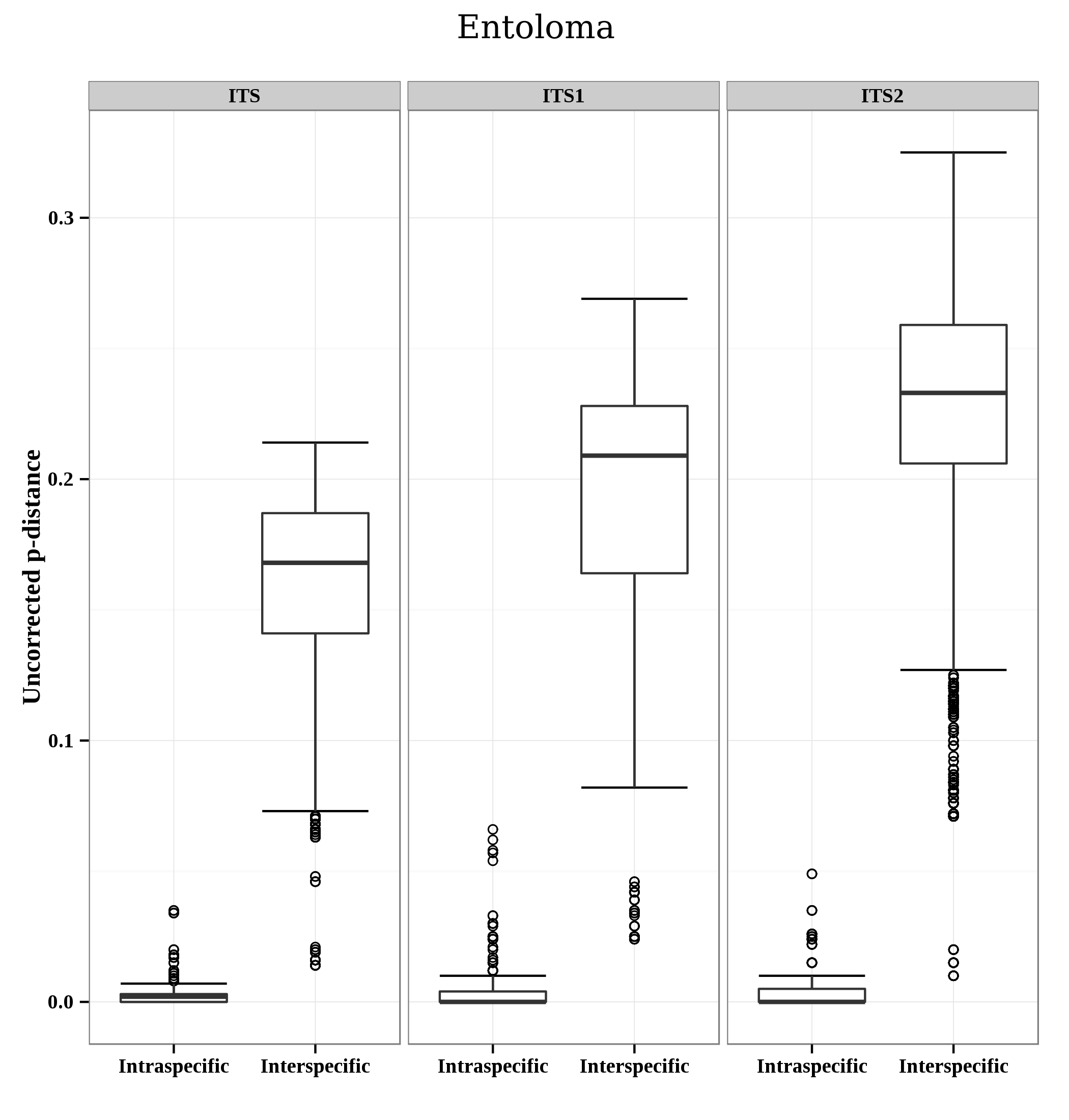

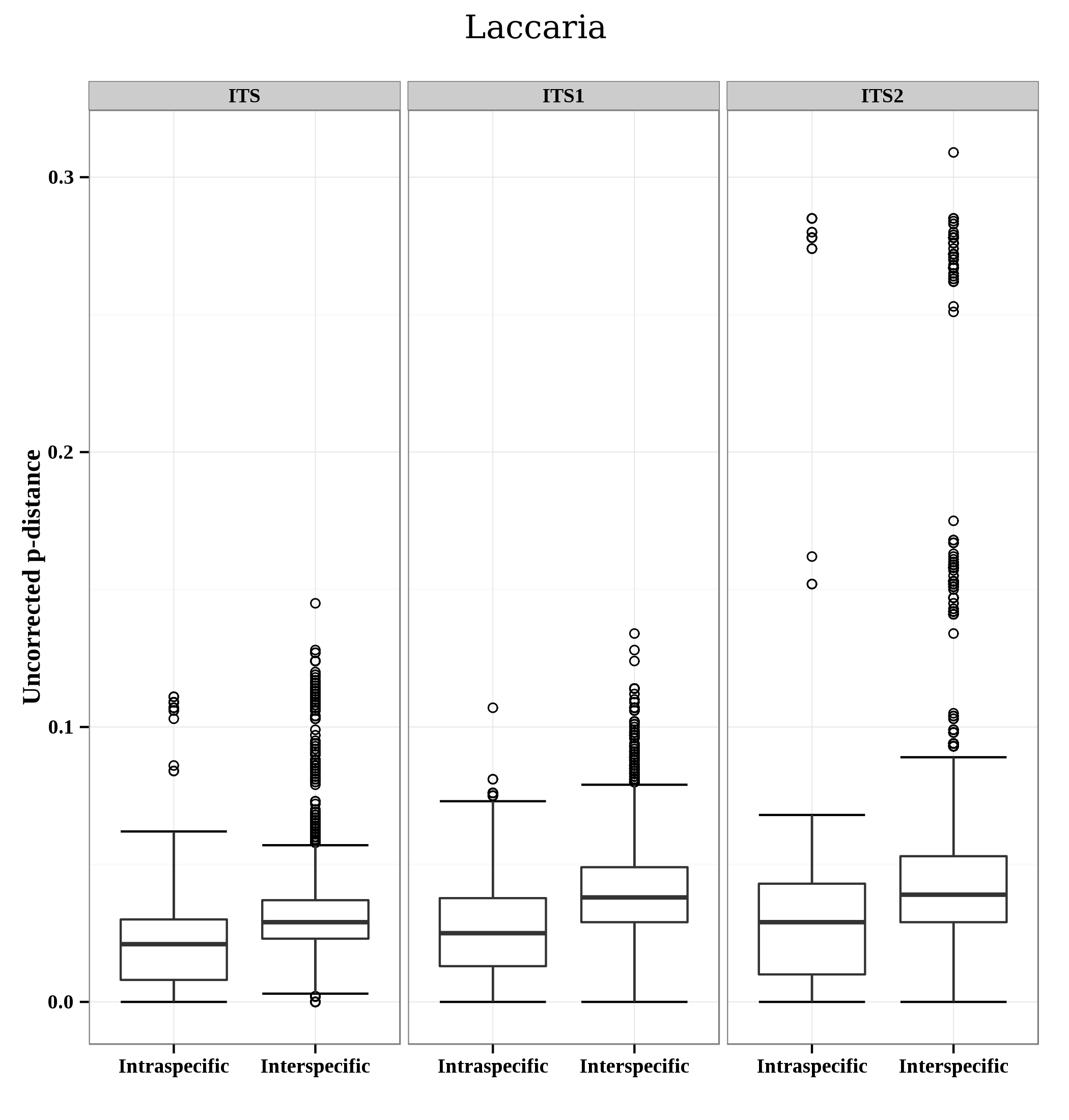

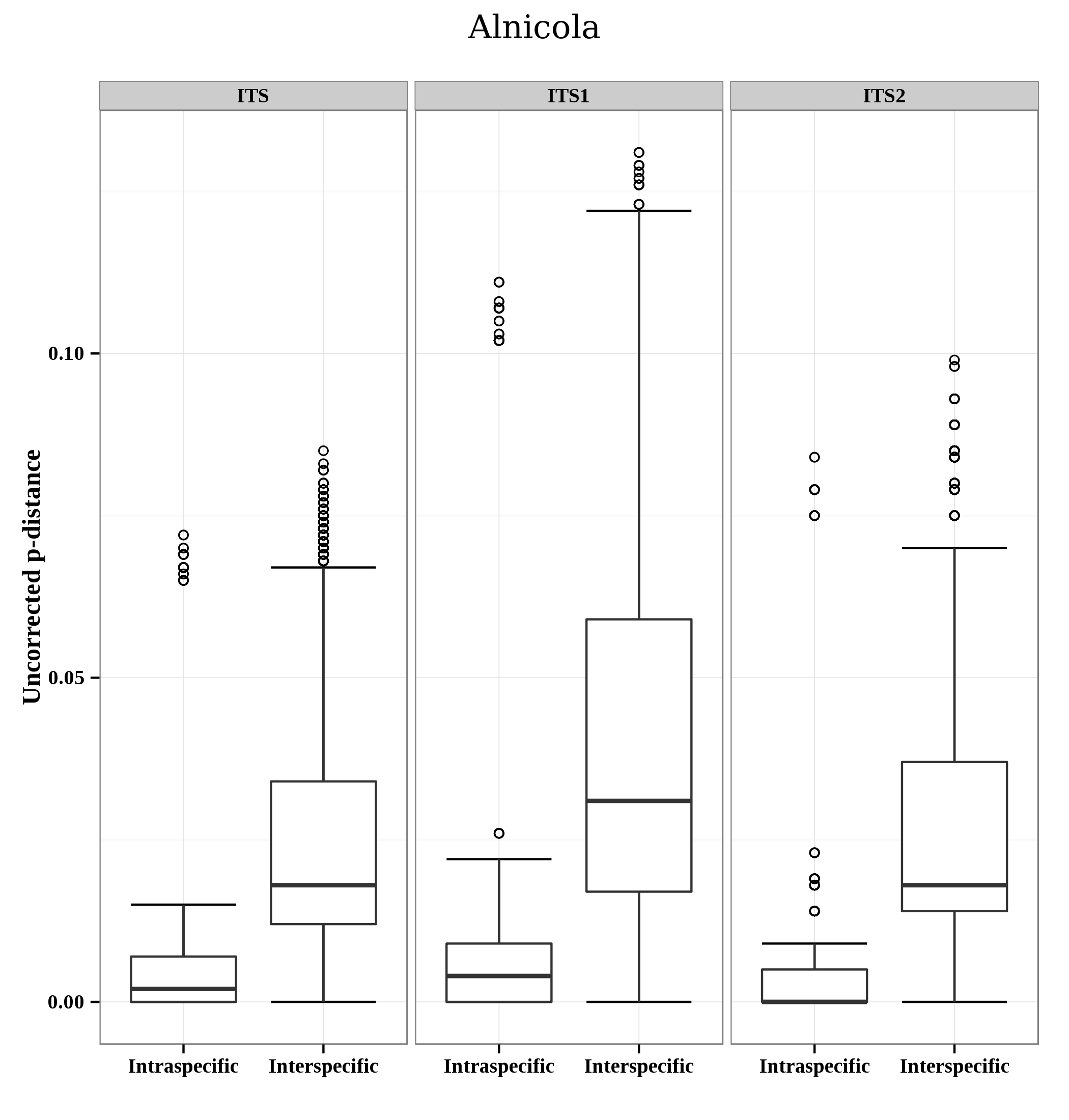

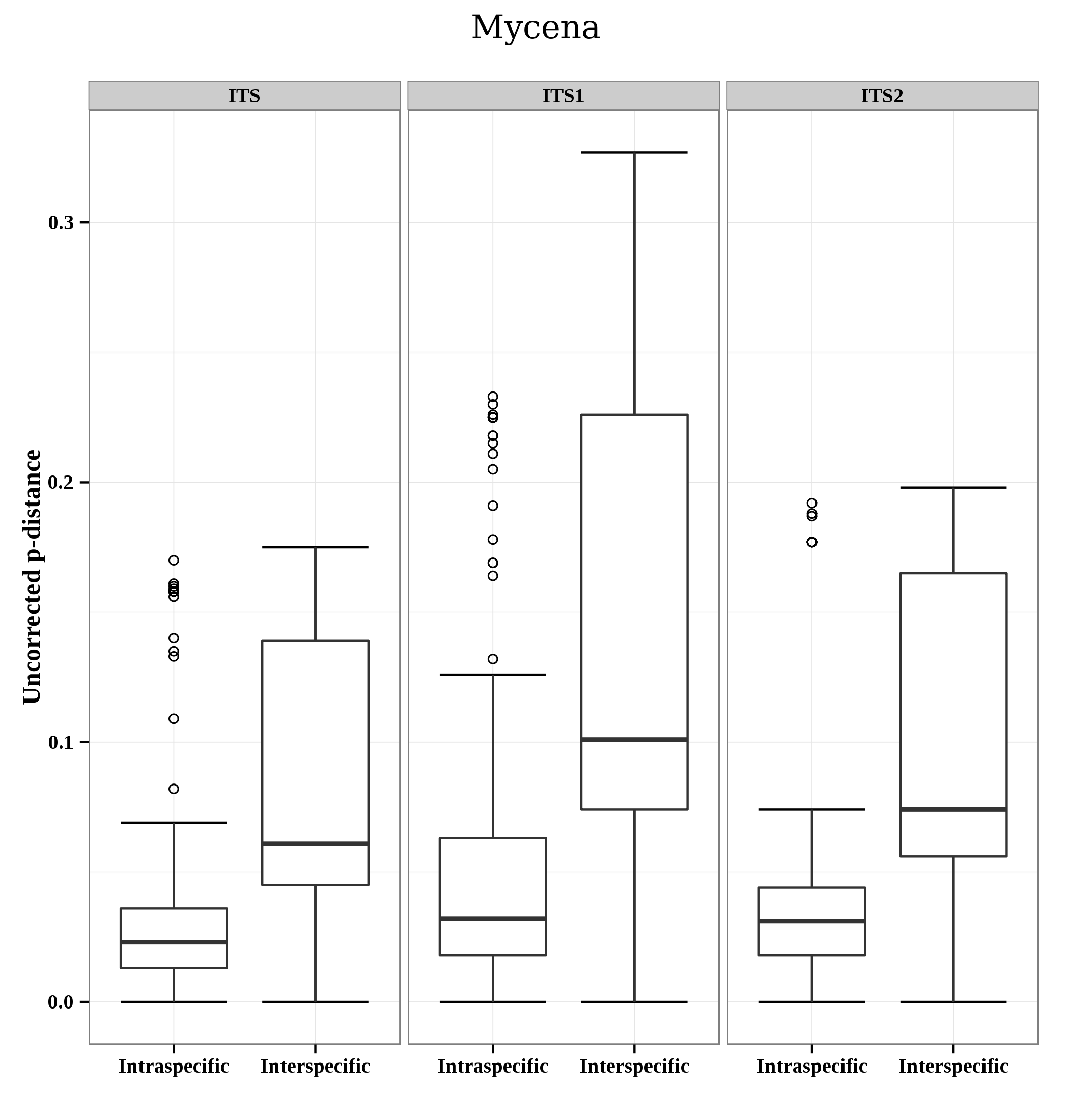

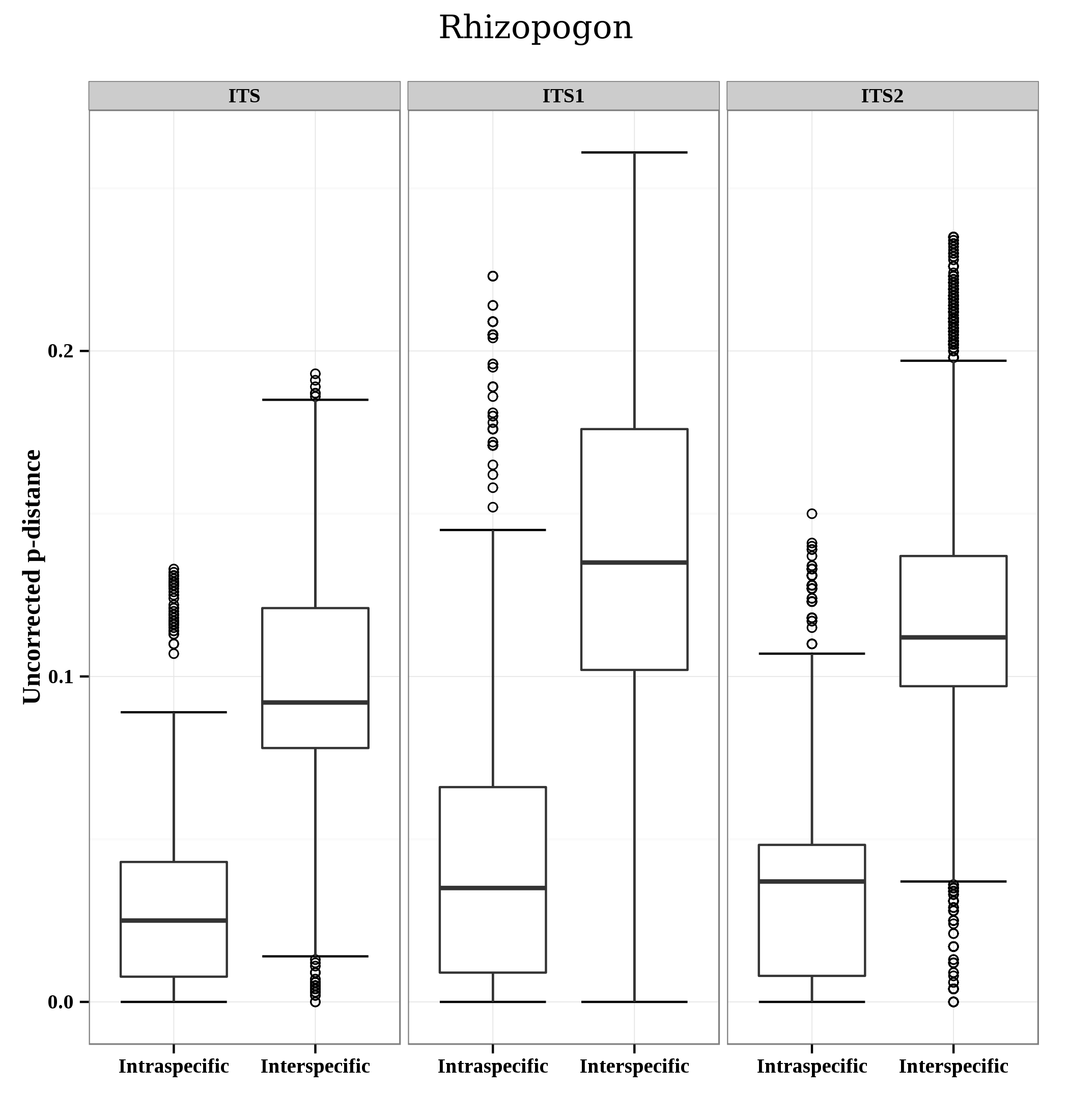

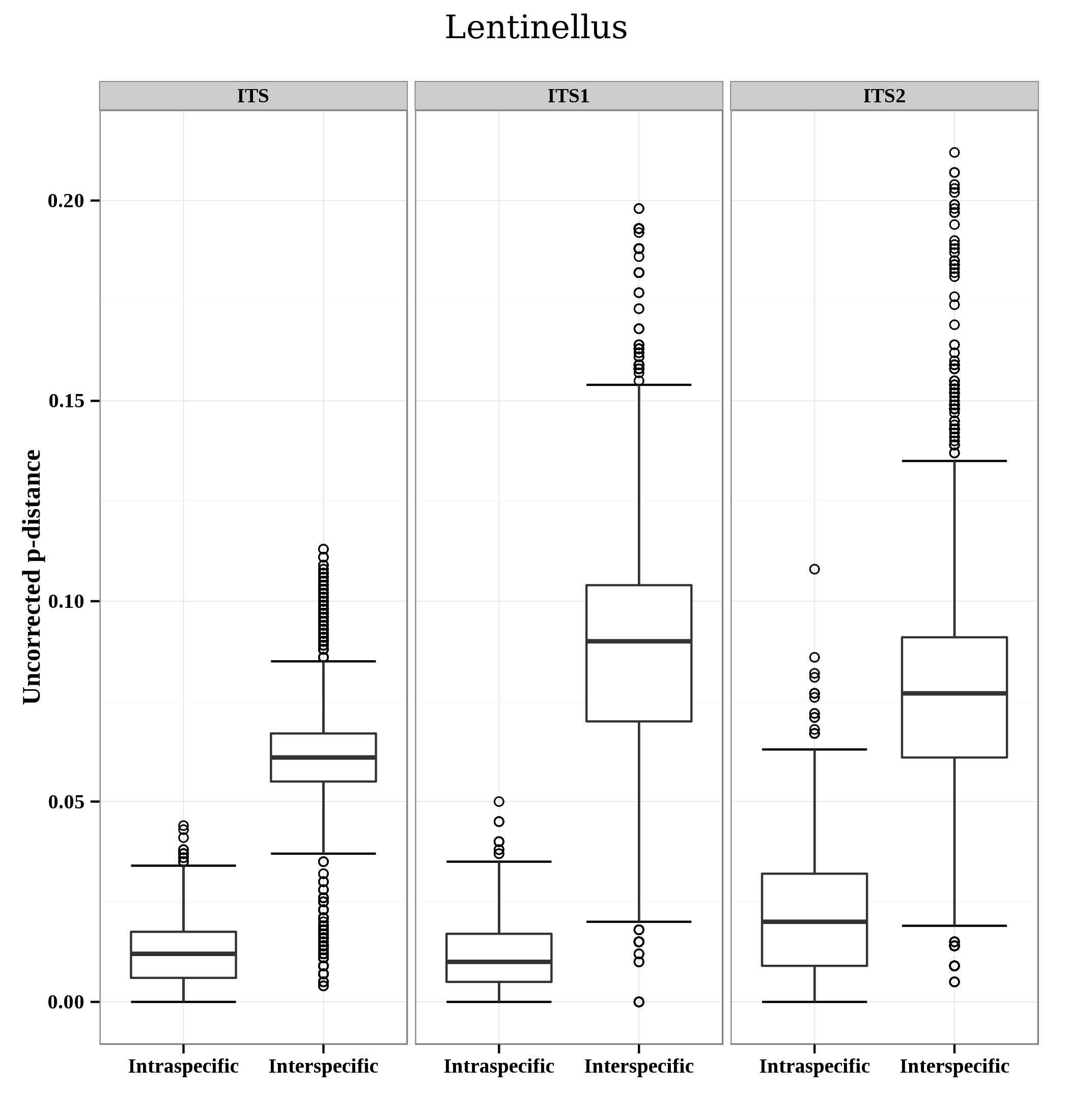

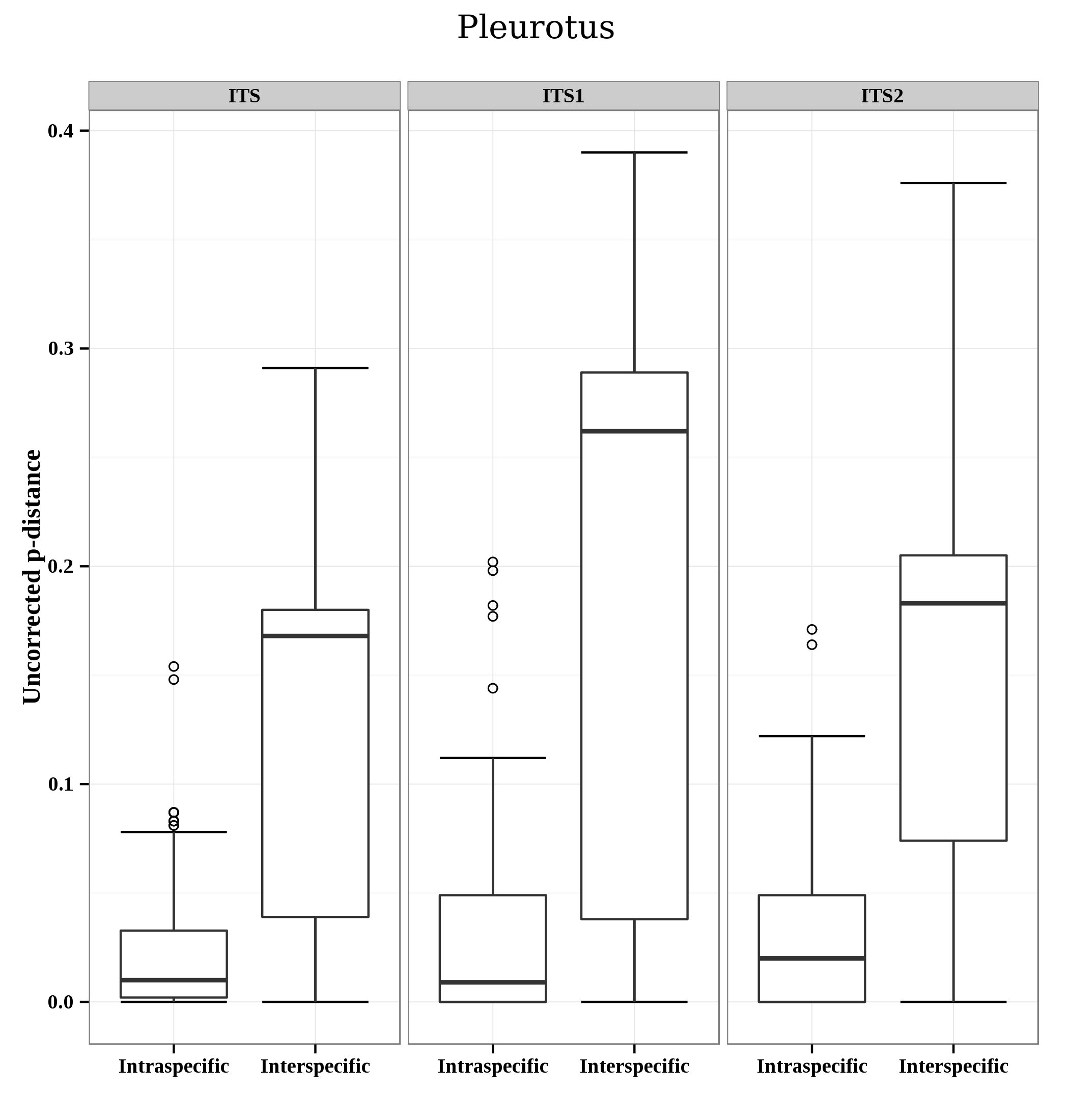

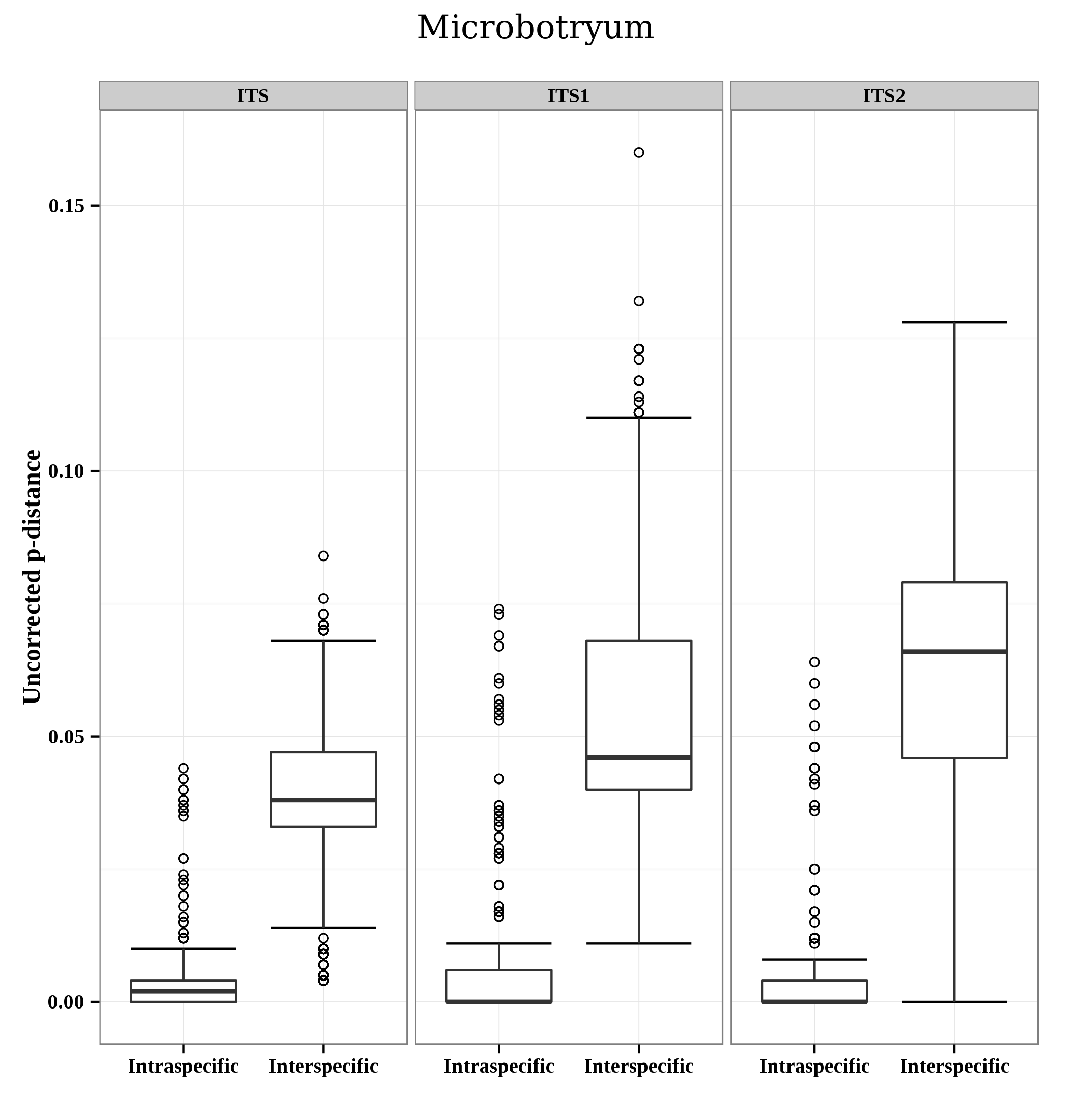

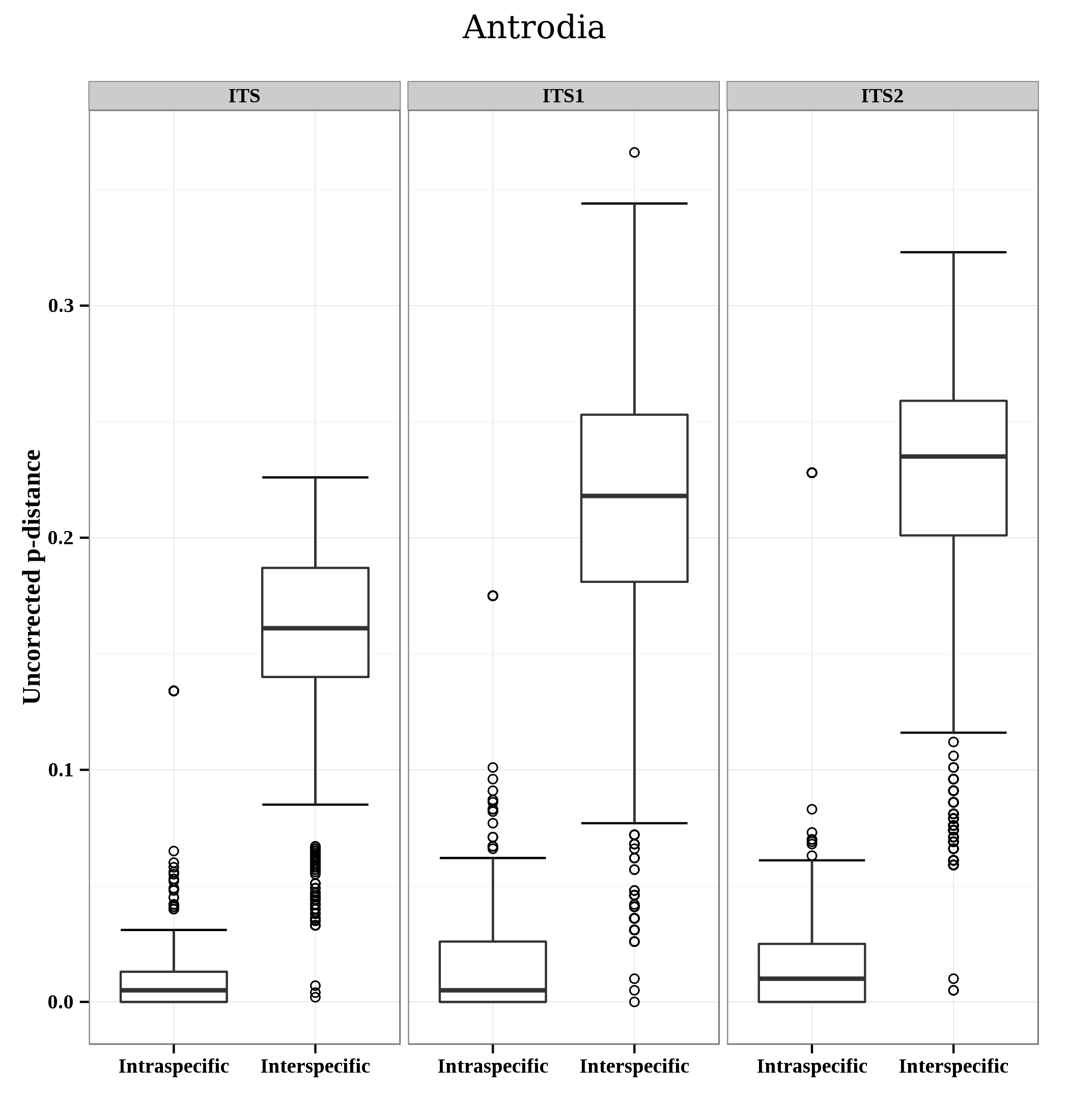

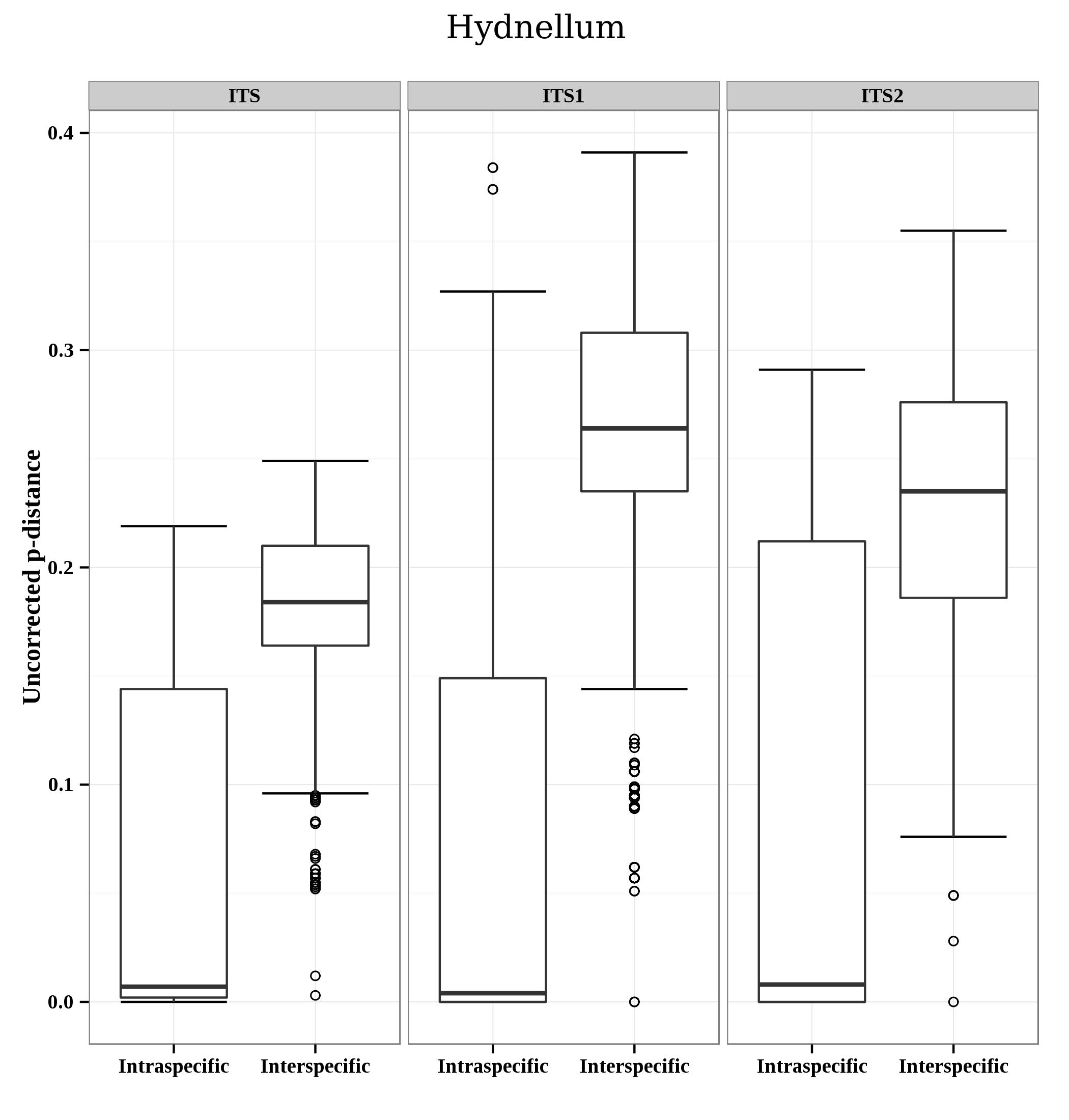

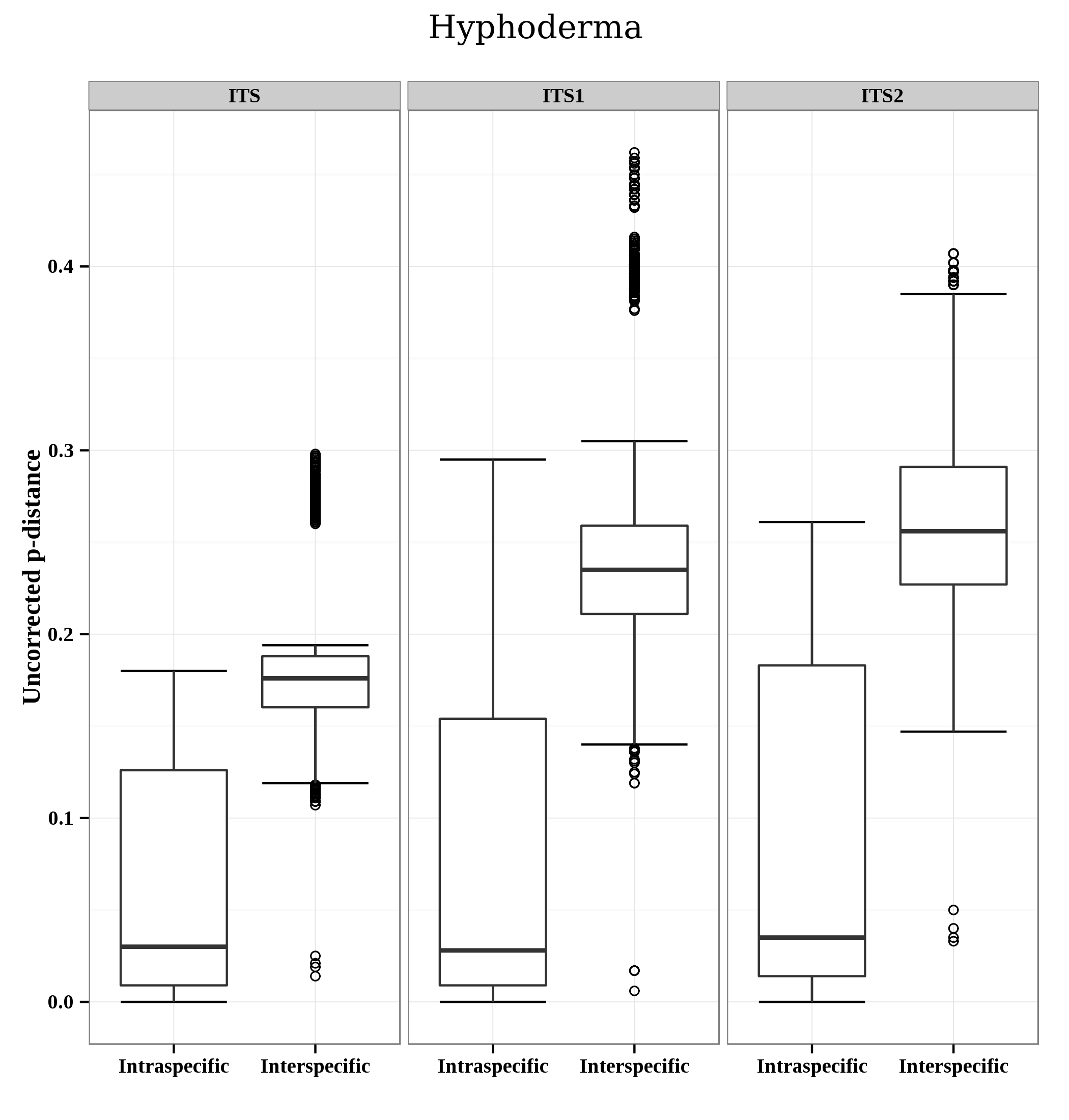

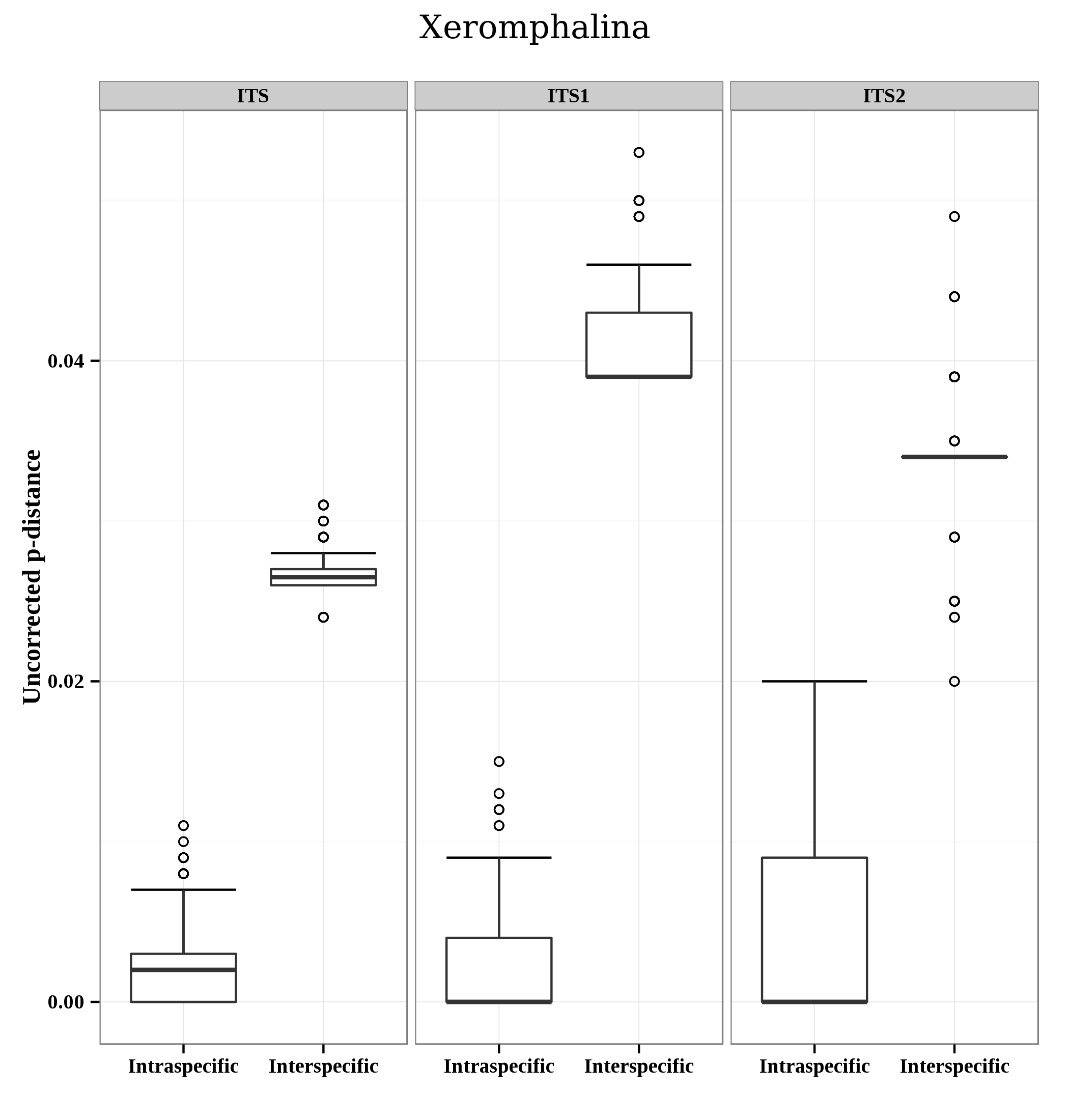

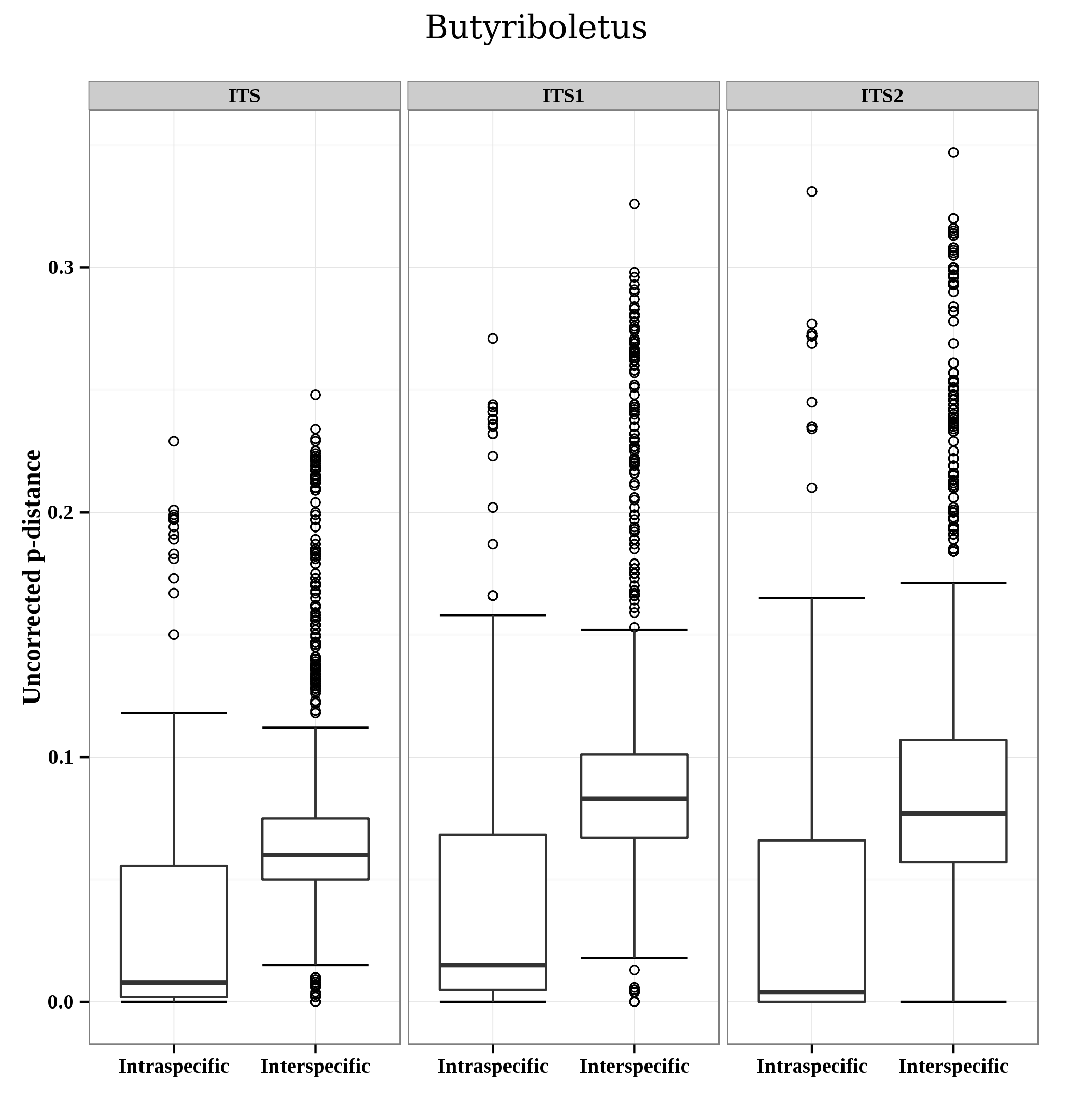

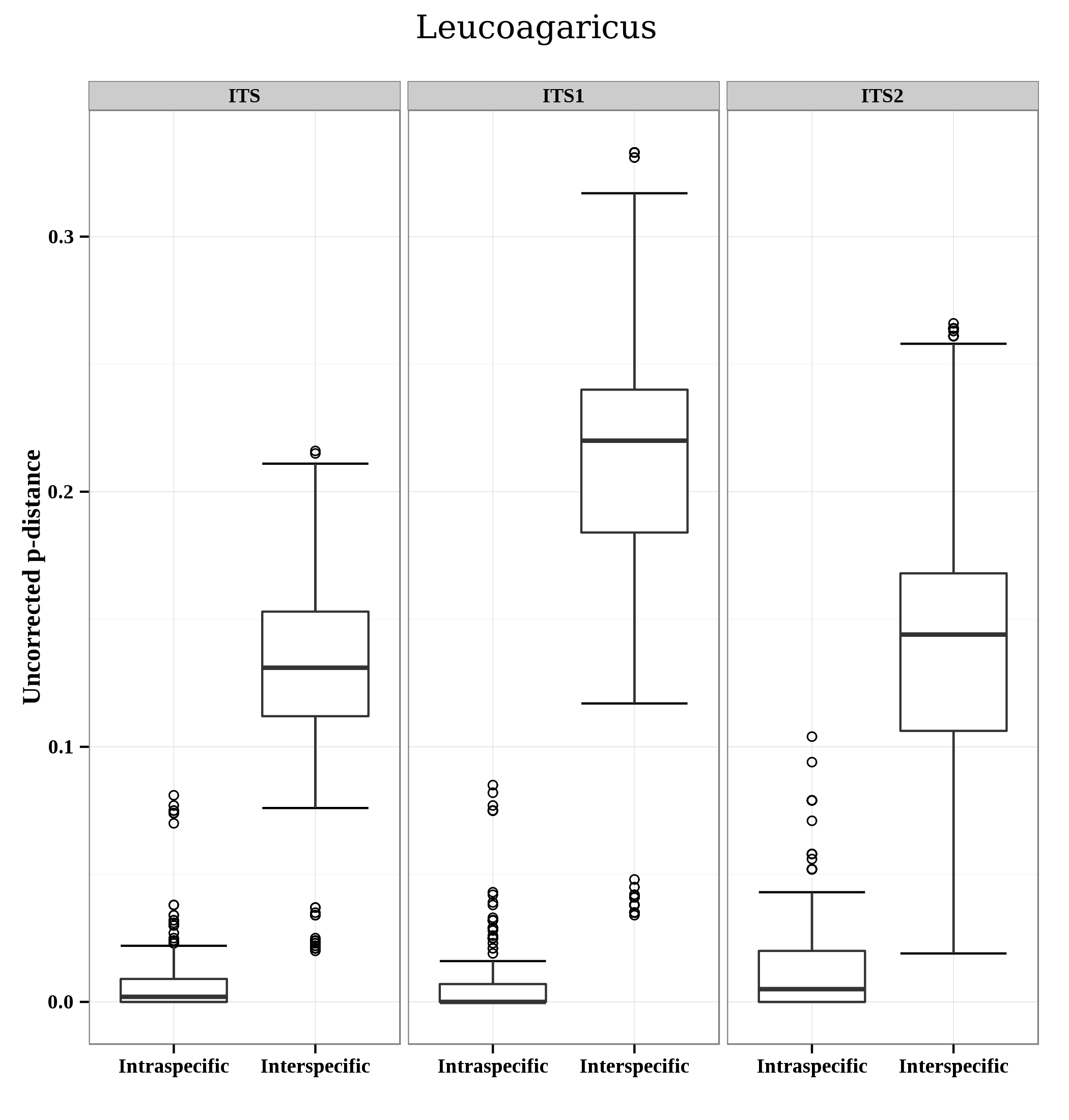

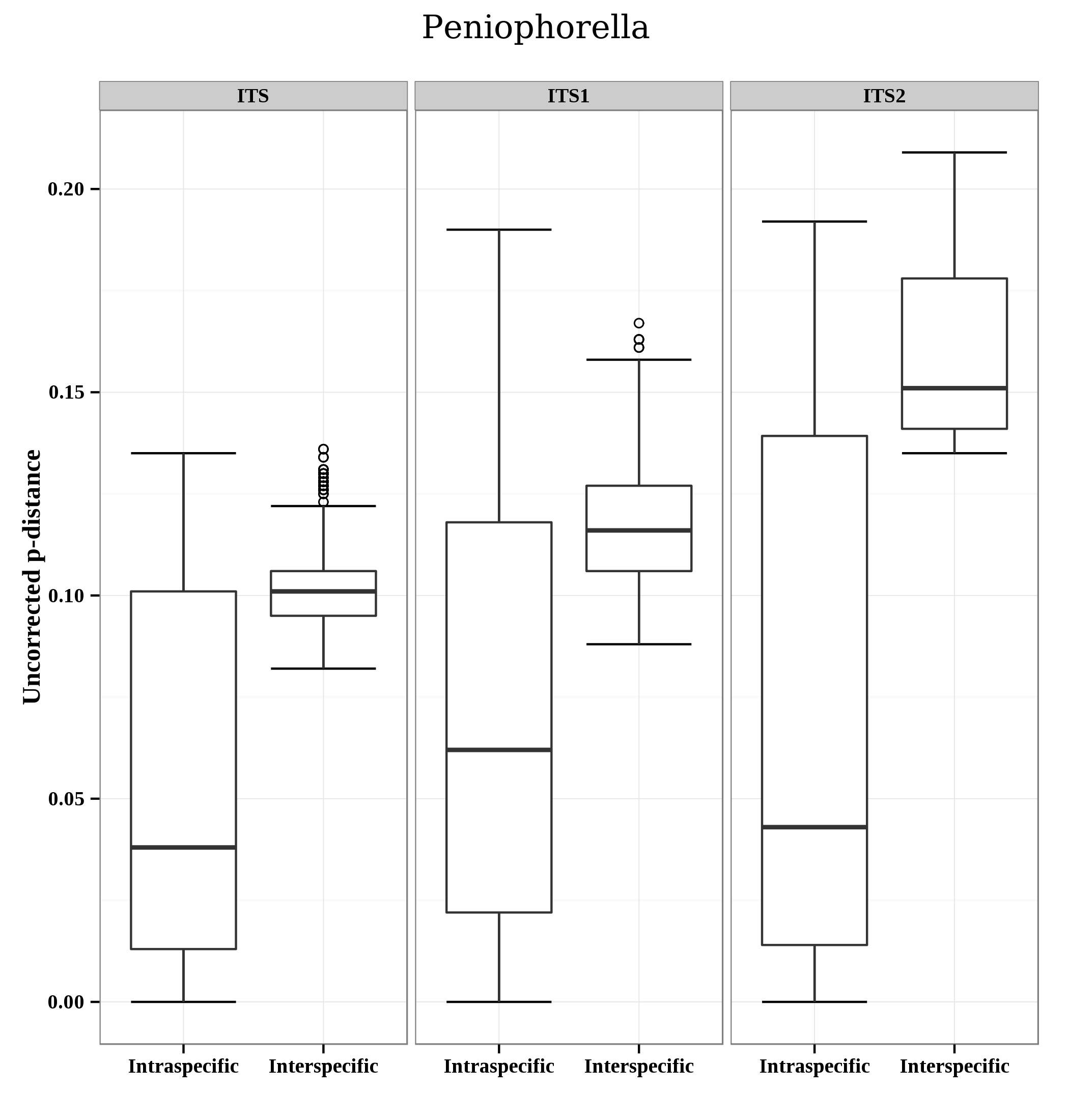

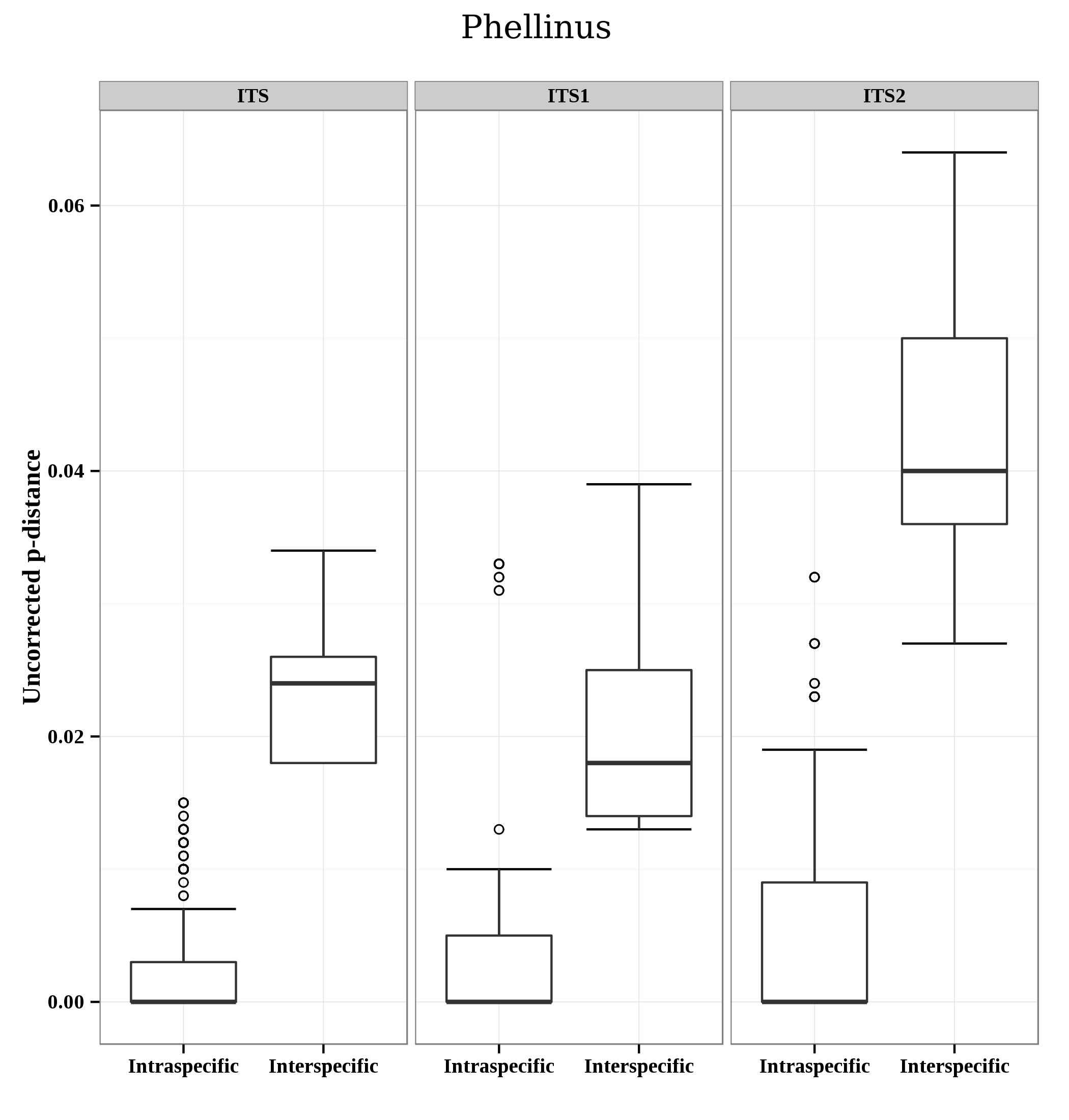

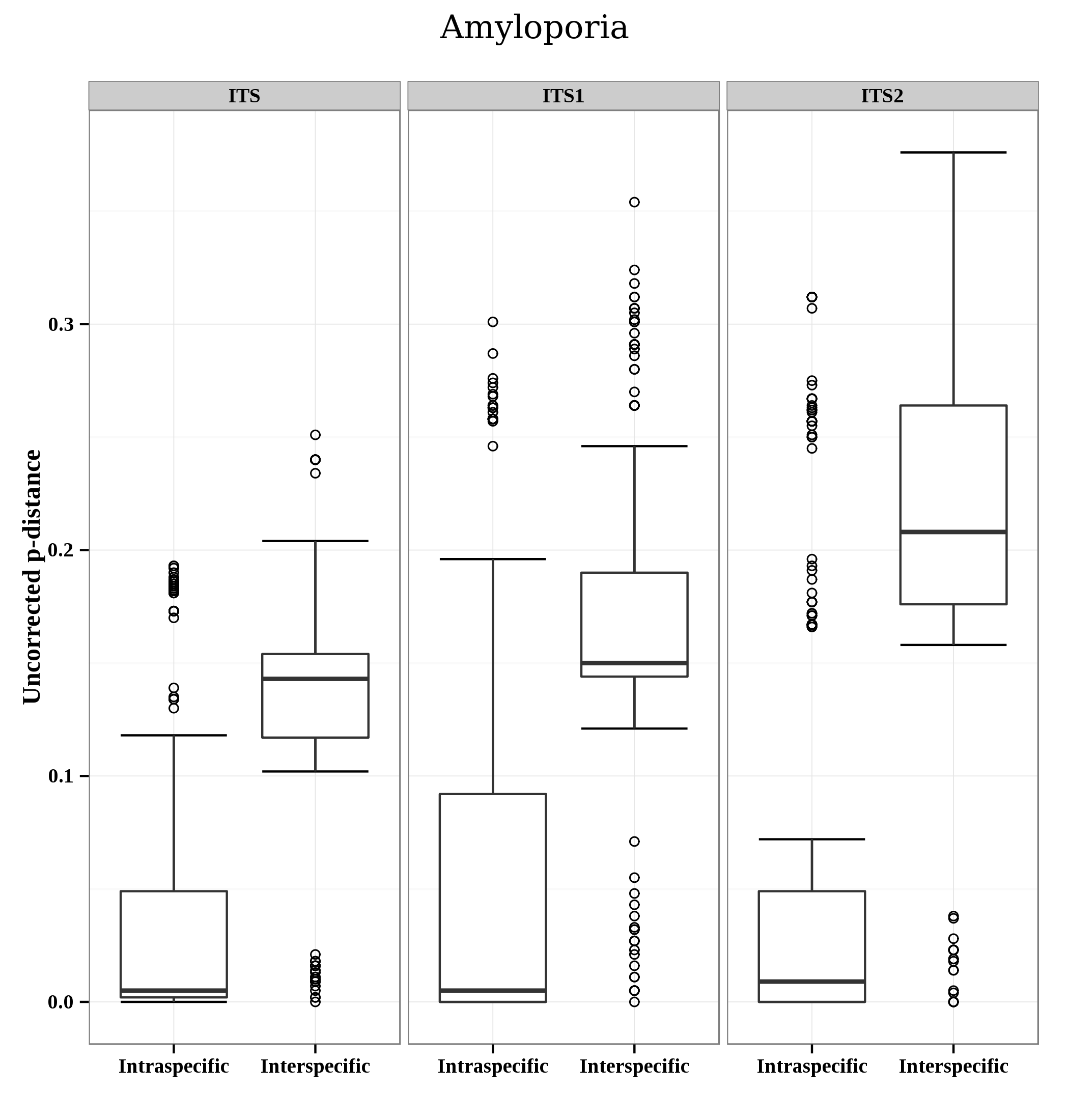

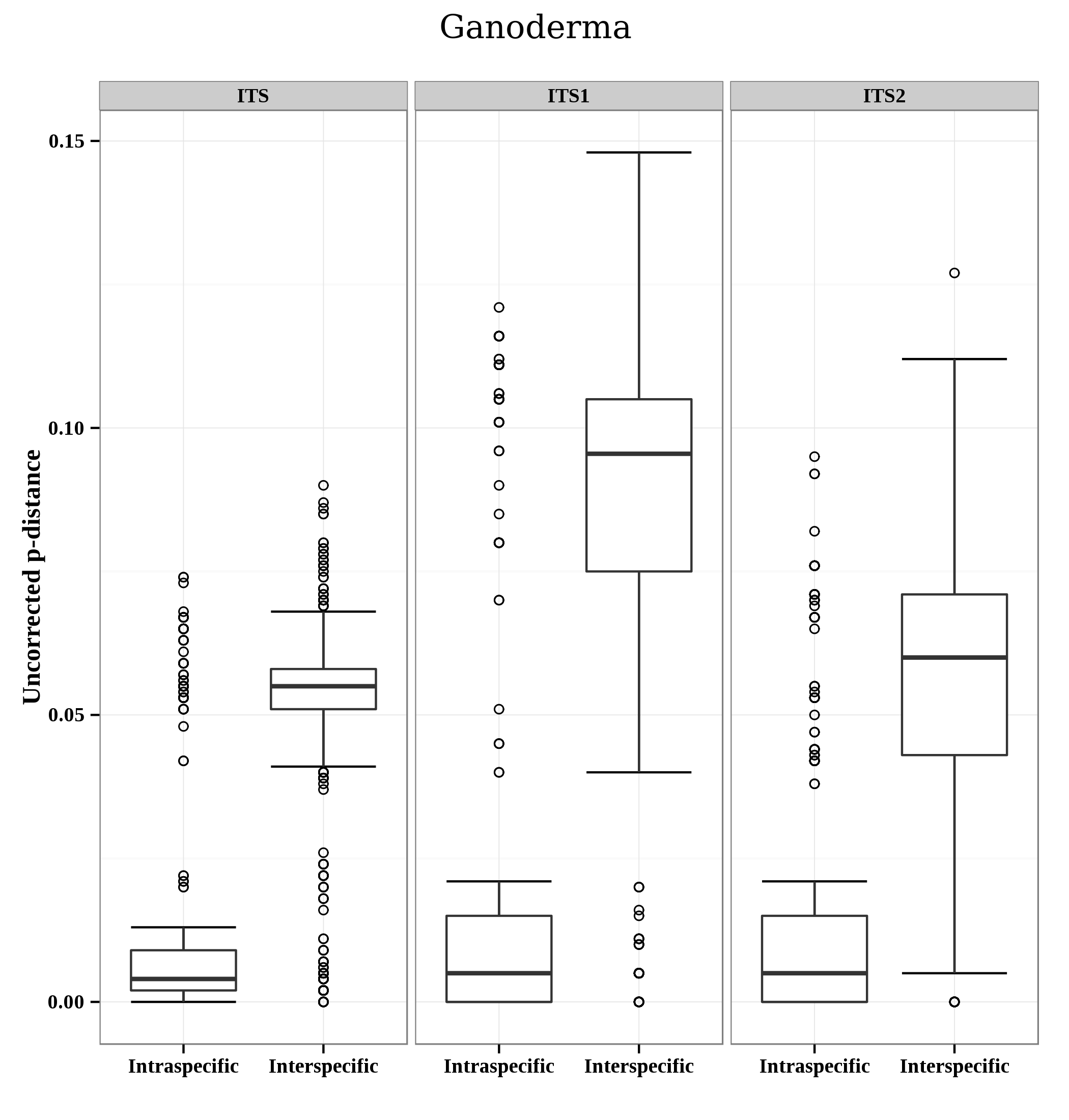

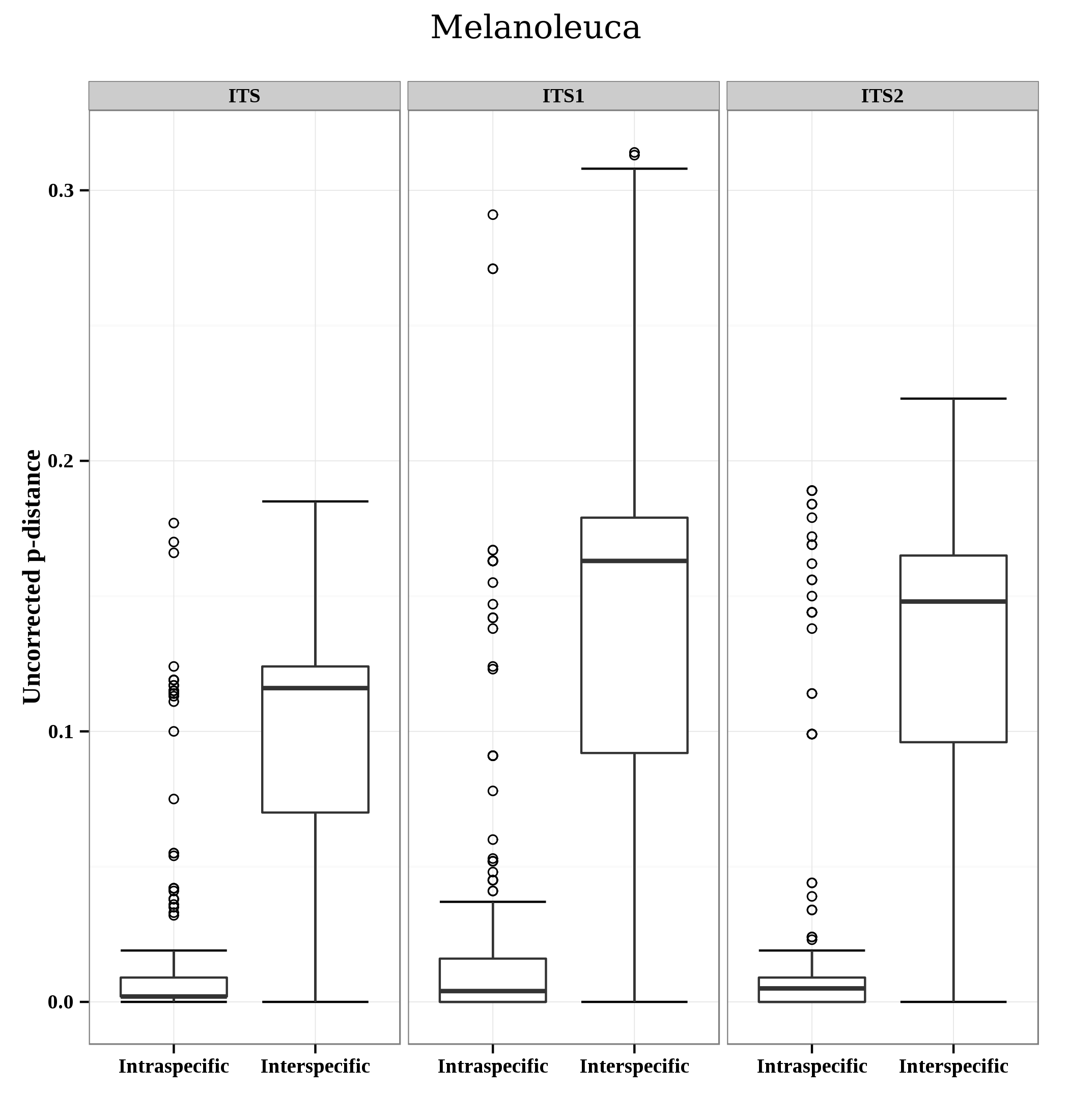

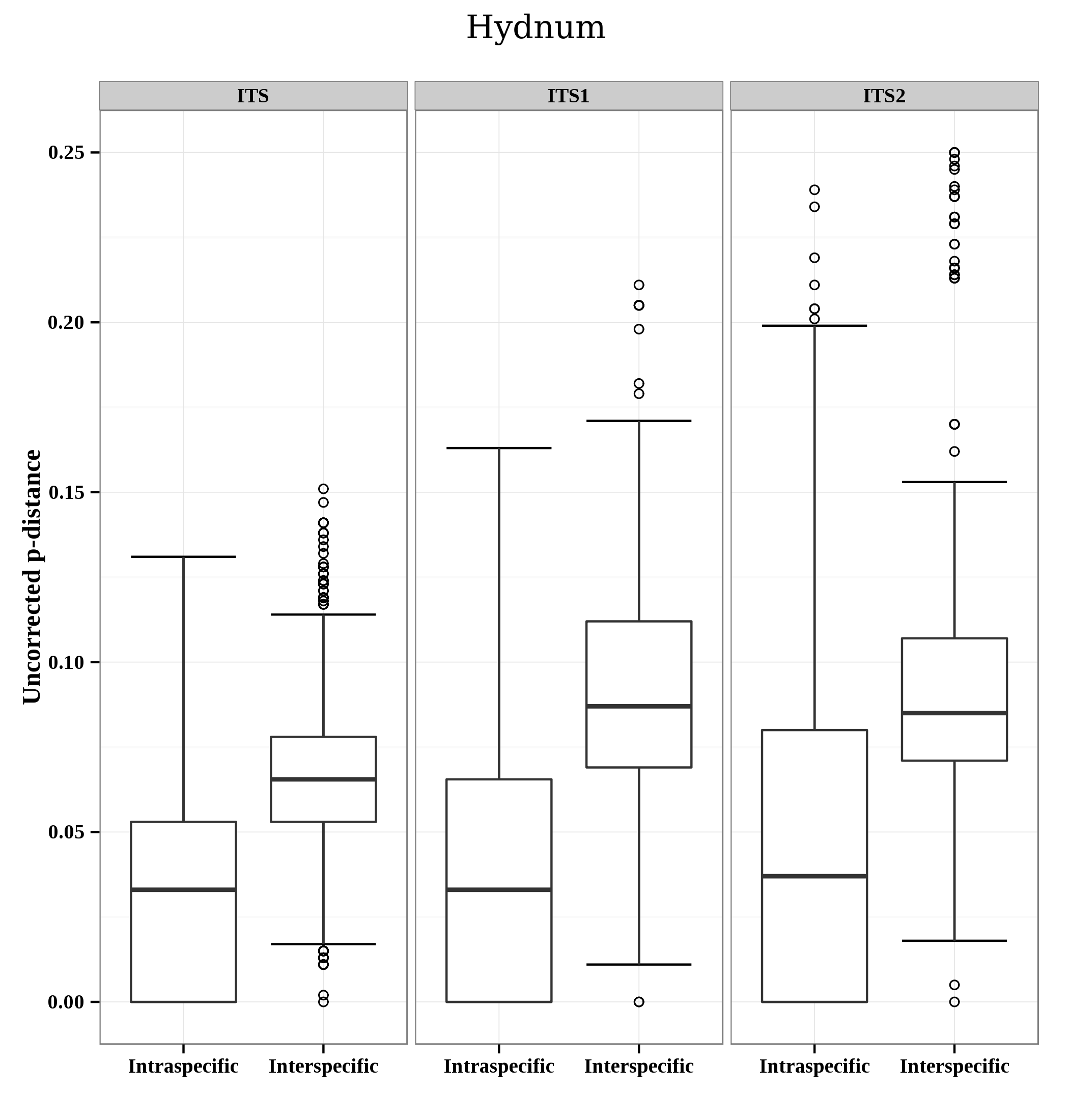

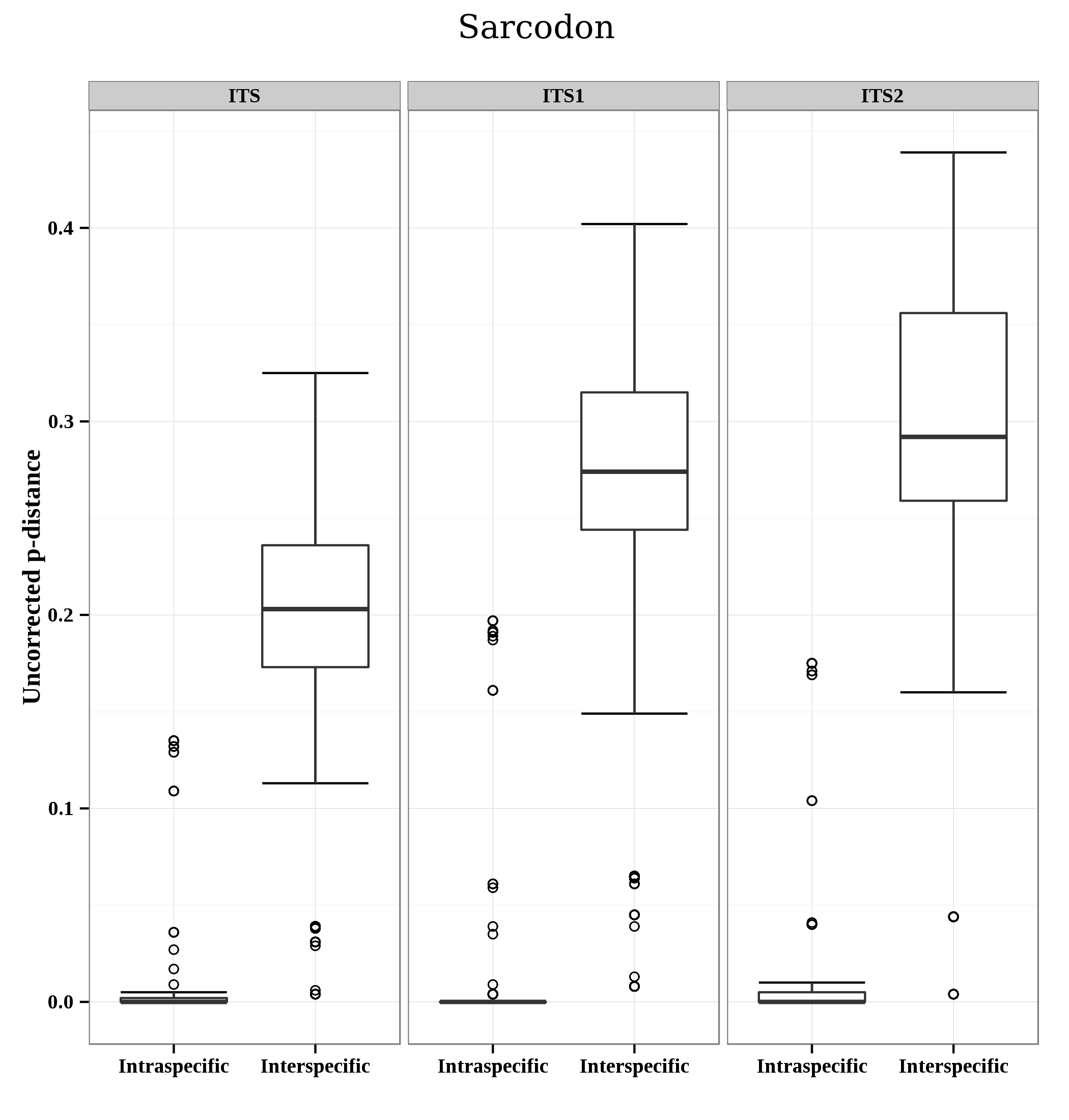

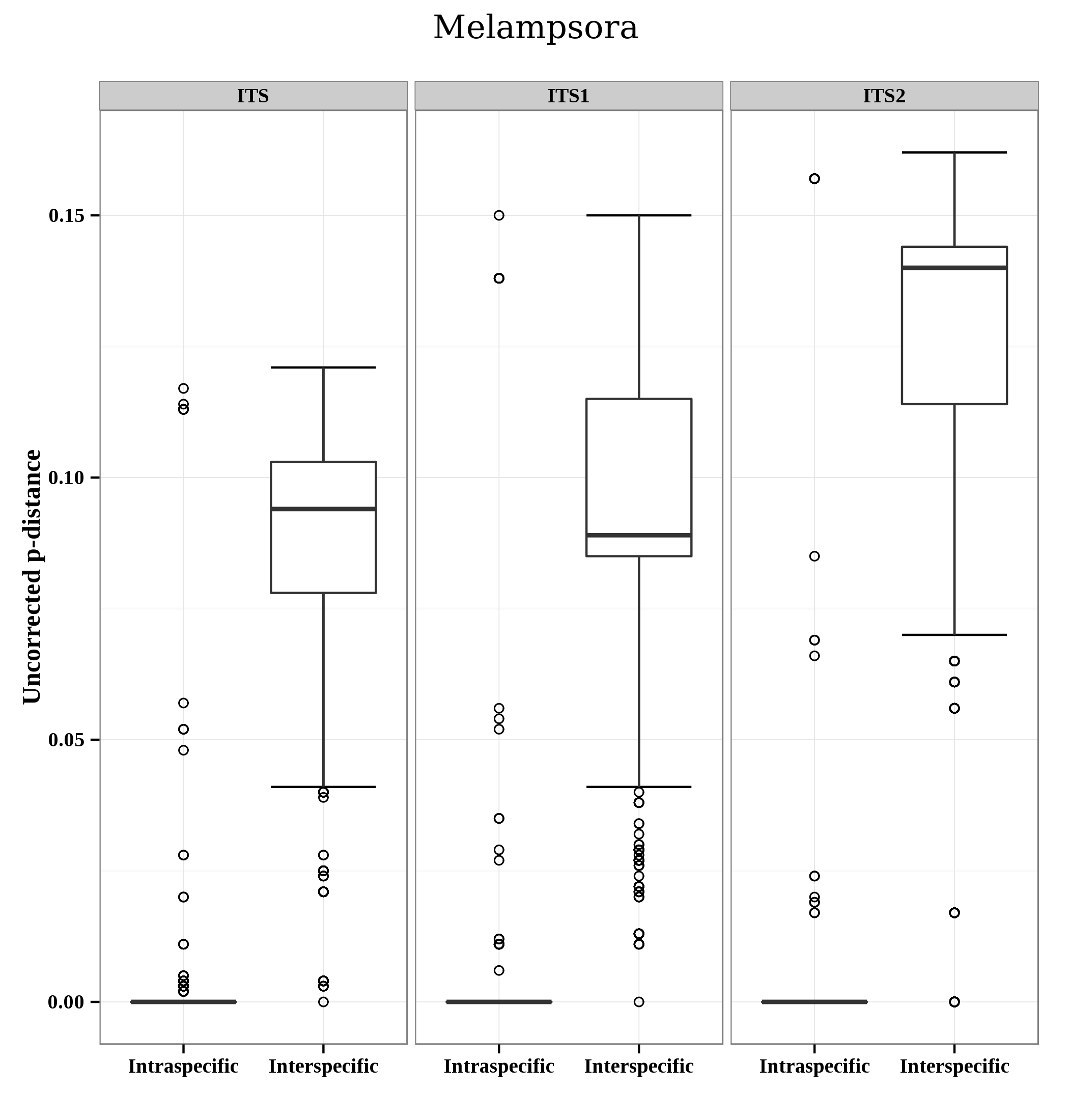

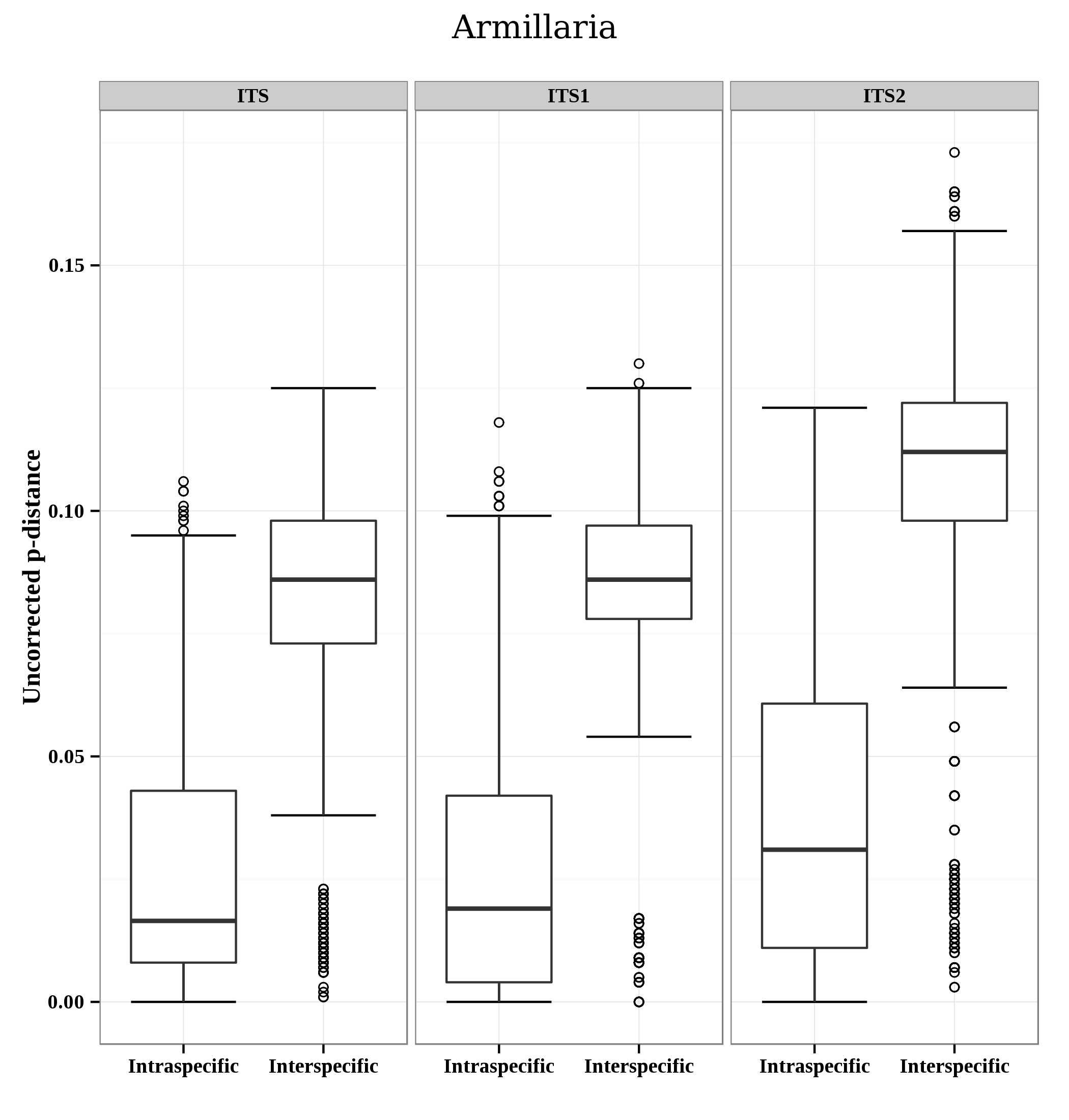

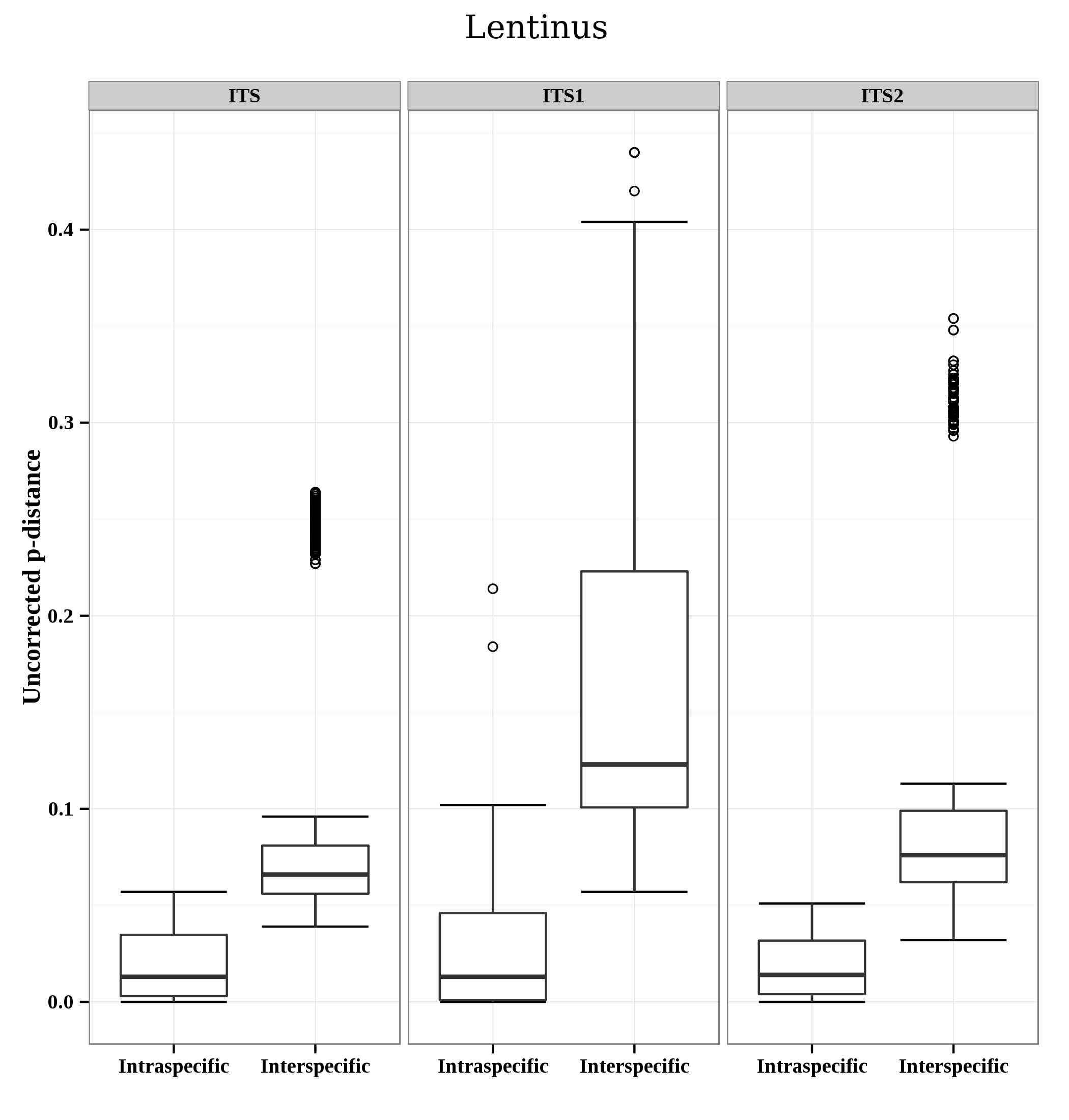

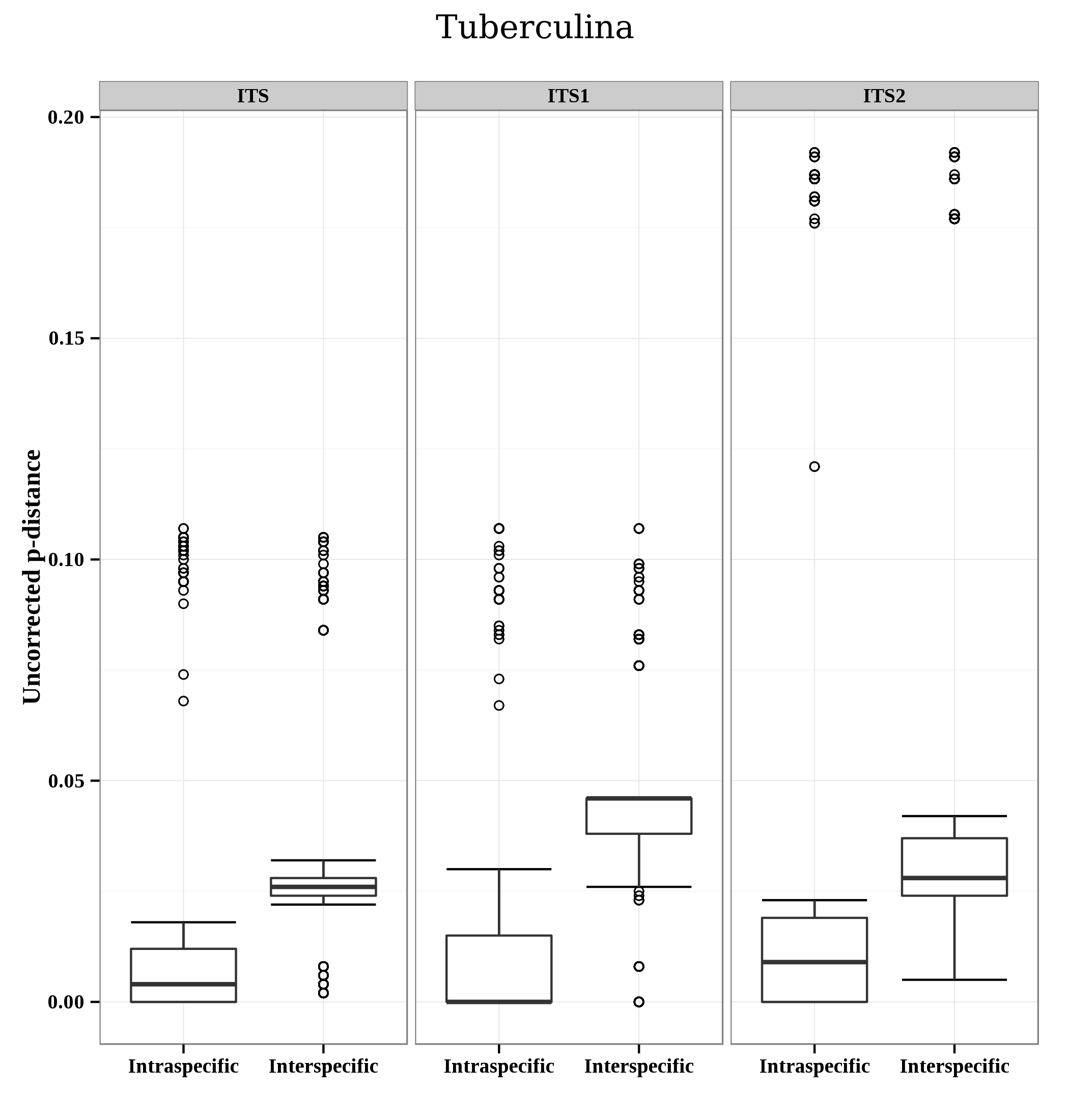

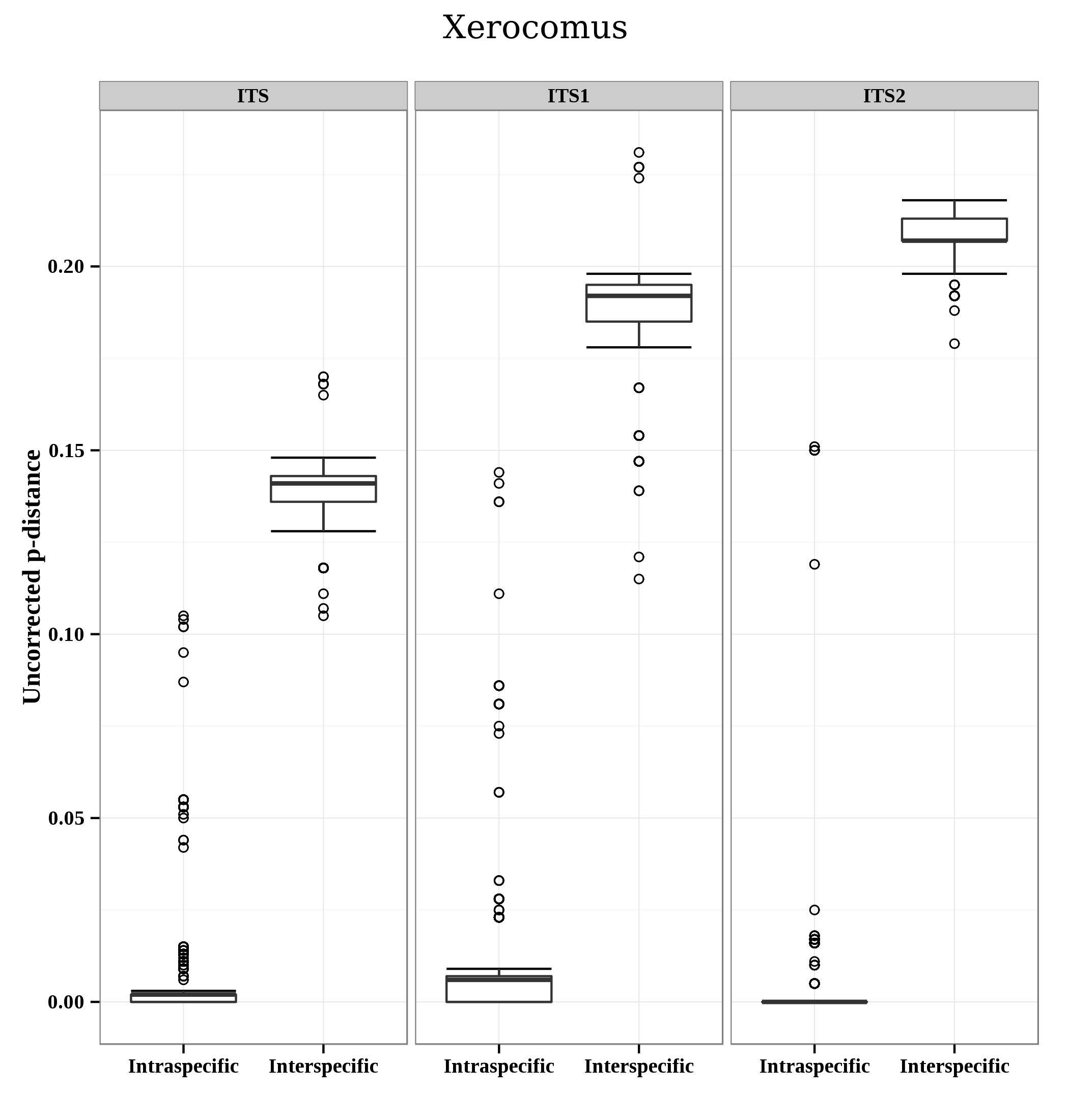

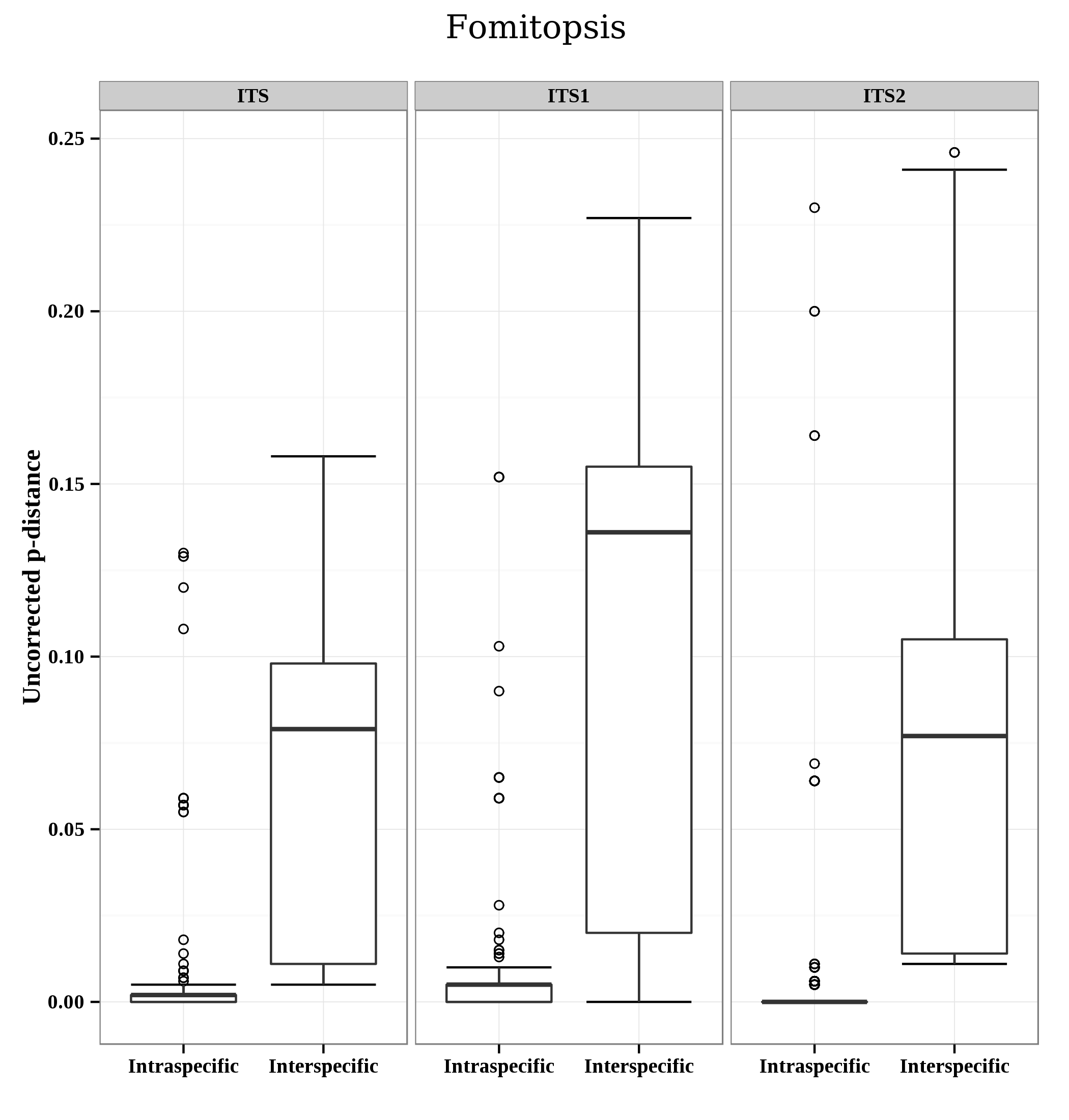

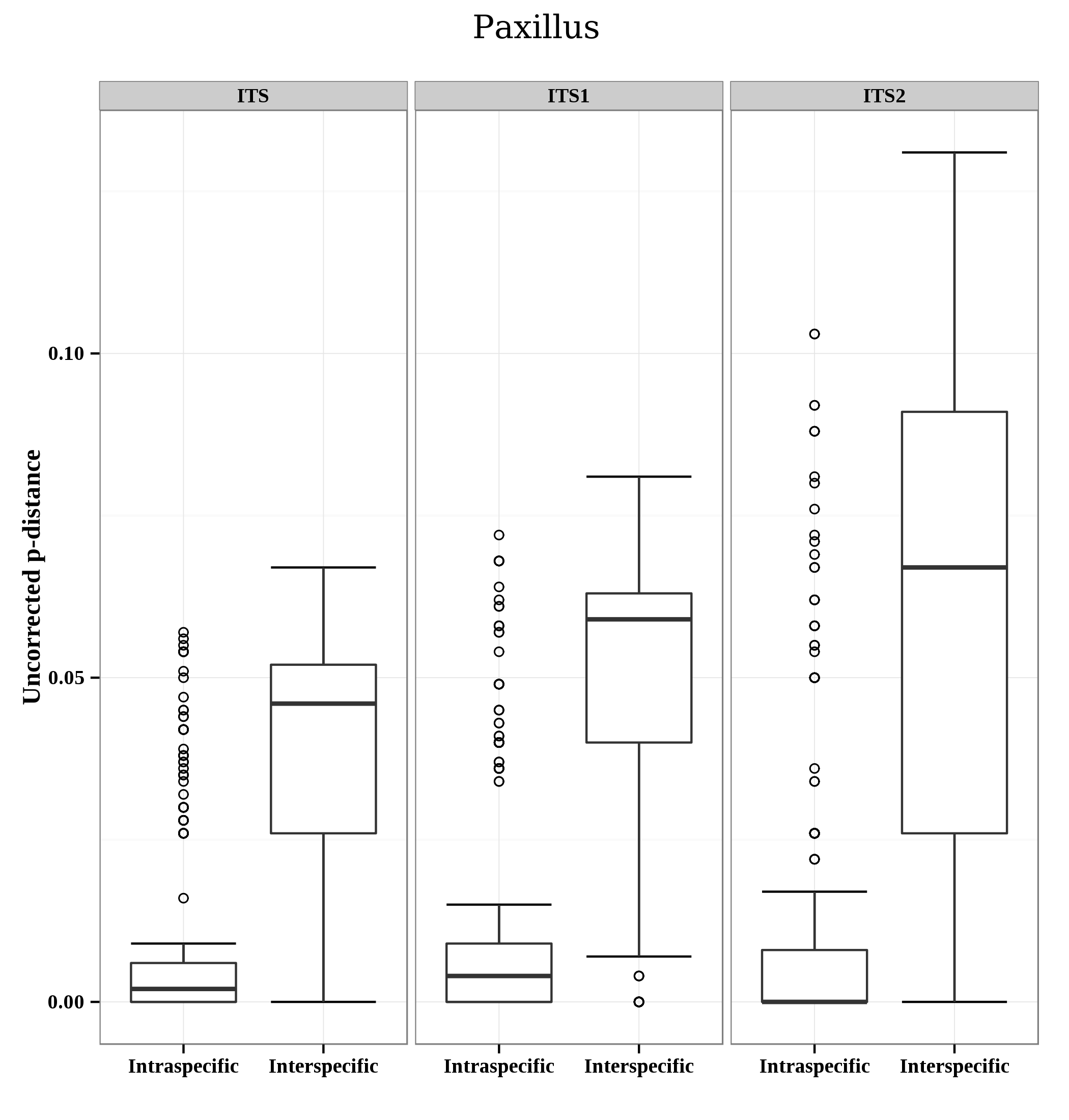

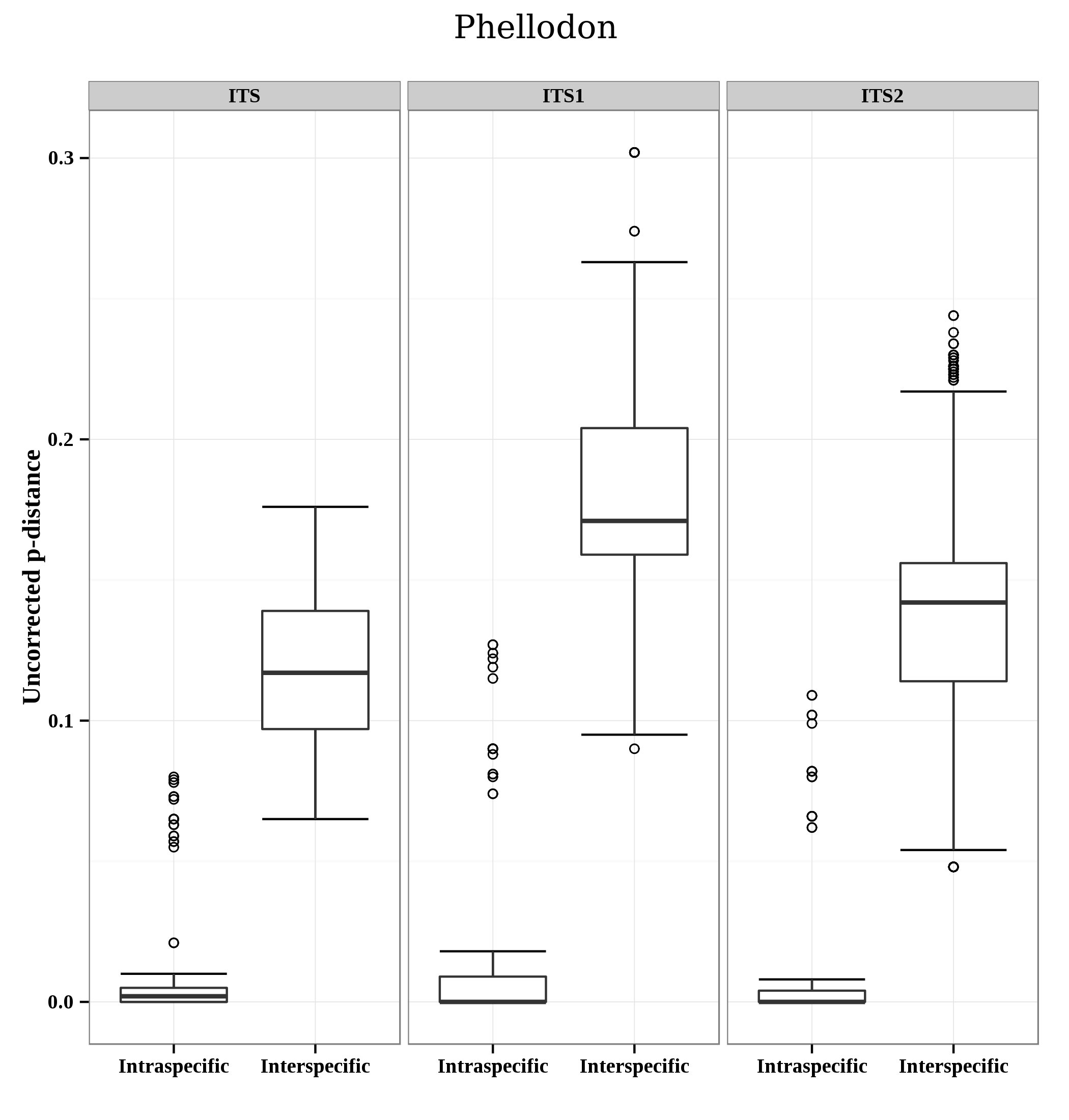

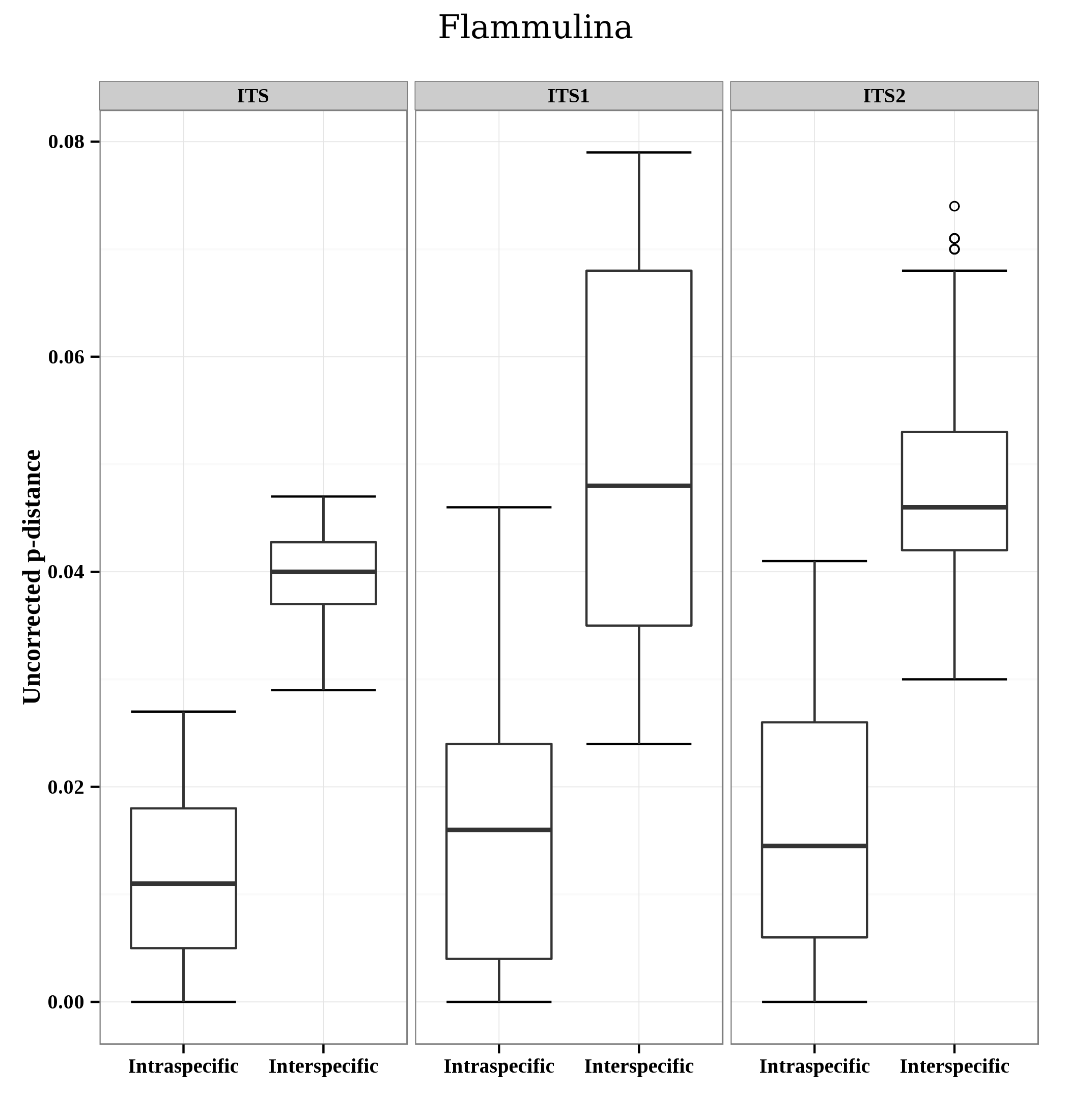

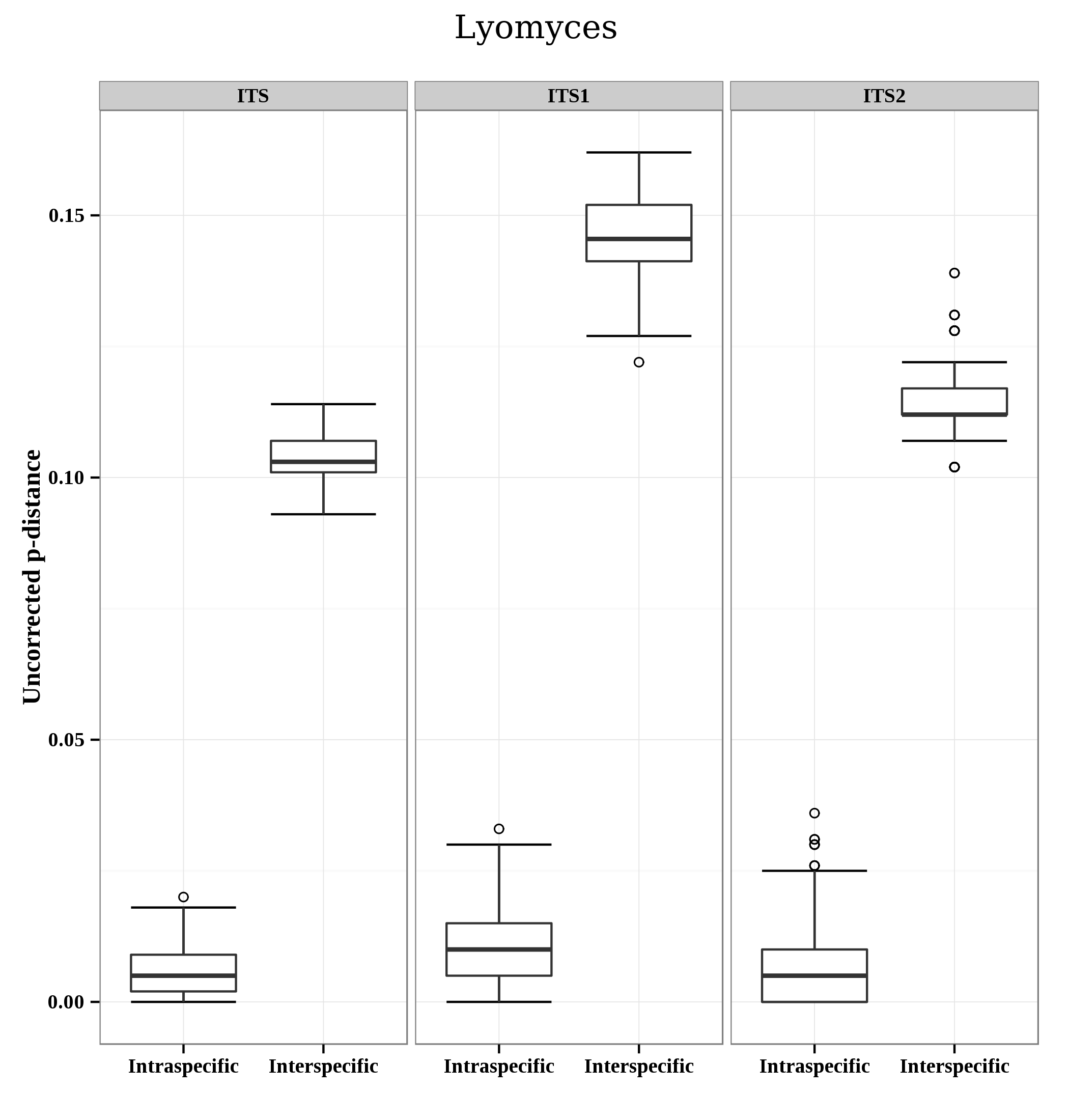

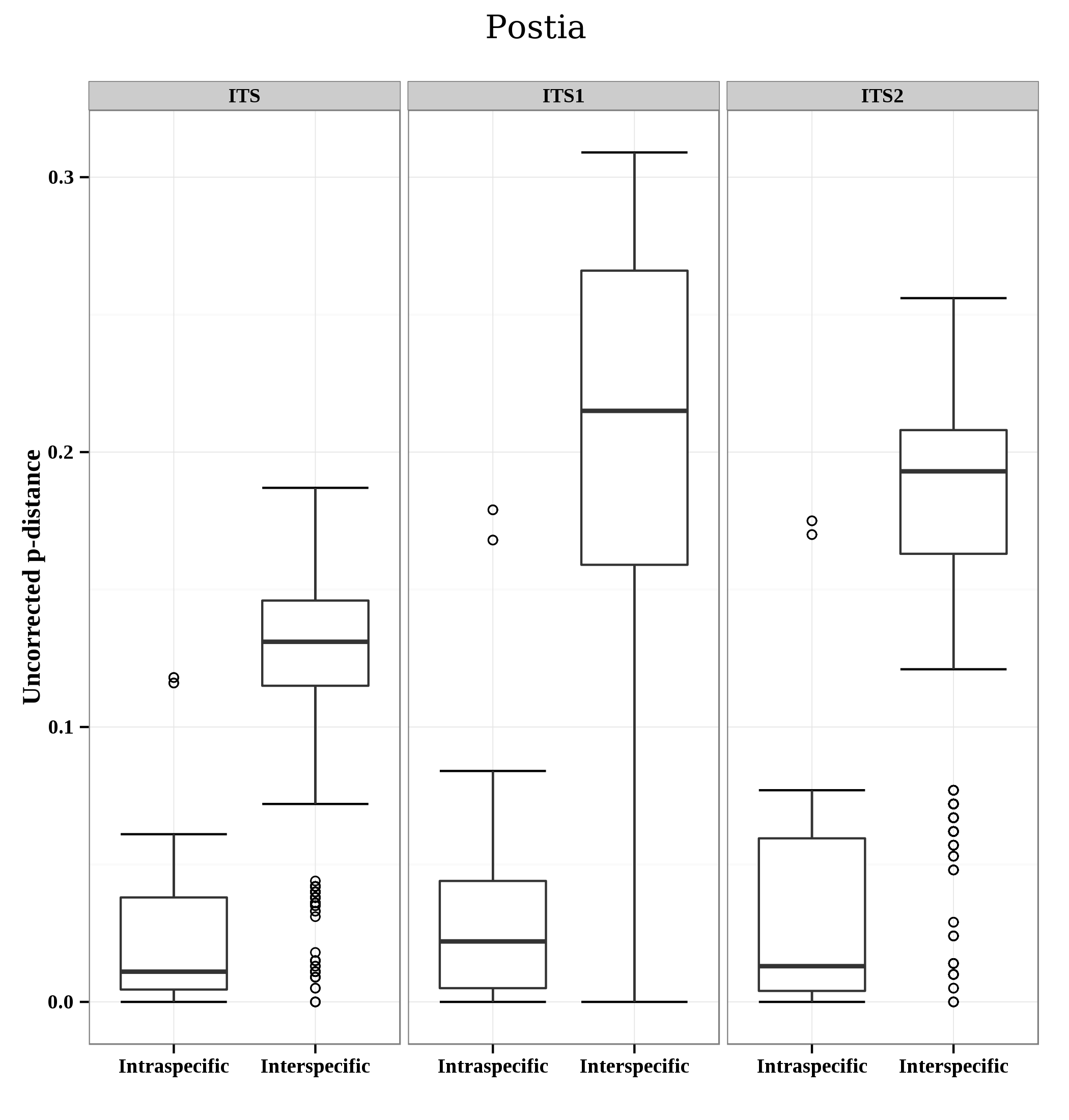

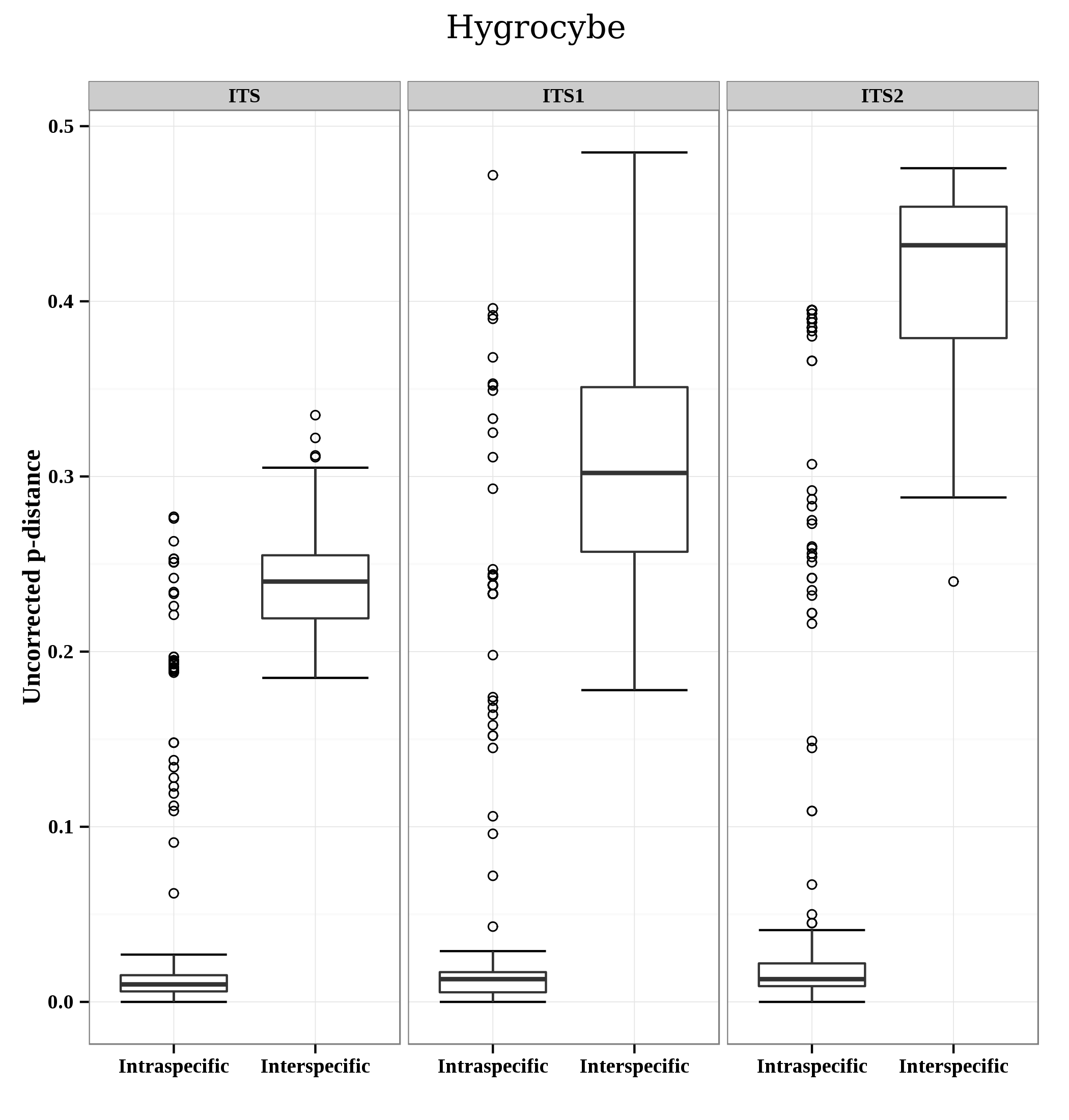

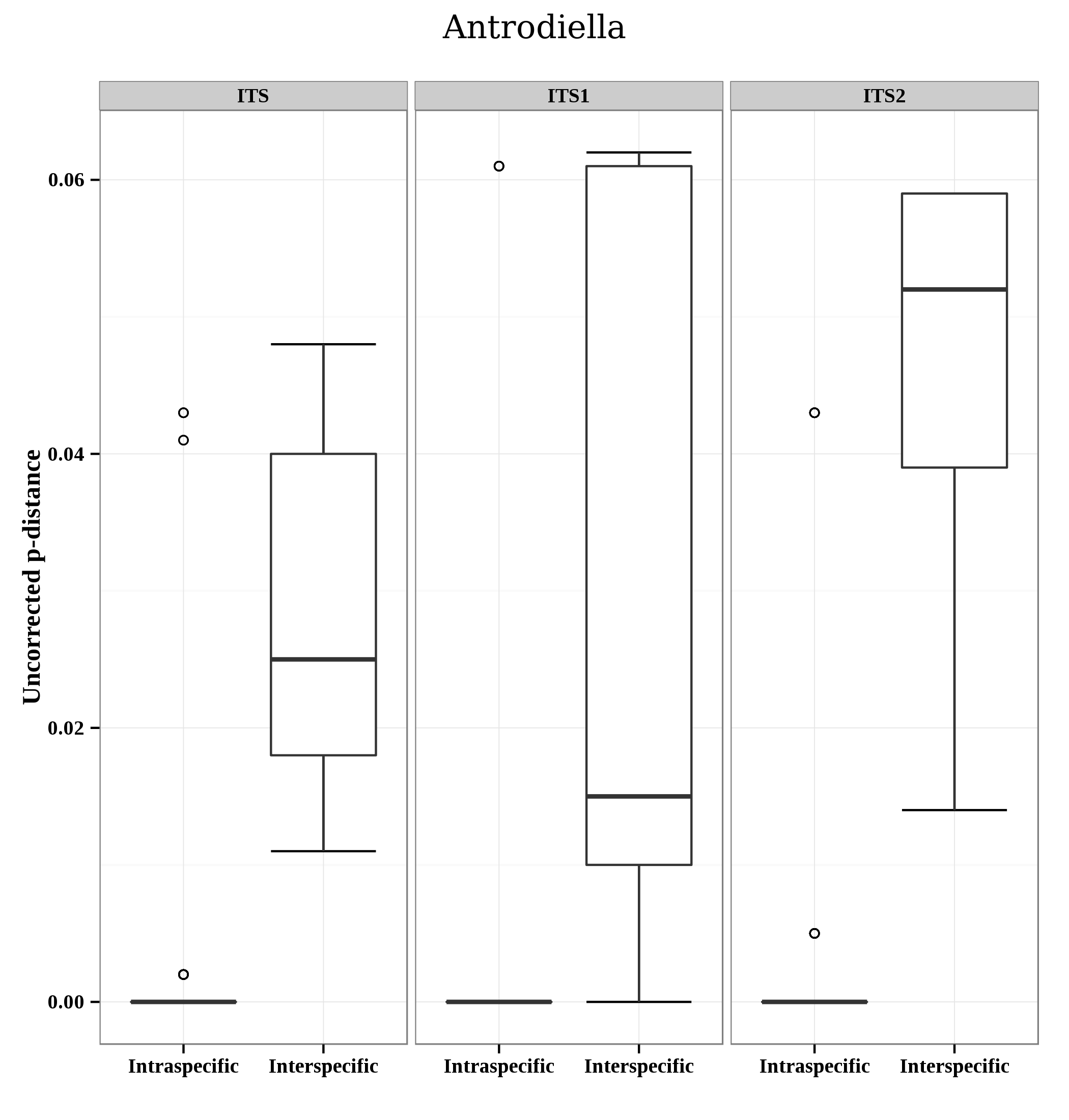

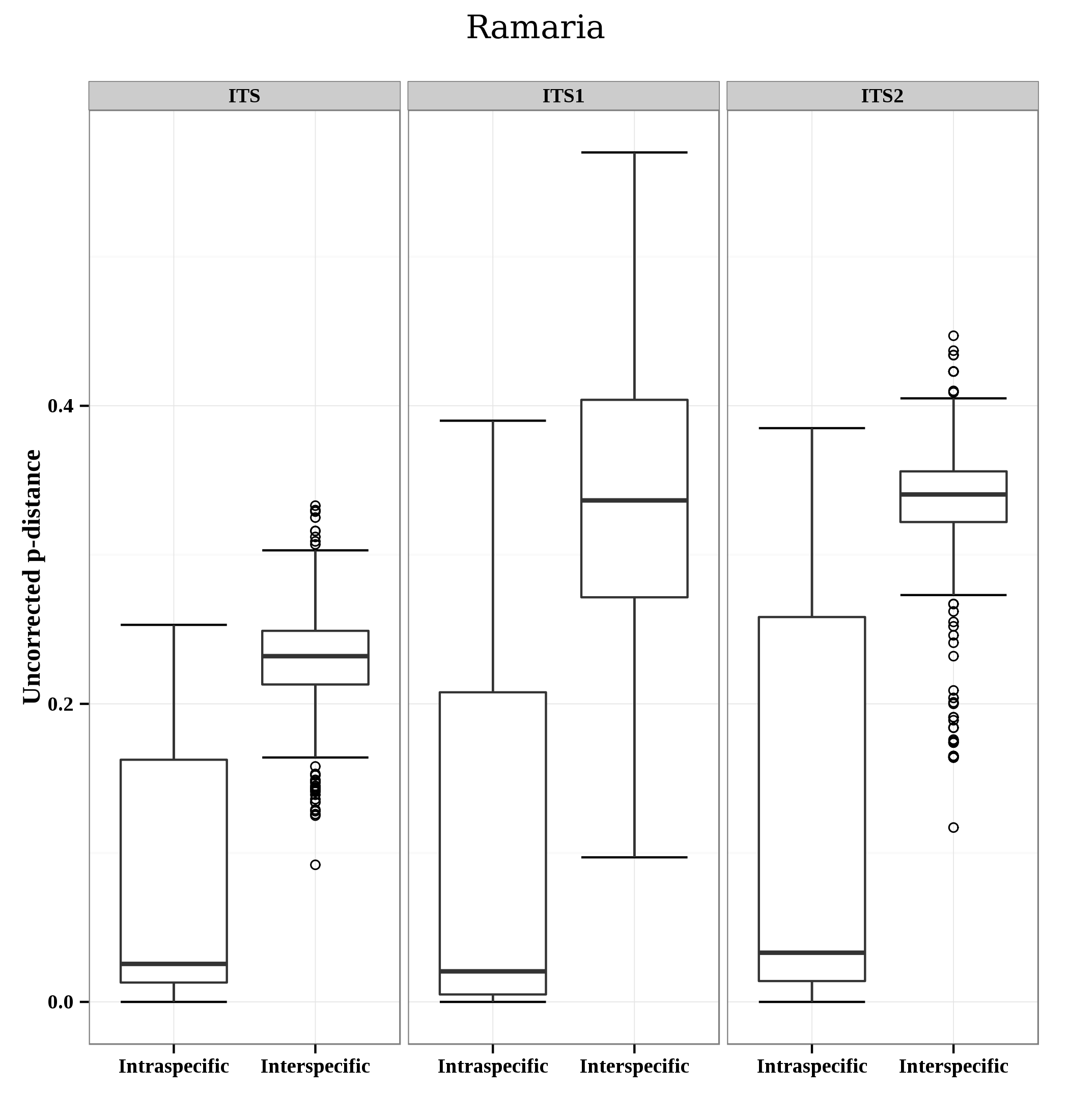

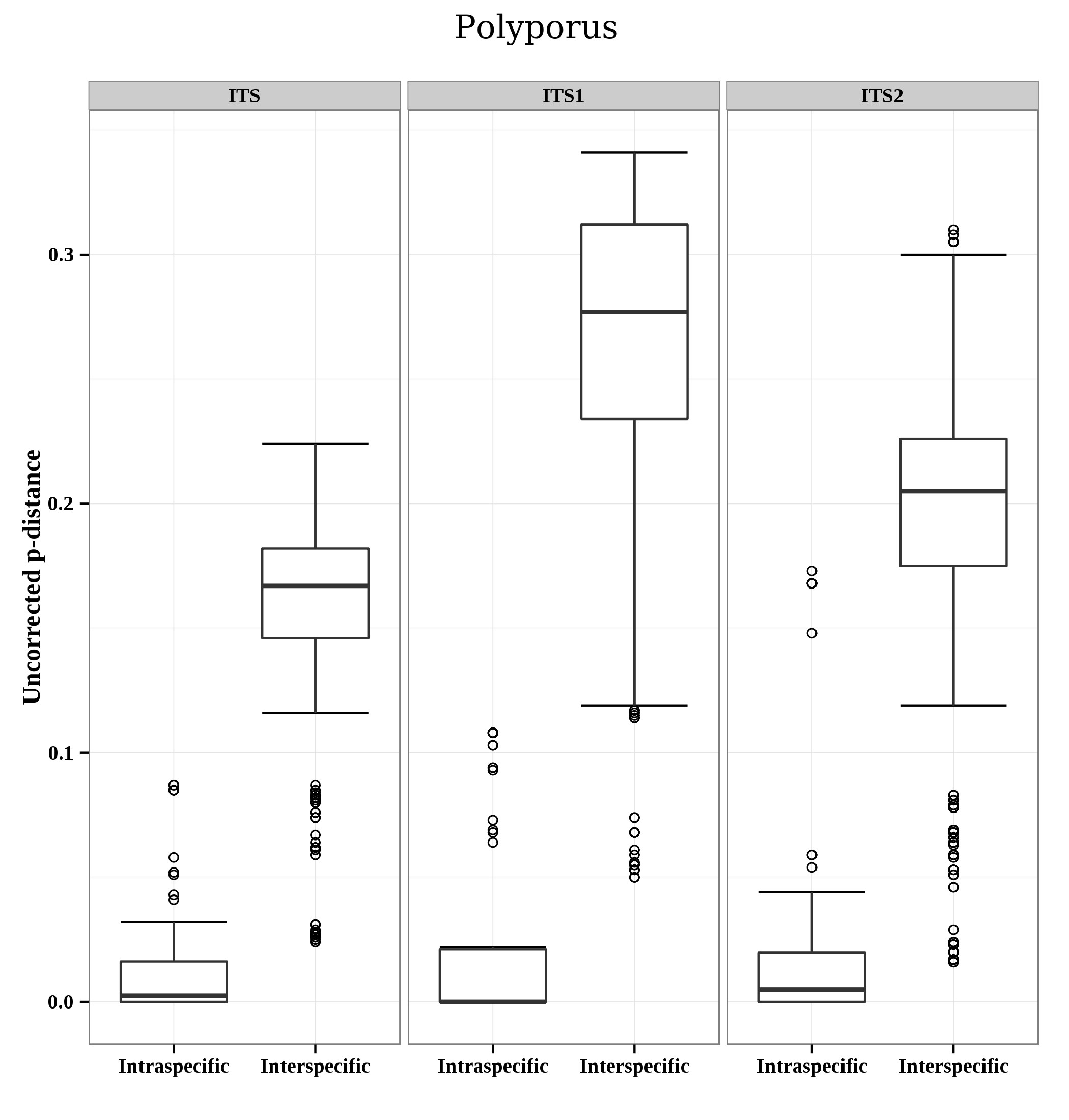

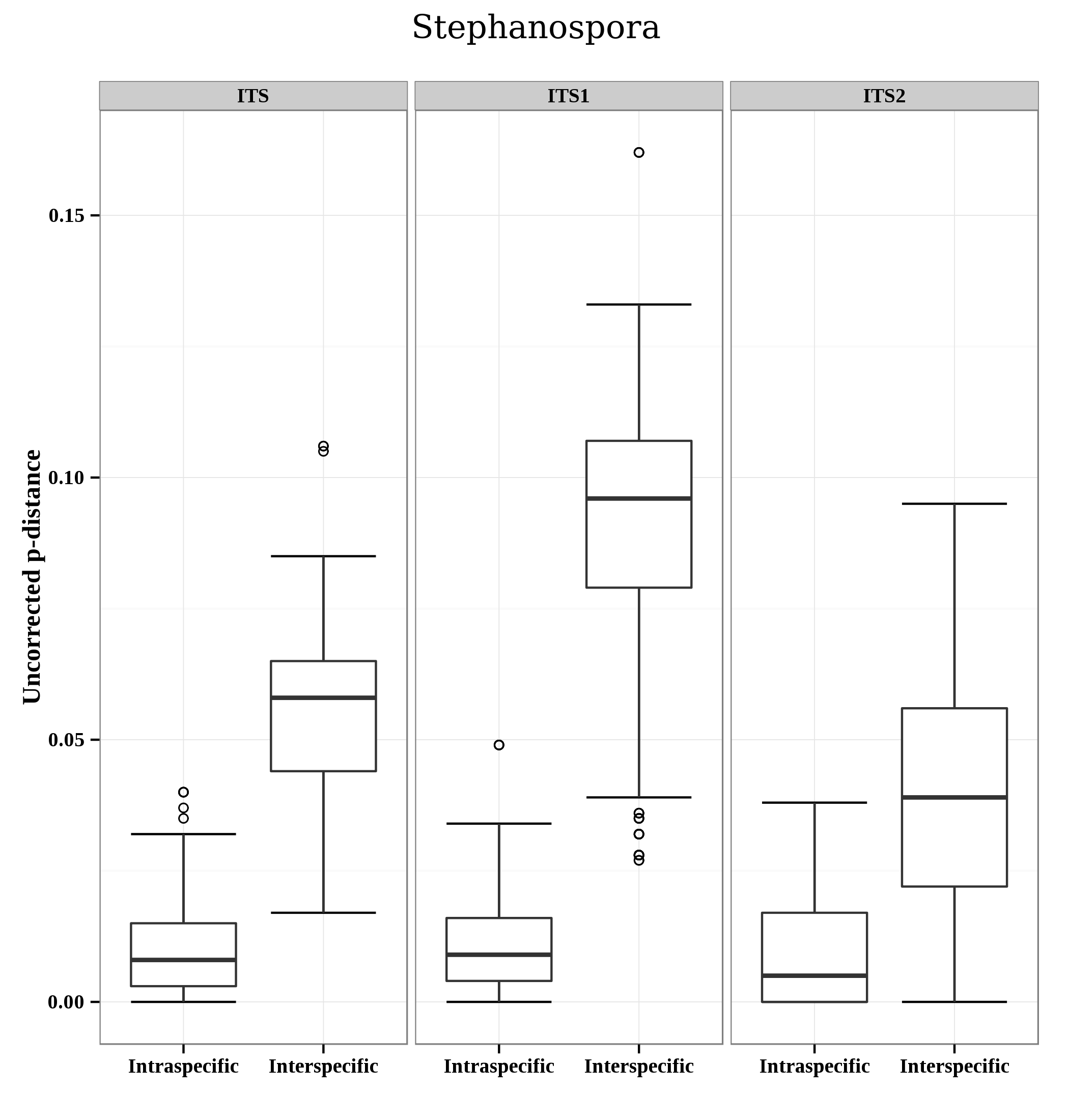

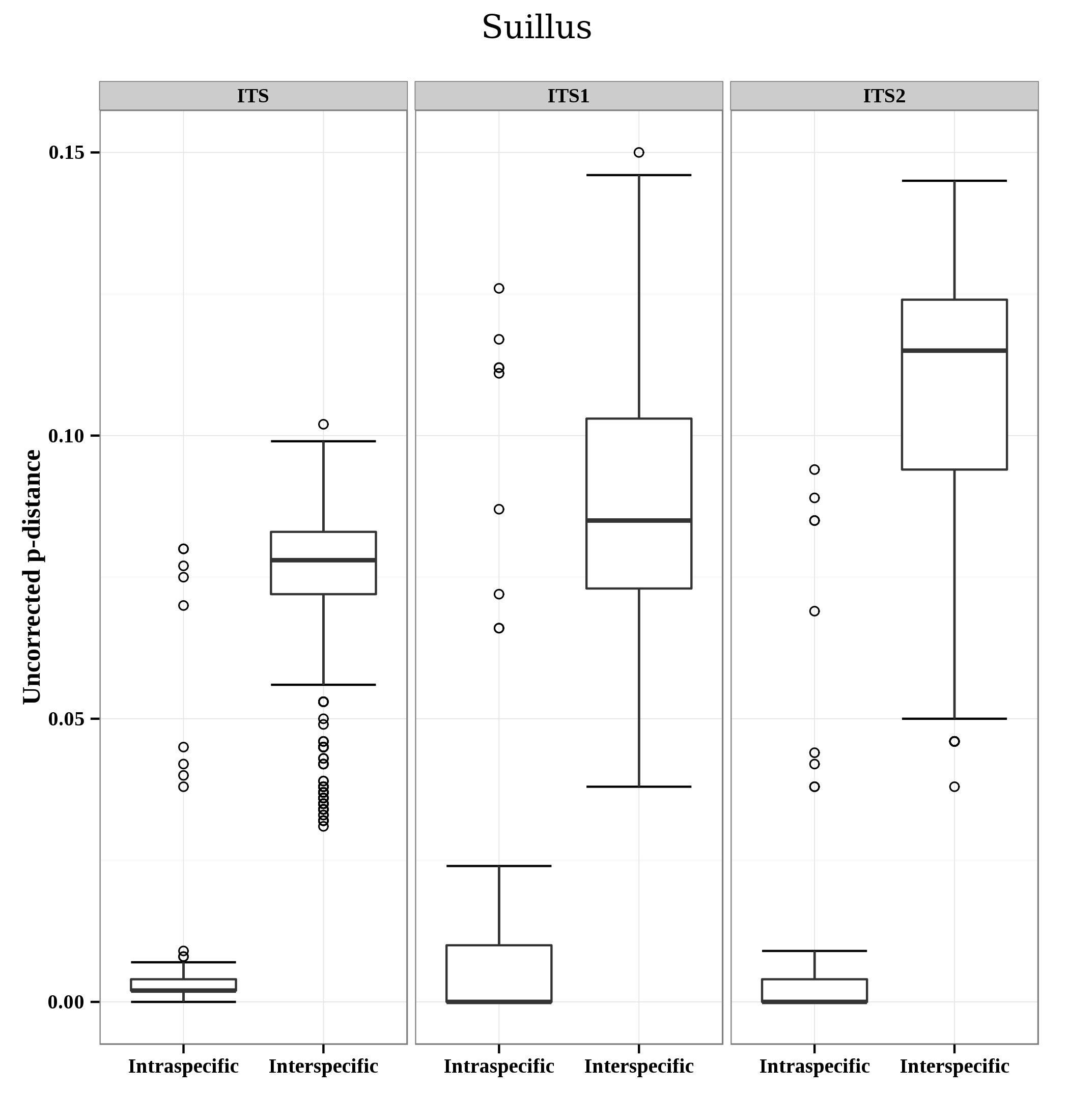

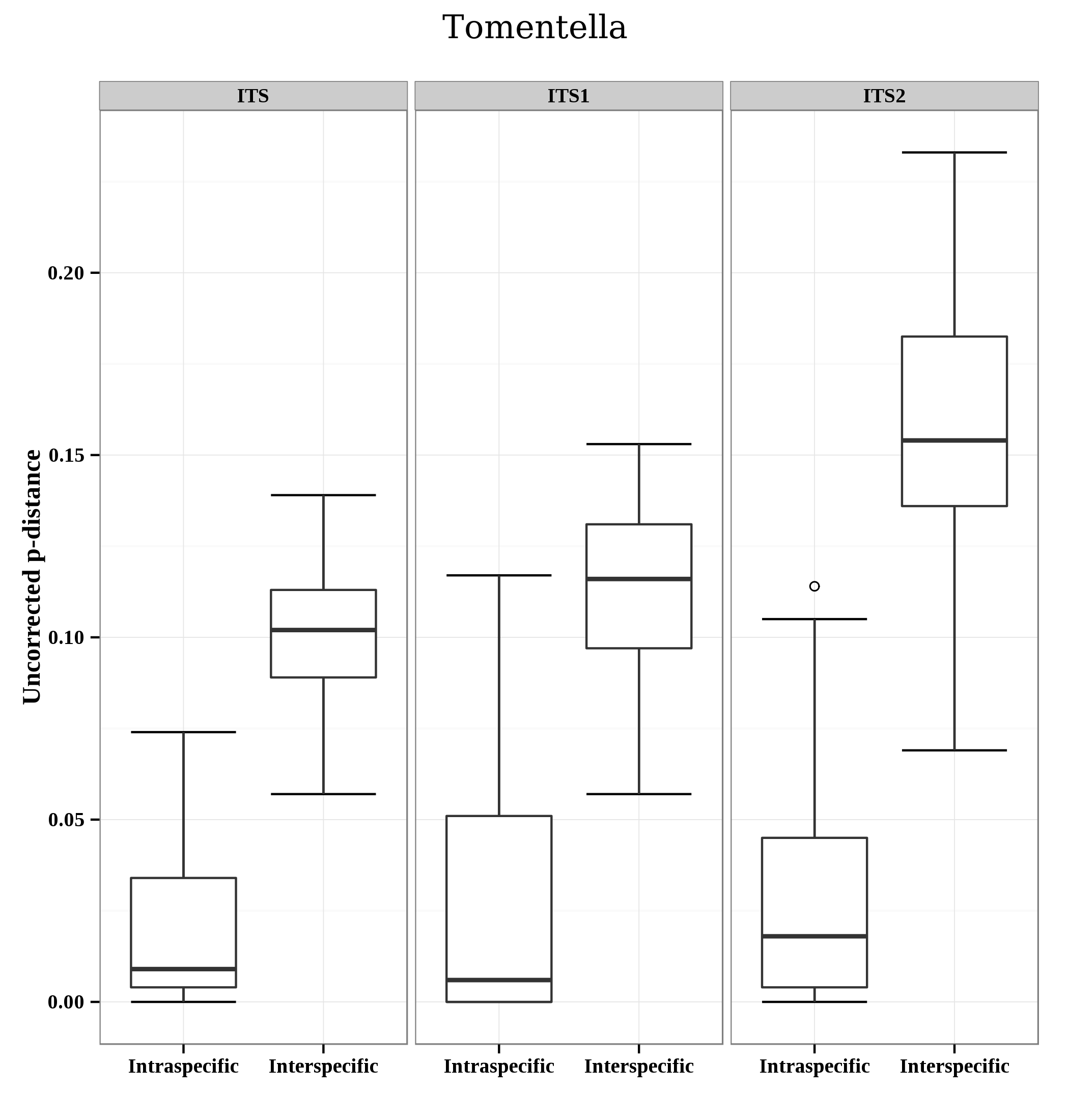

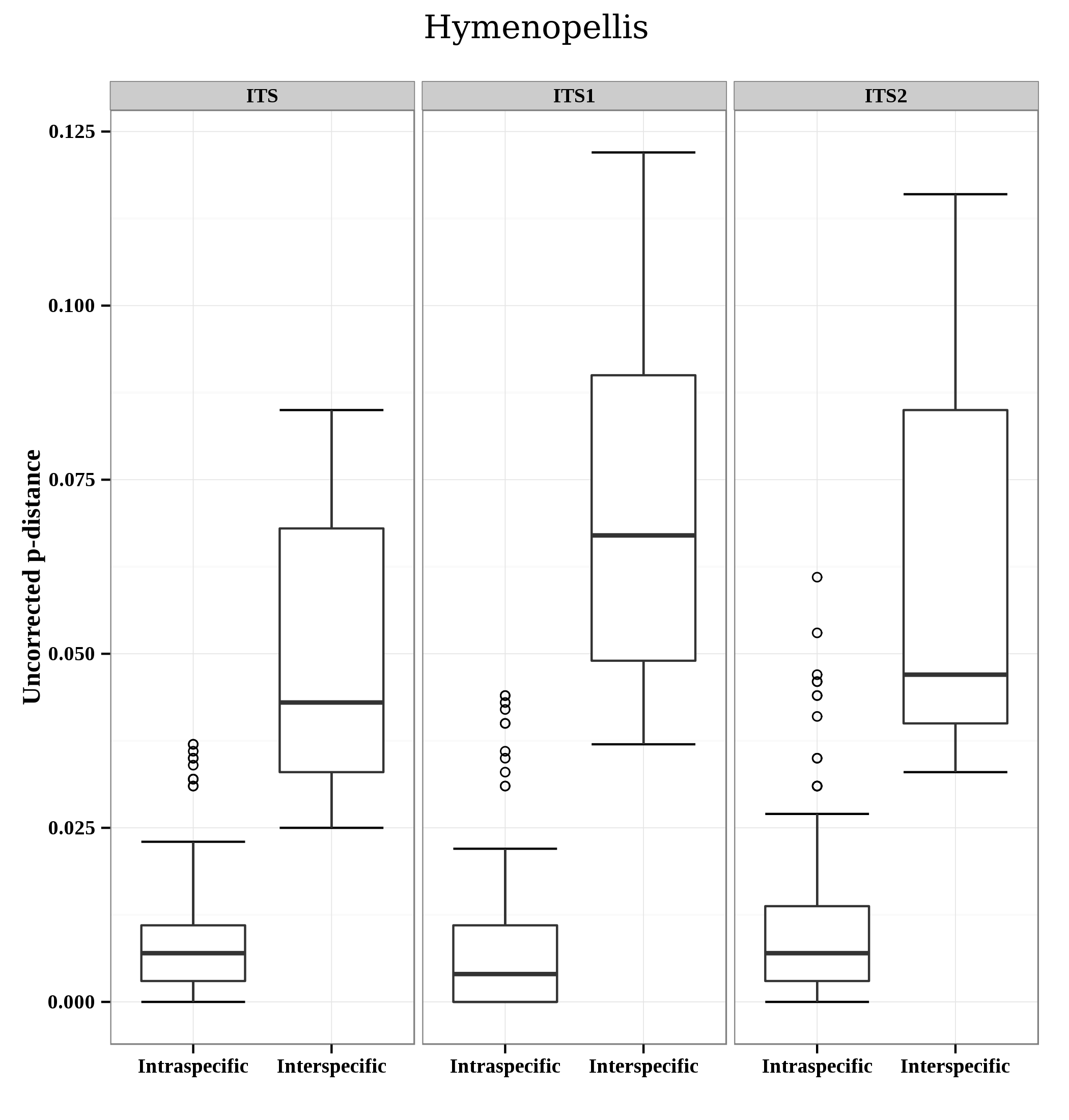

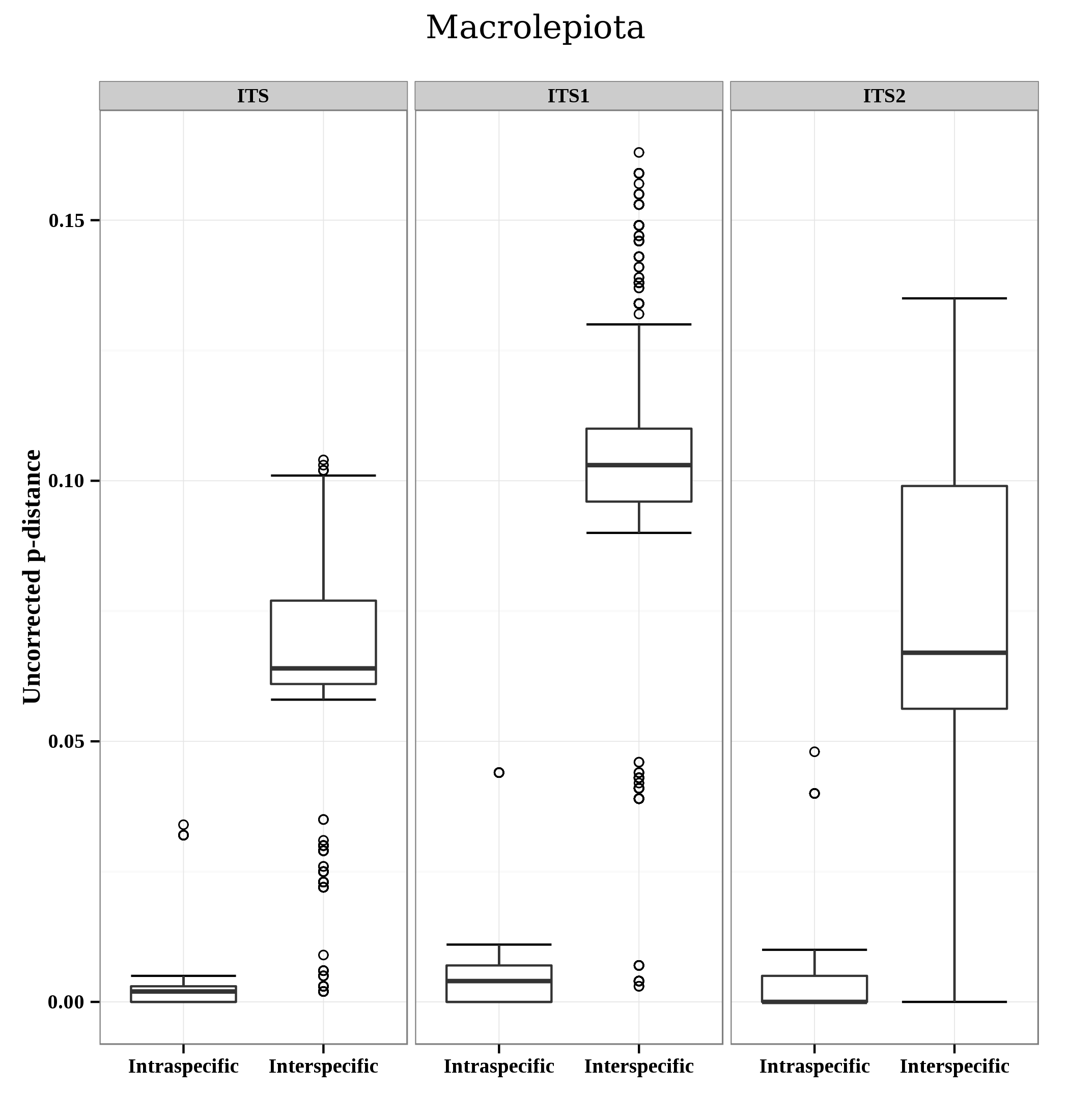

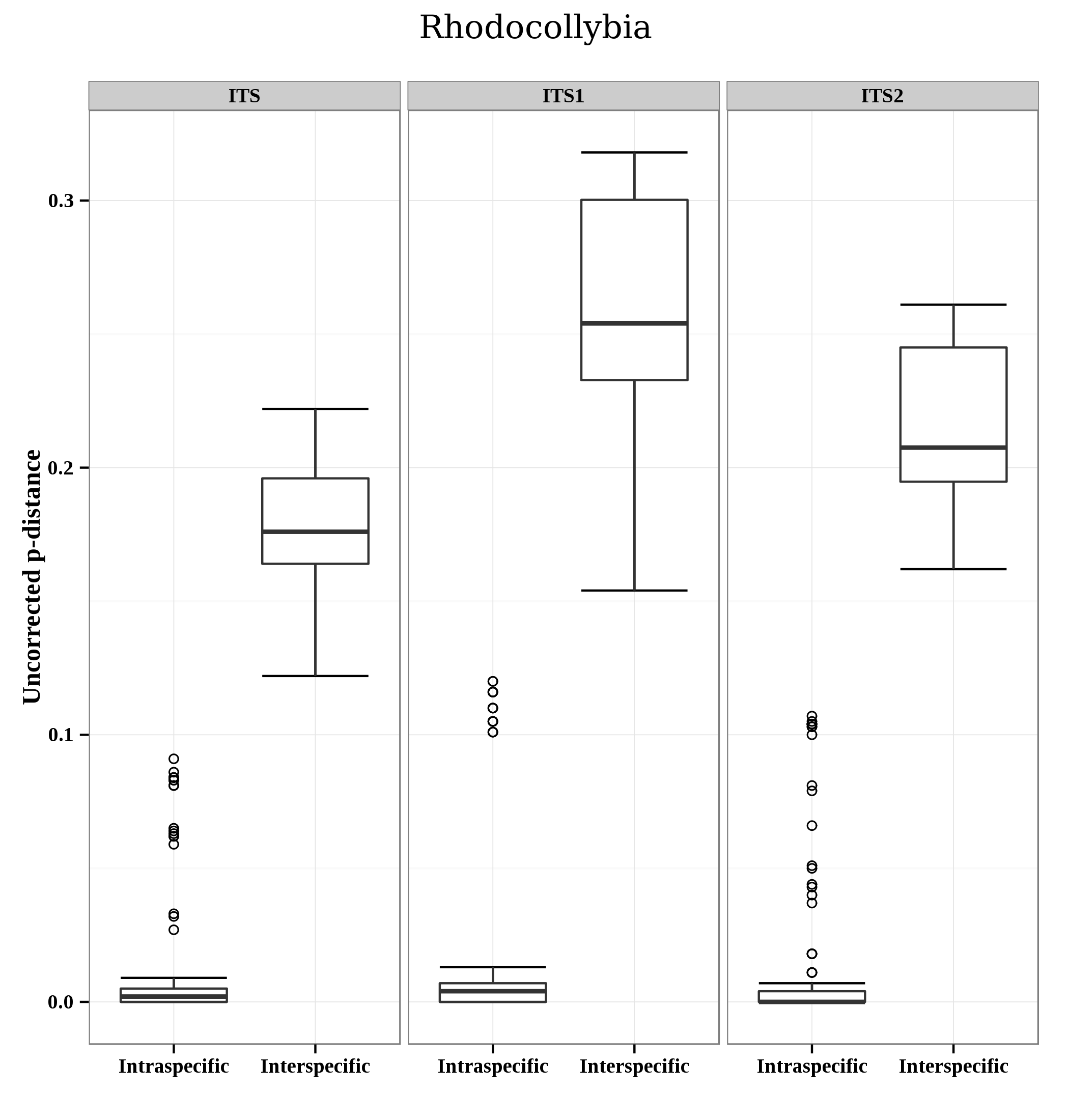

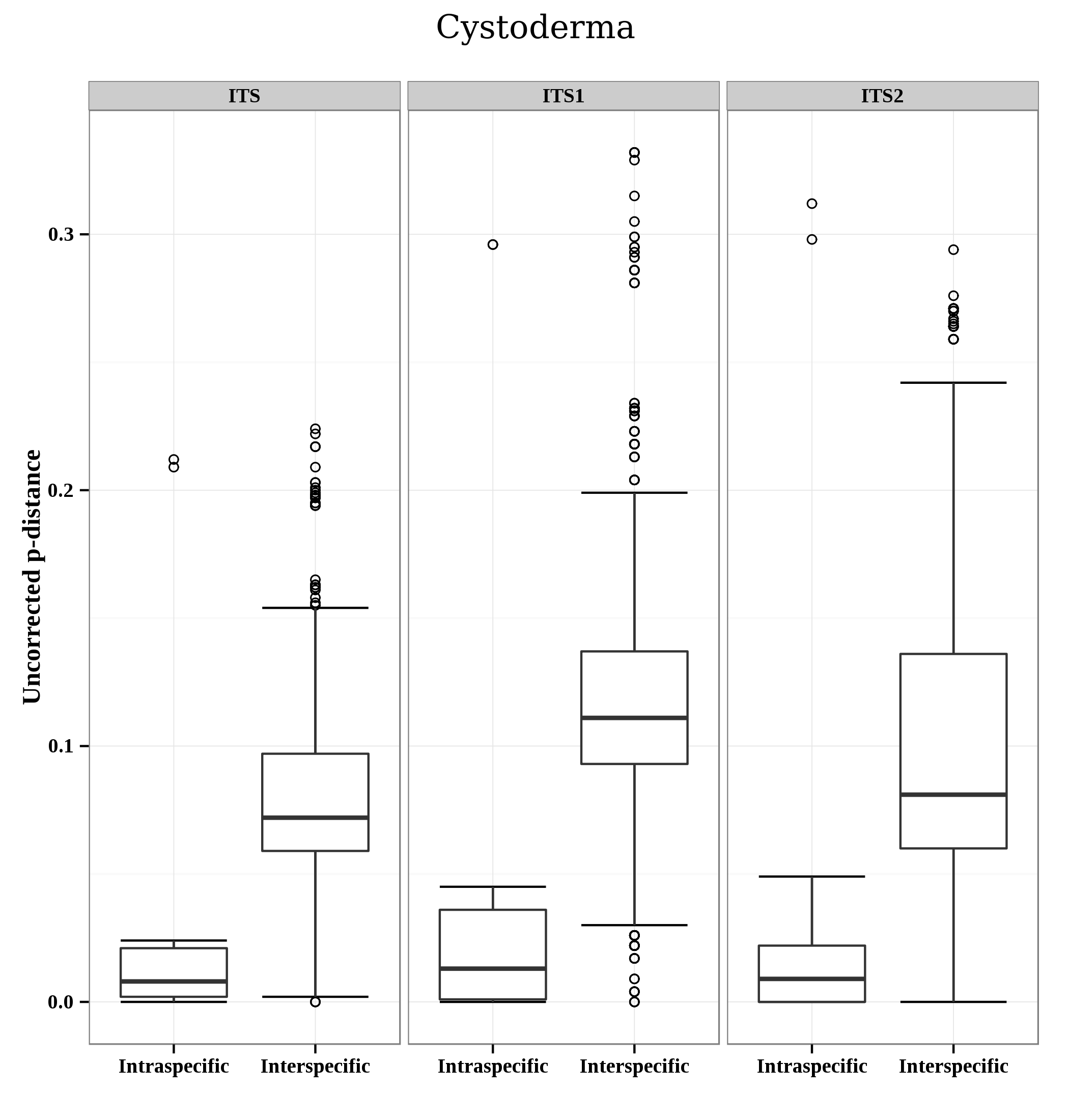

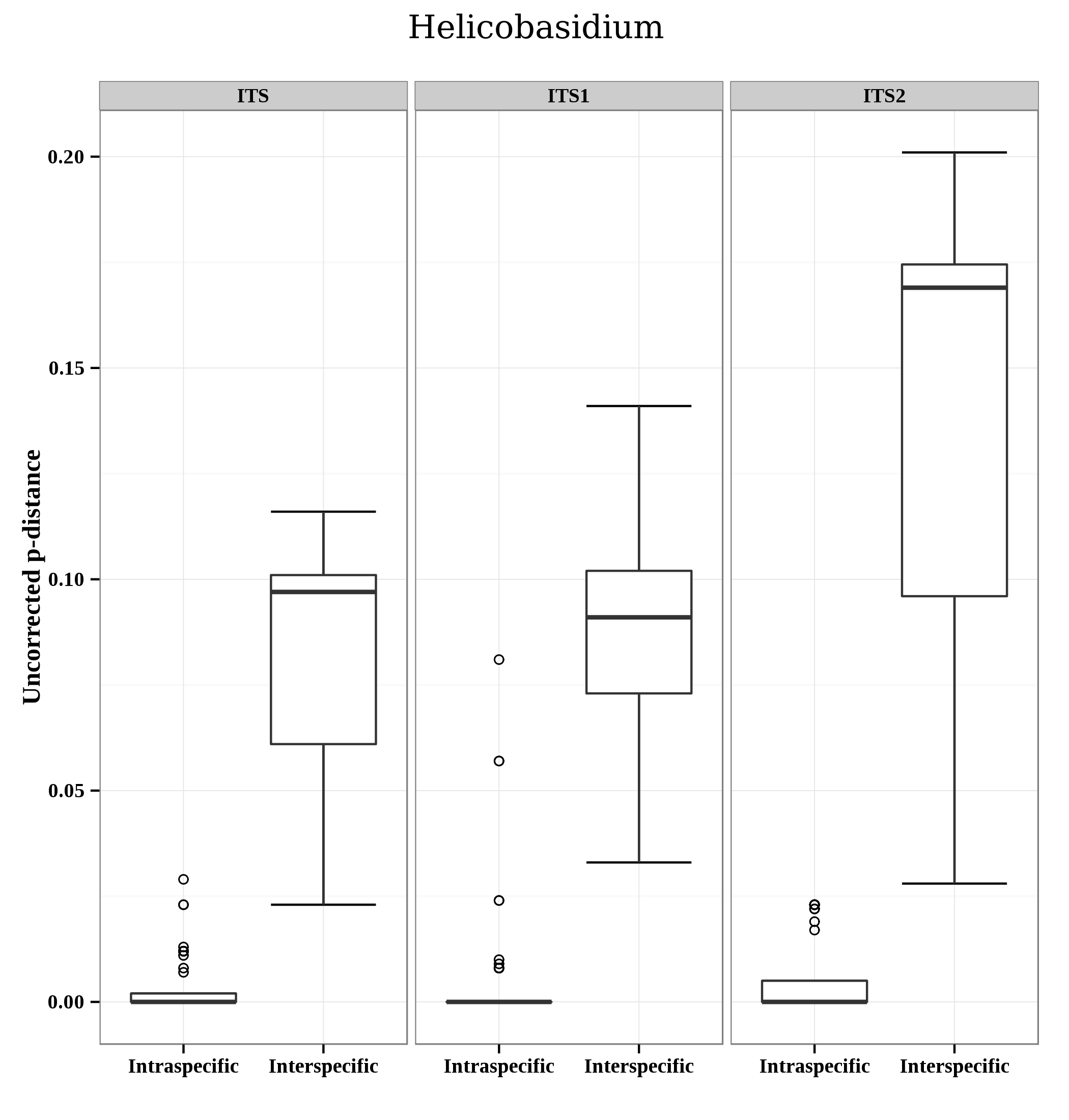

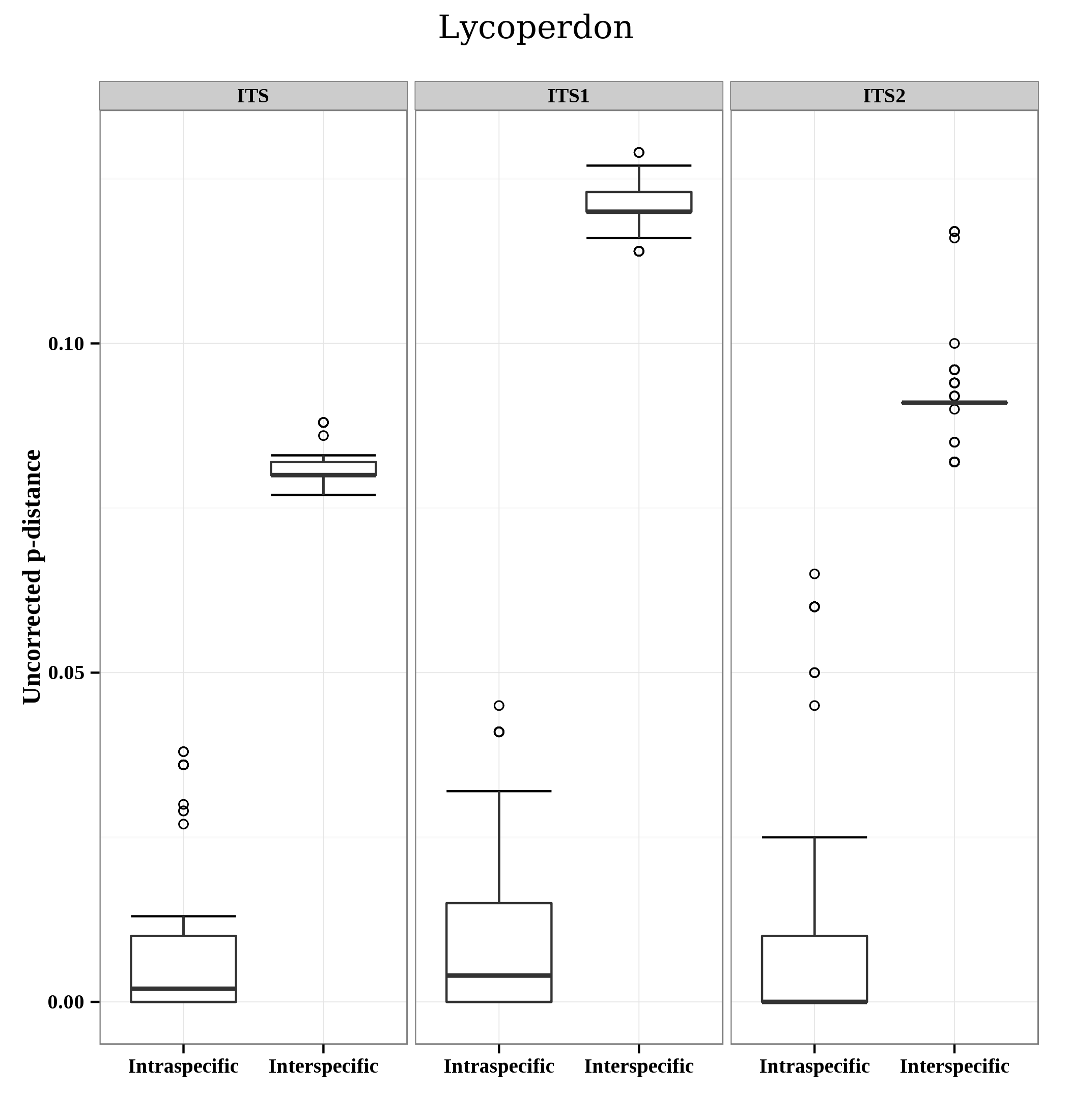

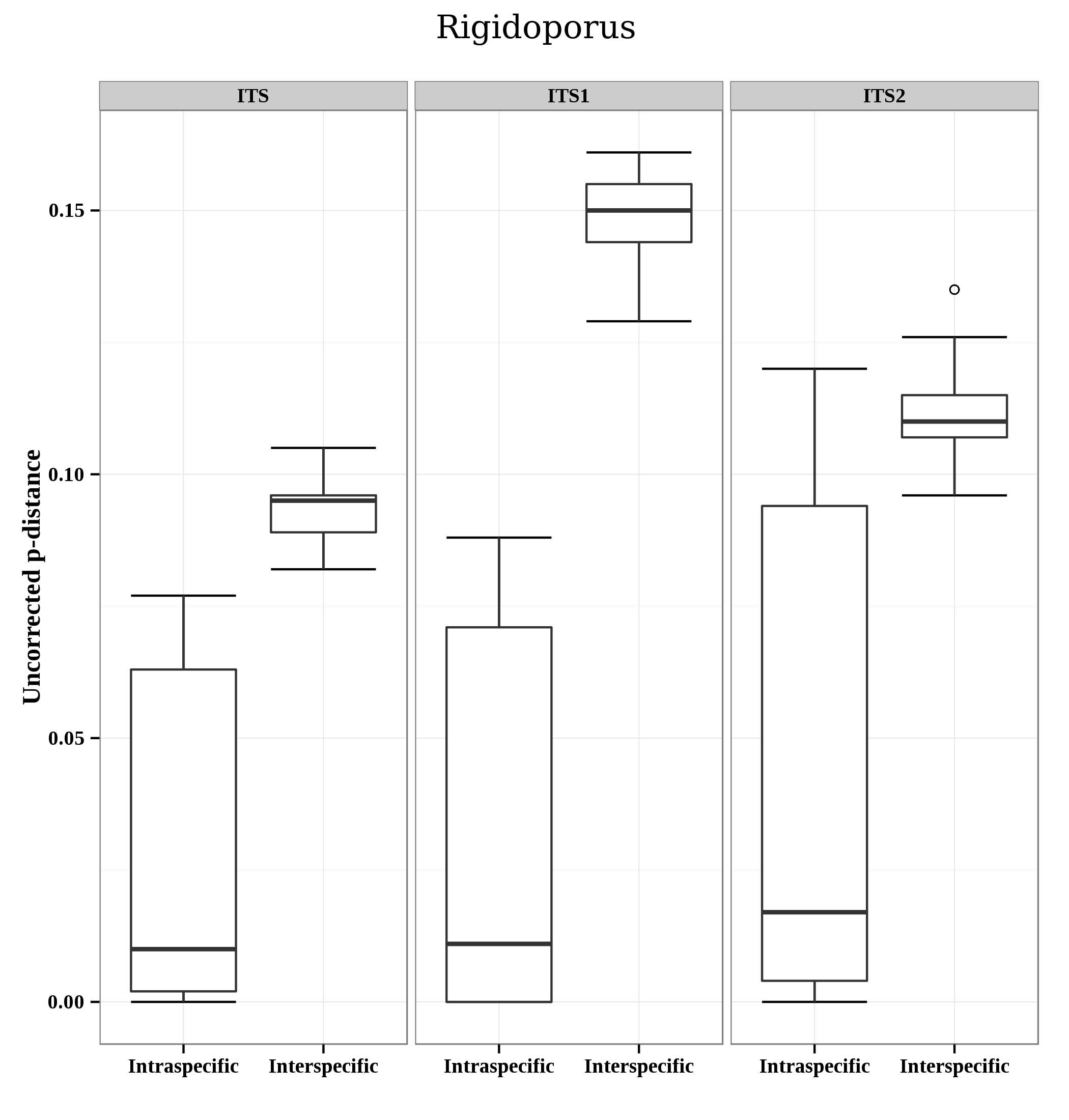

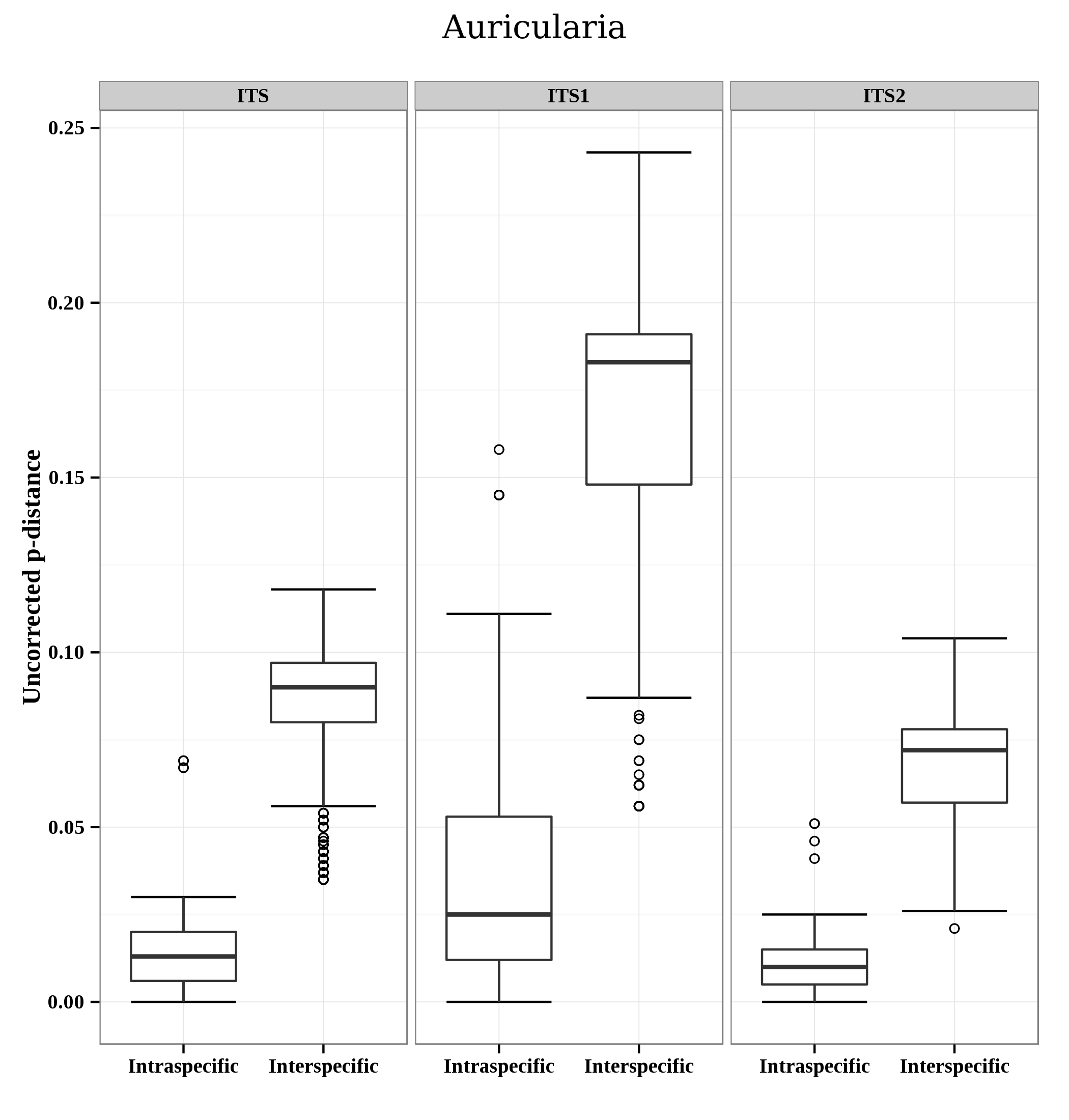

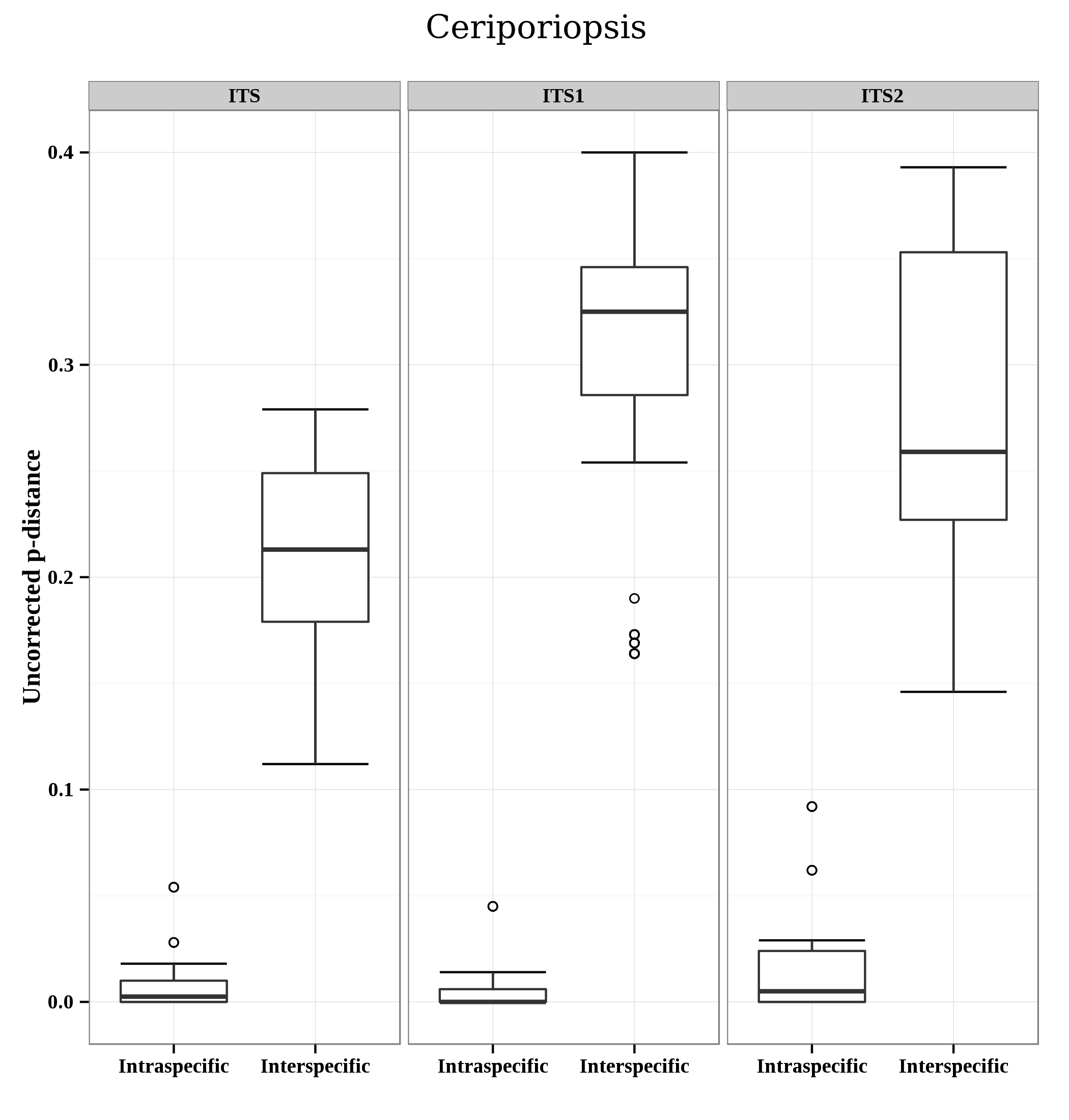

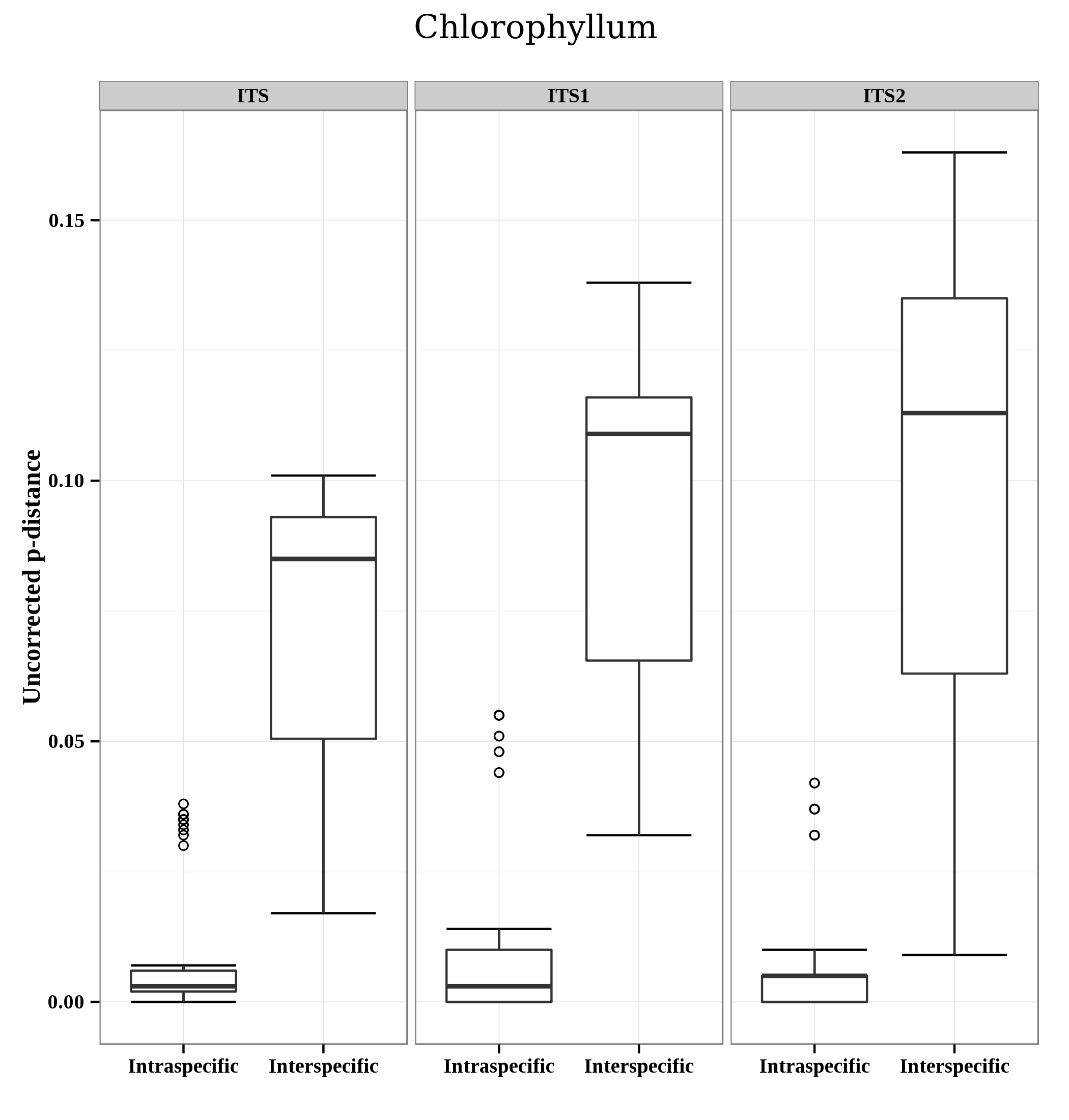

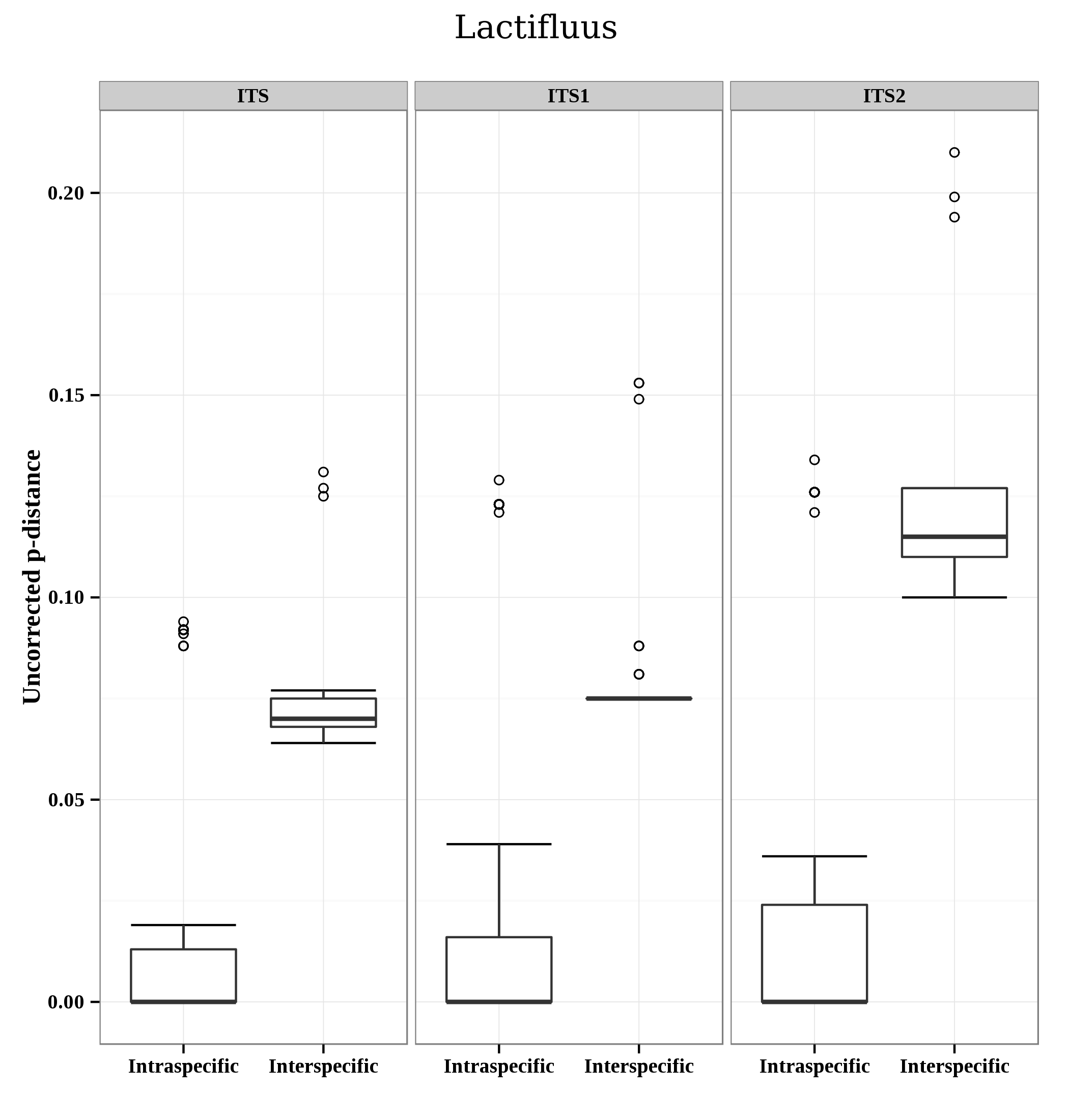

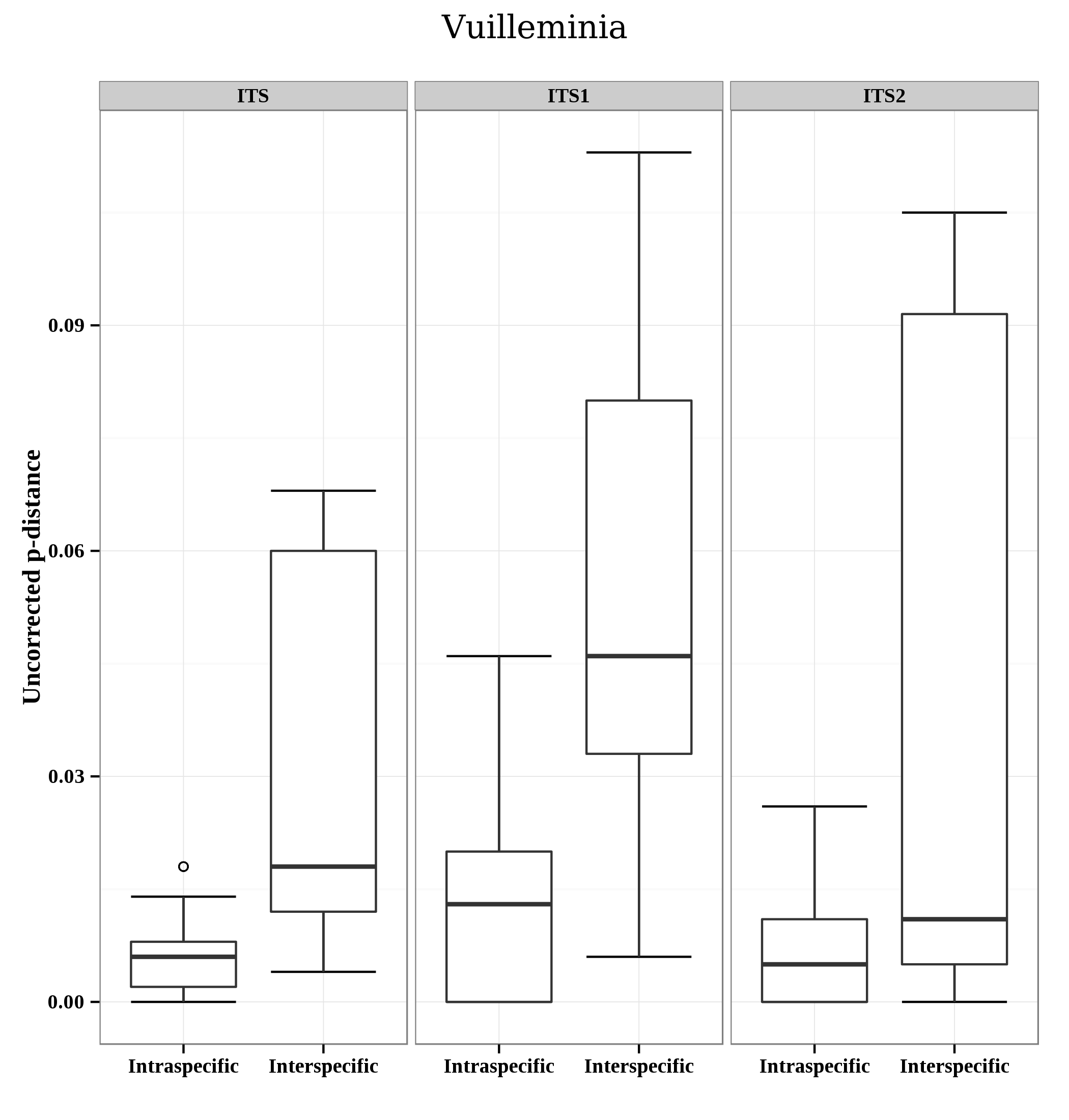

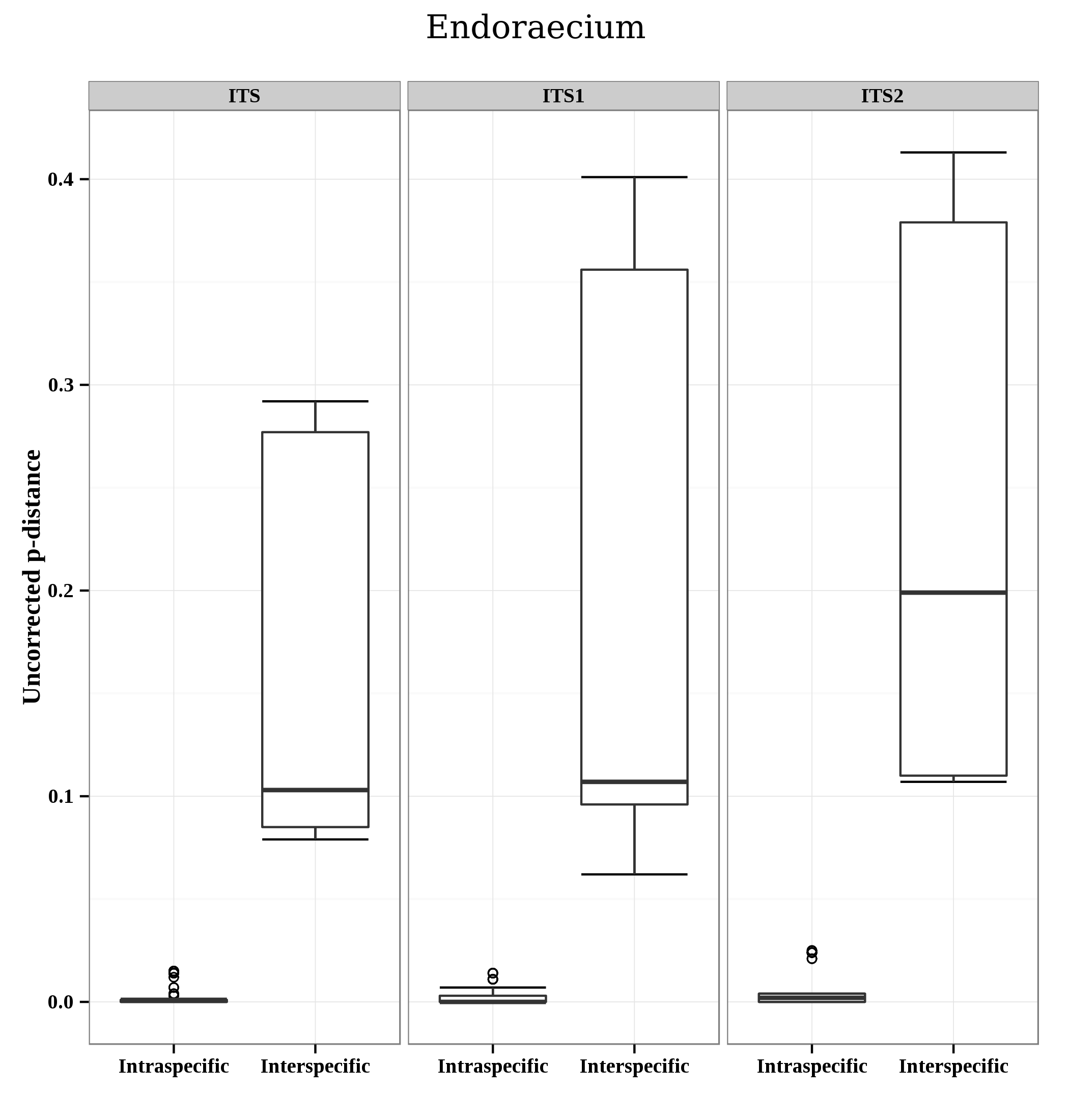

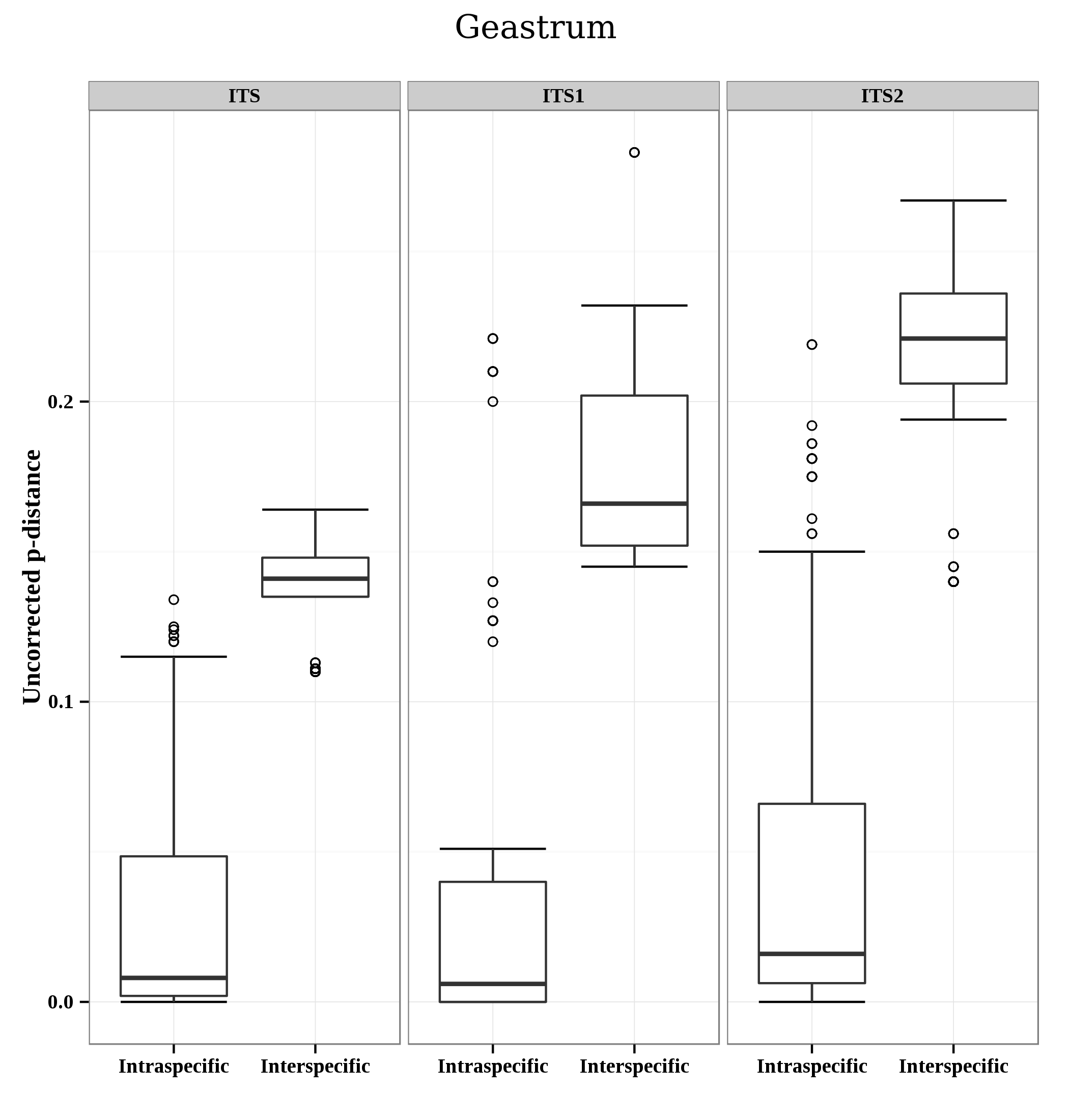

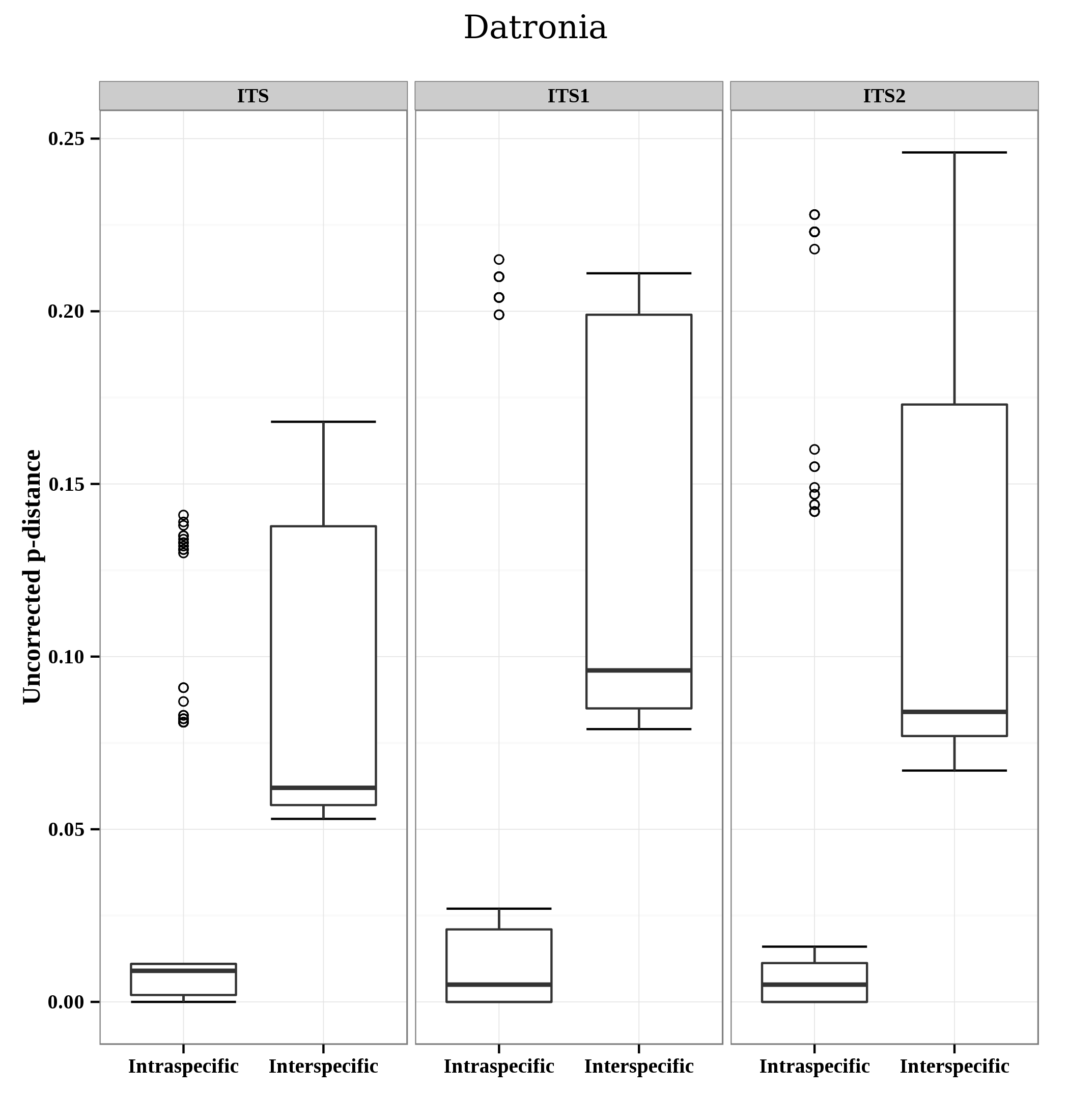

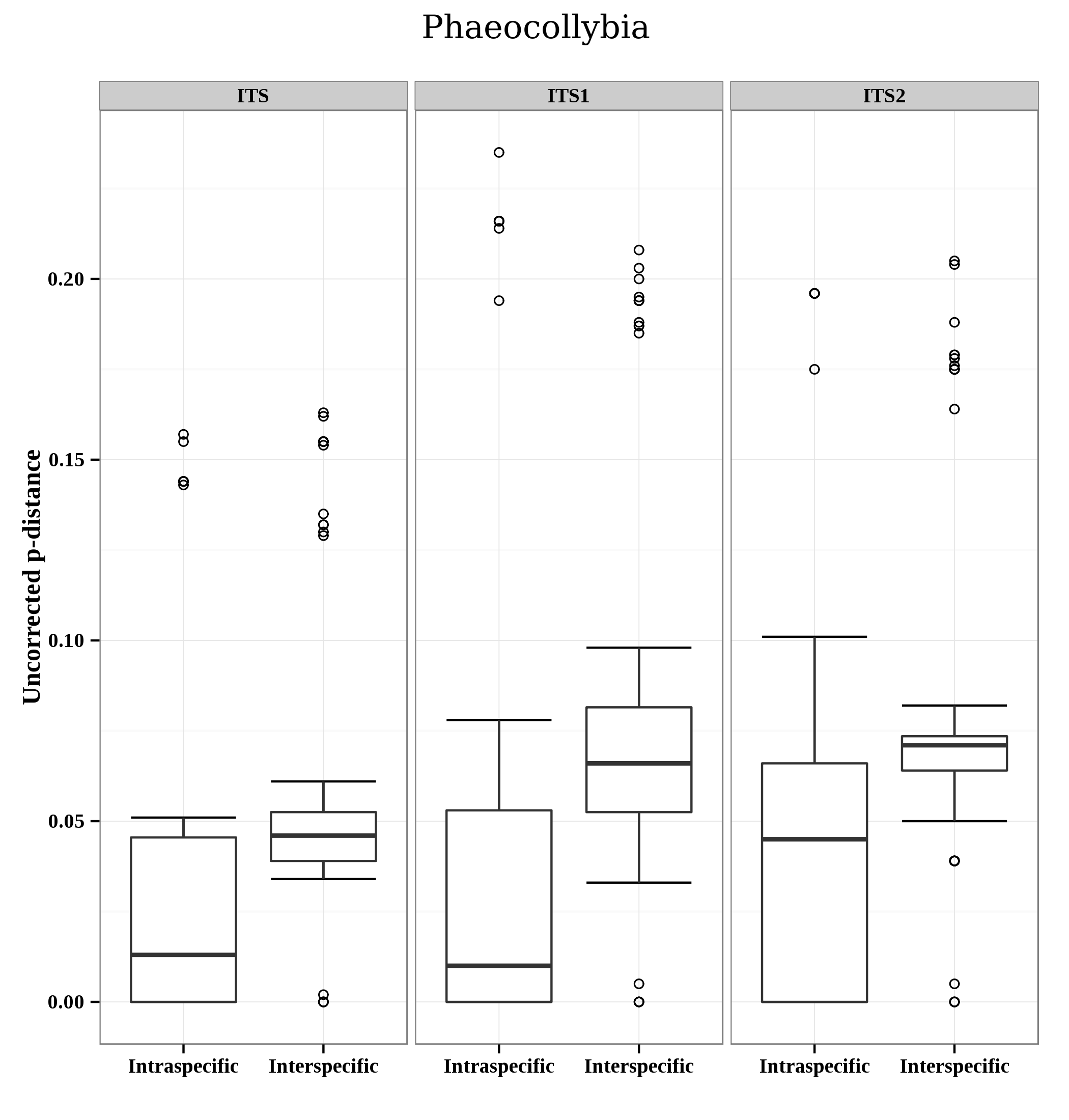

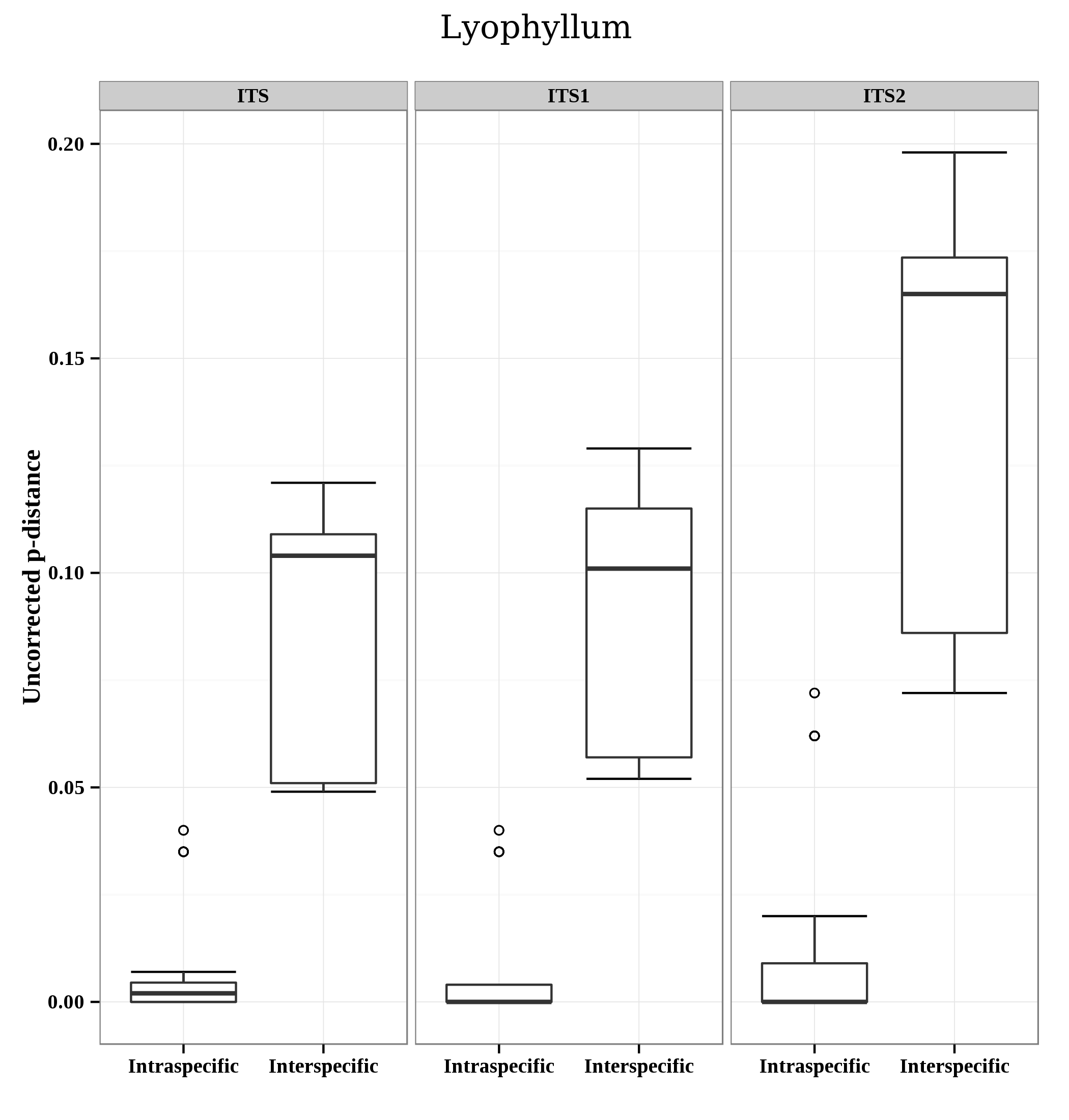

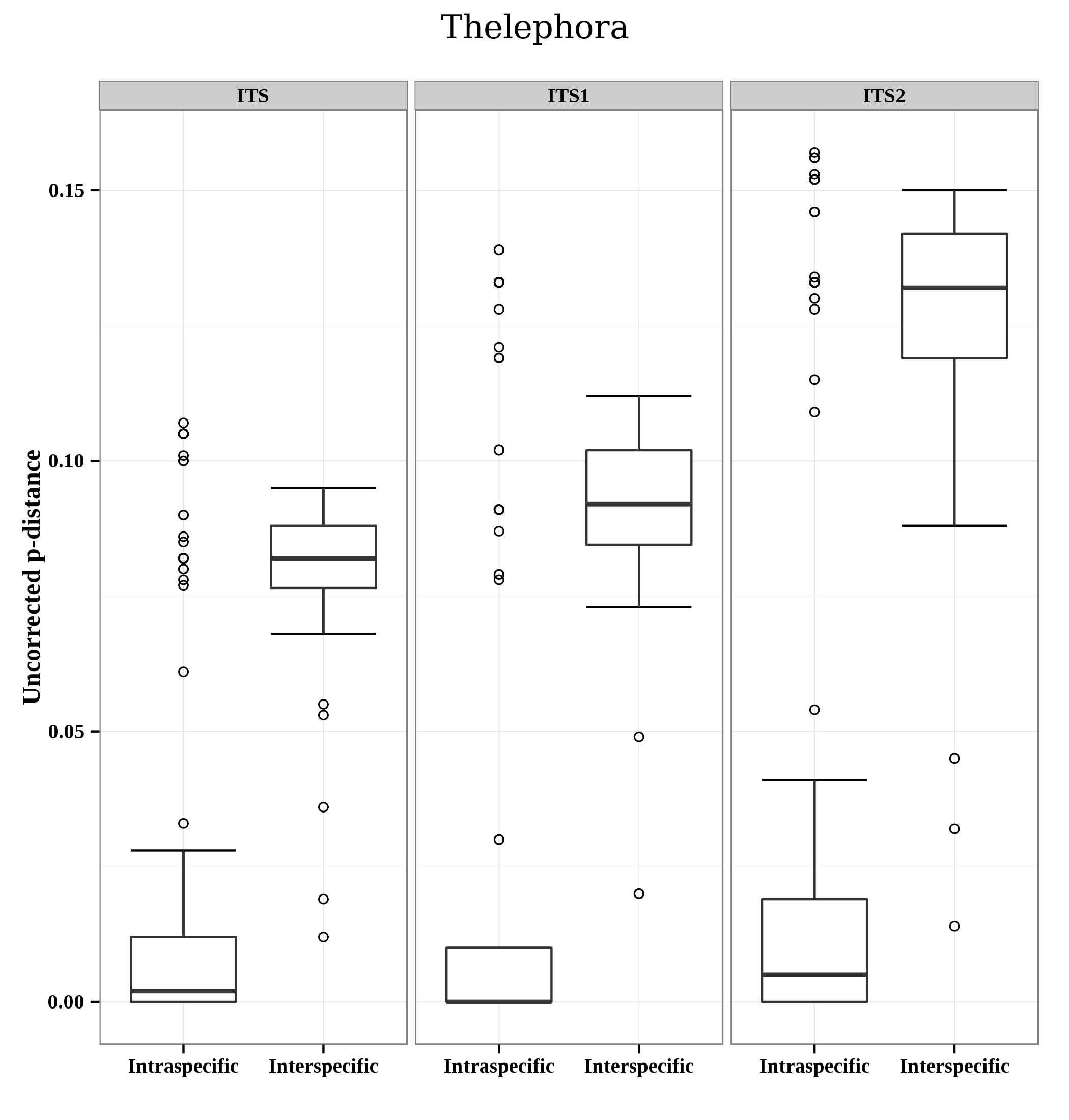

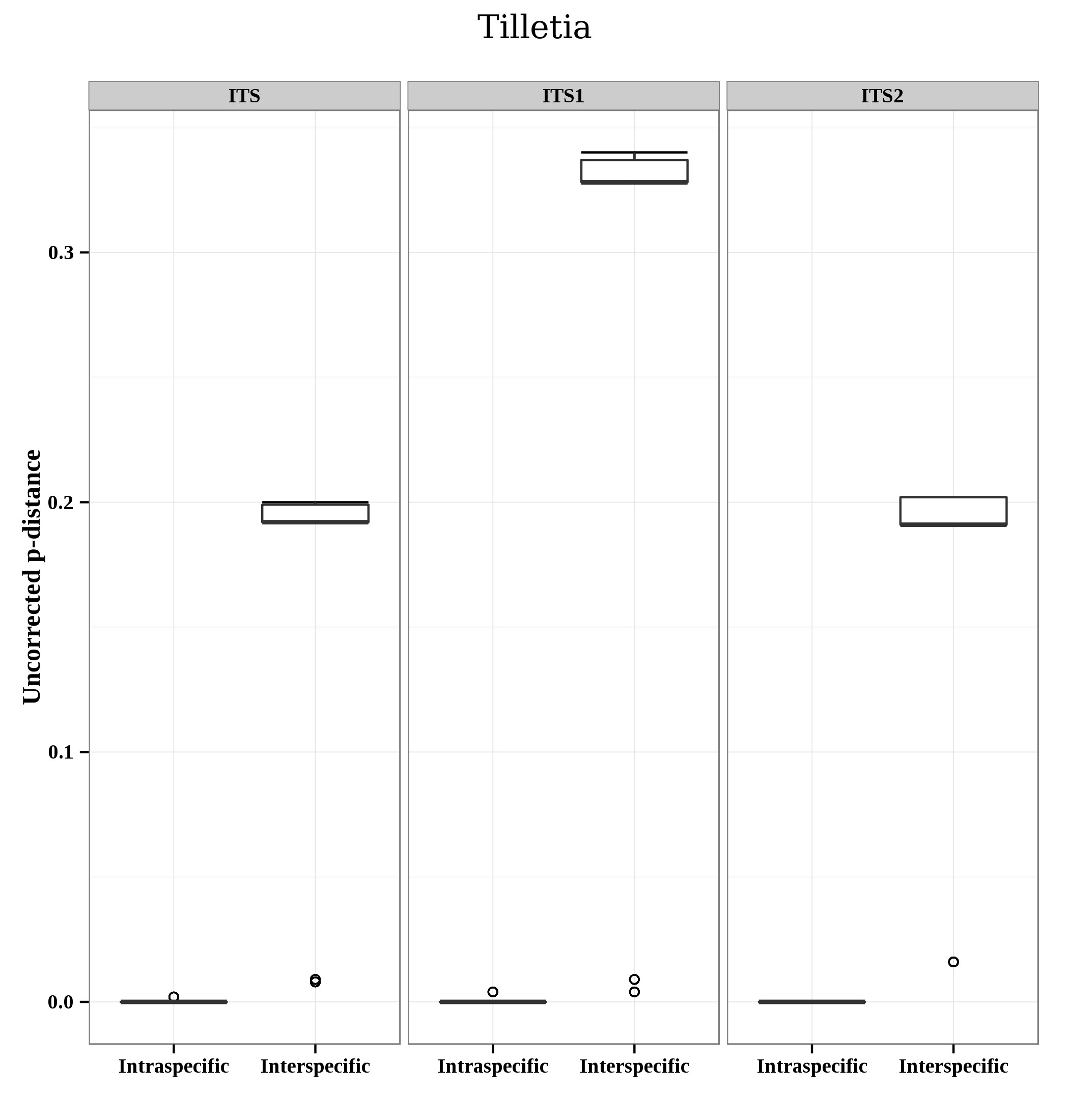

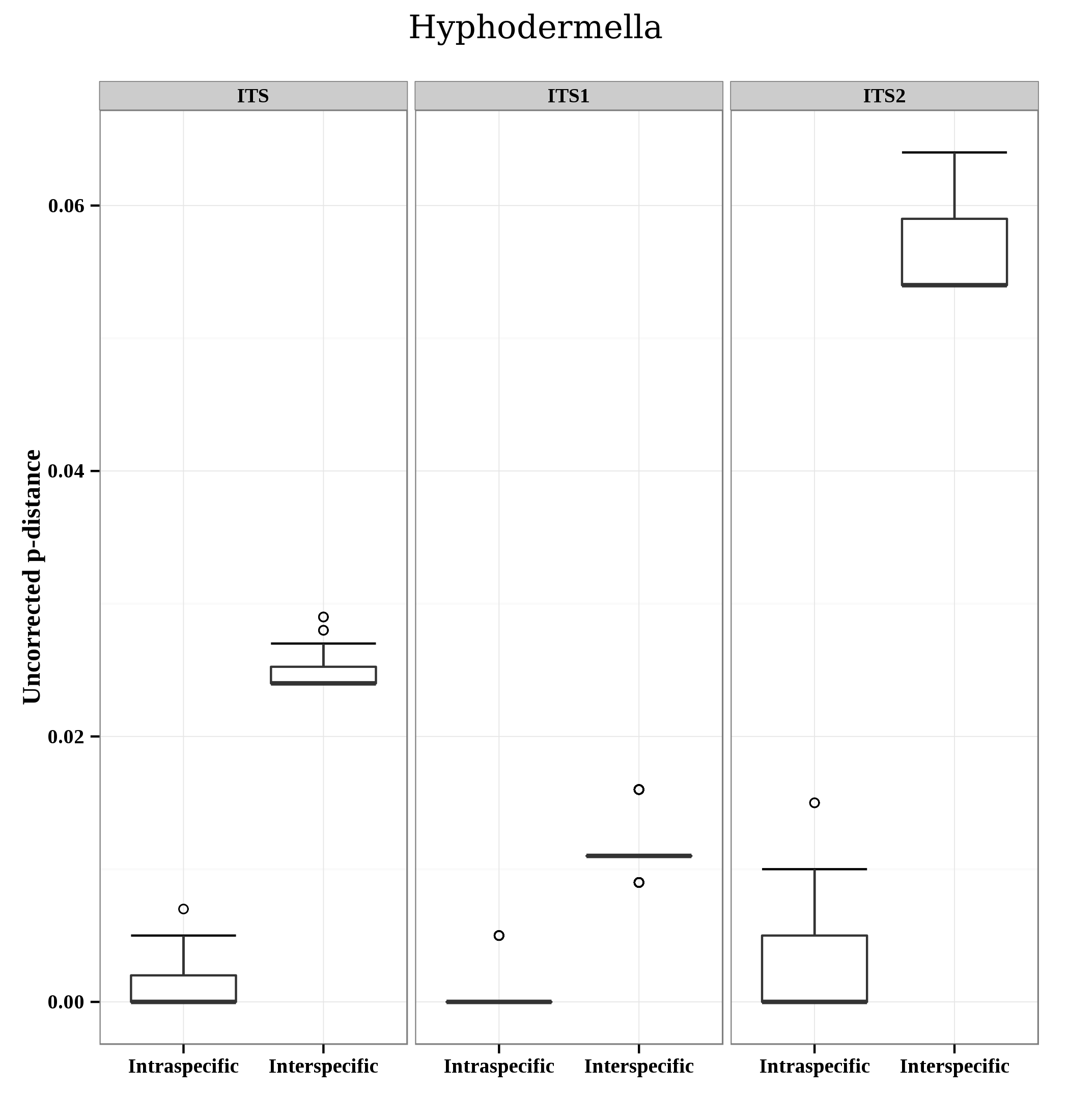

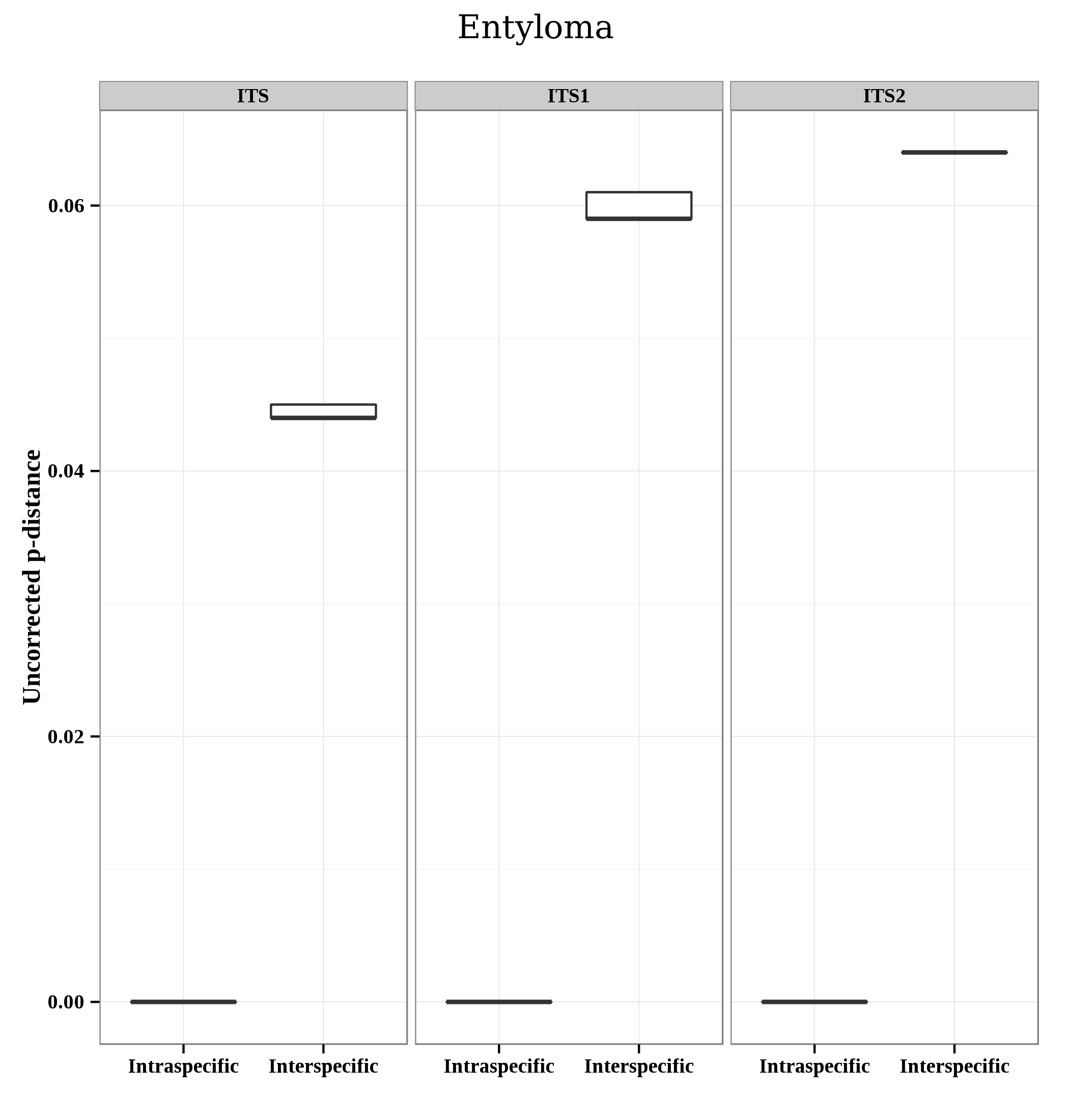

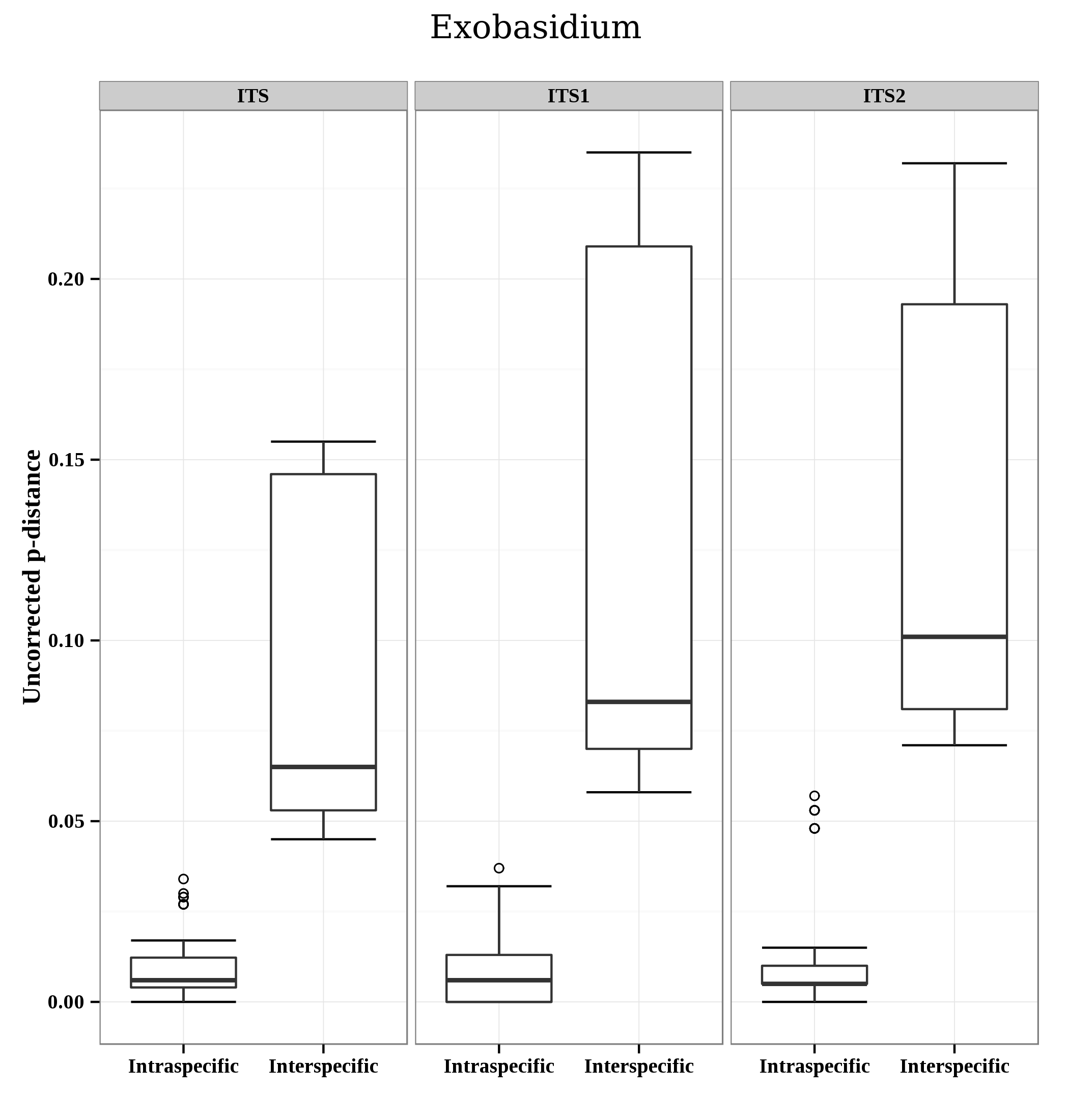

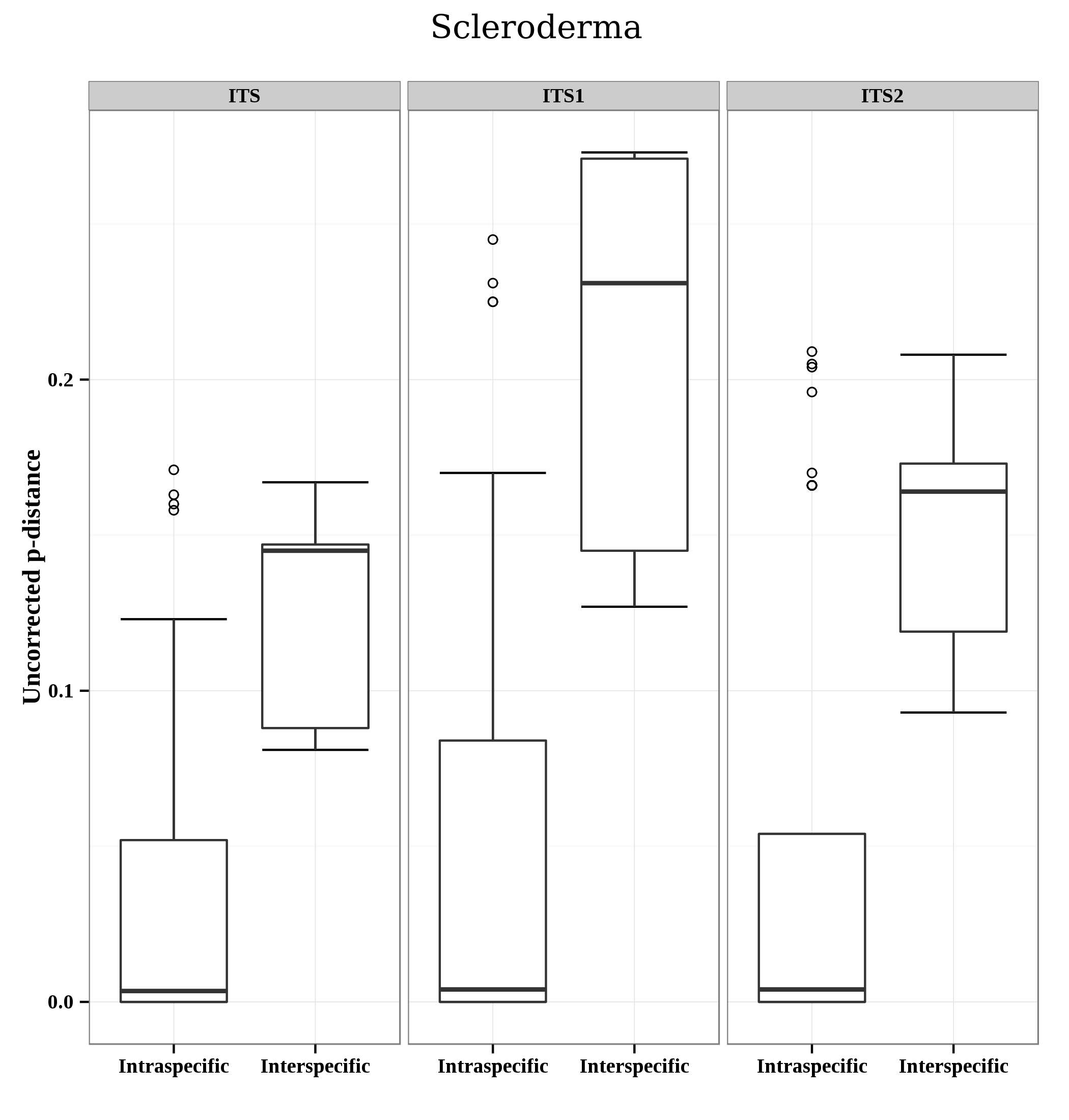

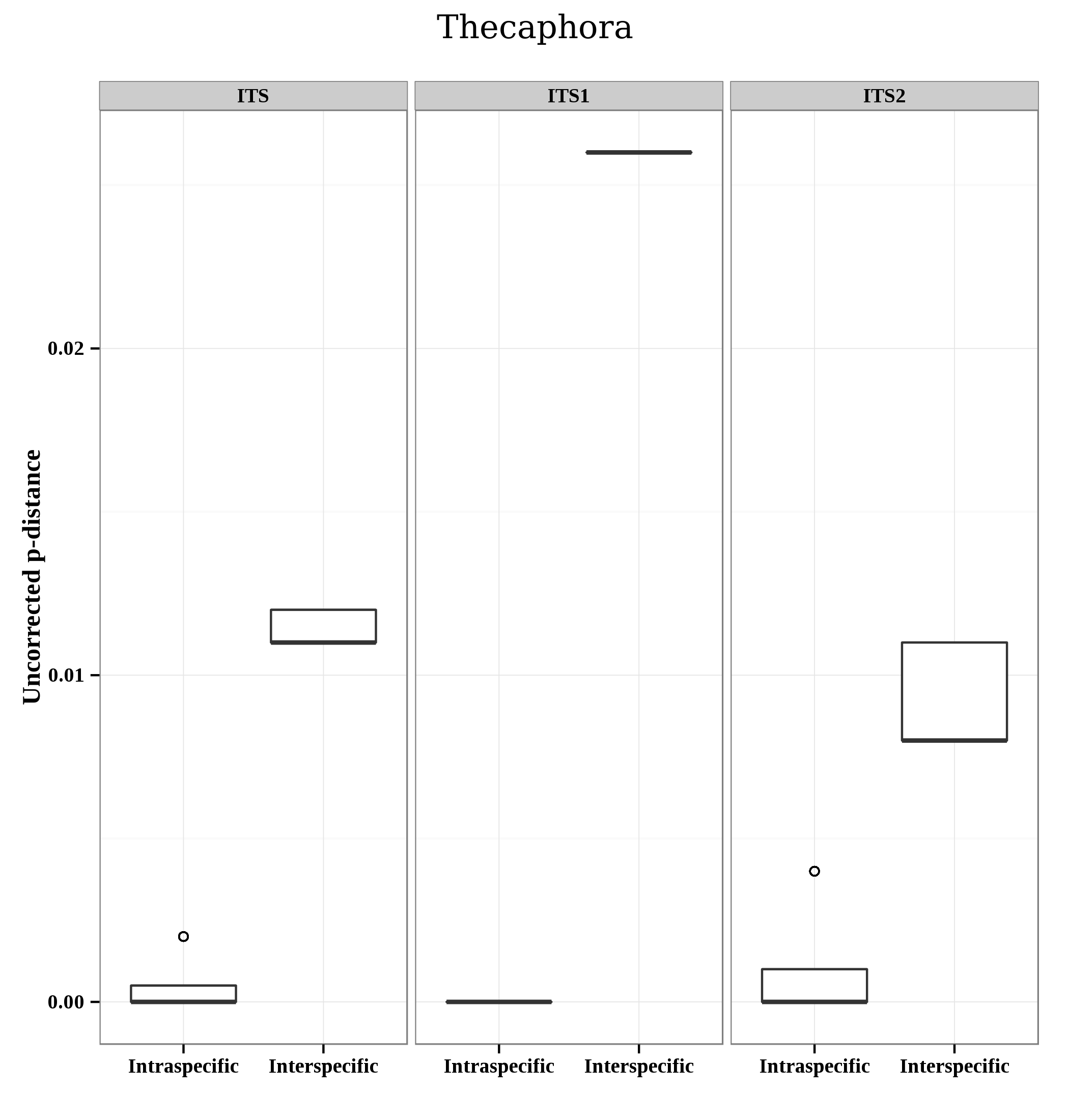

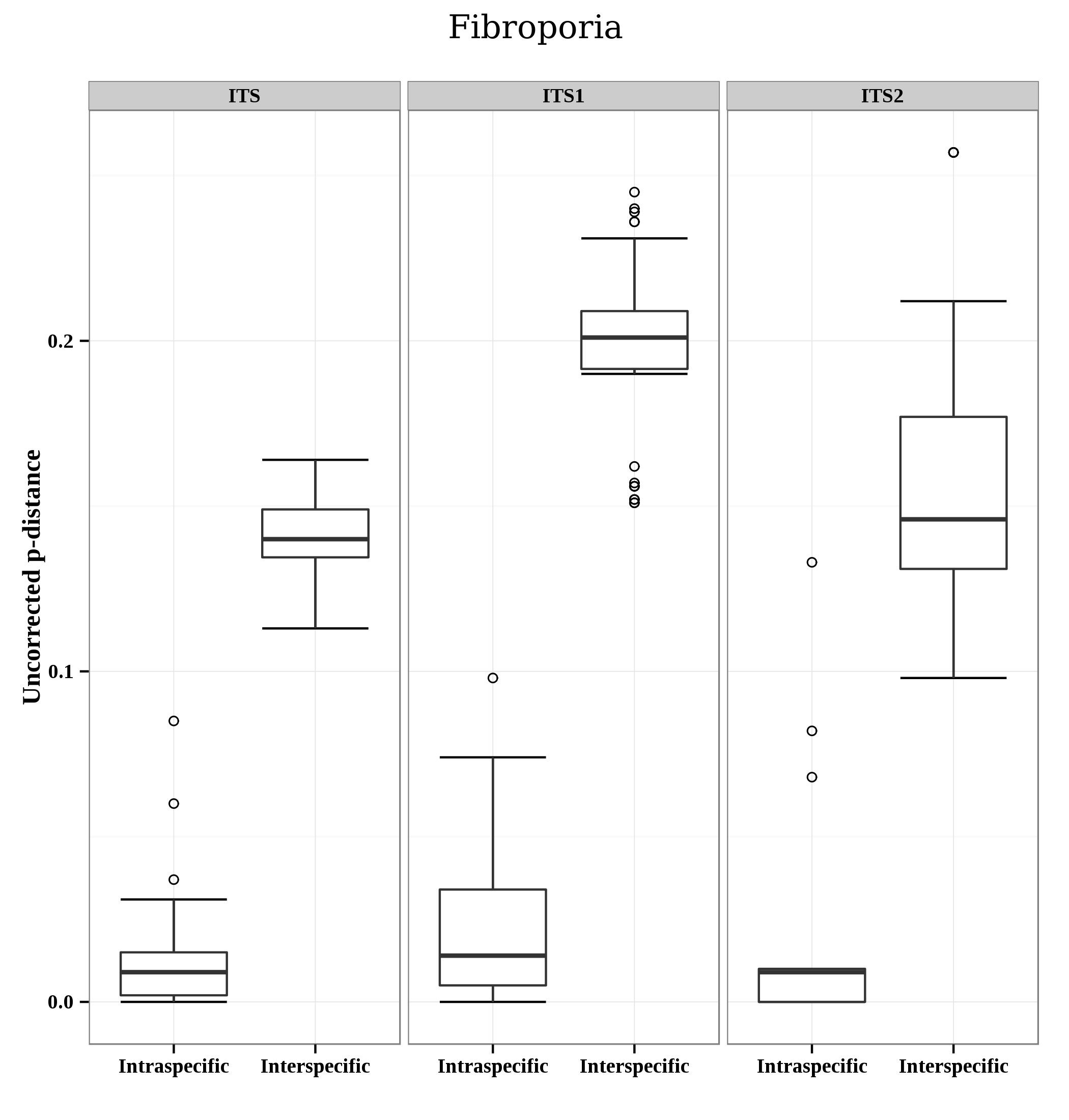

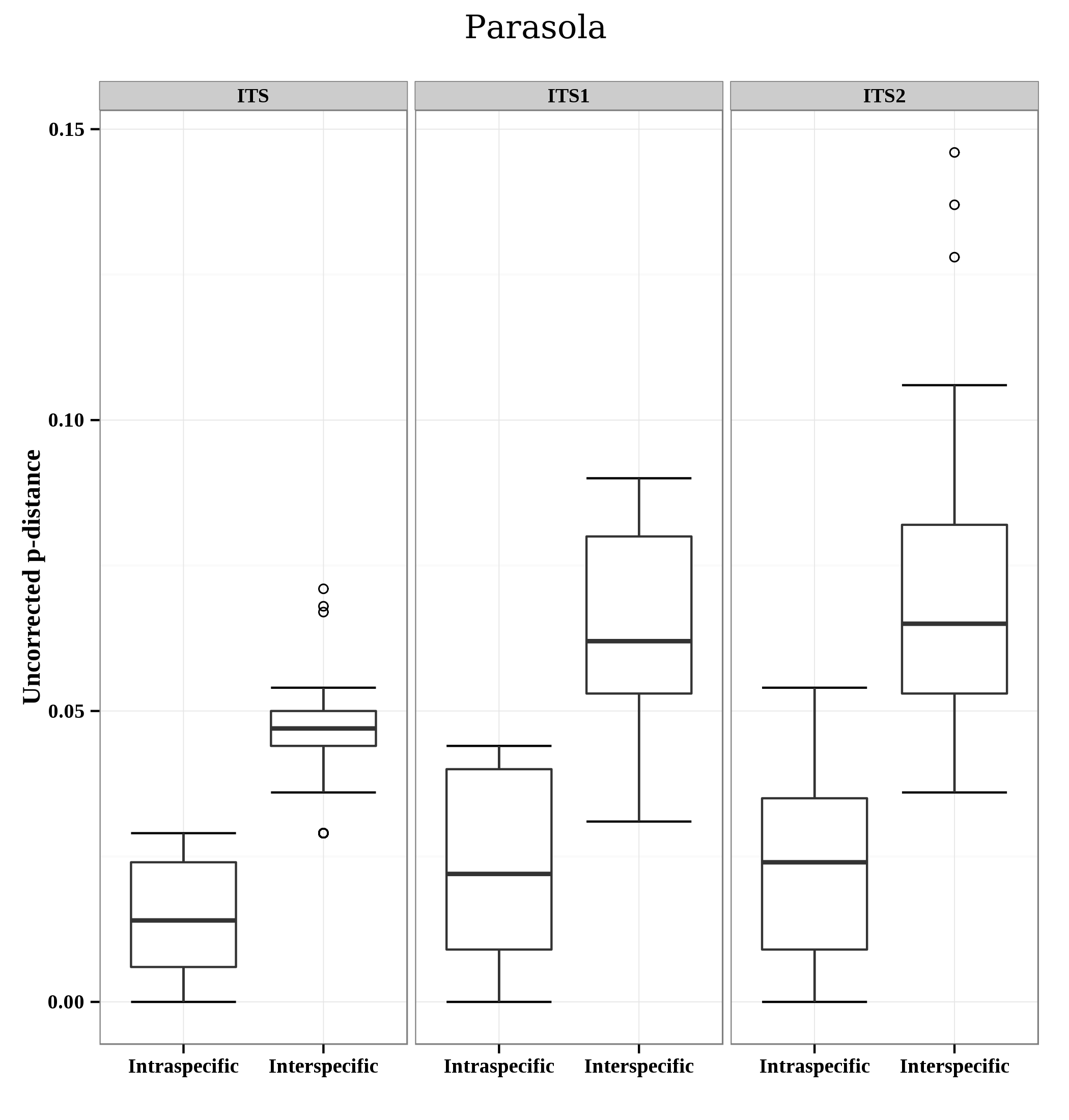

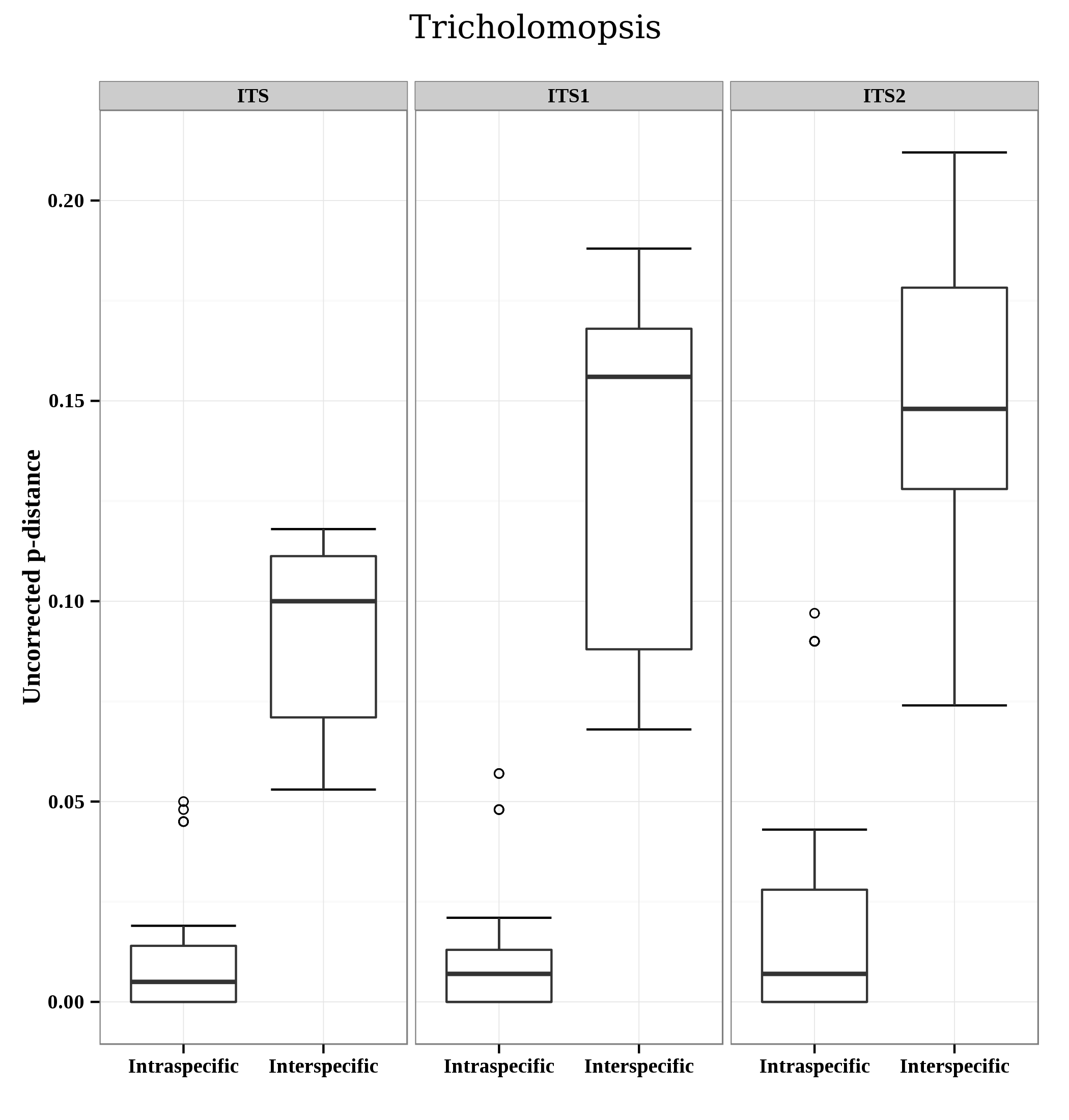

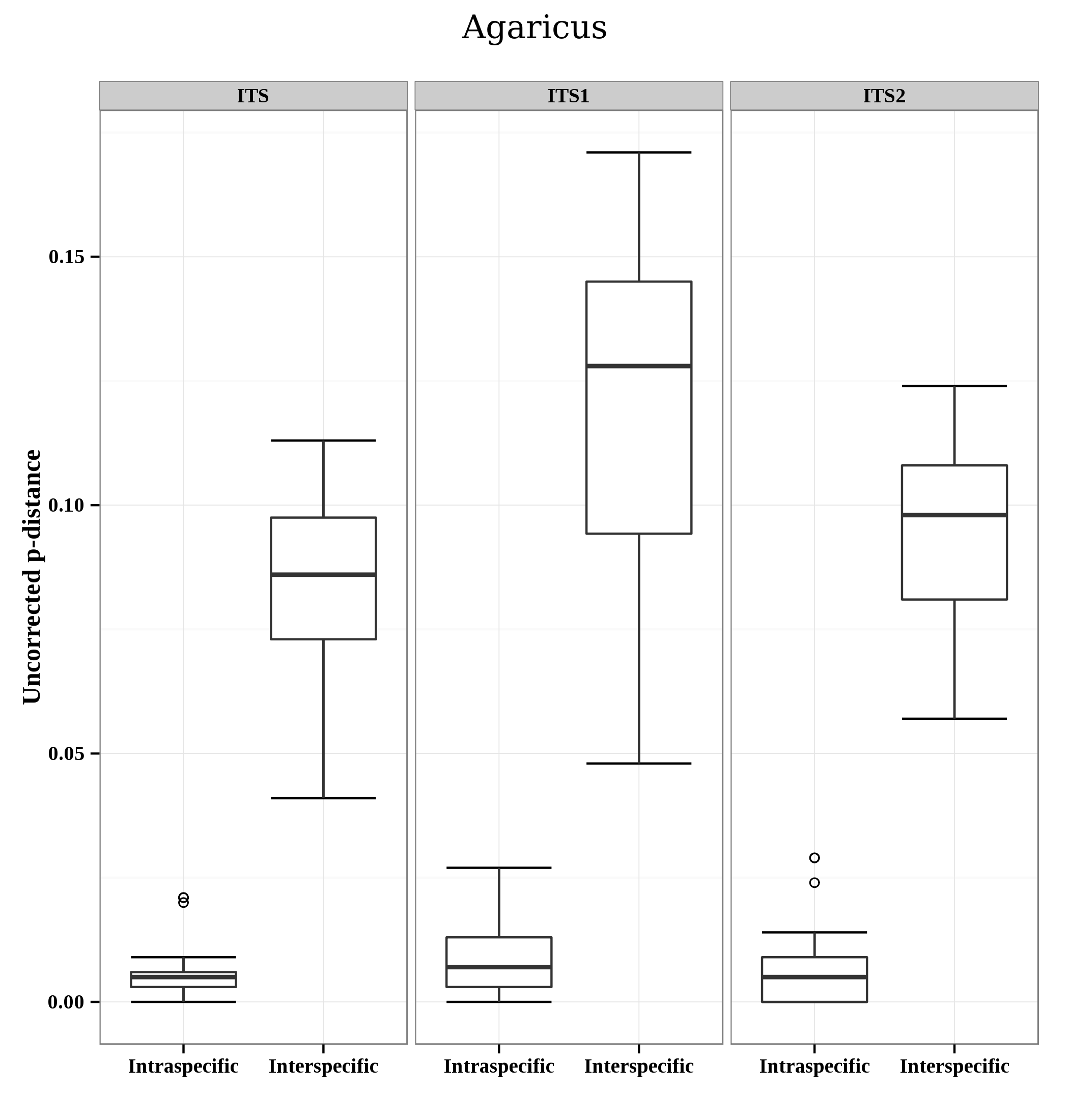

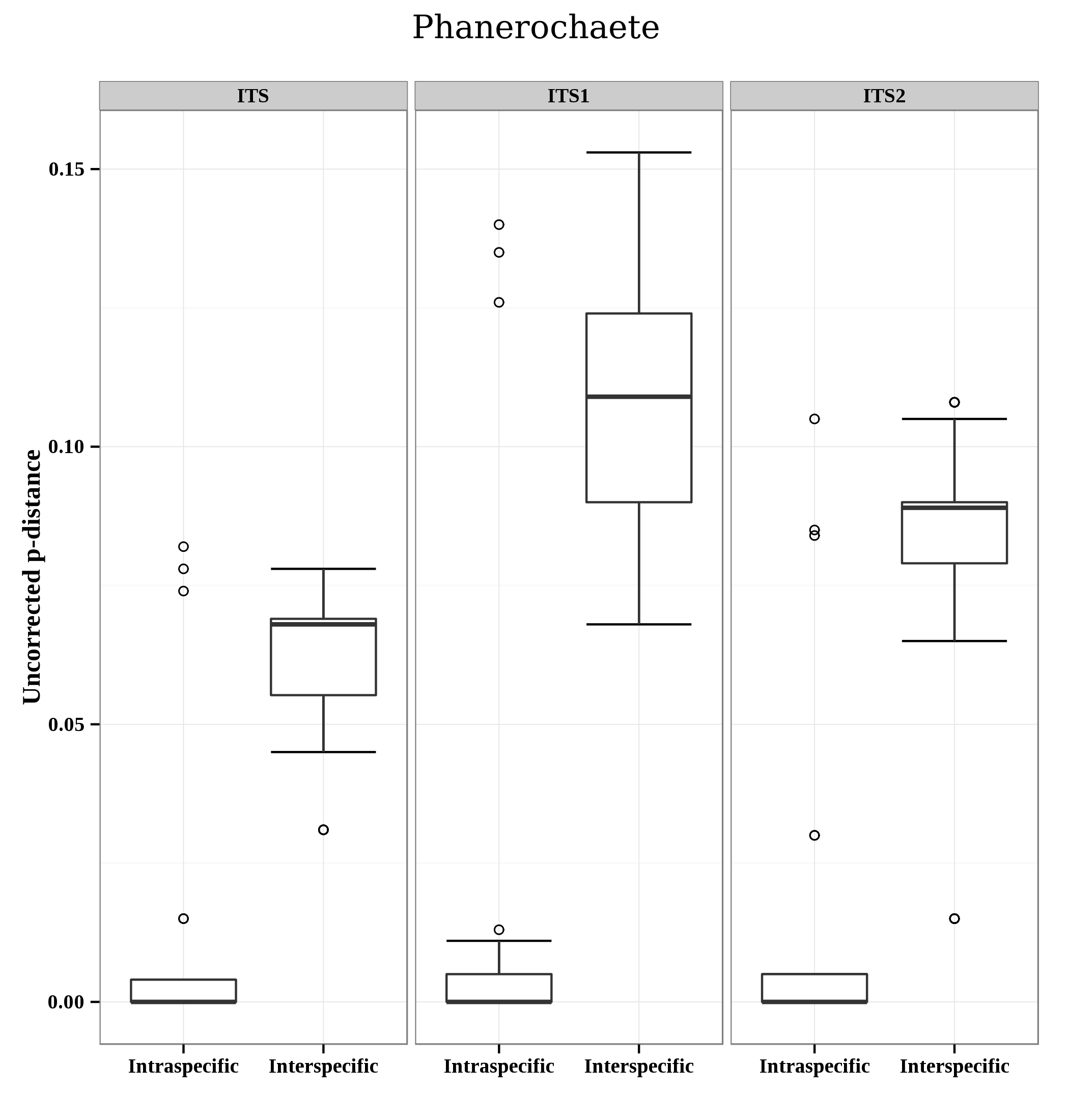

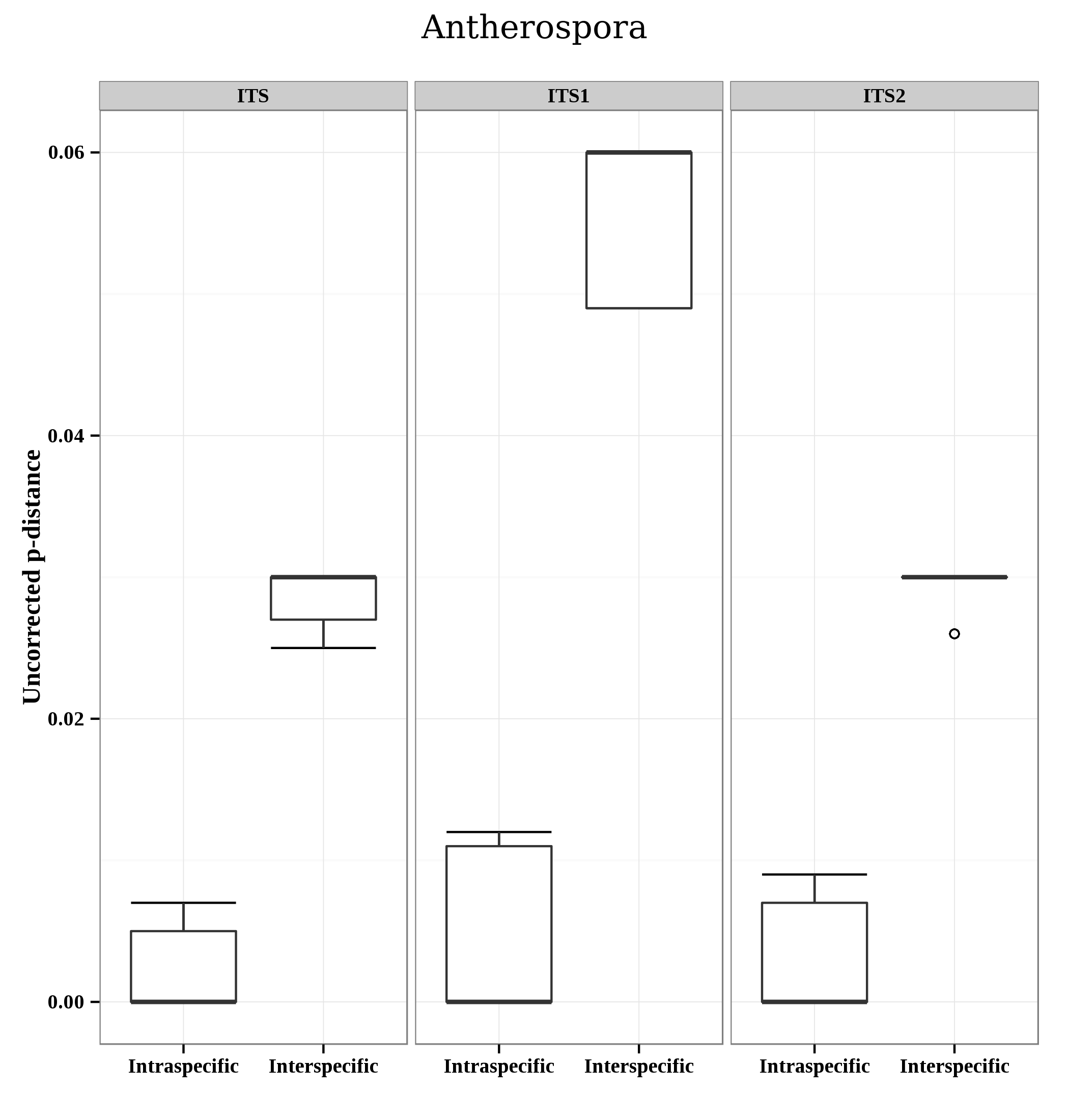

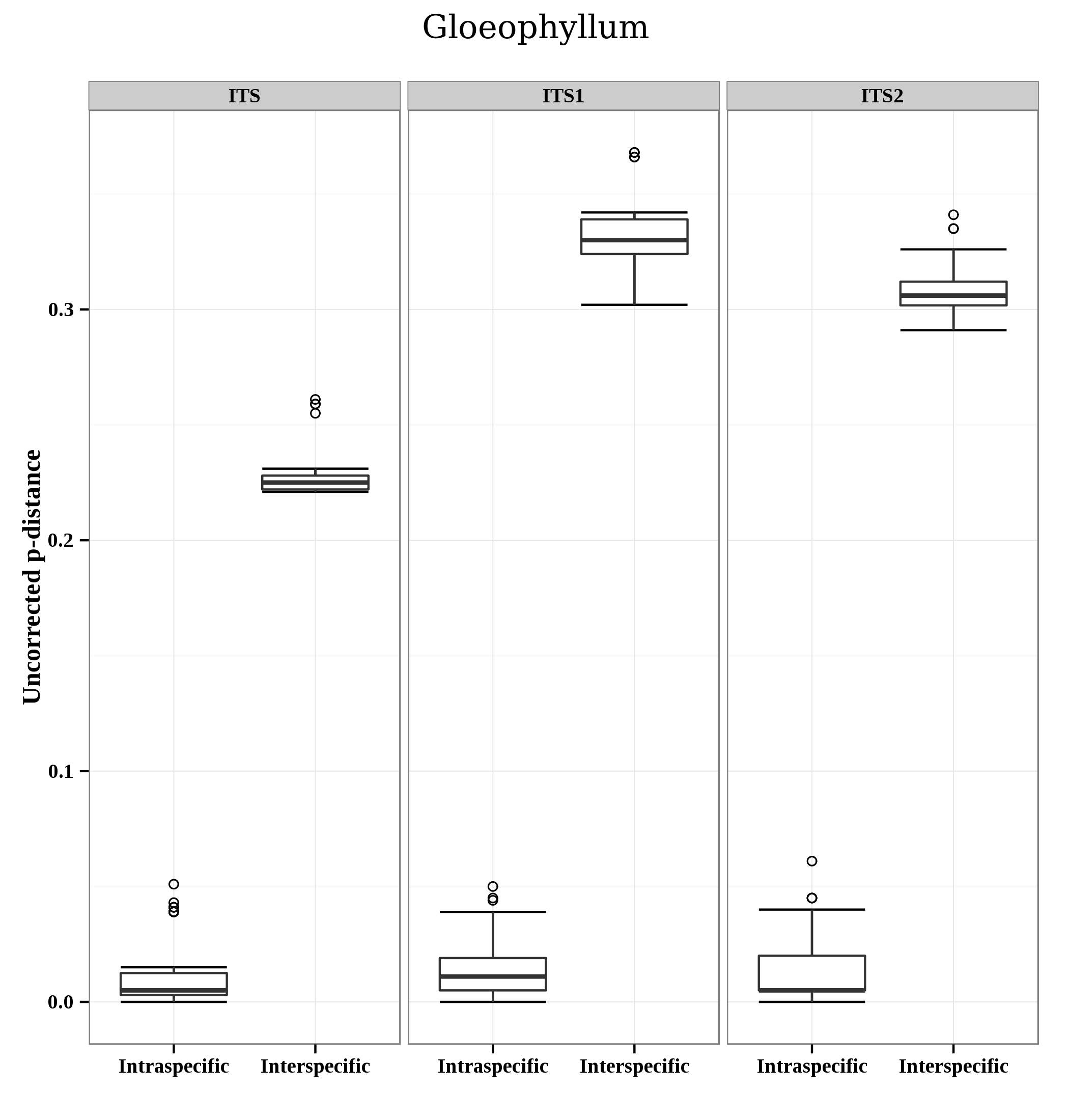

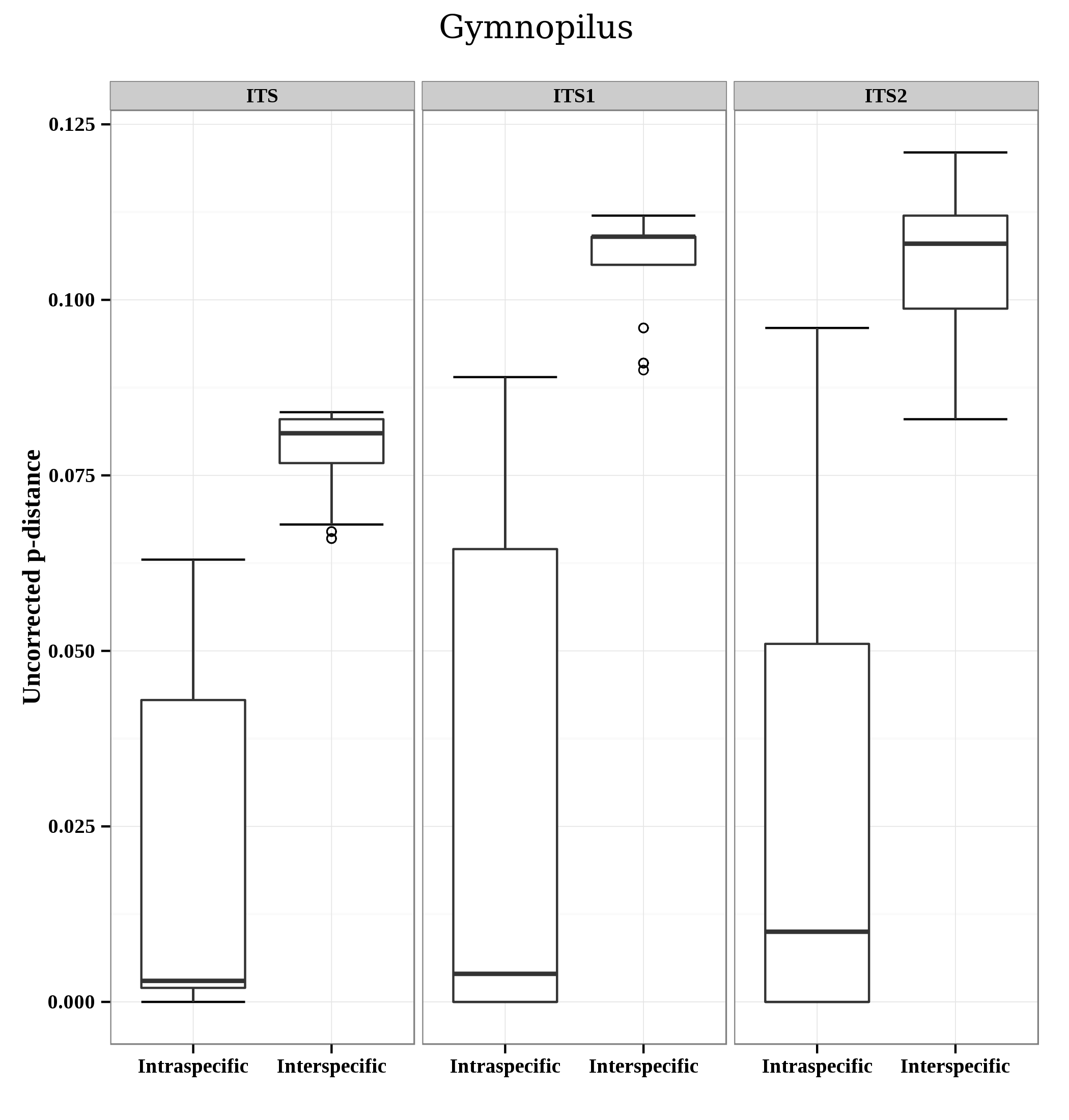

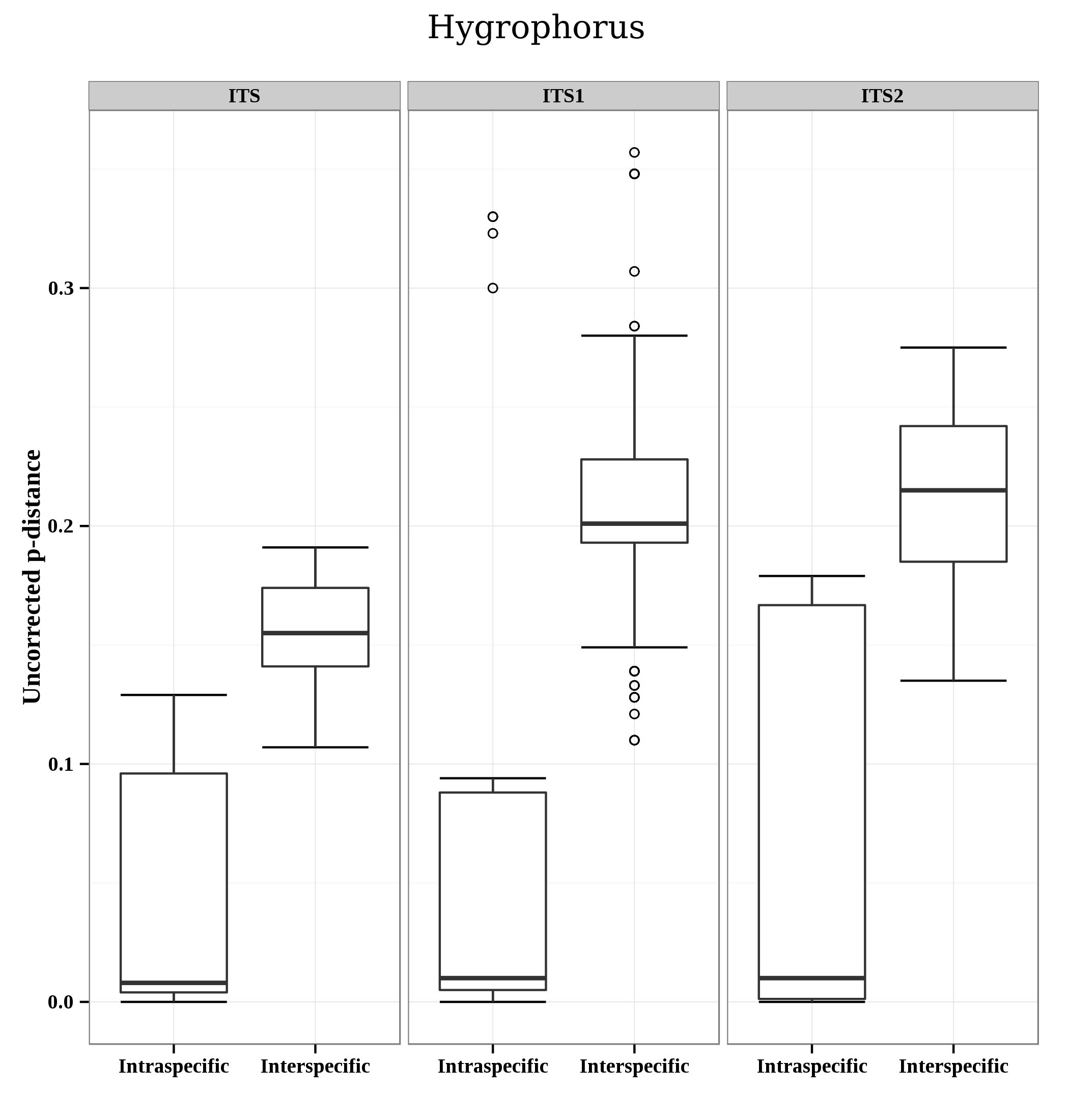

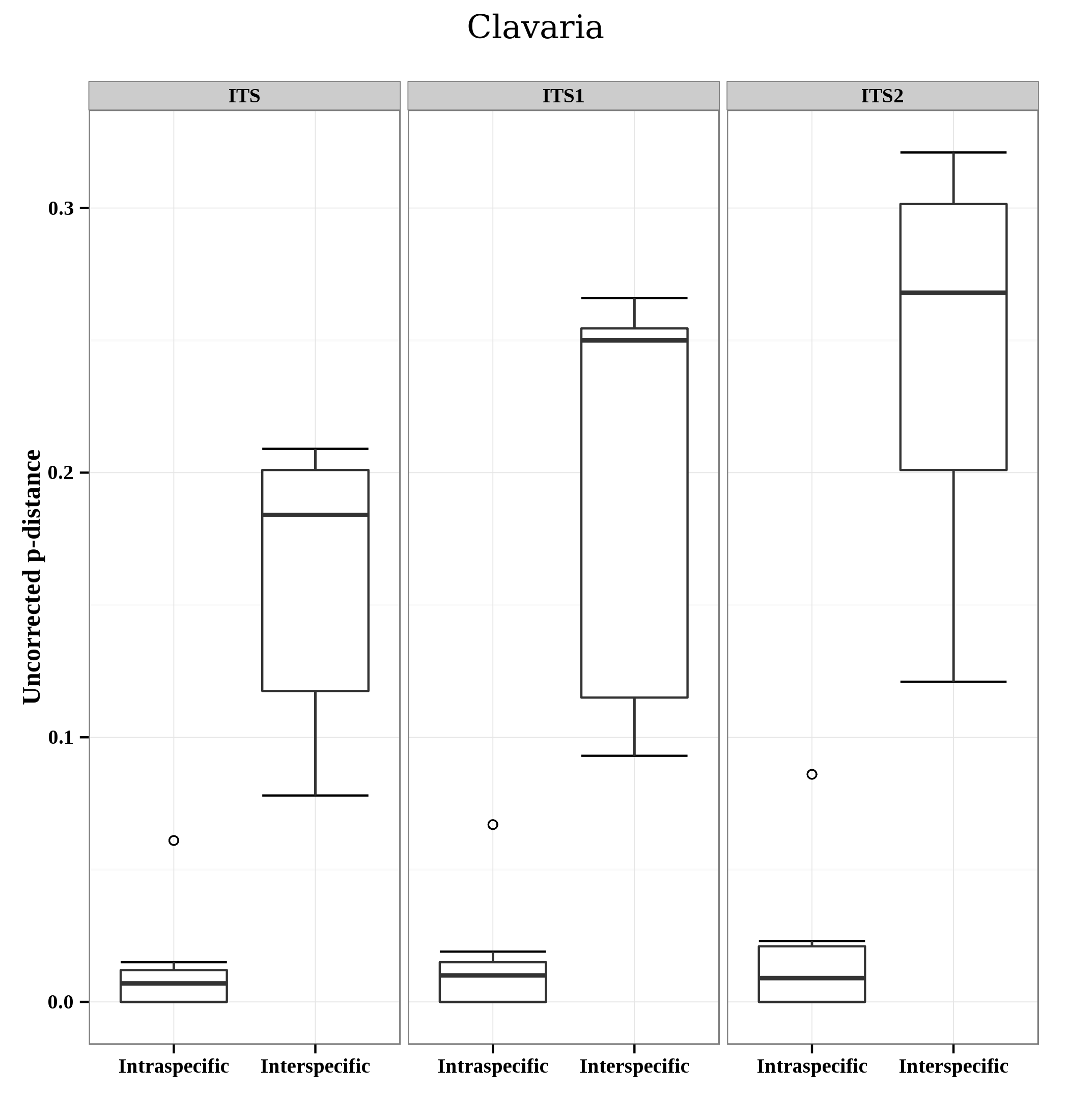

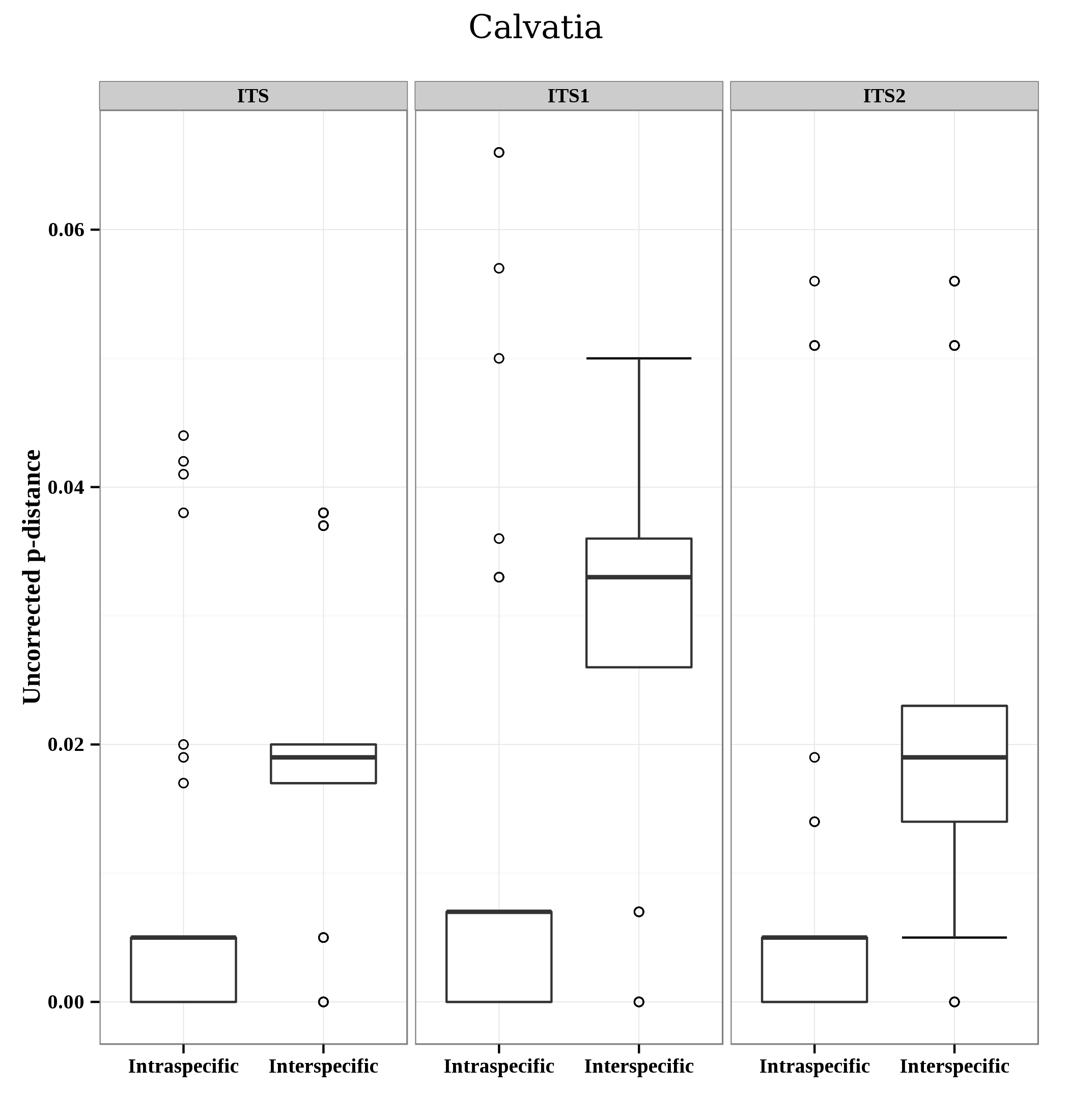

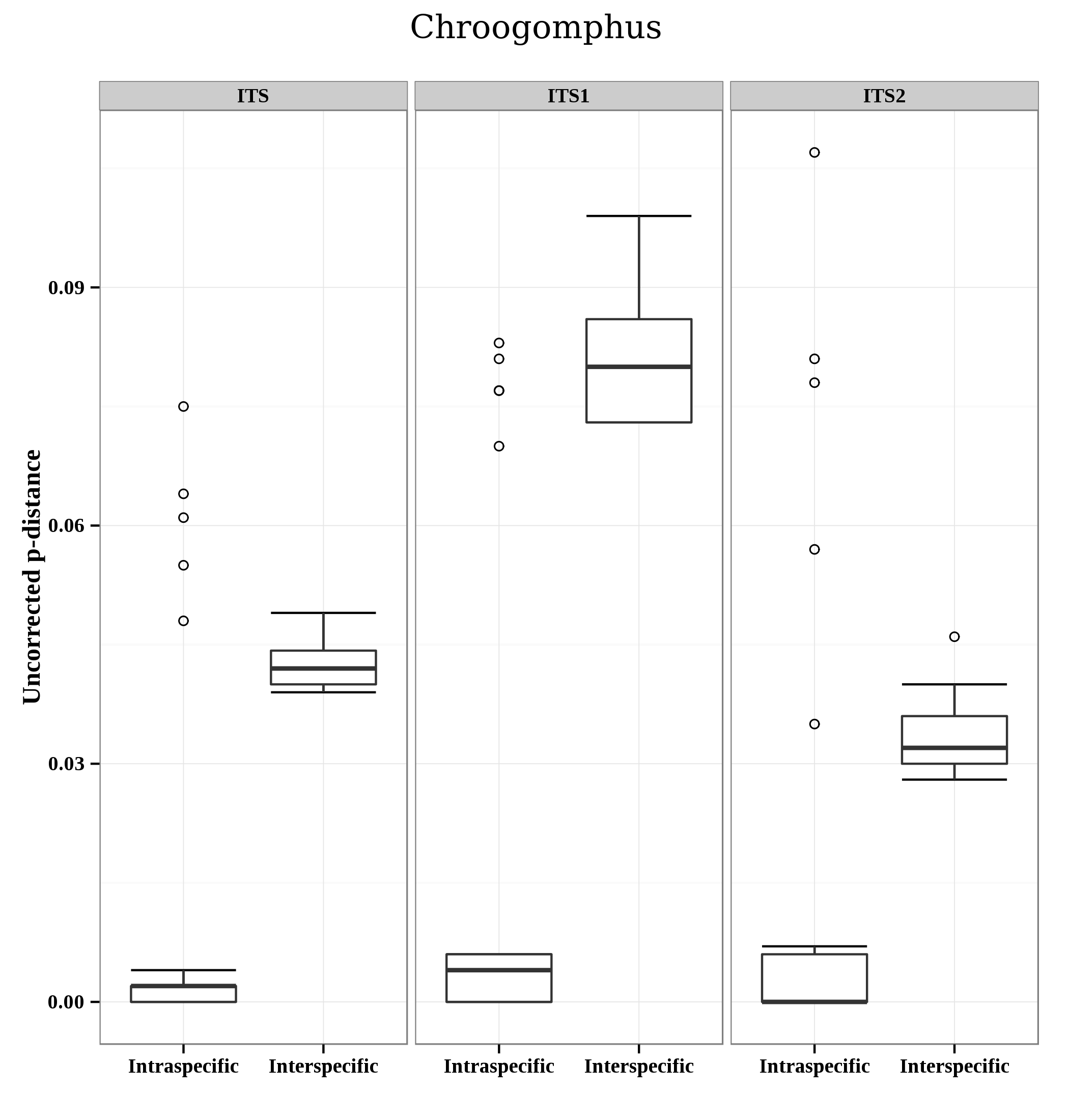

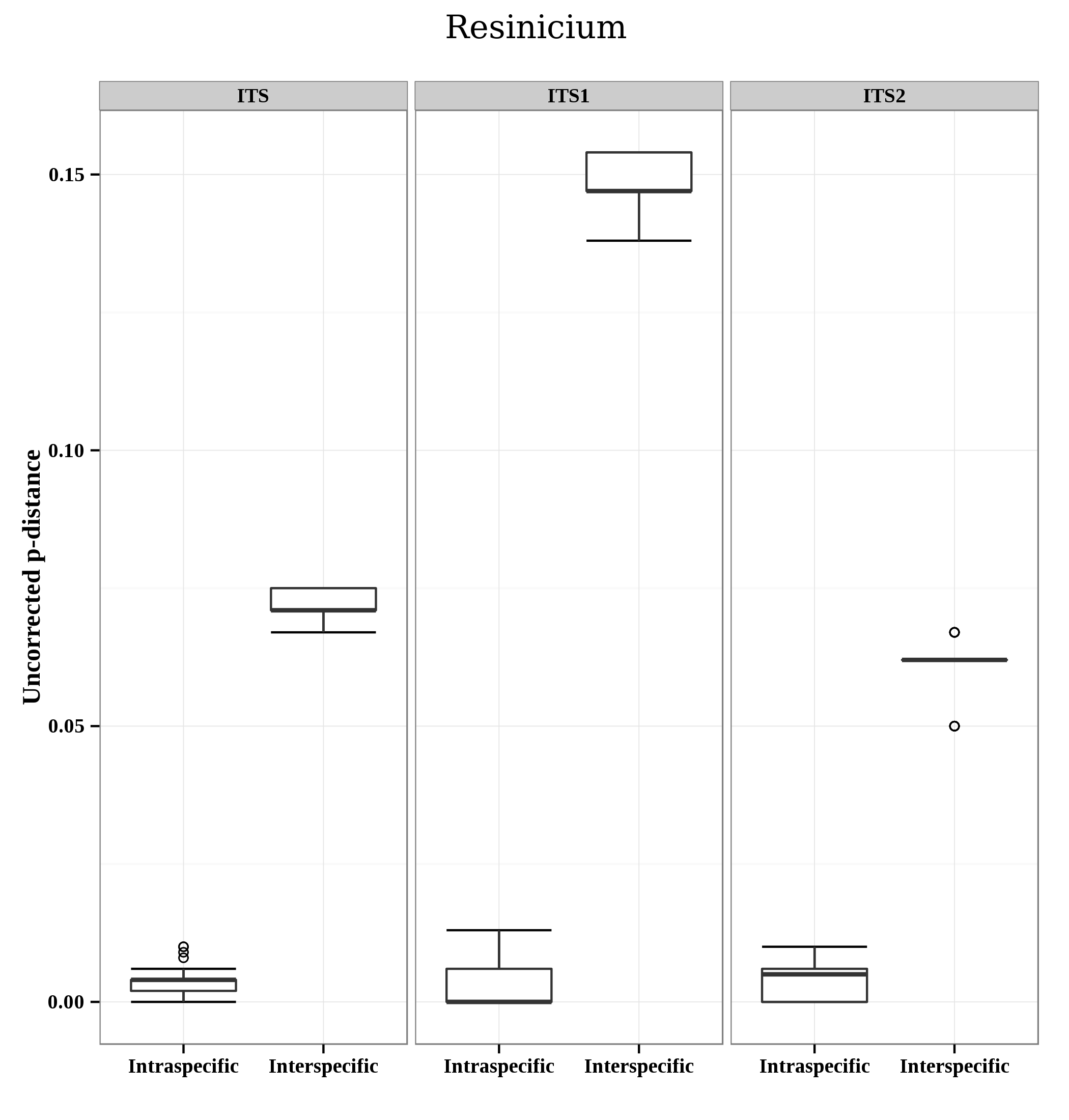

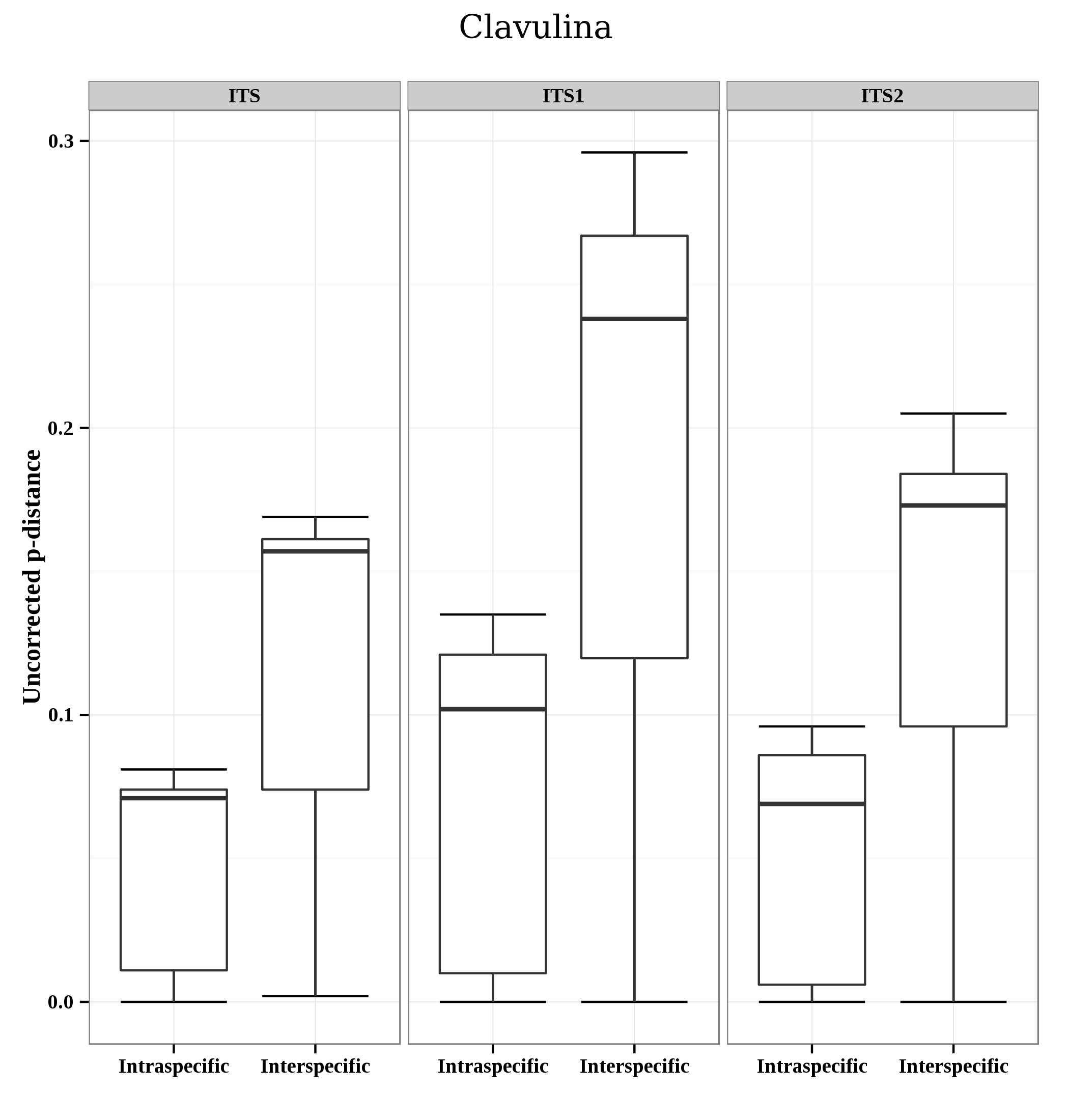

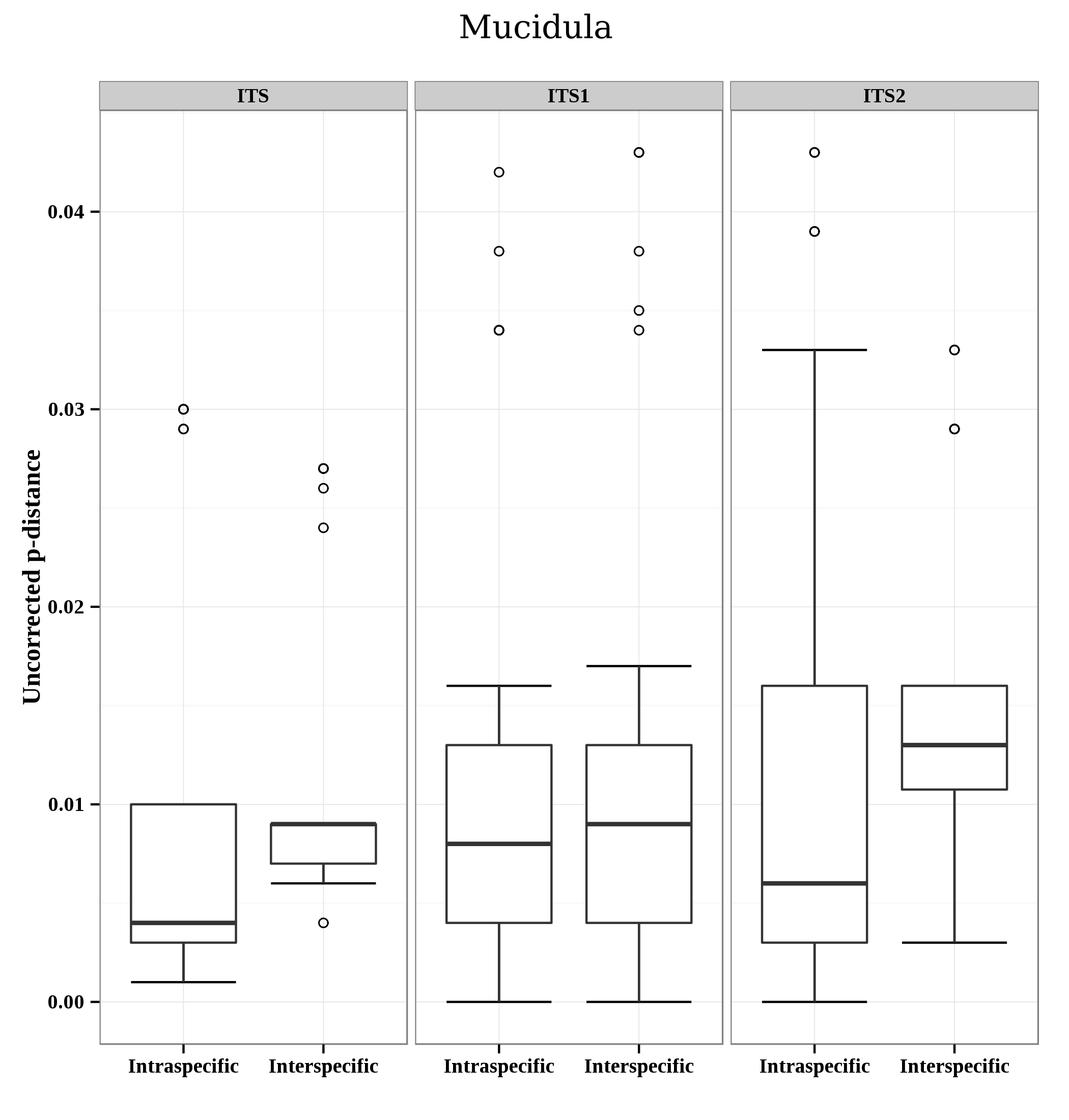

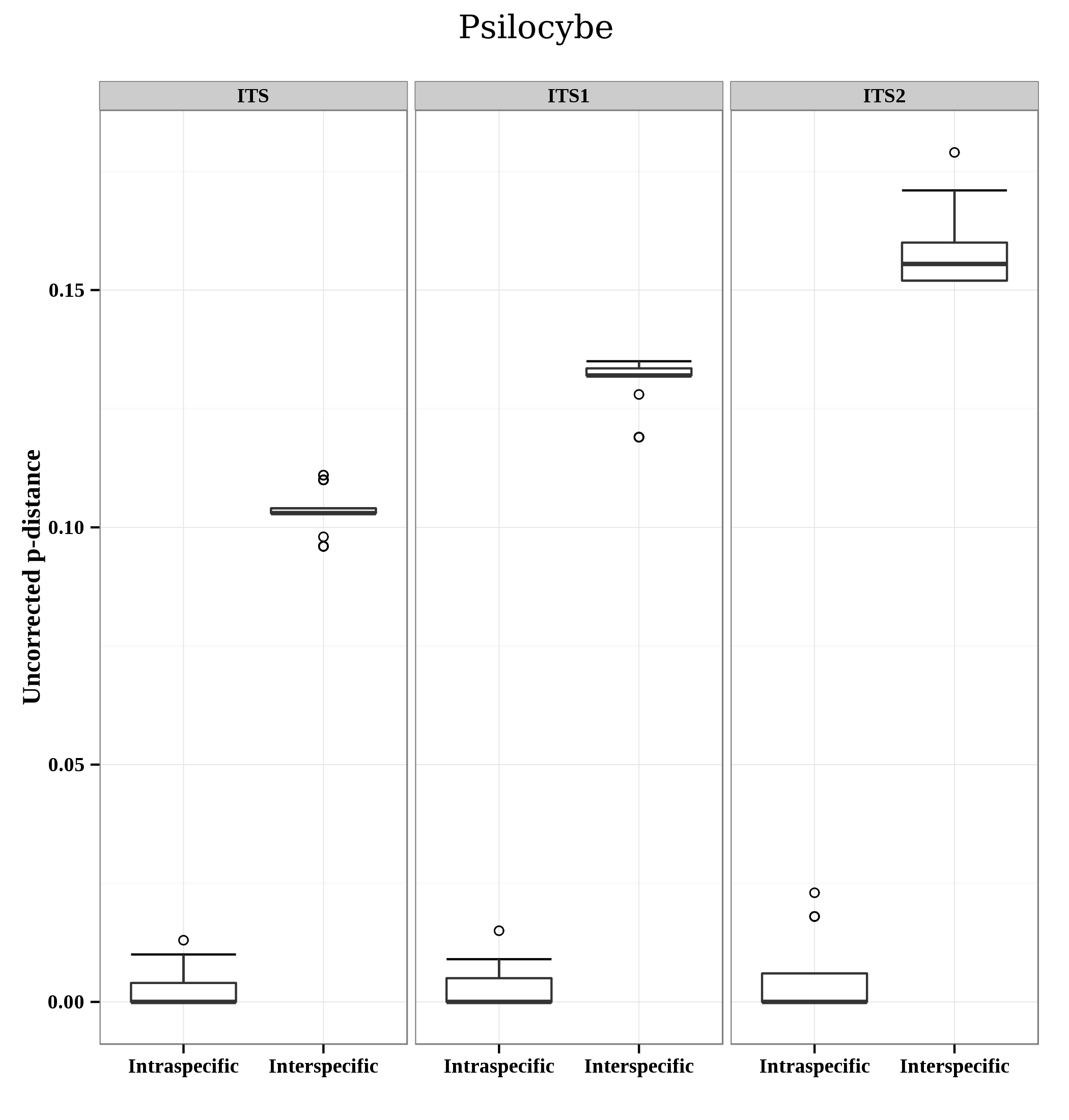

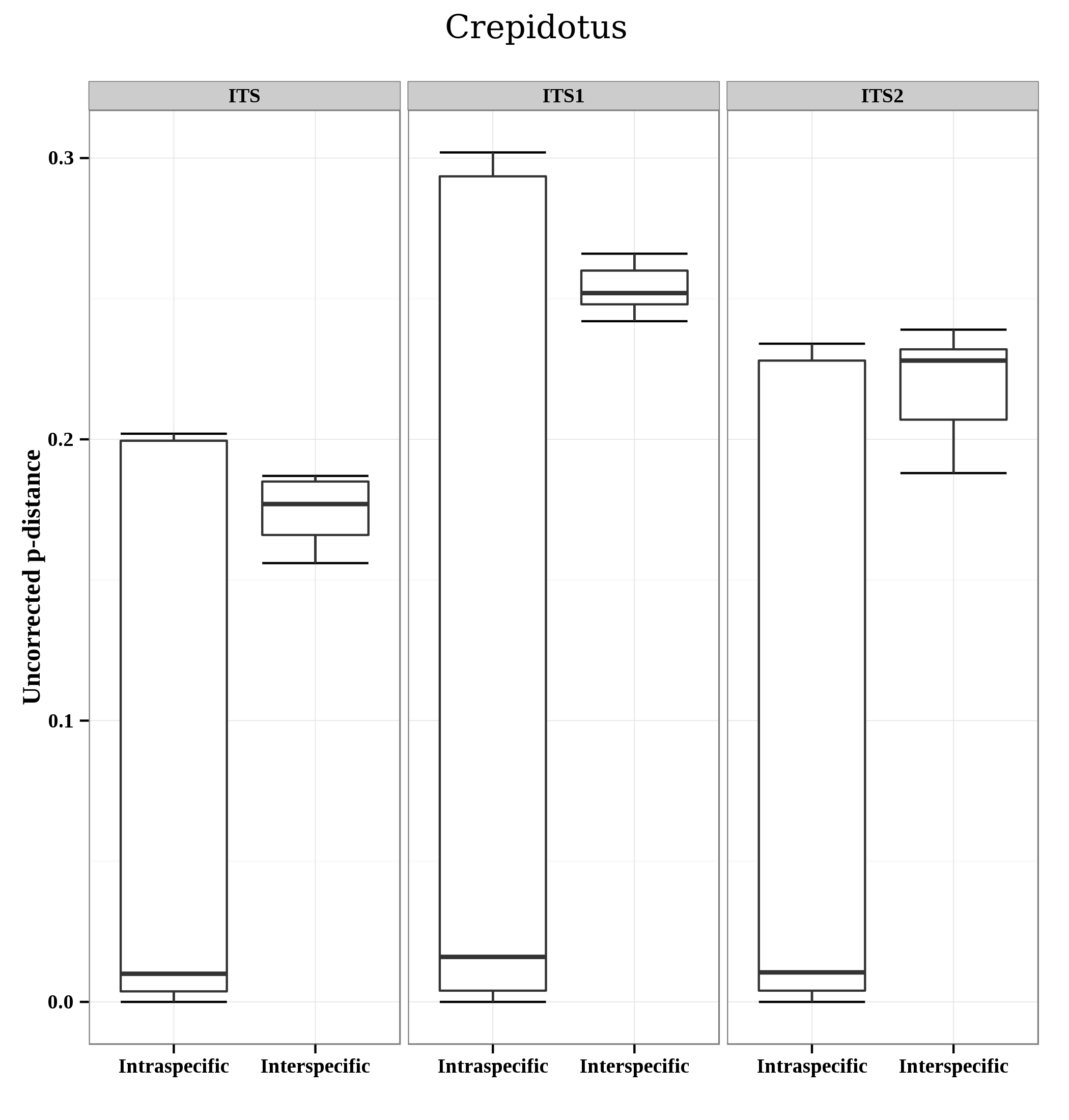

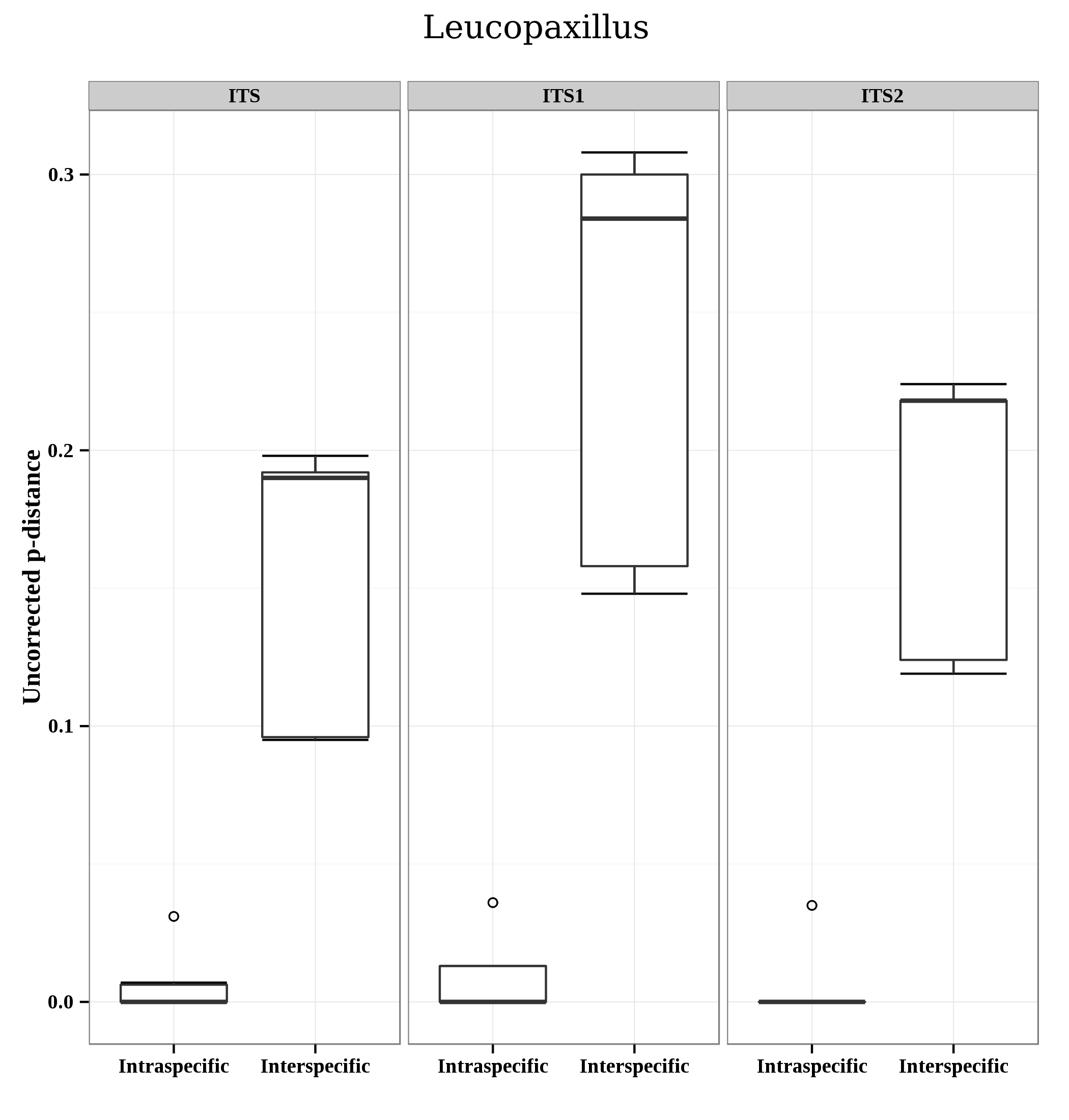

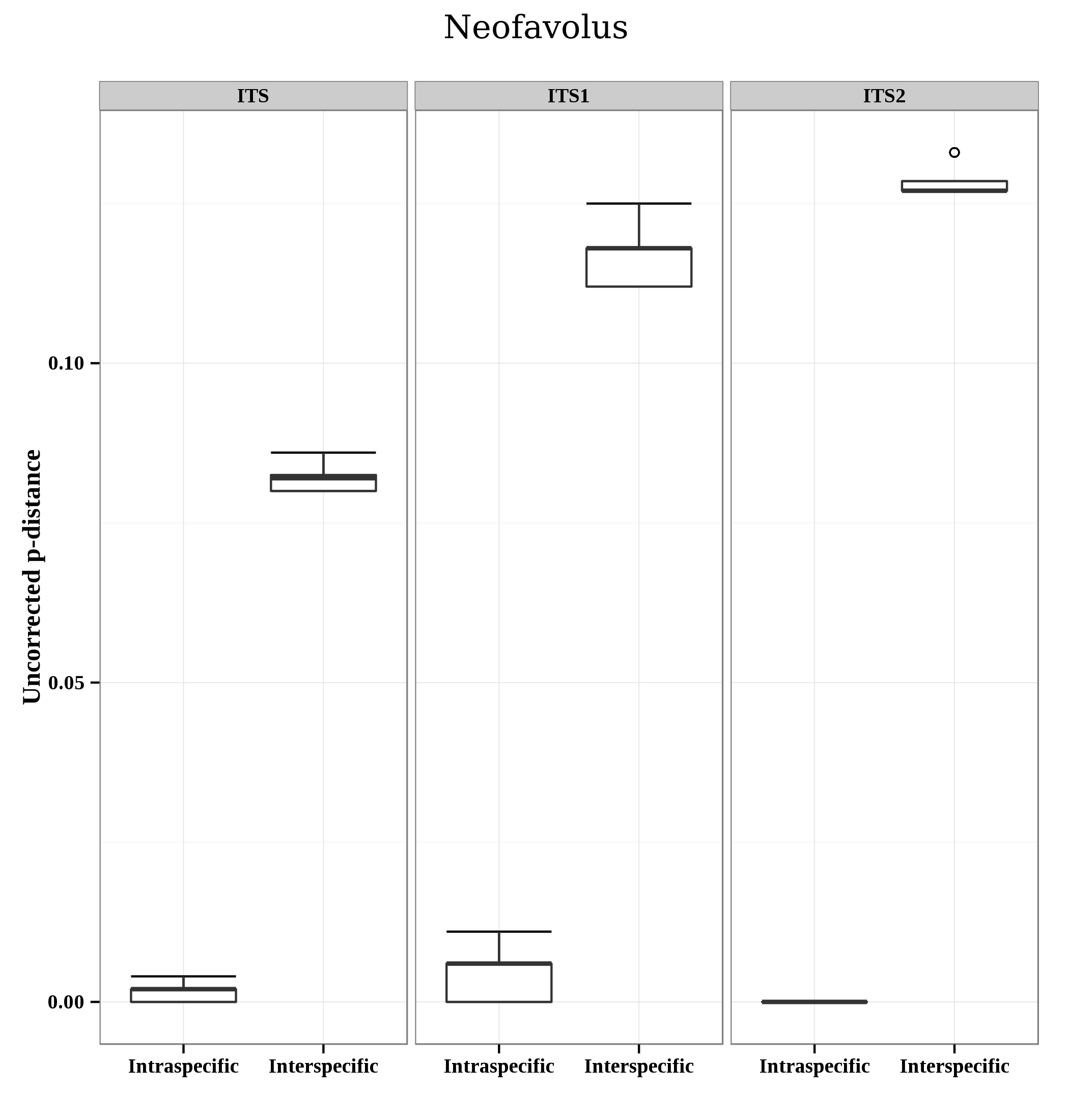

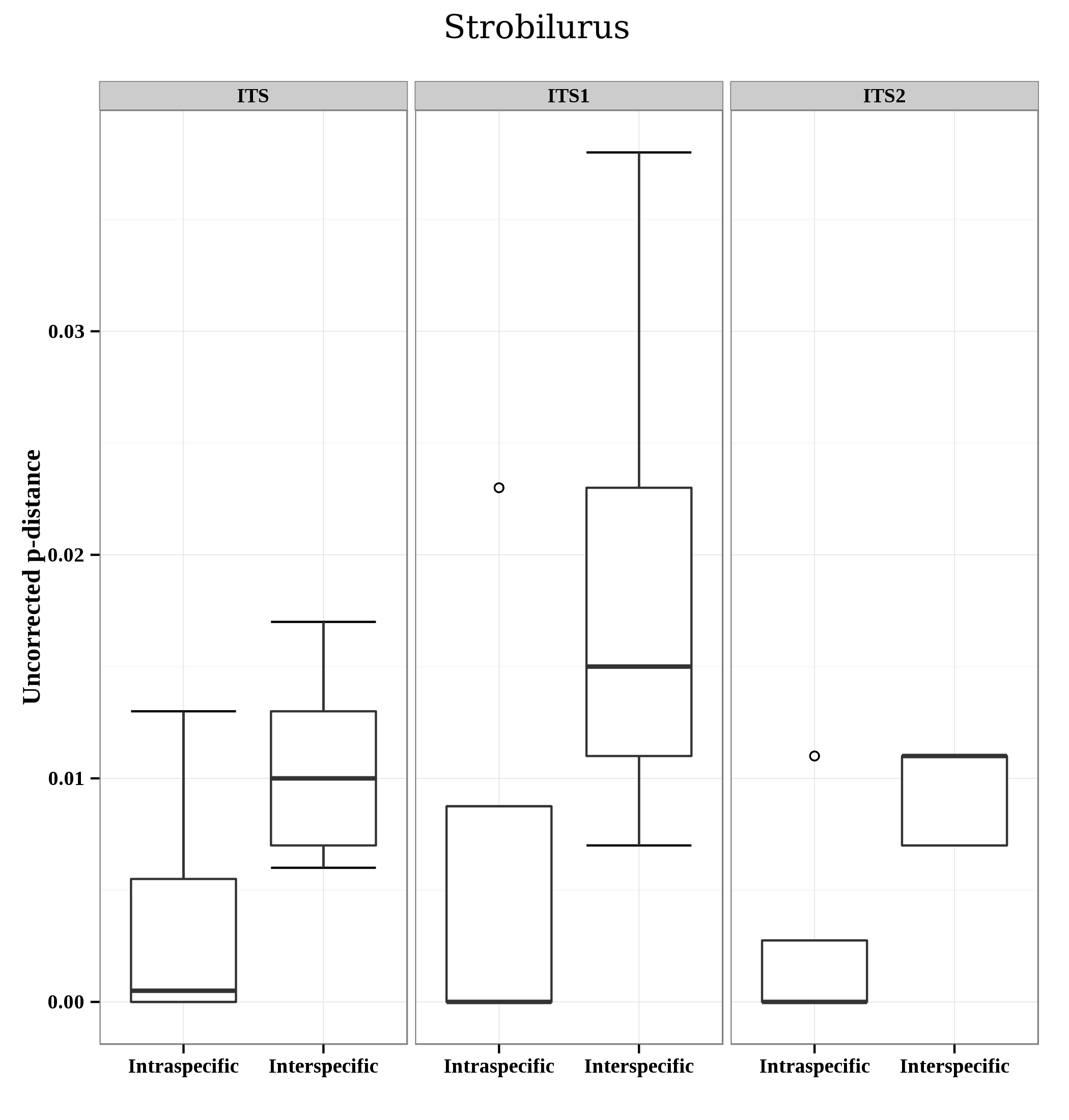

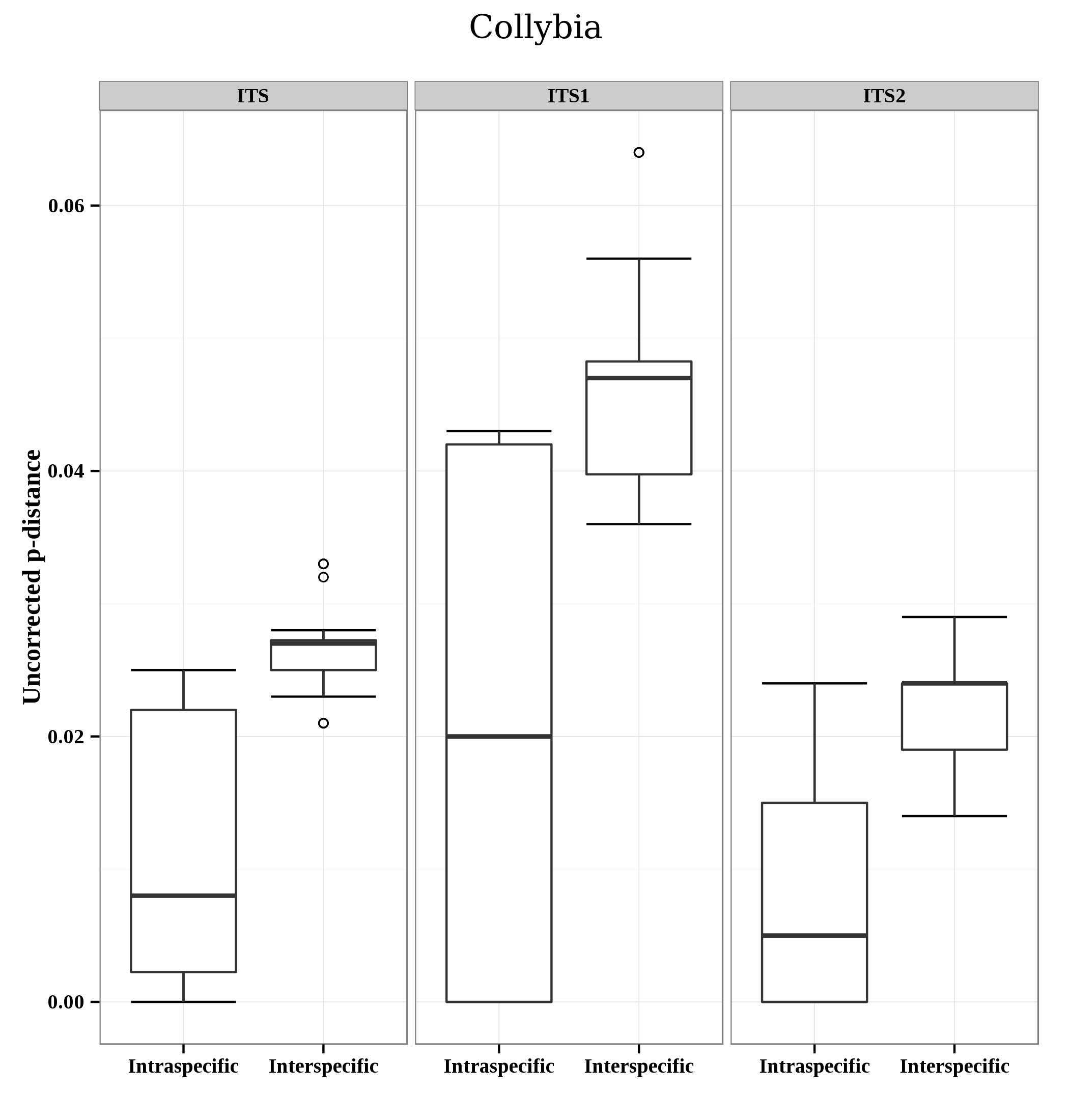

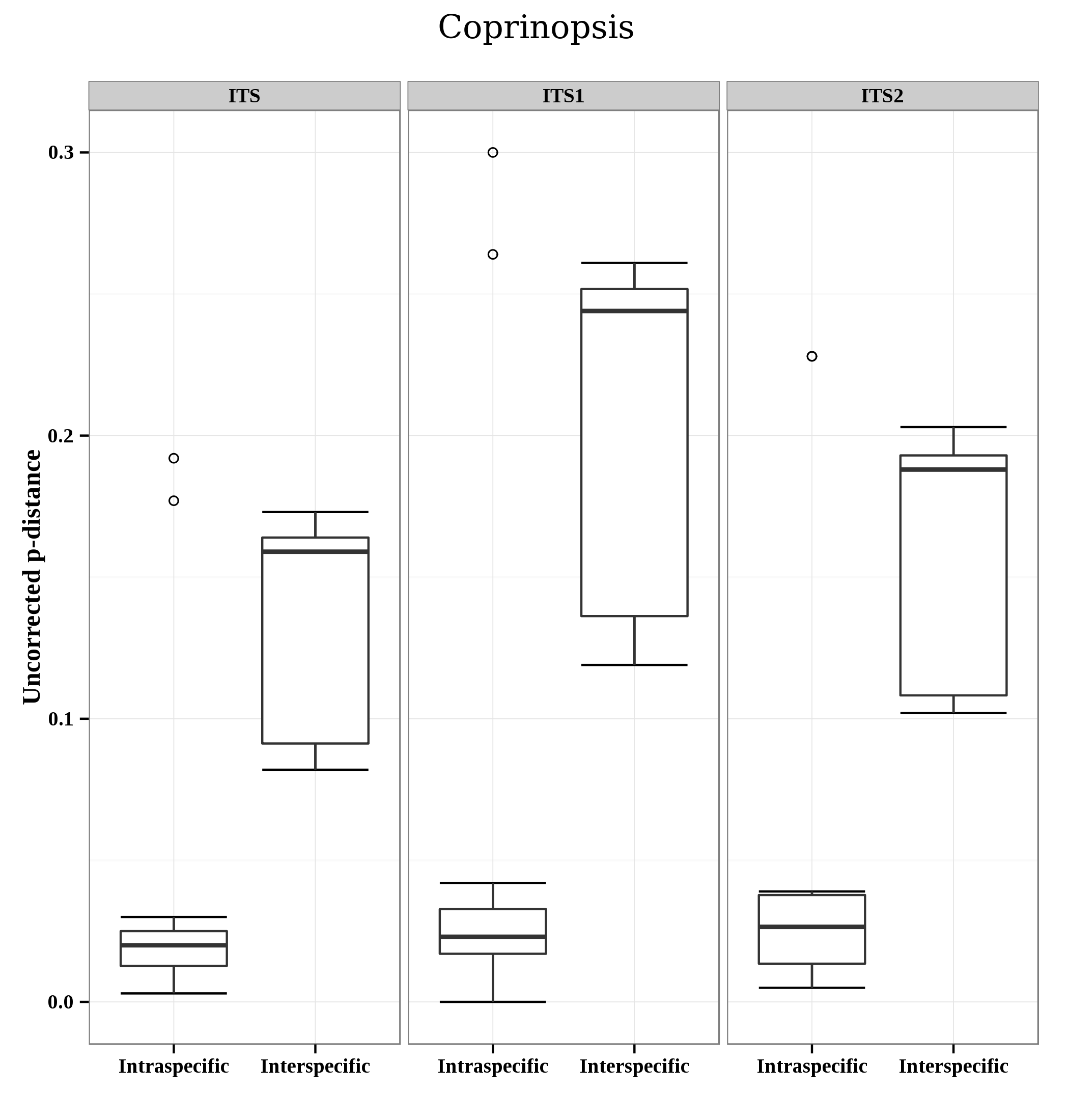

Supplement: Additional file 5: — Barcode gap of all the 113 genera studied for ITS, ITS1 and ITS2 genomic regions by plotting intra- and interspecific distances. (DOCX 9035 kb) [file 12866_2017_958_MOESM5_ESM.docx]
